# New Hybrid Tetrahydropyrrolo[3,2,1-*ij*]quinolin-1-ylidene-2-thioxothiazolidin-4-ones as New Inhibitors of Factor Xa and Factor XIa: Design, Synthesis, and In Silico and Experimental Evaluation

**DOI:** 10.3390/molecules28093851

**Published:** 2023-05-01

**Authors:** Nadezhda P. Novichikhina, Alexander S. Shestakov, Svetlana M. Medvedeva, Anna M. Lagutina, Mikhail Yu. Krysin, Nadezhda A. Podoplelova, Mikhail A. Panteleev, Ivan S. Ilin, Alexey V. Sulimov, Anna S. Tashchilova, Vladimir B. Sulimov, Athina Geronikaki, Khidmet S. Shikhaliev

**Affiliations:** 1 Department of Organic Chemistry, Faculty of Chemistry, Voronezh State University, Universitetskaya pl. 1, 394018 Voronezh, Russia; novichikhina@chem.vsu.ru (N.P.N.);; 2 Dmitry Rogachev National Medical Research Center of Pediatric Hematology, Oncology and Immunology, 117997 Moscow, Russia; 3 Center for Theoretical Problems of Physicochemical Pharmakology, 119991 Moscow, Russia; 4 Dimonta, Ltd., 117186 Moscow, Russia; 5 Research Computing Center, Lomonosov Moscow State University, 119992 Moscow, Russia; 6 School of Pharmacy, Aristotle University of Thessaloniki, 54124 Thessaloniki, Greece

**Keywords:** 5,6-dihydro-4*H*-pyrrolo[3,2,1-*ij*]quinoline-1,2-dione, rhodanines, anticoagulant activity, docking studies

## Abstract

Despite extensive research in the field of thrombotic diseases, the prevention of blood clots remains an important area of study. Therefore, the development of new anticoagulant drugs with better therapeutic profiles and fewer side effects to combat thrombus formation is still needed. Herein, we report the synthesis and evaluation of novel pyrroloquinolinedione-based rhodanine derivatives, which were chosen from 24 developed derivatives by docking as potential molecules to inhibit the clotting factors Xa and XIa. For the synthesis of new hybrid derivatives of pyrrolo[3,2,1-*ij*]quinoline-2-one, we used a convenient structural modification of the tetrahydroquinoline fragment by varying the substituents in positions 2, 4, and 6. In addition, the design of target molecules was achieved by alkylating the amino group of the rhodanine fragment with propargyl bromide or by replacing the rhodanine fragment with 2-thioxoimidazolidin-4-one. The in vitro testing showed that eight derivatives are capable of inhibiting both coagulation factors, two compounds are selective inhibitors of factor Xa, and two compounds are selective inhibitors of factor XIa. Overall, these data indicate the potential anticoagulant activity of these molecules through the inhibition of the coagulation factors Xa and XIa.

## 1. Introduction

In the current trend in drug design, hybrid or multimodal structures containing covalently linked pharmacophore groups are prevalent. This combination leads to the possibility of interaction with several protein targets, due to which a synergistic effect is achieved, and it becomes possible to conduct combination therapy using a single multimodal agent and to reduce unwanted side effects (for recent reviews on the chemistry of low-molecular-weight heterocyclic hybrids and their application in medicine, see [1,2,3,4,5,6,7,8,9,10,11]).

**Figure 1:**
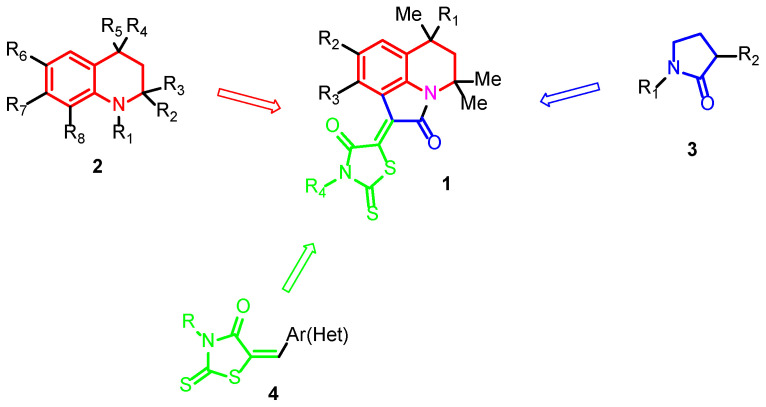
Design of target rhodanine-pyrroloquinoline-2-ones.

The goal of molecular hybridization set in this work was to obtain compounds **1** (Figure 1), which, according to our preliminary data [12], are effective inhibitors of the blood-coagulation factors Xa and XIa. The hope that sufficiently strong anticoagulants can be obtained in this way is based on the fact that for each of the building blocks, derivatives are known that have pronounced inhibitory activities against blood-coagulation factors.

Tetrahydroquinoline **2** derivatives inhibit factors Xa [13,14] and XIa [15,16], pyrrolidin-2-one **3**, factor Xa [17], and rhodanine **4**, factor VIII [18]. Previously, as a result of the hybridization of **2** and **3**, we obtained derivatives of 5,6-dihydro-4*H*-pyrrolo[3,2,1-*ij*]quinoline-1,2-diones, which exhibited inhibitory activity against factors Xa [19] and XIIa [20]. The introduction of the rhodanine fragment into the composition of the closest nonhydrogenated analog of the hybridization products **2** and **3** makes it possible to obtain factor Xa inhibitors (IC_50_~1 μM) [21].

The introduction of rhodanine into the structure of potential physiologically active compounds is associated with the risk of false-positive results in screening studies [22,23,24]. However, epalrestat containing a rhodanine fragment was successfully used to treat diabetes mellitus [25]. For compounds containing fragments of rhodanine, antibacterial activity [26,27,28,29,30,31], fungicidal [26,32], antiviral [30], anti-tuberculosis [33], anti-inflammatory [26,27,34], and anti-cancer [26,27,30,35,36,37] activities were noted. These compounds can be used in the treatment of diabetes mellitus [25,27], Alzheimer’s disease [26,27], and a number of other diseases [38,39]. Rhodanine and its derivatives are widely used in molecular hybridization. Methods for obtaining conjugates of rhodanine with benzothiophene [34,40], benzofuran [40], pyrazoles [41], quinoline [42], carbazole [43], thienopyrimidine [44], carbohydrates [37], benzyloxyarylidene [45], tetrazolquinoline [46], and biaryloxazolidinone [47] have been described.

Bioisosteric substitution is one of the most important strategies in the structural optimization of lead compounds for the development of new drugs. In particular, the introduction of halogen atoms into the molecule can significantly improve the pharmacokinetics, lipophilicity, and biological activity in general. On the other hand, halogen atoms are also the preferred structural moieties of anticoagulants due to their electron-withdrawing properties [48,49,50,51].

Thus, in this work, we continue the study of the anticoagulant activity of new hybrid molecules based on 5,6-dihydro-4*H*-pyrrolo[3,2,1-*ij*]quinoline-1,2-diones and rhodanines, further developing our findings published in [19,20,21]. A virtual library was constructed and screened by docking, the best selected hybrid molecules were synthesized, and their anticoagulant activities were evaluated against factors Xa and XIa.

## 2. Results

Molecular-docking prediction.

In the search for new inhibitors of the coagulation factors Xa and XIa, a virtual library was created, containing 543 new hybrid molecules of compounds that can be synthesized. For this virtual library, we developed a convenient synthesis of new hybrid tetrahydropyrrolo[3,2,1-*ij*]quinolin-1-ylidene-2-thioxothiazolidin-4-ones through the condensation of polyfunctional pyrrolo[3,2,1-*ij*]quinolindiones with rhodanine and 2-thioxoimidazolidin-4-one. All the molecules from this library were docked into the coagulation factors Xa and XIa using the SOL docking program, and twenty-four new hybrid molecules based on 5,6-dihydro-4H-pyrrolo[3,2,1-*ij*]quinolin-1,2-diones and rhodanines were selected, using the docking results, as the best inhibitor candidates.

The selection of the best inhibitors was made using the threshold values of the SOL score and the reliability of the docking. The threshold scores separating the good inhibitors from the inactive compounds were determined by docking native ligands and known inhibitors of the Xa and XIa factors. The atomistic models of the factors Xa and XIa used in the docking were constructed from high-quality PDB structures, 3CEN and 4CRC, respectively. The docking reliability was determined by clustering the solutions of the genetic algorithm used in the SOL program. The docking with 50 independent runs of the algorithm was considered successful if the population of the first cluster containing the most energetically favorable positions of the ligand was greater than 10. Otherwise, the docking was considered unsuccessful, and the ligands were not included in the pool of perspective inhibitors. The docking of native ligands from the 3CEN and 4CRC complexes into the corresponding proteins resulted in RMSD < 1.4 Å between the crystallized- and docked-ligand positions.

### 2.1. Synthesis

The 6-Methyl-5,6-dihydro-4*H*-pyrrolo[3,2,1-*ij*]quinoline-1,2-dione [52,53] with various substituents in positions 4, 6, and 8 was used as the initial template. Therefore, in position 4, there were two methyl groups or an alicyclic fragment, while in position 6, along with the methyl group, there was another methyl group or a benzene ring with or without a substituent; alkyl, alkoxy, and acyloxy substituents or halogen atoms were in position 8.

**Scheme 1:**
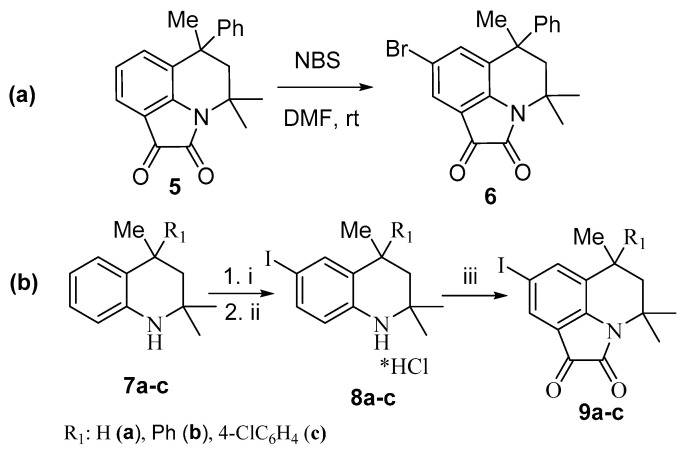
Preparation of 8-bromo-pyrrolo[3,2,1-*ij*]quinoline-1,2-dione (**a**) and 8-iodo-substituted pyrrolo[3,2,1-*ij*]quinoline-1,2-diones (**b**). Reagents and conditions: (i) I_2_, dioxane:pyridine (1:1); (ii) HCl; (iii) (COCl)_2_, PhCH_3_, reflux, 2 h.

The synthesis of 8-halogen-substituted pyrroloquinolinediones is shown in Scheme 1. The bromination of 6-methyl-6-phenyl-5,6-dihydro-4*H*-pyrrolo[3,2,1-*ij*]quinoline-1,2-dione **5** was carried out according to the procedure described in [54], using *N*-bromosuccinimide as a brominating agent and DMF as a solvent. To obtain 8-iodo-substituted pyrrolo[3,2,1-*ij*]quinoline-1,2-diones, the initial substrates, 2,2,4-trimethyl-1,2,3,4-tetrahydroquinolines, were subjected to iodination, in accordance with the procedure presented in [55]. The subsequent reaction of 6-iodo-2,2,4-trimethyl-4-R-1,2,3,4-tetrahydroquinoline hydrochlorides **8a**–**c** with oxalyl chloride gave the corresponding diones **9a**–**c** (Scheme 1).

**Scheme 2:**
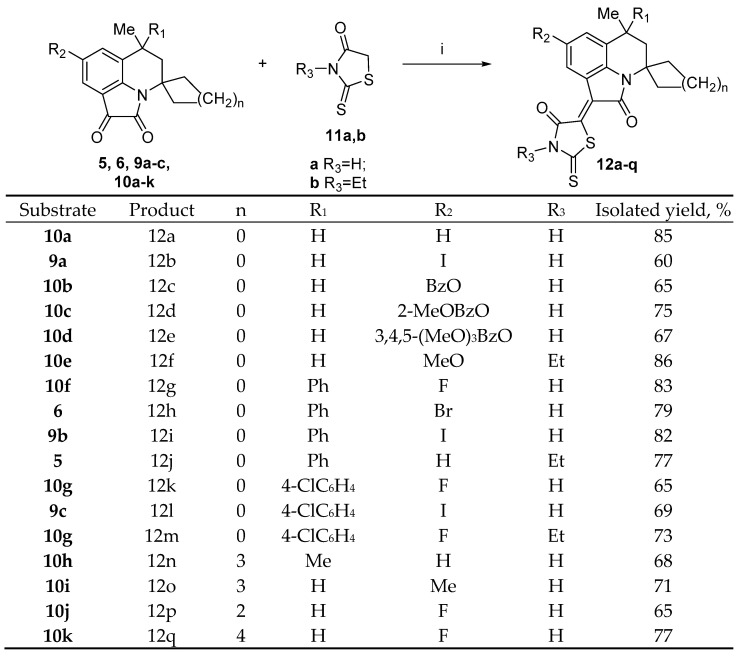
Synthesis of the compounds **12a**–**q**. Reagents and conditions: (i) AcOH, AcONa, and reflux, 1–6 h.

To obtain new hybrid molecules based on 6-methyl-5,6-dihydro-4*H*-pyrrolo[3,2,1-*ij*]quinoline-1,2-diones, they were introduced into the condensation reaction with rhodanines **11a**,**b** at reflux in an acetic-acid medium in the presence of freshly melted sodium acetate (Scheme 2).

**Scheme 3:**
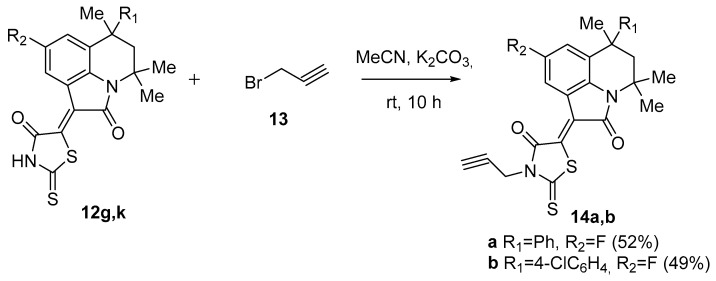
Synthesis of compounds **14a**,**b**.

The compounds **12g** and **12k** containing a free NH group in the rhodanine moiety were further involved in alkylation reactions with propargyl bromide **13** (Scheme 3).

**Scheme 4:**
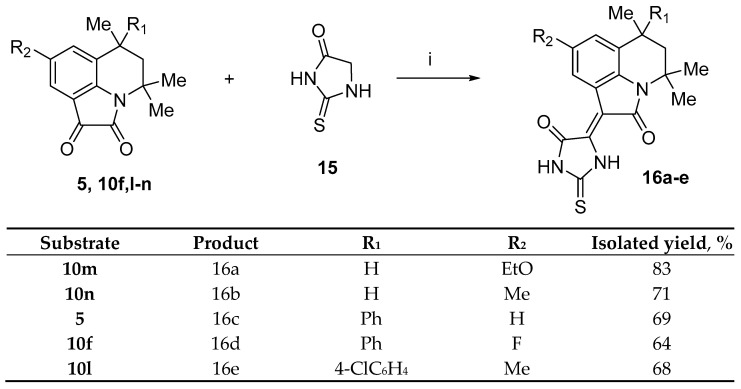
Synthesis of compounds **16a**–**e**. Reagents and conditions: (i) AcOH, AcONa, and reflux, 4–10 h.

As a structural analog of rhodanine, 2-thioxoimidazolidin-4-one **15** was used in reaction with 5,6-dihydro-4*H*-pyrrolo[3,2,1-*ij*]quinoline-1,2-diones **5**, **10** (Scheme 4).

### 2.2. Anticoagulant Studies

**Figure 2:**
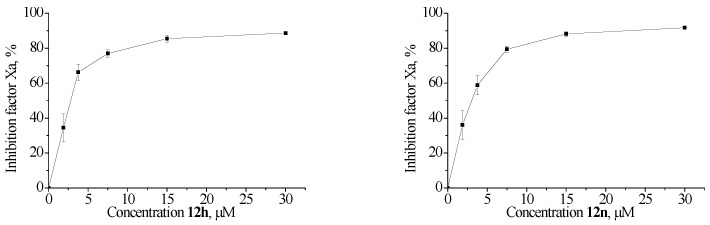
The percentage of inhibition for the hydrolysis rate of factor-Xa-specific chromogenic substrate in buffer solution in the presence of different concentrations of inhibitors.

**Figure 3:**
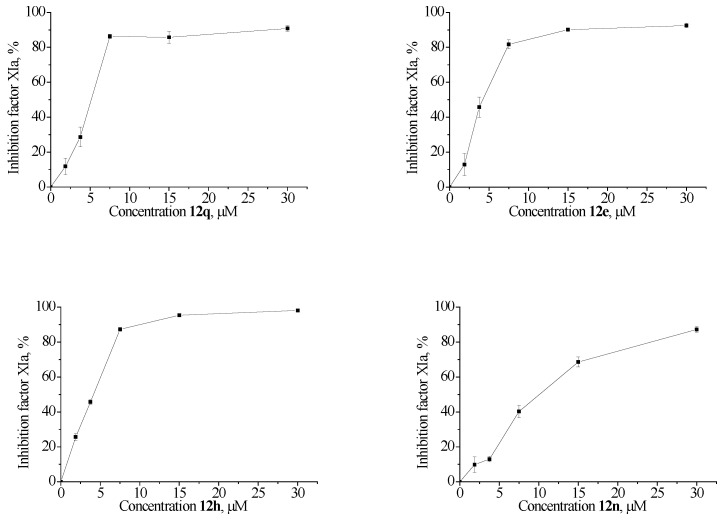
The percentage of inhibition for the hydrolysis rate of factor-XIa-specific chromogenic substrate in buffer solution in the presence of different concentrations of inhibitors.

Compounds **12**, **14** and **16** were evaluated for their anticoagulant activity against factors Xa and Xia, and the results are presented in Table 1. For the best Xa and XIa inhibitors, additional measurements were performed to determine the IC_50_ values. We measured the kinetics of the hydrolysis of the specific substrates S2765 or S2366 by factors Xa and XIa, respectively, in the buffer solution and in the presence of various concentrations of compounds. The results are presented in Figure 2 and Figure 3.

**Figure 4:**
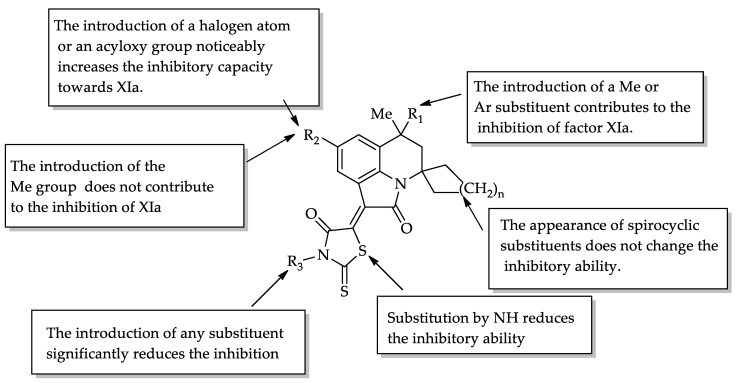
Structure-activity relationship.

It was found that the compounds exhibited anticoagulant activity against factor Xa and XIa with a percentage inhibition in the range of 2–99% with inhibitors of 30 μM. It should be mentioned that of the 24 tested compounds, compounds **12d**, **12g**, **12h**, **12k**, **12l**, and **12n** showed good activity against factor Xa, with the IC_50_ ranging from 2.28 to 3.70 μΜ; compound **12h** showed the best activity. On the other hand, compounds **12d**, **12e**, **12h**, **12l**, and **12q** displayed good activity against factor Xia, with the IC_50_ in the range of 3.73–4.32 μΜ. Comparing the data in Table 1, we can conclude that factor XIa is much more sensitive to structural changes than factor Xa. A comparison of the data on the inhibition at a concentration of 30 μM and at IC_50_ allowed us to trace the following patterns (Figure 4):

− The introduction of a methyl or aryl substituent at position 6, in addition to the methyl substituent, contributes to the inhibition of factor XIa (compare **12n** with **12o** and **12a**);
− The appearance of a methyl substituent in position 8 reduces the inhibitory ability of factor XIa (**12o** and **12p**);
− The appearance of a halogen atom (especially fluorine) at position 8 significantly increases the inhibitory ability (compounds **12g**, **12h**, **12i**, **12k**, and **12l**), but this effect is eliminated by the appearance of substituents in the nitrogen atom of rhodanine (compounds **12m**, **14a**, and **14b**);
− The introduction of acyloxy substituents at position 8 supports the manifestation of its inhibition of factor XIa (compounds **12c**, **12d**, and **12e**);
− The appearance of substituents in the nitrogen atom of rhodanine significantly reduces the inhibitory ability (compounds **12f**, **12m**, **14a**, and **14b**);
− The replacement of rhodanine by 2-thioxoimidazolidin-4-one leads to a decrease in the inhibitory ability (compounds **16a**, **16b**, and **16d**).

### 2.3. Molecular Docking

**Figure 5:**
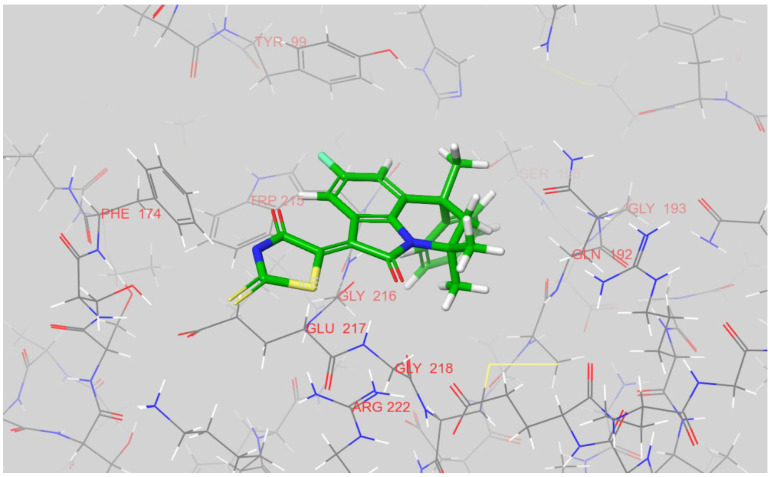
The docked pose of **12k** inside the active site of factor Xa. The figure was prepared in Maestro.

According to the predicted binding modes, all three compounds, **12k**, **12g**, and **12n**, block the access to the catalytic triad of factor Xa. In its docked pose, the **12k** places its chloro-phenyl fragment inside a S1 pocket of the enzyme while the rhodanine moiety points toward the solvent and is located near a positively charged side chain of Arg-222 (see Figure 5). A hydrophobic S4 pocket is left unoccupied.

**Figure 6:**
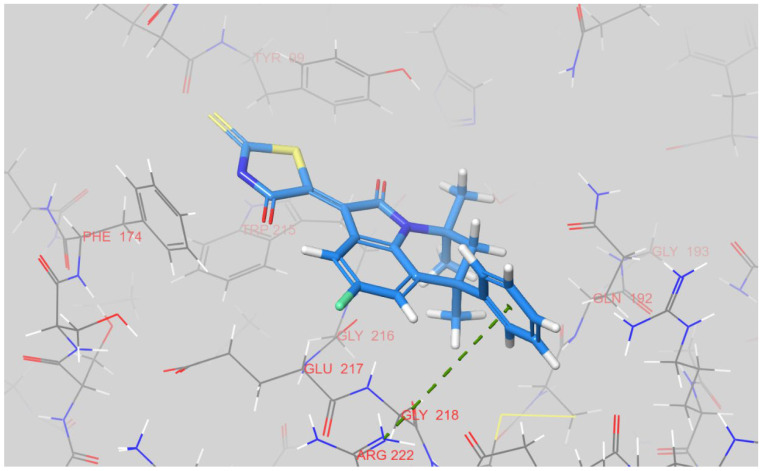
The docked pose of **12g** inside an active site of factor Xa. A green dashed line indicates pi-cation interaction between phenyl ring of a ligand and guanidine group of Arg-222. The figure was prepared in Maestro.

Despite high level of similarity between **12k** and **12g**, the latter binds to factor Xa in a different manner (Figure 6). Its rhodanine ring is placed between two aromatic residues, Tyr-99 and Phe-174, occupying the S4 pocket. A S1 pocket is almost unoccupied, with only a methyl group placed at its top. A phenyl ring is located outside the active site and it is engaged into pi-cation interaction with Arg-222. Taking this high degree of similarity into account, we assume that although they are different geometrically, both energy minima observed for **12k** and **12g** are very similar energetically. Both the conformations depicted on Figure 4 and Figure 5 can represent the relevant binding modes for both **12k** and **12g**.

**Figure 7:**
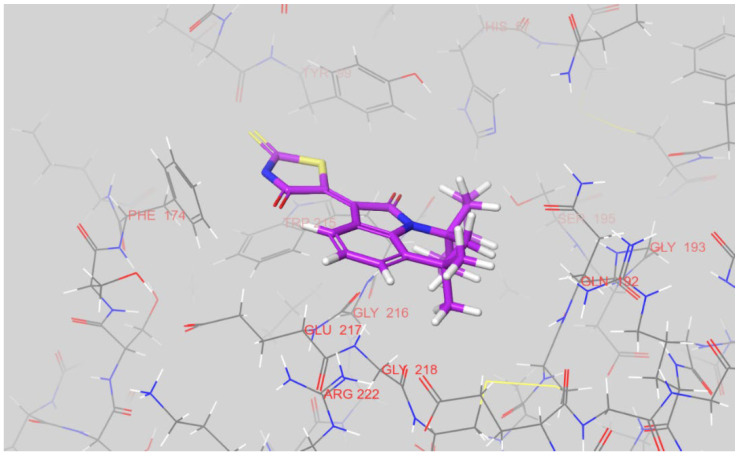
The docked pose of **12n** inside an active site of factor Xa. The figure was prepared in Maestro.

The predicted conformation of **12n** is very similar to the docked pose of **12g** (Figure 7). The rhodanine moiety of **12n** is similarly positioned between Tyr-99 and Phe-174, inside S4 pocket. The cyclohexene ring of the spiro-system is located at the top of the S1 pocket, and does not extend inside. The S1 pocket is, therefore, left almost unoccupied. In summary, the rhodanine-containing factor-Xa inhibitors studied in the present work possess two distinct conformations in terms of the position of the rhodanine moiety. This moiety can be found either inside the S4 pocket, sandwiched between the aromatic rings of Tyr-99 and Phe-174, or pointing toward the solvent and located near a guanidine fragment of Arg-222. To profile the residues involved in binding for the rest of compounds, we calculated all the intermolecular interactions for each ligand with residues of the active site using the MMFF94 force field. An amino-acid residue was not included in the table of interactions for a given ligand if there was not a single ligand interaction with its atoms equal to or greater than 0.5 kcal/mol. The identified interaction residues of factor Xa for all the tested compounds can be found in Table S1.

Comparing the binding modes of **12g**, **12k**, and **12n** with the bound geometry of rivaroxaban, a subnanomolar factor-Xa inhibitor, the three identified inhibitors were found to lack the two hydrogen bonds observed in the acyclicamide and cyclic-carbamate parts of the rivaroxaban, which interact with the backbone oxygen and backbone nitrogen of Gly-219, respectively. Similar to **12k**, rivaroxaban occupies the S1 pocket through a hydrophobic aromatic ring, but it is buried more deeply in the protein. Unlike **12g** and **12n**, the rivaroxaban tail group lying in the S4 pocket is placed orthogonally, not in the same plane as the central part of the molecule, and it is not charged. The absence of the interactions described above can be exploited to obtain additional potency for inhibitors that are found during optimization studies in the future.

**Figure 8:**
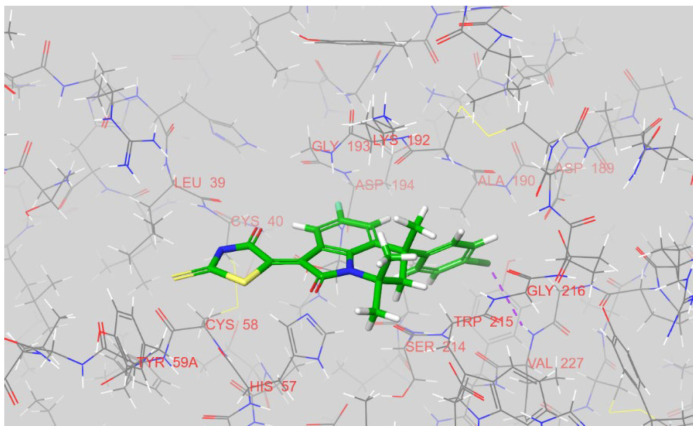
The docked pose of **12k** inside an active site of factor XIa. The dashed purple line indicates a halogen atom between a chlorine atom of the ligand and a backbone nitrogen of Val-227. The figure was prepared in Maestro.

As in the case of factor Xa, **12k**, **12g**, and **12n** were observed to have two distinct binding conformations after docking into factor Xia, with respect to the location of the rhodanine ring. The first was found for **12k**, where the rhodanine cycle is positioned inside a S1’ pocket near His-57 (see Figure 8). The chloro-phenyl fragment of **12k** is buried in a S1 pocket, with a halogen bond between a chlorine atom and Val-227NH observed. A S2’ pocket that is able to engage basic moieties is left unoccupied.

**Figure 9:**
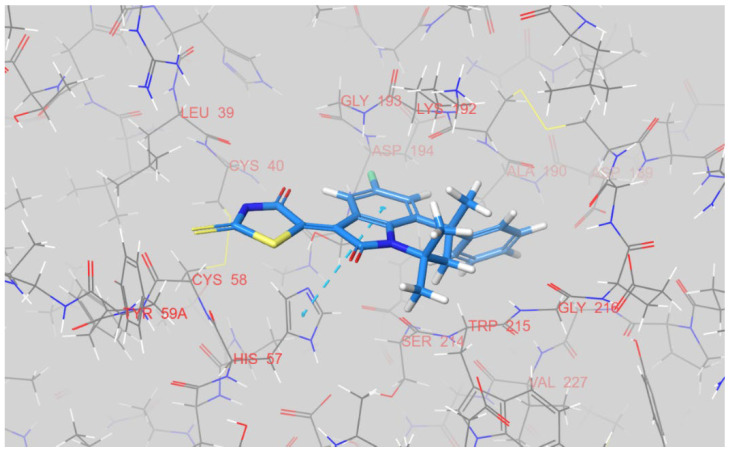
Predicted binding mode of **12g** with factor XIa. The dashed blue line indicates pi-stacking between the ligand central core and His-57. The figure was prepared in Maestro.

The **12g** binds to factor XIa in a similar manner to **12k** (Figure 9). Again, the rhodanine ring is found in a S1’ pocket, and the phenyl ring is deeply inserted in a S1 pocket. However, since **12g** lacks a chlorine atom, no halogen bonds are observed for a S1 pocket. The favourable position of the central scaffold provides pi-stacking between a fluoro-phenyl ring and His-57 instead. Similar to **12k**, **12g** does not occupy a S2’ pocket.

**Figure 10:**
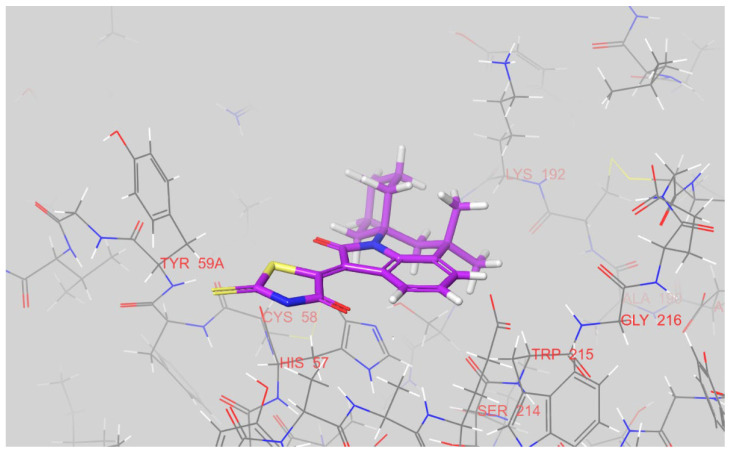
The docked pose of **12n**. The figure was prepared in Maestro.

The docked pose of **12n** differs from the poses of the aforementioned compounds and is depicted in Figure 10. The rhodanine moiety is predicted to be near the edge of the factor-XIa active site inside a S2 pocket. In general, it is not common for existing factor-XIa inhibitors to occupy this pocket. A cyclohexene ring of **12n** partly fills a S1’ pocket. A S1 pocket is almost unoccupied, with only a methyl group placed at its top. No specific interactions are visually observable. The residues that participate in interactions with ligands for the remainder of the tested compounds are outlined in Supplementary Materials Table S1.

We can conclude that, as in the case of binding to factor Xa, the rhodanine moiety does not tend to be buried in deep pockets of factor XIa. For both coagulation factors, a hydrophilic and charged rhodanine ring can instead be found in open solvent-exposed pockets and surfaces, decreasing the likelihood of a desolvation penalty.

#### ADME Properties

The ADME properties of all the compounds are presented in Table 2. One of the most significant issues is how well a drug is absorbed from an oral solution. In this case, all the compounds showed high levels of gastro-internal absorption.

Moreover, the positive values of all the compounds, except for the compounds **12a** and **12b** and the reference drug, Rivaroxaban, showed that they can be transported across the cell membrane by the ATP-binding cassette (ABC) transporter, which is a component of P-glycoprotein. By contrast, the compounds **12a**–**12q** and **14a**–**14b** and the reference drug, Rivaroxaban, were also predicted to be P-glycoprotein I and II inhibitors, which can inhibit their transport. Regarding skin permeability, if the logKp is greater than −2.5, a drug cannot be skin-permeable. In this framework, all the compounds demonstrated improved skin permeability.

The volume of distribution (VDss) suggests the total amount of drug that needs to be homogeneously distributed in the blood. The VDss values are considered low if they are below 0.15 log VDss, and high VDss values are >0.45 log VDss. Therefore, only compounds **12e**, **16a**, and **16b** and Rivaroxaban can be regarded as low-VDss compounds. The permeability of the blood–brain barrier (BBB) indicates whether a substance can enter the brain. Since it is assumed that compounds with logBB > 0.3 can pass through the BBB, all the compounds’ logBB values indicate a low ability to pass through the BBB. Similarly, the majority of the compounds have low central nervous system (CNS) permeability, except for compounds **12e** and **12o** and Rivaroxaban, whose logPS values are lower than −2.0.

The metabolism prediction suggested that most of the compounds are both substrates and inhibitors of CYP3A4. The maximum tolerated dose (MRTD) is a crucial criterion in toxicity evaluation; a value of less than 0.44 log(mg/kg/day) is considered low, while a value of more than 0.477 log(mg/kg/day) is considered high. Thus, none of the compounds showed an optimal MRTD. Moreover, most of the compounds showed no AMES toxicity and no hepatotoxicity. They ae therefore considered non-toxic, except for compounds **12d** and **12g**–**i**, as well as the reference drug, Rivaroxaban.

## 3. Materials and Methods

The ^1^H- and ^13^C-NMR spectra were recorded on Bruker DRX-500 (500.13 and 125.76 MHz, respectively), Bruker AV 600, and Bruker AM 300 (600.13, 300.13 and 150.90 MHz, respectively) in DMSO-*d*_6_, with TMS as internal standard. The IR spectra of solid samples were recorded on a Vertex 70 FT-IR spectrometer using a Platinum ATR (Bruker) attachment equipped with a diamond prism in a frequency range from 4000 to 400 cm^−1^, with a resolution of 2 cm^−1^. The results were obtained by averaging 16 scans. High-resolution mass spectra were recorded on an Agilent Technologies LCMS 6230B spectrometer (electrospray ionization). Melting points were determined on a Stuart SMP30 apparatus. Assaying of the purity of the starting materials and the synthesized compounds, as well as the analysis of reaction mixtures, was conducted by TLC on Merck TLC silica-gel 60 F_254_ plates. Eluents: CHCl_3_, petroleum ether, ethyl acetate, and their mixtures, in different ratios. Visualization of TLC plates was prepared under UV light or in iodine vapor. Commercially available solvents and reagents (Sigma-Aldrich, Merck, Acros Organics) were used in this work.

### 3.1. Synthesis

Procedure for the synthesis of 8-bromo-4,4,6-trimethyl-6-phenyl-5,6-dihydro-4*H*-pyrrolo[3,2,1-*ij*]quinoline-1,2-dione **6**.

The following procedure was adapted from previously published research [54]. A mixture of 4,4,6-trimethyl-6-phenyl-5,6-dihydro-4*H*-pyrrolo[3,2,1-*ij*]quinoline-1,2-dione (1 g, 3.28 mmol) and *N*-bromosuccinimide (0.64 g, 3.61 mmol) in DMF (20 mL) was stirred at room temperature for 12 h. The mixture was diluted with water (40 mL). The resulting precipitate was filtered, washed in water, dried, and recrystallized from *2*-PrOH.

Red powder, 1.18 g; yield 94 %; R*_f_* = 0.27 (chloroform); m.p. 168–170 °C; IR (KBr, ν_max_/cm^−1^): 1729 (C=O), 1295, 775, 707 (C-Br), 468; ^1^H NMR (500.13 MHz, DMSO-*d*_6_), δ (ppm): 0.75 (3H, s, C^6^-CH_3_); 1.59 (3H, s, C^4^-CH_3_); 1.71 (3H, s, C^4^-CH_3_); 2.09 (1H, d, *J* = 14.4 Hz, C^5^-H); 2.45 (1H, d, *J* = 14.4 Hz, C^5^-H); 7.14 (2H, d, *J* = 7.8 Hz, Ph); 7.20 (1H, t, *J* = 7.2 Hz, Ph); 7.29 (2H, t, *J* = 7.6 Hz, Ph); 7.65 (1H, s, H-7(9)); 7.77 (1H, s, H-7(9)); ^13^C NMR (125.76 MHz, DMSO-*d*_6_), δ (ppm*):* 24.9, 27.7, 30.2, 39.7, 50.3, 53.9, 114.9, 118.2, 125.2, 126.4, 126.6, 128.3, 130.0, 138.0, 146.5, 147.1, 156.4, 182.8. HPLC-HRMS-ESI, *m*/*z* ([M + H]^+^), calcd for C_20_H_18_BrNO_2_ + H^+^ 384.0595, found 384.0598.

General procedure for the synthesis of 6-iodo-2,2,4-trimethyl-4-*R*_1_-1,2,3,4-tetrahydroquinoline hydrochloride **8a**–**c**.

The following procedure was adapted from previously published research [55]. To a solution of corresponding 2,2,4-trimethyl-1,2,3,4-tetrahydroquinoline **7** (0.01 mol) in mixture of dioxane:pyridine (1:1, 500 mL) at 0–5 °C was added molecular iodine (0.03 mol) in small portions, and mixture was subjected to constant stirring for 20–30 min. After 30 min, the ice bath was removed and the mixture was allowed to reach room temperature after 1 h. Progress of the reaction was monitored by thin-layer chromatography (TLC) on SiO_2_ with 10:1 petroleum ether–ethyl acetate as the eluent and visualization by iodine vapor and UV radiation. After the completion of the conversion, the reaction mixture was quenched by addition of saturated aqueous solution of Na_2_S_2_O_3_ until complete discoloration of the solution. The resulting solution was extracted with CH_2_Cl_2_ (3 × 10 mL); the combined organic layers were washed with saturated brine, dried over anhydrous Na_2_SO_4_, and concentrated under vacuo at 50–60 °C. The oily residue was dissolved in minimal amounts of acetone, and concentrated HCl (0.01 mol, 1 mL) was added dropwise under stirring. The precipitate formed was filtered off and ether was washed.

*6-Iodo-2,2,4-trimethyl-1,2,3,4-tetrahydroquinoline hydrochloride* (**8a**). White powder, 1.78 g; yield 59%; R*_f_* = 0.48 (5:1 petroleum ether/ethyl acetate); m.p. 125–127 °C; ^1^H NMR (600.13 MHz, DMSO-*d*_6_), δ (ppm): 1.02–1.22 (9H, set of singlets, C^4^-CH_3_ +2 C^2^-CH_3_); 1.63 (1H, dd, *J* = 12.5 Hz, *J* = 5.5 Hz, C^3^-H); 1.69 (1H, dd, *J* = 12.5 Hz, *J* = 5.5 Hz, C^3^-H); 2.72–2.82 (1H, m, C^4^-H); 6.27–7.39 (3H, CH_arom_); ^13^C NMR (150.90 MHz, DMSO-*d*_6_), δ (ppm): 20.5, 27.4, 27.8, 30.9, 43.9, 48.7, 84.9, 116.7, 126.9, 134.9, 135.1, 144.4. HPLC-HRMS-ESI, *m/z* ([M + H]^+^), calcd for C_12_H_16_IN + H^+^ 302.0414, found 302.0412.

*6-Iodo-2,2,4-trimethyl-4-phenyl-1,2,3,4-tetrahydroquinoline hydrochloride* (**8b**). White powder, 2.75 g; yield 73%; R*_f_* = 0.49 (5:1 petroleum ether/ethyl acetate); m.p. 140–142 °C; IR (KBr, ν_max_/cm^−1^): 3324, 3251 (NH), 702, 517; ^1^H NMR (600.13 MHz, DMSO-*d*_6_), δ (ppm): 0.86 (3H, s, C^4^-CH_3_); 1.22 (3H, s, C^2^-CH_3_); 1.64 (3H, s, C^2^-CH_3_); 1.96 (1H, d, *J* = 14.3 Hz, C^3^-H); 2.27 (1H, d, *J* = 14.3 Hz, C^5^-H); 6.80 (1H, d, *J* = 8.5 Hz, H-8); 7.12–7.15 (3H, m, Ph); 7.22–7.25 (2H, m, Ph); 7.32 (1H, s, H-5(7)); 7.47 (1H, d, *J* = 8.5 Hz, H-5(7)); ^13^C-NMR (150.90 MHz, DMSO-*d*_6_), δ (ppm): 28.2, 28.7, 31.5, 40.7, 49.9, 52.1, 84.7, 121.2, 126.4, 127.0, 127.1, 128.6, 134.1, 136.4, 138.1, 150.0. HPLC-HRMS-ESI, *m/z* ([M + H]^+^), calcd for C_18_H_20_IN + H^+^ 378.0713, found 378.0715.

*4-(4-Chlorophenyl)-6-iodo-2,2,4-trimethyl-1,2,3,4-tetrahydroquinoline hydrochloride* (**8c**). White powder, 2.51 g; yield 61%; R*_f_* = 0.55 (5:1 petroleum ether/ethyl acetate); m.p. 146–148 °C; IR (KBr, ν_max_/cm^−1^): 2457, 809, 819, 500; ^1^H NMR (600.13 MHz, DMSO-*d*_6_), δ (ppm): 0.90 (3H, s, C^4^-CH_3_); 1.22 (3H, s, C^2^-CH_3_); 1.64 (3H, s, C^2^-CH_3_); 1.97 (1H, d, *J* = 14.4 Hz, C^3^-H); 2.24 (1H, d, *J* = 14.4 Hz, C^3^-H); 6.83 (1H, d, *J* = 8.5 Hz, H-8); 7.15 (2H, d, *J* = 8.4 Hz, C_6_H_4_); 7.28 (2H, d, *J* = 8.3 Hz, C_6_H_4_); 7.34 (1H, s, H-5(7)); 7.49 (1H, d, *J* = 8.5 Hz, H-7(5)); ^13^C-NMR (150.90 MHz, DMSO-*d*_6_), δ (ppm): 28.0, 28.4, 31.4, 40.5, 49.6, 52.4, 85.7, 121.7, 128.4, 128.6, 129.2, 129.2, 131.1, 131.2, 136.6, 138.0, 149.0. HPLC-HRMS-ESI, *m/z* ([M + H]^+^), calcd for C_18_H_19_ClIN + H^+^ 412.0323, found 412.0327.

*8-R_2_-4,4,6-trimethyl-6-R_1_-5,6-dihydro-4H-pyrrolo[3,2,1-ij]quinoline-1,2-diones* **5**, **9a**–**c**, **10a**–**o** were obtained according to the published method [52,53]. **General procedure**: oxalyl chloride (4.7 mL, 5.5 mmol) was added to corresponding tetrahydroquinoline hydrochloride (5 mmol) in anhydrous toluene (50 mL). The reaction mixture was refluxed for 1.5–2 h until complete dissolution of the salt. Excess solvent was removed under vacuum, and the orange–red precipitate that formed after cooling was filtered off, washed with EtOH, and recrystallized from 2-PrOH.

The physicochemical and spectral data of 4,4,6-trimethyl-6-phenyl-5,6-dihydro-1*H*-pyrrolo[3,2,1-*ij*]quinoline-1,2(4*H*)-dione (**5**), 4,4,6-trimethyl-5,6-dihydro-4*H*-pyrrolo[3,2,1-*ij*]quinoline-1,2-dione (**10a**), 4,4,6-trimethyl-1,2-dioxo-1,2,5,6-tetrahydro-4*H*-pyrrolo[3,2,1-*ij*]quinolin-8-yl benzoate (**10b**), 8-methoxy-4,4,6-trimethyl-5,6-dihydro-4H-pyrrolo[3,2,1-*ij*]quinoline-1,2-dione (**10e**) and 4,4,6,8-tetramethyl-5,6-dihydro-4*H*-pyrrolo[3,2,1-*ij*]quinoline-1,2-dione (**10n**) are described in [52]. The data of 6′,6′-dimethyl-5′,6′-dihydrospiro-[cyclohexane-1,4′-pyrrolo[3,2,1-*ij*]quinoline]-1′,2′-dione (**10h**), 6′,8′-dimethyl-5′,6′-dihydrospiro-[cyclohexane-1,4′-pyrrolo[3,2,1-*ij*]quinoline]-1′,2′-dione (**10i**), 8′-fluoro-6′-methyl-5′,6′ -dihydrospiro[cyclopentane-1,4′-pyrrolo[3,2,1-*ij*]quinoline]-1′,2′-dione (**10j**), and 8′-fluoro-6′-methyl-5′,6′-dihydrospiro[cycloheptane-1,4′-pyrrolo[3,2,1-*ij*]quinoline]-1′,2′-dione (**10k**) are described in [53].

*8-Iodo-4,4,6-trimethyl-5,6-dihydro-4H-pyrrolo[3,2,1-ij]quinoline-1,2-dione* (**9a**). Red powder, 1.28 g; yield 72%; R*_f_* = 0.33 (5:1 petroleum ether/ethyl acetate); m.p. 168–170 °C; IR (KBr, ν_max_/cm^−1^): 3427, 1720 (C=O), 1296, 873, 443; ^1^H NMR (300.13 MHz, DMSO-*d*_6_), δ (ppm): 1.30–1.33 (6H, m, C^6^-CH_3_ + C^4^-CH_3_); 1.52 (1H, t, *J* = 12 Hz, C^5^-H); 1.56 (3H, s, C^4^-CH_3_); 1.84 (1H, dd, *J* = 12 Hz, *J* = 3 Hz, C^5^-H); 2.89 (1H, m, C^6^-H); 7.63 (1H, s, H-7(9)); 7.83 (1H, s, H-7(9)); ^13^C NMR (150.90 MHz, DMSO-*d*_6_), δ (ppm): 18.4, 24.8, 25.7, 26.9, 44.6, 54.3, 86.5, 118.2, 129.9, 130.2, 142.5, 147.5, 156.6, 183.1. HPLC-HRMS-ESI, *m/z* ([M + H]^+^), calcd for C_14_H_14_NIO_2_ + H^+^ 356.0142, found 356.0139.

*8-Iodo-4,4,6-trimethyl-6-phenyl-5,6-dihydro-4H-pyrrolo[3,2,1-ij]quinoline-1,2-dione* (**9b**). Orange powder, 1.81 g; 84%; R*_f_* = 0.25 (5:1 petroleum ether/ethyl acetate); m.p. 231–233 °C; IR (KBr, ν_max_/cm^−1^): 1736 (C=O), 1298, 700. ^1^H NMR (600.13 MHz, DMSO-*d*_6_), δ (ppm): 0.73 (3H, s, C^6^-CH_3_); 1.58 (3H, s, C^4^-CH_3_); 1.69 (3H, s, C^4^-CH_3_); 2.06 (1H, d, *J* = 14.4 Hz, C^5^-H); 2.42 (1H, d, *J* = 14.4 Hz, C^5^-H); 7.13 (2H, d, *J* = 7.8 Hz, Ph); 7.20 (1H, t, *J* = 7.3 Hz Ph); 7.29 (2H, t, *J* = 7.6 Hz Ph); 7.76 (1H, s, H-7(9)); 7.89 (1H, s, H-7(9)); ^13^C-NMR (150.90 MHz, DMSO-*d*_6_), δ (ppm): 24.8, 27.6, 30.0, 39.3, 50.1, 53.7, 85.9, 118.4, 126.2, 126.4, 128.2, 130.0, 130.5, 143.4, 146.7, 147.0, 156.0, 182.5. HPLC-HRMS-ESI, *m/z* ([M + H]^+^), calcd for C_20_H_18_NIO_2_ + H^+^ 432.0455, found 432.0457.

*6-(4-Chlorophenyl)-8-iodo-4,4,6-trimethyl-5,6-dihydro-4H-pyrrolo[3,2,1-ij]quinoline-1,2-dione* (**9c**). Orange powder, 1.70 g; yield 73%; R*_f_* = 0.41 (5:1 petroleum ether/ethyl acetate); m.p. 220–222 °C; ^1^H NMR (600.13 MHz, DMSO-*d*_6_), δ (ppm): 0.76 (3H, s, C^6^-CH_3_); 1.58 (3H, s, C^4^-CH_3_); 1.69 (3H, s, C^4^-CH_3_); 2.07 (1H, d, *J* = 14.5 Hz, C^5^-H); 2.41 (1H, d, *J* = 14.5 Hz, C^5^-H); 7.16 (2H, d, *J* = 8.4 Hz, CH_arom_); 7.35 (2H, d, *J* = 8.4 Hz, CH_arom_); 7.77 (1H, s, H-7(9)); 7.90 (1H, s, H-7(9)); ^13^C-NMR (150.90 MHz, DMSO-*d*_6_), δ (ppm): 24.9, 27.5, 29.9, 39.1, 49.9, 53.6, 86.1, 118.5, 128.1, 128.5, 129.5, 130.7, 130.9, 143.3, 146.1, 146.6, 156.1, 182.5. HPLC-HRMS-ESI, *m/z* ([M + H]^+^), calcd for C_20_H_17_ClINO_2_ + H^+^ 466.0065, found 466.0063.

*4,4,6-Trimethyl-1,2-dioxo-1,2,5,6-tetrahydro-4H-pyrrolo[3,2,1-ij]quinolin-8-yl-2-methoxybenzoate* (**10c**). Red solid, 1.53 g; yield 81%; R*_f_* = 0.43 (5:1 petroleum ether/ethyl acetate); m.p. 165–167 °C; ^1^H NMR (600.13 MHz, DMSO-*d*_6_), δ (ppm): 1.32 (3H, d, *J* = 6.7 Hz, C^6^-CH_3_); 1.37 (3H, s, C^4^-CH_3_); 1.58 (1H, t, *J* = 12.9 Hz, C^5^-H); 1.70 (3H, s, C^4^-CH_3_); 1.89 (1H, dd, *J* = 13.7 Hz, *J* = 4.5 Hz, C^5^-H); 2.90–2.96 (1H, m, C^6^-H); 3.88 (3H, s, MeO); 7.10 (1H, t, *J* = 7.5 Hz, CH_arom_); 7.23 (1H, d, *J* = 8.4 Hz, CH_arom_); 7.28 (1H, s, CH_arom_); 7.46 (1H, s, CH_arom_); 7.64–7.66 (1H, m, CH_arom_); 7.94 (1H, d, *J* = 7.4 Hz, CH_arom_); ^13^C-NMR (150.90 MHz, DMSO-*d*_6_), δ (ppm): 17.9, 24.2, 25.3, 26.5, 44.2, 53.7, 55.8, 112.6, 115.7, 116.0, 118.2, 120.0, 127.7, 127.9, 131.5, 134.6, 145.1, 146.1, 156.8, 158.9, 163.8, 183.3. HPLC-HRMS-ESI, *m/z* ([M + H]^+^), calcd for C_22_H_21_NO_5_ + H^+^ 380.1494, found 380.1490.

*4,4,6-Trimethyl-1,2-dioxo-1,2,5,6-tetrahydro-4H-pyrrolo[3,2,1-ij]quinolin-8-yl 3,4,5-trimethoxybenzoate* (**10d**). Orange solid, 1.69 g; yield 77%; R*_f_* = 0.50 (5:1 petroleum ether/ethyl acetate); m.p. 213–215 °C; m.p. 165–167 °C; ^1^H NMR (600.13 MHz, DMSO-*d*_6_), δ (ppm): 1.32 (3H, d, *J* = 6.7 Hz, C^6^-CH_3_); 1.37 (3H, s, C^4^-CH_3_); 1.58 (1H, t, *J* = 13.0 Hz, C^5^-H); 1.71 (3H, s, C^4^-CH_3_); 1.89 (1H, dd, *J* = 13.7 Hz, *J* = 4.5 Hz, C^5^-H); 2.91–2.95 (1H, m, C^6^-H); 3.78 (3H, s, MeO); 3.87 (6H, s, 2MeO); 7.31 (1H, d, *J* = 1.0 Hz, CH_arom_); 7.39 (2H, s, CH_arom_); 7.51 (1H, s, CH_arom_); ^13^C-NMR (150.90 MHz, DMSO-*d*_6_), δ (ppm): 18.0, 24.3, 25.4, 26.5, 44.3, 53.8, 56.1, 60.2, 107.2, 115.7, 116.0, 123.7, 127.8, 128.0, 142.4, 145.2, 146.3, 152.8, 156.9, 164.4, 183.4. HPLC-HRMS-ESI, *m/z* ([M + H]^+^), calcd for C_24_H_25_NO_7_ + H^+^ 440.1705, found 440.1703.

*8-Fluoro-4,4,6-trimethyl-6-phenyl-5,6-dihydro-4H-pyrrolo[3,2,1-ij]quinoline-1,2-dione* (**10f**). Orange solid, 1.11 g; yield 69%; R*_f_* = 0.55 (5:1 petroleum ether/ethyl acetate); m.p. 160–162 °C; IR (KBr, ν_max_/cm^−1^): 1724 (C=O), 704, 427; ^1^H NMR (600.13 MHz, DMSO-*d*_6_), δ (ppm): 0.72 (3H, s, C^6^-CH_3_); 1.59 (3H, s, C^4^-CH_3_); 1.69 (3H, s, C^4^-CH_3_); 2.09 (1H, d, *J* = 14.4 Hz, C^5^-H); 2.48 (1H, d, *J* = 14.4 Hz, C^5^-H, 7.15 (2H, d, *J* = 8.1 Hz, Ph); 7.20 (1H, t, *J* = 7.3 Hz, Ph); 7.29 (2H, t, *J* = 7.6 Hz, Ph); 7.42 (1H, dd, *J* = 6.8 Hz, *J* = 2.2 Hz, H-7(9)); 7.58 (1H, dd, *J* = 10.3 Hz, *J* = 2.2 Hz, H-7(9)); ^13^C-NMR (150.90 MHz, DMSO-*d*_6_), δ (ppm): 24.6 and 24.7 (stereoisomers), 27.6, 30.2, 39.5, 50.1, 53.6, 109.8, 109.9, 117.06, 117.11, 122.5, 122.7, 126.2, 126.4, 128.17, 129.21, 143.6, 146.9, 156.6 *(C-F)* and 157.7 *(C-F)*, 159.3, 183.2. HPLC-HRMS-ESI, *m/z* ([M + H]^+^), calcd for C_20_H_18_FNO_2_+ H^+^ 324.1395, found 324.1401.

*6-(4-Chlorophenyl)-8-fluoro-4,4,6-trimethyl-5,6-dihydro-4H-pyrrolo[3,2,1-ij]quinoline-1,2-dione* (**10g**). Orange solid, 1.28 g; yield 72%; R*_f_* = 0.48 (5:1 petroleum ether/ethyl acetate); m.p. 143–145 °C; IR (KBr, ν_max_/cm^−1^): 1727 (C=O), 1302, 818, 453; ^1^H NMR (600.13 MHz, DMSO-*d*_6_), δ (ppm): 0.75 (3H, s, C^6^-CH_3_); 1.60 (3H, s, C^4^-CH_3_); 1.69 (3H, s, C^4^-CH_3_); 2.10 (1H, d, *J* = 14.5 Hz, C^5^-H); 2.47 (1H, d, *J* = 14.5 Hz, C^5^-H); 7.18 (2H, d, *J* = 8.5 Hz, C_6_H_4_); 7.35 (2H, d, *J* = 8.5 Hz, C_6_H_4_); 7.42 (1H, dd, *J* = 6.8 Hz, *J* = 2.3 Hz, H-7(9)); 7.58 (1H, dd, *J* = 10.3 Hz, *J* = 2.4 Hz, H-7(9))^13^C-NMR (150.90 MHz, DMSO-*d*_6_), δ (ppm): 24.8, 27.5, 30.2, 39.3, 49.9, 53.5, 110.0, 110.1, 117.2, 117.3, 122.4, 122.5, 128.1, 128.5, 128.59, 128.63, 130.9, 143.6, 146.0, 156.6, 157.7, 159.3, 183.2. HPLC-HRMS-ESI, *m/z* ([M + H]^+^), calcd for C_20_H_17_ClFNO_2_+ H^+^ 358.1006, found 358.1008.

*6-(4-Chlorophenyl)-4,4,6,8-tetramethyl-5,6-dihydro-4H-pyrrolo[3,2,1-ij]quinoline-1,2-dione* (**10l**). Orange solid, 1.11 g; yield 63%; R*_f_* = 0.44 (5:1 petroleum ether/ethyl acetate); m.p. 169–171 °C; ^1^H NMR (600.13 MHz, DMSO-*d*_6_), δ (ppm): 0.76 (3H, s, C^6^-CH_3_); 1.59 (3H, s, C^4^-CH_3_); 1.68 (3H, s, C^4^-CH_3_); 2.08 (1H, d, *J* = 14.5 Hz, C^5^-H); 2.33 (3H, s, C^8^-CH_3_); 2.44 (1H, d, *J* = 14.5 Hz, C^5^-H); 7.17 (2H, d, *J* = 8.5 Hz, CH_arom_); 7.32–7.35 (3H, m, CH_arom_); 7.48 (1H, s, CH_arom_); ^13^C-NMR (150.90 MHz, DMSO-*d*_6_), δ (ppm): 20.3, 24.9, 27.6, 30.2, 38.9, 50.1, 53.5, 116.3, 123.3, 126.6, 128.0, 128.5, 130.8, 132.3, 136.6, 145.0, 146.5, 156.7, 184.1. HPLC-HRMS-ESI, *m/z* ([M + H]^+^), calcd for C_21_H_20_ClNO_2_+ H^+^ 354.1256, found 354.1255.

*8-Ethoxy-4,4,6-trimethyl-5,6-dihydro-4H-pyrrolo[3,2,1-ij]quinoline-1,2-dione* (**10m**). Orange solid, 1.16 g; yield 85%; R*_f_* = 0.32 (chloroform); m.p. 169–171 °C; ^1^H NMR (300.13 MHz, DMSO-*d*_6_), δ (ppm): 1.28–1.32 (9H, m, C^6^-CH_3_ +C^4^-CH_3_ +, OCH_2_CH_3_); 1.57 (1H, t, *J* = 15 Hz, C^5^-H); 1.67 (3H, s, C^4^-CH_3_); 1.84 (1H, dd, *J* = 15 Hz, *J* = 6 Hz, C^5^-H); 2.86 (1H, m, C^6^-H); 4.02 (2H, q, *J* = 6 Hz, OCH_2_CH_3_); 6.93 (1H, s, H-7); 7.13 (1H, s, H-9); ^13^C-NMR (150.90 MHz, DMSO-*d*_6_), δ (ppm): 15.1, 18.6, 24.5, 25.9, 27.1, 45.0, 54.0, 64.4, 107.4, 116.4, 122.6, 128.5, 142.1, 155.4, 157.2, 184.7. HPLC-HRMS-ESI, *m/z* ([M + H]^+^), calcd for C_16_H_19_NO_3_+ H^+^ 274.1439, found 274.1436.

*6-(4-Chlorophenyl)-4,4,6-trimethyl-5,6-dihydro-4H-pyrrolo[3,2,1-ij]quinoline-1,2-dione* (**10o**). Orange solid, 0.97 g; yield 57%; R*_f_* = 0.26 (chloroform); m.p. 113–115 °C; ^1^H NMR (600.13 MHz, DMSO-*d*_6_), δ (ppm): 0.77 (3H, s, C^6^-CH_3_); 1.61 (3H, s, C^4^-CH_3_); 1.69 (3H, s, C^4^-CH_3_); 2.10 (1H, d, *J* = 14.5 Hz, C^5^-H); 2.46 (1H, d, *J* = 14.5 Hz, C^5^-H); 7.15–7.20 (3H, m, CH_arom_); 7.33 (2H, d, *J* = 8.5 Hz, CH_arom_); 7.51 (1H, d, *J* = 7.3 Hz, CH_arom_); 7.64 (1H, d, *J* = 7.8 Hz, CH_arom_); ^13^C-NMR (150.90 MHz, DMSO-*d*_6_), δ (ppm): 25.0, 27.7, 30.4, 39.1, 50.0, 53.6, 116.5, 123.0, 123.1, 126.8, 128.1, 128.6, 130.9, 136.5, 146.6, 147.2, 156.8, 183.9. HPLC-HRMS-ESI, *m/z* ([M + H]^+^), calcd for C_20_H_18_ClNO_2_+ H^+^ 340.1099, found 340.1097.

General procedure for the synthesis of (2,4,5,6-tetrahydro-1*H*-pyrrolo[3,2,1-*ij*]quinolin-1-ylidene)-2-thioxothiazolidin-4-ones **12a**–**q**. A mixture of the corresponding pyrrolo[3,2,1-*ij*]quinoline-1,2-dione (**5**, **6**, **9a**–**c**, **10a**–**l**) (1 mmol) and 2-thioxothiazolidin-4-one (**11a**,**b**) (1 mmol) in glacial acetic acid (25 mL) and freshly fused sodium acetate (2 mmol) was heated under reflux for 1–6 h. The resulting precipitate was filtered off, washed with water, and recrystallized from 2-PrOH/acetic acid.

*(Z)-2-Thioxo-5-(4,4,6-trimethyl-2-oxo-5,6-dihydro-4H-pyrrolo[3,2,1-ij]quinolin-1(2H)-ylidene)thiazolidin-4-one* (**12a**). Dark-brown solid, 0.29 g; yield 85%; R*_f_* = 0.50 (10:1 chloroform/ethyl acetate); m.p. 261–263 °C; ^1^H NMR (600.13 MHz, DMSO-*d*_6_), δ (ppm): 1.32 (3H, d, *J* = 6.3 Hz, C^6^-CH3); 1.35 (3H, s, C^4^-CH3); 1.56 (2H, t, *J* = 12.8 Hz, C^5^-H); 1.72 (3H, s, C^4^-CH3); 1.86–1.90 (1H, m, C^6^-H); 7.01 (1H, t, *J* = 12.8 Hz, H-8); 7.33 (1H, d, *J* = 7.7 Hz, H-7(9)); 8.52 (1H, d, *J* = 7.7 Hz, H-7(9)); 13.88 (1H, br.s, NH); ^13^C NMR (150.90 MHz, DMSO-*d*_6_), δ (ppm): 17.9, 24.4, 25.4, 26.7, 44.9, 54.1, 117.63, 122.2, 124.7, 125.2, 125.3, 129.0, 133.4, 141.1, 165.36, 169.1, 199.7. HPLC-HRMS-ESI, *m*/*z* ([M + H]^+^), calcd for C_17_H_16_N_2_O_2_S_2_+ H^+^ 345.0727, found 345.0732.

*(Z)-5-(8-Iodo-4,4,6-trimethyl-2-oxo-5,6-dihydro-4H-pyrrolo[3,2,1-ij]quinolin-1(2H)-ylidene)-2-thioxothiazolidin-4-one* (**12b**). Dark-red solid, 0.28 g; yield 60%; R*_f_* = 0.65 (10:1 chloroform/ethyl acetate); m.p. 294–296 °C; ^1^H NMR (600.13 MHz, DMSO-*d_6_*), δ (ppm): 1.31 (3H, d, *J* = 6.6 Hz, C^6^-CH_3_); 1.33 (3H, s, C^4^-CH_3_); 1.56 (1H, m, C^5^-H); 1.71 (3H, s, C^4^-CH_3_); 1.86–1.88 (1H, m, C^5^-H); 2.92 (1H, s, C^6^-H); 7.64–7.68 (1H, m, H-7(9)); 8.87–8.91 (1H, m, H-7(9)); 14.12 (1H, br.s, NH); ^13^C NMR (150.90 MHz, DMSO-*d*_6_), δ (ppm): 17.8, 24.4, 25.5, 26.5, 44.5, 54.3, 85.7, 119.7, 122.9, 128.1, 133.1, 136.8, 140.6, 164.9, 199.6. HPLC-HRMS-ESI, *m*/*z* ([M + H]^+^), calcd for C_17_H_15_IN_2_O_2_S_2_+ H^+^ 470.9692, found 470.9698.

*(Z)-4,4,6-Trimethyl-2-oxo-1-(4-oxo-2-thioxothiazolidin-5-ylidene)-1,2,5,6-tetrahydro-4H-pyrrolo[3,2,1-ij]quinolin-8-yl benzoate* (**12c**). Brown–purple-needle crystals, 0.30 g; yield 65%; R*_f_* = 0.68 (10:1 chloroform/ethyl acetate); m.p. 276–278 °C; ^1^H NMR (600.13 MHz, DMSO-*d*_6_), δ (ppm): 1.32 (3H, d, *J* = 6.7 Hz, C^6^-CH_3_); 1.37 (3H, s, C^4^-CH_3_); 1.60 (1H, t, *J* = 12.9 Hz, C^5^-H); 1.74 (3H, s, C^4^-CH_3_); 1.93 (1H, dd, J = 13.7 Hz, *J* = 4.5 Hz, C^5^-H); 2.93–2.98 (1H, m, C^6^-H); 7.34 (1H, s, H-7(9)); 7.61 (2H, t, *J* = 7.7 Hz, CH_arom_); 7.76 (1H, t, *J* = 7.5 Hz, CH_arom_); 8.15 (2H, d, *J* = 7.7 Hz, CH_arom_); 8.42 (1H, s, H-7(9)); 14.01 (1H, br.s, NH); ^13^C NMR (150.90 MHz, DMSO-*d*_6_), δ (ppm): 17.8, 24.4, 25.7, 26.7, 44.7, 54.2, 117.9, 118.8, 122.7, 123.9, 126.3, 128.7, 128.9, 129.7, 134.0, 135.0, 138.9, 145.6, 164.9, 165.4, 169.3, 199.7. HPLC-HRMS-ESI, *m*/*z* ([M + H]^+^), calcd for C_24_H_20_N_2_O_4_S_2_+ H^+^ 465.0938, found 465.0944.

*(Z)-4,4,6-Trimethyl-2-oxo-1-(4-oxo-2-thioxothiazolidin-5-ylidene)-1,2,5,6-tetrahydro-4H-pyrrolo[3,2,1-ij]quinolin-8-yl 2-methoxybenzoate* (**12d**). Dark-blue-needle crystals, 0.37 g; yield 75%; R*_f_* = 0.45 (10:1 chloroform/ethyl acetate); m.p. 287–289 °C; ^1^H NMR (600.13 MHz, DMSO-*d*_6_), δ (ppm): 1.33 (3H, d, *J* = 5.9 Hz, C^6^-CH_3_); 1.36 (3H, s, C^4^-CH_3_); 1.60 (1H, t, *J* = 13.0 Hz, C^5^-H); 1.74 (3H, s, C^4^-CH_3_); 1.92 (1H, dd, *J* = 13.7 Hz, *J* = 4.5 Hz, C^5^-H); 2.94–2.98 (1H, m, C^6^-H); 3.90 (3H, s, O-CH_3_); 7.10 (1H, t, *J* = 7.5 Hz, CH_arom_); 7.24 (1H, d, *J* = 8.5 Hz, CH_arom_); 7.29 (1H, s, H-7(9)); 7.66 (1H, t, *J* = 7.6 Hz, CH_arom_); 7.93 (1H, d, *J* = 7.3 Hz, CH_arom_); 8.41 (1H, d, *J* = 1.2 Hz H-7(9)); 14.01 (1H, br.s, NH); ^13^C NMR (150.90 MHz, DMSO-*d*_6_), δ (ppm): 17.9, 24.4, 25.7, 26.6, 44.7, 54.2, 55.8, 112.6, 118.0, 118.5, 118.9, 120.1, 122.7, 124.0, 126.2, 131.4, 134.5, 135.0, 138.8, 145.7, 158.9, 164.3, 165.4, 169.4, 199.7. HPLC-HRMS-ESI, *m*/*z* ([M + H]^+^), calcd for C_25_H_22_N_2_O_5_S_2_+ H^+^ 495.1044, found 495.1045.

*(Z)-4,4,6-Trimethyl-2-oxo-1-(4-oxo-2-thioxothiazolidin-5-ylidene)-1,2,5,6-tetrahydro-4H-pyrrolo[3,2,1-ij]quinolin-8-yl 3,4,5-trimethoxybenzoate* (**12e**). Brown–purple-needle crystals, 0.37 g; yield 67%; R*_f_* = 0.58 (10:1 chloroform/ethyl acetate); m.p. 314–316 °C; IR (KBr, ν_max_/cm^−1^): 3221, 3106 (NH), 1687 (N-C=O), 1321, 1241 (C=S), 1129 (C-S), 736; ^1^H NMR (600.13 MHz, DMSO-*d*_6_)*,* δ (ppm): 1.32 (3H, d, *J* = 6.6 Hz, C^6^-CH_3_); 1.37 (3H, s, C^4^-CH_3_); 1.61 (1H, t, *J* = 12.9 Hz, C^5^-H); 1.74 (3H, s, C^4^-CH_3_); 1.93 (1H, dd, *J* = 13.7 Hz, *J* = 4.3 Hz, C^5^-H); 2.94–2.98 (1H, m, C^6^-H); 3.34 (3H, s, O-CH3); 3.78 (3H, s, O-CH_3_); 3.88 (3H, s, O-CH_3_); 7.31 (1H, s, H-7); 7.42 (2H, s, CH_arom_); 8.40 (1H, s, H-9); 14.01 (1H, br.s, NH); ^13^C NMR (150.90 MHz, DMSO-*d*_6_), δ (ppm): 17.9, 24.4, 25.6, 26.6, 44.7, 54.2, 56.0, 60.1, 107.0, 117.9, 118.0, 118.9, 122.6, 123.6, 123.9, 126.3, 138.9, 142.3, 145.7, 152.8, 164.6, 165.4, 199.8. HPLC-HRMS-ESI, *m*/*z* ([M + H]^+^), calcd for C_27_H_26_N_2_O_7_S_2_+ H^+^ 555.1256, found 555.1259

*(Z)-3-Ethyl-5-(8-methoxy-4,4,6-trimethyl-2-oxo-5,6-dihydro-4H-pyrrolo[3,2,1-ij]quinolin-1(2H)-ylidene)-2-thioxothiazolidin-4-one* (**12f**). Dark-blue-needle crystals, 0.35 g; yield 86%; R*_f_* = 0.59 (10:1 chloroform/ethyl acetate); m.p. 230–232 °C; ^1^H NMR (500.13 MHz, DMSO-*d*_6_), δ (ppm): 1.23 (3H, t, *J* = 7.1 Hz, CH_2_CH_3_); 1.33 (3H, d, *J* = 6.7 Hz, C^6^-CH_3_); 1.35 (3H, s, C^4^-CH_3_); 1.55–1.66 (1H, m, C^5^-H); 1.71 (3H, s, C^4^-CH_3_); 1.89 (1H, dd, *J* = 13.7 Hz, *J* = 4.7 Hz, C^5^-H); 2.89–2.94 (1H, m, C^6^-H); 3.80 (3H, s, O-CH_3_); 4.10 (1H, q, *J* = 7.1 Hz, CH_2_CH_3_); 6.96 (1H, s, H-7(9)); 8.28 (1H, s, H-7(9)); ^13^C NMR (125.76 MHz, DMSO-*d*_6_), δ (ppm): 11.8, 18.1, 24.3, 25.9, 26.7, 39.1, 45.4, 54.1, 55.9, 111.6, 116.1, 118.3, 126.2, 126.3, 135.7, 155.5, 165.3, 165.4, 197.4. HPLC-HRMS-ESI, *m*/*z* ([M + H]^+^), calcd for C_20_H_22_N_2_O_3_S_2_+ H^+^ 403.1146, found 403.1141.

*(Z)-5-(8-Fluoro-4,4,6-trimethyl-2-oxo-6-phenyl-5,6-dihydro-4H-pyrrolo[3,2,1-ij]quinolin-1(2H)-ylidene)-2-thioxothiazolidin-4-one* (**12g**). Dark-blue-needle crystals, 0.36 g; yield 83%; R*_f_* = 0.47 (10:1 chloroform/ethyl acetate); m.p. 308–310 °C; ^1^H NMR (600.13 MHz, DMSO-*d*_6_), δ (ppm): 0.70 (3H, s, C^6^-CH_3_); 1.62 (3H, s, C^4^-CH_3_); 1.70 (3H, s, C^4^-CH_3_); 2.13 (1H, d, *J* = 14.1 Hz, C^5^-H); 2.53 (1H, d, *J* = 14.3 Hz, C^5^-H); 7.08 (2H, d, *J* = 7.7 Hz, CH_arom_); 7.18 (1H, t, *J* = 7.2 Hz, CH_arom_); 7.26 (2H, t, *J* = 6.6 Hz, CH_arom_); 7.37 (1H, d, *J* = 9.8 Hz, H-7(9)); 8.51 (1H, d, *J* = 7.7 Hz, H-7(9)); 14.10 (1H, br.s, NH); ^13^C NMR 150.90 MHz, DMSO-*d*_6_)*,* δ (ppm): 24.8, 27.8, 30.2, 39.9, 50.5, 54.2, 112.9, 113.1, 117.3, 117.5, 118.8, 118.9, 123.5, 126.3, 126.4, 127.5, 127.6 *(C-F)*, 128.3, 136.0, 137.5, 147.3, 157.2, 158.8, 165.2, 169.4, 199.5. HPLC-HRMS-ESI, *m*/*z* ([M + H]^+^), calcd for C_23_H_19_FN_2_O_2_S_2_+ H^+^ 439.0946, found 439.0948.

(*Z)-5-(8-Bromo-4,4,6-trimethyl-2-oxo-6-phenyl-5,6-dihydro-4H-pyrrolo[3,2,1-ij]quinolin-1(2H)-ylidene)-2-thioxothiazolidin-4-one* (**12h**). Dark-brown solid, 0.39 g; yield 79%; R*_f_* = 0.47 (10:1 chloroform/ethyl acetate); m.p. 291–293 °C; ^1^H NMR (500.13 MHz, DMSO-*d*_6_), δ (ppm): 0.74 (3H, s, C^6^-CH_3_); 1.62 (3H, s, C^4^-CH_3_); 1.72 (3H, s, C^4^-CH_3_); 2.14 (1H, d, *J* = 14.4 Hz, C^5^-H); 2.50 (1H, d, *J* = 14.1 Hz, C^5^-H); 7.07 (2H, d, *J* = 7.7 Hz, CH_arom_); 7.18 (1H, t, *J* = 7.2 Hz, CH_arom_); 7.27 (2H, t, *J* = 7.5 Hz, CH_arom_); 7.60 (1H, s, H-7(9)); 8.90 (1H, s, H-7(9)); 14.10 (1H, br.s, NH); ^13^C NMR (125.76 MHz, DMSO-*d*_6_), δ (ppm): 24.9, 27.9, 39.8, 40.0, 50.6, 54.2, 114.5, 120.1, 122.9, 126.4, 126.5, 128.4, 128.6, 132.7, 136.5, 140.3, 147.4, 165.2, 169.6, 199.6. HPLC-HRMS-ESI, *m*/*z* ([M + H]^+^), calcd for C_23_H_19_BrN_2_O_2_S_2_+ H^+^ 499.0145, found 499.0151.

*(Z)-5-(8-Iodo-4,4,6-trimethyl-2-oxo-6-phenyl-5,6-dihydro-4H-pyrrolo[3,2,1-ij]quinolin-1(2H)-ylidene)-2-thioxothiazolidin-4-one* (**12i**). Brown solid, 0.44 g; yield 82%; R*_f_* = 0.51 (10:1 chloroform/ethyl acetate); m.p. 269–271 °C; ^1^H NMR (600.13 MHz, DMSO-*d*_6_), δ (ppm): 0.71 (3H, s, C^6^-CH_3_); 1.61 (3H, s, C^4^-CH_3_); 1.70 (3H, s, C^4^-CH_3_); 2.13 (1H, d, *J* = 14.3 Hz, C^5^-H); 2.48 (1H, d, *J* = 14.4 Hz, C^5^-H); 7.06 (2H, d, *J* = 7.7 Hz, CH_arom_); 7.18 (1H, t, *J* = 7.2 Hz, CH_arom_); 7.27 (2H, t, *J* = 7.6 Hz, CH_arom_); 7.75 (1H, s, H-7(9)); 9.07 (1H, s, H-7(9)); 14.10 (1H, br.s, NH); ^13^C NMR (150.90 MHz, DMSO-*d*_6_), δ (ppm): 24.8, 27.8, 30.0, 50.4, 54.2, 85.8, 120.3, 122.7, 126.2, 126.4, 128.2, 128.7, 133.9, 136.0, 138.3, 140.6, 147.3, 164.8, 169.4, 199.4. HPLC-HRMS-ESI, *m*/*z* ([M + H]^+^), calcd for C_23_H_19_IN_2_O_2_S_2_+ H^+^ 547.0005, found 547.0001.

*(Z)-3-Ethyl-2-thioxo-5-(4,4,6-trimethyl-2-oxo-6-phenyl-5,6-dihydro-4H-pyrrolo[3,2,1-ij]quinolin-1(2H)-ylidene)thiazolidin-4-one* (**12j**). Dark-red solid, 0.35 g; yield 77%; R*_f_* = 0.76 (10:1 chloroform/ethyl acetate); m.p. 254–256 °C; ^1^H NMR (500.13 MHz, DMSO-*d*_6_), δ (ppm): 0.75–0.77 (3H, m, C^6^-CH_3_); 1.23 (3H, tt, J = 7.0 Hz, J = 2.9 Hz, CH_2_CH_3_); 1.64 (3H, s, C^4^-CH_3_); 1.71 (3H, s, C^4^-CH_3_); 2.15 (1H, d, *J* = 14.3 Hz, C^5^-H); 2.52 (1H, d, *J* = 14.3 Hz, C^5^-H); 4.10–4.13 (2H, m, CH_2_CH_3_); 7.07 (2H, d, *J* = 7.7 Hz, CH_arom_); 7.14–7.26 (4H, m, H-8 +CH_arom_); 7.46 (1H, d, *J* = 7.7 Hz, H-7(9)); 8.77 (1H, d, *J* = 7.8 Hz, H-7(9)); ^13^C NMR (125.76 MHz, DMSO-*d*_6_), δ (ppm): 11.9, 24.8, 27.9, 30.3, 50.6, 54.1, 118.3, 122.5, 125.3, 126.1, 126.3, 126.4, 128.1, 131.1, 131.3, 141.3, 147.7, 165.3, 166.3, 197.1. HPLC-HRMS-ESI, *m*/*z* ([M + H]^+^), calcd for C_25_H_24_N_2_O_2_S_2_+ H^+^ 449.1353, found 449.1352.

*(Z)-5-(6-(4-Chlorophenyl)-8-fluoro-4,4,6-trimethyl-2-oxo-5,6-dihydro-4H-pyrrolo[3,2,1-ij]quinolin-1(2H)-ylidene)-2-thioxothiazolidin-4-one* (**12k**). Dark-blue-needle crystals, 0.31 g; yield 65%; R*_f_* = 0.48 (10:1 chloroform/ethyl acetate); m.p. 274–276 °C; ^1^H NMR (600.13 MHz, DMSO-*d*_6_), δ (ppm): 0.74 (3H, s, C^6^-CH_3_); 1.63 (3H, s, C^4^-CH_3_); 1.69 (3H, s, C^4^-CH_3_); 2.13 (1H, d, *J* = 14.2 Hz, C^5^-H); 2.50 (1H, d, *J* = 14.2 Hz, C^5^-H); 7.11 (2H, d, *J* = 7.6 Hz, CH_arom_); 7.32 (2H, d, *J* = 8.1 Hz, CH_arom_); 7.36 (1H, d, *J* = 9.9 Hz, H-7(9)); 8.51 (1H, d, *J* = 9.4 Hz, H-7(9)); 14.10 (1H, br.s, NH); ^13^C NMR (150.90 MHz, DMSO-*d*_6_), δ (ppm): 24.9, 27.7, 30.1, 39.6, 50.2, 54.1, 113.0, 113.2, 117.2, 117.3, 118.9, 119.0, 123.4, 126.9, 127.0, 128.1, 128.4 (C-F), 130.1, 136.1, 137.4, 146.3, 157.2, 158.8, 165.1, 169.4, 199.4. HPLC-HRMS-ESI, *m*/*z* ([M + H]^+^), calcd for C_23_H_18_ClFN_2_O_2_S_2_+ H^+^ 473.0556, found 473.0559.

*(Z)-5-(6-(4-Chlorophenyl)-8-iodo-4,4,6-trimethyl-2-oxo-5,6-dihydro-4H-pyrrolo[3,2,1-ij]quinolin-1(2H)-ylidene)-2-thioxothiazolidin-4-one* (**12l**). Dark-purple solid, 0.40 g; yield 69%; R*_f_* = 0.59 (10:1 chloroform/ethyl acetate); m.p. 285–287 °C; ^1^H NMR (600.13 MHz, DMSO-*d*_6_), δ (ppm): 0.76 (3H, s, C^6^-CH_3_); 1.64 (3H, s, C^4^-CH_3_); 1.69 (3H, s, C^4^-CH_3_); 2.15 (1H, d, *J* = 14.1 Hz, C^5^-H); 2.47 (1H, d, *J* = 14.3 Hz, C^5^-H); 7.09 (2H, d, *J* = 8.1 Hz, CH_arom_); 7.19 (1H, t, *J* = 7.8 Hz, CH_arom_); 7.31 (2H, d, *J* = 8.3 Hz, CH_arom_); 7.46 (1H, d, *J* = 7.7 Hz, H-7(9)); 8.74 (1H, d, *J* = 7.8 Hz, H-7(9)); 13.99 (1H, br.s, NH); ^13^C NMR (150.90 MHz, DMSO-*d*_6_), δ (ppm): 25.0, 27.8, 30.3, 33.1, 50.5, 54.1, 85.8, 118.5, 120.5, 122.6, 124.5, 125.7, 126.4, 128.1, 128.5, 130.9, 134.3, 138.3, 141.0, 146.9, 165.4, 169.3, 199.7. HPLC-HRMS-ESI, *m*/*z* ([M + H]^+^), calcd for C_23_H_18_ClIN_2_IO_2_S_2_+ H^+^ 580.9616, found 580.9615.

*(Z)-5-(6-(4-Chlorophenyl)-8-fluoro-4,4,6-trimethyl-2-oxo-5,6-dihydro-4H-pyrrolo[3,2,1-ij]quinolin-1(2H)-ylidene)-3-ethyl-2-thioxothiazolidin-4-one* (**12m**). Brown solid, 0.37 g; yield 73%; R*_f_* = 0.43 (10:1 chloroform/ethyl acetate); m.p. 235–237 °C; IR (KBr, ν_max_/cm^−1^): 3104, 2909 (NH), 1687(N-C=O), 1321, 1241 (C=S), 1129 (C-S), 736; ^1^H NMR (500.13 MHz, DMSO-*d*_6_), δ (ppm): 0.85*,* (3H, s, C^6^-CH_3_); 1.26 (3H, t, *J* = 6.4 Hz, CH_2_CH_3_), 1.64 (3H, s, C^4^-CH_3_); 1.71 (3H, s, C^4^-CH_3_); 2.15 (1H, d, *J* = 14.3 Hz, C^5^-H); 2.50 (1H, d, *J* = 12.3 Hz, C^5^-H; 4.15 (2H, q, *J* = 6.6 Hz, CH_2_CH_3_); 7.11 (2H, d, *J* = 8.1 Hz, CH_arom_); 7.32 (2H, d, *J* = 7.9 Hz, CH_arom_); 7.41 (1H, s, H-7); 8.53 (1H, s, H-9); ^13^C NMR (125.76 MHz, DMSO-*d*_6_), δ (ppm): 11.7, 25.1, 27.7, 30.1, 39.2, 39.8, 50.9, 54.3, 112.9, 113.2, 117.3, 117.4, 124.6, 127.4, 127.5, 128.1, 128.4, 131.1, 133.0, 137.8, 146.3, 157.2, 159.1, 165.3, 166.5, 196.9. HPLC-HRMS-ESI, *m*/*z* ([M + H]^+^), calcd for C_25_H_22_ClFN_2_O_2_S_2_+ H^+^ 501.0869, found 501.0871

*(Z)-5-(6′,6′-Dimethyl-2′-oxo-5′,6′-dihydrospiro[cyclohexane-1,4′-pyrrolo[3,2,1-ij]quinolin]-1′(2′H)-ylidene)-2-thioxothiazolidin-4-one* (**12n**). Brown solid, 0.27 g; yield 68%; R*_f_* = 0.58 (10:1 chloroform/ethyl acetate); m.p. 254–256 °C; IR (KBr, ν_max_/cm^−1^): 3385, 3225 (NH), 1686 (N-C=O), 1434, 1320, 1243 (C=S), 1131 (C-S), 747; ^1^H NMR (500.13 MHz, DMSO-*d*_6_), δ (ppm): 1.20–1.32 (2H, m, (CH_2_)_5_); 1.33 (6H, s, C^6^-(CH_3_)_2_); 1.55–1.70 (6H, m, (CH_2_)_5_ + C^5^-H); 2.05 (2H, s, (CH_2_)_5_); 2.53–2.60 (1H, m, C^5^-H + (CH_2_)_5_); 7.04 (1H, t, *J* = 7.3 Hz, H-8); 7.43 (1H, d, *J* = 7.7 Hz, H-7(9)); 8.58 (1H, d, *J* = 7.6 Hz, H-7(9)); 13.85 (1H, br.s, NH); ^13^C NMR (125.76 MHz, DMSO-*d*_6_), δ (ppm): 21.8, 25.0, 30.7, 31.5, 33.8, 42.3, 57.9, 118.2, 122.5, 125.1, 125.5, 129.0, 129.7, 133.8, 140.3, 166.0, 169.3, 200.1. HPLC-HRMS-ESI, *m*/*z* ([M + H]^+^), calcd for C_21_H_22_N_2_O_2_S_2_+ H^+^ 399.1197, found 399.1196.

*(Z)-5-(6′,8′-Dimethyl-2′-oxo-5′,6′-dihydrospiro[cyclohexane-1,4′-pyrrolo[3,2,1-ij]quinolin]-1′(2′H)-ylidene)-2-thioxothiazolidin-4-one* (**12o**). Dark-purple solid, 0.28 g; yield 71%; m.p. 294–296 °C; R*_f_* = 0.61 (10:1 chloroform/ethyl acetate); ^1^H NMR (500.13 MHz, DMSO-*d*_6_), δ (ppm): 1.20–1.31 (2H, m, (CH_2_)_5_); 1.33 (3H, d, *J* = 6.8 Hz, C^6^-CH_3_); 1.45–1.60 (4H, m, (CH_2_)_5_); 1.69–1.72 (1H, m, (CH_2_)_5_); 1.77–1.87 (3H, m, C^5^-H + (CH_2_)_5_); 2.30 (3H, s, C^8^-CH_3_); 2.52 (1H, dd, *J* = 14.0 Hz, J = 4.5 Hz, C^5^-H); 2.76–2.81 (1H, m, C^6^-H); 7.13 (1H, s, H-7(9)); 8.37 (1H, s, H-7(9)); 13.85 (1H, br.s, NH); ^13^C NMR (125.76 MHz, DMSO-*d*_6_), δ (ppm): 18.2, 20.9, 21.4, 21.9, 24.8, 24.9, 31.4, 33.5, 37.8, 58.0, 117.9, 125.4, 125.6, 125.8, 129.7, 131.0, 132.9, 139.7, 165.7, 168.9, 199.8. HPLC-HRMS-ESI, *m*/*z* ([M + H]^+^), calcd for C_21_H_22_N_2_O_2_S_2_+ H^+^ 399.1196, found 399.1198.

*(Z)-5-(8′-Fluoro-6′-methyl-2′-oxo-5′,6′-dihydrospiro[cyclopentane-1,4′-pyrrolo[3,2,1-ij]quinolin]-1′(2′H)-ylidene)-2-thioxothiazolidin-4-one* (**12p**). Dark-blue-needle crystals, 0.25 g; yield 65%; R*_f_* = 0.55 (10:1 chloroform/ethyl acetate); m.p. 288–290 °C; ^1^H NMR (500.13 MHz, DMSO-*d*_6_), δ (ppm): 1.31 (3H, d, *J* = 6.8 Hz, C^6^-CH_3_); 1.52–1.59 (2H, m, (CH_2_)_4_); 1.61–1.65 (1H, m, (CH_2_)_4_); 1.66–1.82 (3H, m, (CH_2_)_4_); 1.95–2.03 (2H, m, (CH_2_)_4_+ C^5^-H); 2.85–2.95 (2H, m, (CH_2_)_4_ + C^6^-H); 7.16 (1H, dd, *J* = 8.4 Hz, *J* = 1.7 Hz, H-7(9)); 8.24–8.27 (1H, m, H-7(9)); 13.85 (1H, br.s, NH); ^13^C NMR (125.76 MHz, DMSO-*d*_6_), δ (ppm): 17.7, 24.6, 25.0, 26.6, 35.3, 37.8, 42.9, 64.1, 111.6, 111.8, 115.5, 115.7, 117.8, 117.9, 123.8, 127.0, 127.1, 135.2, 137.9, 157.0, 158.9, 164.9, 169.2, 199.48. HPLC-HRMS-ESI, *m*/*z* ([M + H]^+^), calcd for C_19_H_17_FN_2_O_2_S_2_+ H^+^ 389.0789, found 389.0791.

*(Z)-5-(8′-Fluoro-6′-methyl-2′-oxo-5′,6′-dihydrospiro[cycloheptane-1,4′-pyrrolo[3,2,1-ij]quinolin]-1′(2′H)-ylidene)-2-thioxothiazolidin-4-one* (**12q**). Brown solid, 0.32 g; yield 77%; R*_f_* = 0.66 (10:1 chloroform/ethyl acetate); m.p. 343–345 °C; ^1^H NMR (500.13 MHz, DMSO-*d*_6_), δ (ppm): 1.30 (3H, d, *J* = 6.8 Hz, C^6^-CH_3_); 1.32–1.45 (2H, m, (CH_2_)_6_); 1.50–1.65 (6H, m, (CH_2_)_6_); 1.66–1.75 (2H, m, (CH_2_)_6_); 1.83–1.90 (2H, m, C^5^-H + (CH_2_)_6_); 2.24 (1H, dd, *J* = 9.5 Hz, *J* = 4.3 Hz, C^6^-H); 2.79–2.84 (1H, m, C^6^-H); 3.06–3.12 (1H, m, (CH_2_)_6_); 7.18 (1H, dd, *J* = 8.2 Hz, *J* = 1.8 Hz, H-7(9)); 8.27 (1H, dd, *J* = 8.4 Hz, *J* = 2.4 Hz, H-7(9)); 13.95 (1H, br.s, NH); ^13^C NMR (125.76 MHz, DMSO-*d*_6_), δ (ppm): 18.1, 22.5, 23.1, 25.3, 29.7, 29.9, 36.3, 37.5, 39.7, 42.4, 61.3, 111.7, 111.9, 115.7, 115.9, 118.2, 118.3, 123.9, 127.1, 127.2, 135.2, 137.7, 157.0, 158.9, 165.4, 169.2, 199.6. HPLC-HRMS-ESI, *m*/*z* ([M + H]^+^), calcd for C_21_H_21_FN_2_O_2_S_2_+ H^+^ 417.1102, found 417.1100.

General procedure for synthesis of substituted 5,6-dihydro-4*H*-pyrrolo[3,2,1-*ij*]quinolin-1(2*H*)-ylidene)-3-(prop-2-yn-1-yl)-2-thioxo- thiazolidin-4-one **14a**,**b**. To a suspension of compound 12 (0.75 mmol) in acetonitrile (20 mL) were added potassium carbonate (1.1 mmol) and propargyl bromide **13** (0.9 mmol), and the mixture was stirred at room temperature for 10 h. Completion of reaction was monitored by TLC. Next, the reaction mixture was diluted with water; brown solid was precipitated out. The precipitated product was filtered, washed with water, dried, and recrystallized from 2-PrOH.

(*Z)-5-(8-Fluoro-4,4,6-trimethyl-2-oxo-6-phenyl-5,6-dihydro-4H-pyrrolo[3,2,1-ij]quinolin-1(2H)-ylidene)-3-(prop-2-yn-1-yl)-2-thioxothiazolidin-4-one* (**14a**). Brown solid, 0.18 g; yield 52%; R*_f_* = 0.25 (10:1 chloroform/ethyl acetate); m.p. 190–192 °C; IR (KBr, ν_max_/cm^−1^): 2361, 2342 (C≡C), 1686 (N-C=O), 1433, 1321, 1241 (C=S), 1130 (C-S), 736;^1^H NMR (500.13 MHz, DMSO-*d*_6_), δ (ppm): 0.71 (3H, s, C^6^-CH_3_); 1.63 (3H, s, C^4^-CH_3_); 1.71 (3H, s, C^4^-CH_3_); 2.14 (1H, d, *J* = 14.4 Hz, C^5^-H); 2.54 (1H, d, *J* = 14.4 Hz, C^5^-H); 3.47–3.48 (1H, t, *J* = 2.2 Hz, ≡CH); 4.35 (2H, d, *J* = 1.4 Hz, CH_2_); 7.08–7.10 (2H, m, CH_arom_); 7.18 (1H, t, *J* = 7.3 Hz, CH_arom_); 7.24–7.29 (2H, m, CH_arom_); 7.41–7.45 (1H, m, H-7(9)); 8.62 – 8.65 (1H, m, H-7(9)); ^13^C NMR (125.76 MHz, DMSO-*d*_6_), δ (ppm): 21.3, 24.7, 27.7, 30.2, 39.9, 50.4, 54.2, 75.8, 78.0, 113.2, 113.4, 117.9, 118.1, 118.8, 126.2, 126.4, 126.6, 127.6, 128.2, 134.6, 137.4, 147.2, 157.2, 158.7, 165.3, 178.6, 196.3. HPLC-HRMS-ESI, *m*/*z* ([M + H]^+^), calcd for C_26_H_21_FN_2_O_2_S_2_+ H^+^ 477.1102, found 477.1108.

*(Z)-5-(6-(4-Chlorophenyl)-8-fluoro-4,4,6-trimethyl-2-oxo-5,6-dihydro-4H-pyrrolo[3,2,1-ij]quinolin-1(2H)-ylidene)-3-(prop-2-yn-1-yl)-2-thioxothiazolidin-4-one* (**14b**). Brown solid, 0.19 g; yield 49%; R*_f_* = 0.20 (10:1 chloroform/ethyl acetate); m.p. 125–127 °C; ^1^H NMR (500.13 MHz, DMSO-*d*_6_), δ (ppm): 0.85 (3H, s, C^6^-CH_3_); 1.64 (3H, s, C^4^-CH_3_); 1.71 (3H, s, C^4^-CH_3_); 2.15 (1H, d, *J* = 14.5 Hz, C^5^-H); 2.50 (1H, d, *J* = 14.5 Hz, C^5^-H); 3.25 (1H, s, ≡CH); 4.33 (1H, s, CH_2_); 7.11–7.14 (2H, m, CH_arom_); 7.28–7.31 (3H, m, CH_arom_); 8.63–8.65 (1H, m, H-7(9)); ^13^C NMR (125.76 MHz, DMSO-*d*_6_), δ (ppm): 21.4, 25.1, 27.7, 30.1, 39.8, 50.9, and 51.0 (stereoisomers), 54.3, 75.5, 77.8, 113.4 and 113.6, 117.6 and 117.8, 119.2 and 119.3, 127.37 and 127.43, 128.1, 128.4, 128.6, 131.1, 134.7, 137.5, 146.3, 157.2 and 159.1, 165.5, 178.5, 196.2. HPLC-HRMS-ESI, *m*/*z* ([M + H]^+^), calcd for C_26_H_20_ClFN_2_O_2_S_2_ + H^+^ 511.0713, found 511.0717.

General procedure for synthesis of substituted 4,4,6-trimethyl-1-(5-oxo-1-R_3_-2-thioxoimidazolidin-4-ylidene)-6-R_1_-8-R_2_-5,6-dihydro-4*H*-pyrrolo[3,2,1-*ij*]quinolin-2(1*H*)-one 16a-g. A mixture of pyrrolo[3,2,1-*ij*]quinoline-1,2-dione (**10**,**11**) (1 mmol) and 2-thioxoimidazolidin-4-one (1**6a**–**c**) (1 mmol), in glacial acetic acid (25 mL) and freshly fused sodium acetate (2 mmol), was heated under reflux for 4–10 h. The solid obtained after cooling was filtered, washed with water, dried and recrystallized from 2-PrOH.

(*Z)-8-Ethoxy-4,4,6-trimethyl-1-(5-oxo-2-thioxoimidazolidin-4-ylidene)-5,6-dihydro-4H-pyrrolo[3,2,1-ij]quinolin-2(1H)-one* (**16a**). Brown solid, 0.31 g; yield 83%; R*_f_* = 0.69 (10:1 chloroform/ethyl acetate); m.p. 343–345 °C; ^1^H NMR (500.13 MHz, DMSO-*d*_6_), δ (ppm): 1.31 (3H, d, *J* = 7.4 Hz, C^6^-CH_3_); 1.32 (3H, s, C^4^-CH_3_); 1.35 (3H, t, *J* = 7 Hz, OCH_2_CH_3_); 1.56 (1H, t, *J* = 13 Hz, C^5^-H); 1.72 (3H, s, C^4^-CH_3_); 1.97 (1H, dd, *J* = 13.8 Hz, *J* = 4.8 Hz, C^5^-H); 2.88–2.92 (1H, m, C^6^-H); 4.03 (2H, q, *J* = 7 Hz, OCH_2_CH_3_); 6.89 (1H, d, *J* = 1.9 Hz, H-7(9)); 8.51 (1H, d, *J* = 2.3 Hz, H-7(9)); 9.32 (1H, s, NH); 9.55 (1H, br.s, NH); ^13^C NMR (125.76 MHz, DMSO-*d*_6_), δ (ppm): 14.8, 18.2, 24.3, 25.8, 26.8, 45.4, 53.8, 63.6, 112.1, 114.6, 118.8, 125.4, 125.5, 133.6, 138.5, 154.2, 166.2, 178.8, 180.2. HPLC-HRMS-ESI, *m*/*z* ([M + H]^+^), calcd for C_19_H_21_N_3_O_3_S + H^+^ 372.1377, found 372.1376.

*(Z)-4,4,6,8-Tetramethyl-1-(5-oxo-2-thioxoimidazolidin-4-ylidene)-5,6-dihydro-4H-pyrrolo[3,2,1-ij]quinolin-2(1H)-one* (**16b**). Light-brown powder, 0.24 g; yield 71%; R*_f_* = 0.55 (10:1 chloroform/ethyl acetate); m.p. 357–359 °C; IR (KBr, ν_max_/cm^−1^): 3287, 3095 (NH), 1662 (N-C=O), 1543, 1353, 1220 (C=S), 769, 495; ^1^H NMR (500.13 MHz, DMSO-*d*_6_), δ (ppm): 1.31 (3H, d, *J* = 6.7 Hz, C^6^-CH_3_); 1.34 (3H, s, C^4^-CH_3_); 1.55 (1H, t, *J* = 13 Hz, C^5^-H); 1.72 (3H, s, C^4^-CH_3_); 1.86 (1H, dd, *J* = 13.6 Hz, J = 4.6 Hz, C^5^-H); 2.31 (3H, s, C^8^-CH_3_); 2.86–2.91 (1H, m, C^6^-H); 7.11 (1H, s, H-7(9)); 8.61 (1H, s, H-7(9)); 9.23 (1H, s, NH); 9.50 (1H, br.s, NH); ^13^C NMR (125.76 MHz, DMSO-*d*_6_), δ (ppm): 18.1, 21.2, 24.3, 25.4, 26.8, 45.3, 53.7, 118.3, 124.3, 125.2, 125.9, 128.1, 130.5, 137.4, 137.9, 166.4, 178.7, 180.0. HPLC-HRMS-ESI, *m*/*z* ([M + H]^+^), calcd for C_18_H_19_N_3_O_2_S + H^+^ 342.1272, found 342.1271.

*(Z)-4,4,6-Trimethyl-1-(5-oxo-2-thioxoimidazolidin-4-ylidene)-6-phenyl-5,6-dihydro-4H-pyrrolo[3,2,1-ij]quinolin-2(1H)-one* (**16c**). Orange powder, 0.28 g; yield 69%; R*_f_* = 0.66 (10:1 chloroform/ethyl acetate); m.p. 357–359 °C; ^1^H NMR (500.13 MHz, DMSO-*d*_6_), δ (ppm): 0.75 (3H, s, C^6^-CH_3_); 1.65 (3H, s, C^4^-CH_3_); 1.71 (3H, s, C^4^-CH_3_); 2.15 (1H, d, *J* = 14.3 Hz, C^5^-H); 2.51 (1H, d, *J* = 14.0 Hz, C^5^-H); 7.06 (1H, d, *J* = 7.8 Hz, H-7(9)); 7.14–7.19 (3H, m, CH_arom_); 7.24 (2H, t, *J* = 7.6 Hz, CH_arom_); 7.40 (1H, d, *J* = 7.8 Hz, CH_arom_); 8.96 (1H, d, *J* = 7.8 Hz, H-7(9)); 9.31 (1H, s, NH); 9.55 (1H, br.s, NH); ^13^C NMR (125.76 MHz, DMSO-*d*_6_), δ (ppm): 24.9, 28.1, 30.5, 39.4, 51.0, 54.0, 119.0, 122.1, 124.9, 125.7, 126.1, 126.2, 126.6, 128.2, 129.6, 138.9, 139.7, 148.1, 166.5, 178.7, 180.2. HPLC-HRMS-ESI, *m*/*z* ([M + H]^+^), calcd for C_23_H_21_N_3_O_2_S + H^+^ 404.1428, found 404.1427.

*(Z)-8-Fluoro-4,4,6-trimethyl-1-(5-oxo-2-thioxoimidazolidin-4-ylidene)-6-phenyl-5,6-dihydro-4H-pyrrolo[3,2,1-ij]quinolin-2(1H)-one* (**16d**). Orange powder, 0.27 g; yield 64%; R*_f_* = 0.38 (10:1 chloroform/ethyl acetate); m.p. 352–354 °C; IR (KBr, ν_max_/cm^−1^): 1726 (C=O), 1434, 1303, 1270 (C=S), 817, 454; ^1^H NMR (500.13 MHz, DMSO-*d*_6_), δ (ppm): 0.72 (3H, s, C^6^-CH_3_); 1.64 (3H, s, C^4^-CH_3_); 1.71 (3H, s, C^4^-CH_3_); 2.15 (1H, d, *J* = 14.3 Hz, C^5^-H); 2.52 (1H, d, *J* = 14.3 Hz, C^5^-H); 7.08 (2H, d, *J* = 7.6 Hz, CH_arom_); 7.18 (1H, t, *J* = 7.1 Hz, CH_arom_); 7.24–7.32 (3H, m, CH_arom_); 8.78 (1H, d, *J* = 9.5 Hz, CH_arom_); 9.42 (1H, s, NH); 9.68 (1H, s, NH); ^13^C NMR (125.76 MHz, DMSO-*d*_6_), δ (ppm): 24.8, 27.9, 30.3, 39.9, 50.8, 54.0, 113.0, 113.2, 115.7, 115.9, 119.5, 119.6, 124.0, 126.2, 126.5, 126.89, 126.94, 128.2, 136.1, 140.6, 147.5, 157.1, 158.9, 166.2, 178.5, 180.1. HPLC-HRMS-ESI, *m*/*z* ([M + H]^+^), calcd for C_23_H_20_FN_3_O_2_S + H^+^ 422.1334, found 422.1339.

*(Z)-6-(4-Chlorophenyl)-4,4,6,8-tetramethyl-1-(5-oxo-2-thioxoimidazolidin-4-ylidene)-5,6-dihydro-4H-pyrrolo[3,2,1-ij]quinolin-2(1H)-one* (**16e**). Light-brown powder, 0.31 g; yield 68%; R*_f_* = 0.41 (10:1 chloroform/ethyl acetate); m.p. 258–260 °C; ^1^H NMR (500.13 MHz, DMSO-*d*_6_), δ (ppm): 0.75 (3H, s, C^6^-CH_3_); 1.62 (3H, s, C^4^-CH_3_); 1.69 (3H, s, C^4^-CH_3_); 2.12 (1H, d, *J* = 14.3 Hz, C^5^-H); 2.37 (3H, s, C^8^-CH_3_); 2.48 (1H, d, *J* = 14.4 Hz, C^5^-H); 7.10 (2H, d, *J* = 8.6 Hz, CH_arom_); 7.27–7.32 (3H, m, CH_arom_); 8.60 (1H, s, CH_arom_); 12.8 (1H, s, NH); ^13^C NMR (125.76 MHz, DMSO-*d*_6_), δ (ppm): 21.3, 25.0, 25.5, 27.9, 30.3, 50.8, 54.0, 118.6, 125.3, 126.6, 127.0, 128.2, 128.6, 130.8, 130.9, 131.3, 132.4, 138.3, 147.1, 165.7, 167.3, 170.9. HPLC-HRMS-ESI, *m*/*z* ([M + H]^+^), calcd for C_24_H_22_ClN_3_O_2_S + H^+^ 452.1195, found 452.1201.

Spectra for all compounds are in supplementary file.

### 3.2. In Vitro Assays

The inhibition of blood-clotting factors Xa and XIa by the synthesized compounds **12a**–**q**, **14a**,**b**, and **16a**–**e** was studied by measuring the kinetics of hydrolysis of substrates specific to each of these enzymes in the presence of these compounds. A specific low-molecular-weight chromogenic substrate, S2765 (Z-D-Arg-Gly-Arg-pNA·2HCl), was used in the case of factor Xa, while the substrate S2366 (pyroGlu-Pro-ArgpNA·HCl) (both substrates from Chromogenix, USA) was used for factor XIa.

A buffer containing 140 m*M* NaCl, 20 mM HEPES, and 0.1% PEG 6000 (pH 8.0) was placed in the wells of a 96-well plate, followed by the addition of factor Xa (final concentration, 2.5 nmol·L^–1^) or XIa (final concentration 0.8 nmol·L^–1^), the substrate S2765 (final concentration, 200 μmol·L^–1^) or S2366 (final concentration, 200 μmol·L^–1^), and a solution of the test compound in DMSO (final concentration, 30 μmol·L^–1^; the DMSO content in the well was no more than 2%). The kinetics of the formation of *p*-nitroaniline were measured using a THERMOmax Microplate Reader (Molecular Devices Corporation, USA) using the absorption of light with a wavelength of 405 nm. The initial rate of substrate degradation was determined from the initial slope of the 4-nitroaniline-formation curve. The rate of substrate degradation by the enzyme in the presence of the inhibitor was expressed as a percentage relative to the rate of substrate degradation in the absence of the inhibitor. The results are presented in Table 1. The data obtained were processed using GraphPad Prism and OriginPro 8 software.

### 3.3. Molecular-Docking Studies

All simulations were performed by the SOL docking program [56,57]. This program docks flexible low-molecular-weight ligands into the active site of a rigid target protein based on the docking paradigm, which assumes that the ligand bonds with the protein near the global energy minimum of the protein–ligand complex. The search for the global energy minimum was performed using a genetic algorithm with following main parameters: the population size and the number of generations are 30,000 and 1000, respectively. First, using the SOLGRID module, a grid of potentials describing the interaction of the probe-ligand atoms with the protein was calculated. These interactions were obtained using the MMFF94 force field. The SOL scoring function estimated the free energy of protein–ligand binding. Docking solutions that were the ligand-docked positions obtained in several dozen (50 by default) independent runs of the genetic algorithm, and that corresponded to the lowest energy of the protein–ligand complex, were subjected to cluster analysis to assess the reliability of the global energy minimum. A high population of the first cluster-containing-ligand poses with lowest energy and a low number of clusters were the main clues that identified the reliability of docking results. Otherwise, re-docking was carried out with increases in parameters of the genetic algorithm.

The SOL docking program was validated and successfully used for the development of inhibitors of a number of target proteins: thrombin, urokinase (uPA), coagulation factor Xa (see references in [57]), and coagulation factors XIa [12] and XIIa [20,58]. For all these target proteins, when docking native ligands using the SOL program, a high accuracy of ligand positioning was achieved and the score cut-off separating inhibitors from inactive compounds was estimated. The high positioning accuracy meant that the root-mean-square deviation, RMSD, between all atoms in the crystallized-native-ligand pose and the docked-ligand pose, corresponding to the lowest energy of the protein–ligand complex, was less than 2.0 Å. The SOL score cut-offs were determined as explained below.

To predict the anticoagulant activity by docking, we retrieved high-quality structures of target proteins from PDB: structure of FXa from the 3CEN complex and FXIa from 4CRC. Both crystal complexes possess good resolution (<2.0 Å) and no missing residues. These structures were used to construct atomistic models of FXa and FXIa. Protein structures were manually cleaned from native ligands, water molecules, and salt ions. Their protonation was made in the Aplite program [59]. Extracted native ligands were protonated using Avogadro [60]. Validation of prepared protein structures was performed by the docking of native ligands and docking of known FXa and FXIa inhibitors. Both docking procedures for native ligands (the native ligand into the FXa model prepared from 3CEN and the native ligand into the FXIa model prepared from 4CRC) were successful, with RMSD values between the best docking pose and the native conformation of less than 1.4 Å. To study the known binders, we retrieved 10 crystal complexes of FXa and 10 complexes of FXIa with highly active inhibitors (see Table S2). The mean score for the known FXa inhibitors was −6.76 ± 0.44 kcal/mol and the mean score for the known FXIa inhibitors was −5.53 ± 0.56 kcal/mol. These values further served as score cut-offs for the selection of candidates.

#### ADME Properties

For the prediction of the ADMET properties of all compounds, the free web pkCSM server was used: https://biosig.lab.uq.edu.au/pkcsm/prediction (accessed 12 April 2023). For the prediction, all the molecules were first converted into SMILES (simplified molecule input line entry specification).

## 4. Conclusions

We applied docking in the search for new coagulation-factor-Xa and -XIa inhibitors. Eight of the twenty-four synthesized and experimentally tested quinolin-1,2-dione and rhodanine compounds exhibited selective or dual inhibitory activity against the coagulation factors Xa and Xia, with the IC_50_ ranging from 2.28–12.22 µM.

By varying the substituents in the central core and the rhodanine ring, we explored the primary SAR for the identified chemical series of dihydropyrrolo[3,2,1-*ij*]quinolinones condensed with rhodanine. Some substitutions were found to compromise activities for both factor Xa and factor XIa. For example, the N-alkylation of rhodanine is detrimental to anticoagulant activity, possibly due to its position close to active site walls in docking-predicted binding modes, or to the removal of negative charge. Several substitutions are associated with an increase in inhibition activity against only one of the two coagulation factors. For example, the introduction of acyloxy or halogen atoms at the 8th position of dihydropyrrolo[3,2,1-*ij*]quinolinone scaffold helps to increase activity against factor XIa.

According to the binding modes predicted by the docking, the three best factor-Xa inhibitors, **12k**, **12g**, and **12n** block access to the catalytic triad of factor Xa. The inhibitors **12g** and **12n**, on one hand, and of **12k**, on the other, bind to factor Xa in a different manner. While the **12k** inhibitor occupies the S1 pocket and the S4 hydrophobic pocket remains unoccupied, in the case of the **12g** and **12n** inhibitors, the S4 pocket is occupied, while the S1 pocket is almost unoccupied. These factor-Xa inhibitors have two different conformations with respect to the position of the rhodanine fragment: either they are located inside the S4 pocket, sandwiched between the aromatic rings of Tyr-99 and Phe-174, while the S1 pocket is practically unoccupied; or the rhodanine is directed into the solvent and located next to the guanidine fragment of Arg-222.

For both coagulation factors, the hydrophilic and charged ring of rhodanine is not buried in deep pockets of the target, but can rather be found in open pockets and on solvent-exposed surfaces, reducing the likelihood of a desolvation penalty.

As can be seen from the results of this work, for a more accurate selection of compounds for experiments to determine their inhibitory activities, docking plays a key role. However, docking alone is not sufficient, and it must be supplemented with post-processing. In post-processing, for the ligands with the best docking characteristics, quantum-chemical calculations of the protein–ligand-binding enthalpy can be performed, or the relative stability of the ligands in their docked positions can be estimated using sufficiently long (about 100 ns) molecular dynamic trajectories.

In addition to exploring SAR, we highlighted some optimization points for the identified chemical series, which were inferred from the comparison of the predicted binding modes with bound conformations of known inhibitors, and which can be exploited to increase potency against factor Xa. In all the cases studied, the P1 part of the identified inhibitors binding to the S1 pocket can be extended to fit the pocket better, and to be buried more deeply. The other aromatic rings in this part also need to be explored. The di-methyl part of **12k**, **12g**, and **12n** does not seem to contribute to activity specifically and can be substituted with other groups bearing H-bond acceptors/donors, considering its position near Gly-219 after docking to factor Xa. These and other modifications are can be exploited in the further development of the design.

## Figures and Tables

**Table 1:** Results of docking and the experimental measurements of anticoagulant activity of compounds **12a**–**q**, **14a**,**b**, and **16a**–**e** against factors Xa and XIa. The SOL score is the estimation of the protein–ligand-binding free energy calculated by the SOL docking program.

N°	Factor Xa, SOL Score, kcal/mol	Factor XIa, SOL Score, kcal/mol	Percent Inhibition at 30 μM		IC_50_, μM	
			Xa	XIa	Xa	XIa
**12a**	−5.28	−4.54	94	22	9.41 ± 0.35	-
**12b**	−5.69	−5.09	81	12	3.53 ± 0.08	-
**12c**	−6.04	−5.73	58	72	-	-
**12d**	−6.15	−5.84	63	98	2.35 ± 0.22	3.79 ± 0.03
**12e**	−5.59	−6.13	49	94	-	3.74 ± 0.33
**12f**	−5.15	−4.61	18	5	-	-
**12g**	−5.78	−5.41	99	98	3.70 ± 0.03	5.03 ± 0.18
**12h**	−5.96	−5.63	73	98	2.28 ± 0.23	3.73 ± 0.03
**12i**	−6.22	−5.43	75	96	3.44 ± 0.06	5.04 ± 0.33
**12j**	−5.42	−5.27	89	77	-	-
**12k**	−6.08	−5.65	99	99	2.85 ± 0.23	5.24 ± 0.29
**12l**	−6.43	−5.41	78	95	2.87 ± 0.21	4.30 ± 0.06
**12m**	−5.58	−5.69	18	0	-	-
**12n**	−6.15	−5.14	94	91	2.60 ± 0.50	9.41 ± 0.51
**12o**	−6.40	−5.12	48	15	-	-
**12p**	−5.96	−4.87	67	77	3.03 ± 0.18	12.22 ± 0.50
**12q**	−6.52	−4.96	47	91	-	4.32 ± 0.06
**14a**	−4.84	−5.26	36	3	-	-
**14b**	−5.23	−5.30	49	17	-	-
**16a**	−5.14	−4.34	2	6	-	-
**16b**	−5.45	−4.66	31	5		
**16c**	−5.82	−5.30	45	57		
**16d**	−5.80	−5.19	17	2		
**16e**	−6.19	−4.88	59	86		
**Rivaroxaban**	−6.89	-	94	8		

**Table 2:** ADMET values of Rivaroxaban and tested compounds.

No	Model Name—Predicted Value																					
	Water Solubility	Caco2 Permeability	Intestinal Absorption (Human)	Skin Permeability	P-Glycoprotein Substrate	P-Glycoprotein I Inhibitor	P-Glycoprotein II Inhibitor	VDss (Human)	Fraction Unbound (Human)	BBB Permeability	CNS Permeability	CYP2D6 Substrate	CYP3A4 Substrate	CYP2D6 Inhibitor	CYP3A4 Inhibitor	Total Clearance	Renal OCT2 Substrate	AMES Toxicity	Max. Tolerated Dose (Human)	Oral Rat Acute Toxicity (LD50)	Hepatotoxicity	Skin Sensitization
**12a**	−5.119	1.112	92.065	−3.099	No	Yes	No	0.477	0.164	0.173	−1.834	No	Yes	No	No	−0.094	No	No	−1.104	2.913	No	No
**12b**	−5.833	1.099	91.664	−3.123	No	Yes	No	0.422	0.121	0.085	−1.728	No	Yes	No	Yes	−0.537	No	No	−1.041	2.913	No	No
**12c**	−5.570	1.002	91.414	−2.732	Yes	Yes	Yes	0.403	0	−0.250	−1.672	No	Yes	No	Yes	−0.277	No	No	−0.470	3.116	Yes	No
**12d**	−5.196	1.195	93.192	−2.788	Yes	Yes	Yes	0.175	0.004	−0.670	−1.844	No	Yes	No	Yes	−0.141	No	Yes	−0.340	3.384	Yes	No
**12e**	−5.339	1.325	93.529	−2.770	Yes	Yes	Yes	−0.095	0	−1.117	−2.847	No	Yes	No	Yes	0.271	No	No	−0.144	3.137	No	No
**12f**	−5.250	1.187	93.334	−3.599	Yes	Yes	No	0.259	0.140	−0.178	−1.996	No	Yes	No	No	−0.059	No	No	−1.082	3.007	Yes	No
**12g**	−5.565	1.008	94.135	−2.745	Yes	Yes	Yes	0.461	0.016	−0.199	−1.667	No	Yes	No	Yes	−0.246	No	Yes	−0.669	3.126	Yes	No
**12h**	−5.785	0.959	92.948	−2.769	Yes	Yes	Yes	0.744	0.007	−0.074	−1.462	No	Yes	No	Yes	−0.251	No	Yes	−0.605	2.971	Yes	No
**12i**	−5.759	0.957	93.583	−2.771	Yes	Yes	Yes	0.746	0.009	−0.079	−1.495	No	Yes	No	Yes	−0.540	No	Yes	−0.619	0.953	Yes	No
**12j**	−5.553	0.977	94.048	−2.754	Yes	Yes	Yes	0.834	0.020	0.121	−1.463	No	Yes	No	Yes	0.092	No	No	−0.541	2.910	No	No
**12k**	−5.985	0.995	92.660	−2.754	Yes	Yes	Yes	0.455	0.008	−0.225	−1.548	No	Yes	No	Yes	−0.378	No	No	−0.638	3.233	No	No
**12l**	−6.128	0.944	92.108	−2.774	Yes	Yes	Yes	0.730	0	−0.105	−1.376	No	Yes	No	Yes	−0.672	No	No	−0.594	2.998	No	No
**12m**	−6.188	1.001	92.450	−2.742	Yes	Yes	Yes	0.515	0	−0.117	−1.489	No	Yes	No	Yes	−0.187	No	No	−0.572	3.271	No	No
**12n**	−6.079	1.108	91.003	−3.101	Yes	Yes	Yes	0.570	0.063	0.223	−1.514	No	Yes	No	Yes	−0.003	No	No	−1.274	2.912	No	No
**12o**	−6.194	1.115	90.689	−3.054	Yes	Yes	Yes	0.606	0.057	0.320	−2.537	No	Yes	No	Yes	0.196	No	No	−1.247	2.918	No	No
**12p**	−5.785	1.294	91.302	−3.416	Yes	Yes	Yes	0.290	0.088	−0.009	−1.690	No	Yes	No	Yes	−0.152	No	No	−1.176	2.985	No	No
**12q**	−6.27	1.290	90.525	−3.365	Yes	Yes	Yes	0.344	0.043	0.017	−1.507	No	Yes	No	Yes	−0.127	No	No	−1.243	3.008	No	No
**14a**	−6.077	1.27	97.261	−2.789	Yes	Yes	Yes	0.553	0	−0.132	−1.537	No	Yes	No	Yes	0.037	No	No	−0.321	2.954	No	No
**14b**	−6.530	1.261	96.471	−2.790	Yes	Yes	Yes	0.602	0	−0.133	−1.537	No	Yes	No	Yes	−0.094	No	No	−0.279	3.070	No	No
**16a**	−4.326	0.956	94.396	−3.340	Yes	No	No	0.000	0.233	−0.581	−1.985	No	Yes	No	No	−0.317	No	No	−0.248	2.712	No	No
**16b**	−4.539	1.269	94.806	−3.441	Yes	No	No	0.110	0.252	−0.119	−2.116	No	No	No	No	−0.422	No	No	−0.262	2.866	No	No
**16c**	−4.521	0.969	96.467	−2.811	Yes	Yes	Yes	0.545	0.024	−0.106	−2.016	No	Yes	No	Yes	−0.313	No	No	−0.118	2.511	Yes	No
**16d**	−4.721	1.418	96.112	−2.876	Yes	Yes	Yes	0.310	0.017	−0.240	−2.049	No	Yes	No	Yes	−0.457	No	No	−0.215	2.855	No	No
**16e**	−5.087	0.951	95.655	−2.814	Yes	Yes	Yes	0.634	0.018	−0.111	−1.817	No	Yes	No	Yes	−0.557	No	No	−0.139	2.766	No	No
**Rivaroxaban**	−4.382	1.2876	92.803	−2.967	No	Yes	No	−0.687	0	−1.022	−2.660	No	Yes	No	Yes	0.296	No	Yes	−0.232	2.707	Yes	No
**Unit**	Numeric (log mol/L)	Numeric (log Papp in 10^−6^ cm/s)	Numeric (% Absorbed)	Numeric (log Kp)	Categorical (Yes/No)	Categorical (Yes/No)	Categorical (Yes/No)	Numeric (log L/kg)	Numeric (Fu)	Numeric (log BB)	Numeric (log PS)	Categorical (Yes/No)	Categorical (Yes/No)	Categorical (Yes/No)	Categorical (Yes/No)	Numeric (log mL/min/kg)	Categorical (Yes/No)	Categorical (Yes/No)	Numeric (log mg/kg/day)	Numeric (mol/kg)	Categorical (Yes/No)	Categorical (Yes/No)

## Data Availability

See the file Electronic Supplementary Material.pdf containing ^1^H and ^13^C NMR, IR and data from HPLC-MS-ESI analysis for new synthesized compounds (Figures S1–S127).

## Supplementary Materials

The following supporting information can be downloaded at: https://www.mdpi.com/article/10.3390/molecules28093851/s1. The copies of ^1^H- and ^13^C-NMR spectra, IR, and data from HPLC-MS-ESI analysis for all new synthesized compounds were submitted along with the manuscript [61,62,63,64,65,66,67].
