# Supplementary material for: New Hybrid Tetrahydropyrrolo[3,2,1-ij]quinolin-1-ylidene-2-thioxothiazolidin-4-ones as New Inhibitors of Factor Xa and Factor XIa: Design, Synthesis, and In Silico and Experimental Evaluation
Source: Molecules. 2023 May 1;28(9):3851. doi: 10.3390/molecules28093851 (PMC10179972; doi:10.3390/molecules28093851)
Supplement: Supplementary file 1 [file molecules-28-03851-s001.zip › supplement material_final 10.04_VBS.pdf]

Electronic Supplementary Material file

New hybrid tetrahydropyrrolo[3,2,1-*ij*]quinolin-1-ylidene-2-thioxothiazolidin-4-ones as New Inhibitors of Factor Xa and Factor XIa: Design, Synthesis, Docking, in silico and experimental evaluation

Nadezhda P. Novichikhina [1], Alexander S. Shestakov [1], Svetlana M. Medvedeva [1], Anna M. Lagutina [1], Mikhail Yu. Krysin [1], Nadezhda A. Podoplelova [2,3], Mikhail A. Panteleev [2,3], Ivan S. Ilin[4,5], Alexey V. Sulimov[4,5], Anna S. Tashchilova[4,5], Vladimir B. Sulimov [4,5], Athina Geronikaki [6,*] and Khidmet S. Shikhaliev [1,*]

1 Department of Organic Chemistry, Faculty of Chemistry, Voronezh State University, Universitetskaya pl. 1, 394018 Voronezh, Russia

2 Dmitry Rogachev National Medical Research Center of Pediatric Hematology, Oncology and Immunology, 117997 Moscow, Russia

3 Center for Theoretical Problems of Physicochemical Pharmakology, 119991 Moscow, Russia

4 Dimonta, Ltd., 117186 Moscow, Russia

5 Research Computing Center, Lomonosov Moscow State University, 119992 Moscow, Russia

6 School of Pharmacy, Aristotle University of Thessaloniki, 54124 Thessaloniki, Greece

* Correspondence: geronik@pharm.auth.gr; shikh1961@yandex.ru.

Contents.

Copies of spectral data of synthesized compounds

^{1}H, ^{13}C NMR, IR and data HPLC-HRMS-ESI spectra of 8-bromo-4,4,6-trimethyl-6-phenyl-5,6-dihydro-4*H*-pyrrolo[3,2,1-*ij*]quinoline-1,2-dione 6 4

^{1}H, ^{13}C NMR and data HPLC-HRMS-ESI spectra of 6-iodo-2,2,4-trimethyl-1,2,3,4-tetrahydroquinoline hydrochloride 8a 6

^{1}H, ^{13}C NMR, IR and data HPLC-HRMS-ESI spectra of 6-iodo-2,2,4-trimethyl-4-phenyl-1,2,3,4-tetrahydroquinoline hydrochloride 8b 7

^{1}H, ^{13}C NMR, IR and data HPLC-HRMS-ESI spectra of 4-(4-chlorophenyl)-6-iodo-2,2,4-trimethyl-1,2,3,4-tetrahydroquinoline hydrochloride 8c 9

^{1}H, ^{13}C NMR, IR and data HPLC-HRMS-ESI spectra of 8-iodo-4,4,6-trimethyl-5,6-dihydro-4*H*-pyrrolo[3,2,1-*ij*]quinoline-1,2-dione 9a 11

^{1}H, ^{13}C NMR, IR and data HPLC-HRMS-ESI spectra of 8-iodo-4,4,6-trimethyl-6-phenyl-5,6-dihydro-4*H*-pyrrolo[3,2,1-*ij*]quinoline-1,2-dione 9b 13

^{1}H, ^{13}C NMR and data HPLC-HRMS-ESI spectra of 6-(4-chlorophenyl)-8-iodo-4,4,6-trimethyl-5,6-dihydro-4*H*-pyrrolo[3,2,1-*ij*]quinoline-1,2-dione 9c 15

^{1}H, ^{13}C NMR and data HPLC-HRMS-ESI spectra of 4,4,6-trimethyl-1,2-dioxo-1,2,5,6-tetrahydro-4*H*-pyrrolo[3,2,1-*ij*]quinolin-8-yl-2-methoxybenzoate 10c 17

^{1}H, ^{13}C NMR and data HPLC-HRMS-ESI spectra of 4,4,6-trimethyl-1,2-dioxo-1,2,5,6-tetrahydro-4*H*-pyrrolo[3,2,1-*ij*]quinolin-8-yl 3,4,5-trimethoxybenzoate 10d 18
^{1}H, ^{13}C NMR, IR and data HPLC-HRMS-ESI spectra of 8-fluoro-4,4,6-trimethyl-6-phenyl-5,6-dihydro-4*H*-pyrrolo[3,2,1-*ij*]quinoline-1,2-dione 10f 20
^{1}H, ^{13}C NMR, IR and data HPLC-HRMS-ESI spectra of 6-(4-chlorophenyl)-8-fluoro-4,4,6-trimethyl-5,6-dihydro-4*H*-pyrrolo[3,2,1-*ij*]quinoline-1,2-dione 10g.................................. 22
^{1}H, ^{13}C NMR and data HPLC-HRMS-ESI spectra of 6-(4-chlorophenyl)-4,4,6,8-tetramethyl-5,6-dihydro-4*H*-pyrrolo[3,2,1-*ij*]quinoline-1,2-dione 10l.. 24
^{1}H, ^{13}C NMR and data HPLC-HRMS-ESI spectra of 8-ethoxy-4,4,6-trimethyl-5,6-dihydro-4*H*-pyrrolo[3,2,1-*ij*]quinoline-1,2-dione 10m 25
^{1}H, ^{13}C NMR and data HPLC-HRMS-ESI spectra of 6-(4-chlorophenyl)-4,4,6-trimethyl-5,6-dihydro-4*H*-pyrrolo[3,2,1-*ij*]quinoline-1,2-dione 10o 27
^{1}H, ^{13}C NMR and data HPLC-HRMS-ESI spectra of (*Z*)-2-thioxo-5-(4,4,6-trimethyl-2-oxo-5,6-dihydro-4*H*-pyrrolo[3,2,1-*ij*]quinolin-1(2*H*)-ylidene)thiazolidin-4-one 12a 28
^{1}H, ^{13}C NMR and data HPLC-HRMS-ESI spectra of (*Z*)-5-(8-iodo-4,4,6-trimethyl-2-oxo-5,6-dihydro-4*H*-pyrrolo[3,2,1-*ij*]quinolin-1(2*H*)-ylidene)-2-thioxothiazolidin-4-one 12b 30
^{1}H, ^{13}C NMR and data HPLC-HRMS-ESI spectra of (*Z*)-4,4,6-trimethyl-2-oxo-1-(4-oxo-2-thioxothiazolidin-5-ylidene)-1,2,5,6-tetrahydro-4*H*-pyrrolo[3,2,1-*ij*]quinolin-8-yl benzoate 12c.. 31
^{1}H, ^{13}C NMR and data HPLC-HRMS-ESI spectra of (*Z*)-4,4,6-trimethyl-2-oxo-1-(4-oxo-2-thioxothiazolidin-5-ylidene)-1,2,5,6-tetrahydro-4*H*-pyrrolo[3,2,1-*ij*]quinolin-8-yl 2-methoxybenzoate 12d 33
^{1}H, ^{13}C NMR, IR and data HPLC-HRMS-ESI spectra of (*Z*)-4,4,6-trimethyl-2-oxo-1-(4-oxo-2-thioxothiazolidin-5-ylidene)-1,2,5,6-tetrahydro-4*H*-pyrrolo[3,2,1-*ij*]quinolin-8-yl 3,4,5-trimethoxybenzoate 12e 34
^{1}H, ^{13}C NMR and data HPLC-HRMS-ESI spectra of (*Z*)-3-ethyl-5-(8-methoxy-4,4,6-trimethyl-2-oxo-5,6-dihydro-4*H*-pyrrolo[3,2,1-*ij*]quinolin-1(2*H*)-ylidene)-2-thioxothiazolidin-4-one 12f 36
^{1}H, ^{13}C NMR and data HPLC-HRMS-ESI spectra of (*Z*)-5-(8-fluoro-4,4,6-trimethyl-2-oxo-6-phenyl-5,6-dihydro-4*H*-pyrrolo[3,2,1-*ij*]quinolin-1(2*H*)-ylidene)-2-thioxothiazolidin-4-one 12g.. 38
^{1}H, ^{13}C NMR and data HPLC-HRMS-ESI spectra of (*Z*)-5-(8-bromo-4,4,6-trimethyl-2-oxo-6-phenyl-5,6-dihydro-4*H*-pyrrolo[3,2,1-*ij*]quinolin-1(2*H*)-ylidene)-2-thioxothiazolidin-4-one 12h.. 39
^{1}H, ^{13}C NMR and data HPLC-HRMS-ESI spectra of (*Z*)-5-(8-iodo-4,4,6-trimethyl-2-oxo-6-phenyl-5,6-dihydro-4*H*-pyrrolo[3,2,1-*ij*]quinolin-1(2*H*)-ylidene)-2-thioxothiazolidin-4-one 12i... 41
^{1}H, ^{13}C NMR and data HPLC-HRMS-ESI spectra of (*Z*)-3-ethyl-2-thioxo-5-(4,4,6-trimethyl-2-oxo-6-phenyl-5,6-dihydro-4*H*-pyrrolo[3,2,1-*ij*]quinolin-1(2*H*)-ylidene)thiazolidin-4-one 12j 42
^{1}H, ^{13}C NMR and data HPLC-HRMS-ESI spectra of (*Z*)-5-(6-(4-chlorophenyl)-8-fluoro-4,4,6-trimethyl-2-oxo-5,6-dihydro-4*H*-pyrrolo[3,2,1-*ij*]quinolin-1(2*H*)-ylidene)-2-thioxothiazolidin-4-one 12k.. 44

^{1}H, ^{13}C NMR and data HPLC-HRMS-ESI spectra of (*Z*)-5-(6-(4-chlorophenyl)-8-iodo-4,4,6-trimethyl-2-oxo-5,6-dihydro-4*H*-pyrrolo[3,2,1-*ij*]quinolin-1(2*H*)-ylidene)-2-thioxothiazolidin-4-one 12l 45
^{1}H, ^{13}C NMR, IR and data HPLC-HRMS-ESI spectra of (*Z*)-5-(6-(4-chlorophenyl)-8-fluoro-4,4,6-trimethyl-2-oxo-5,6-dihydro-4*H*-pyrrolo[3,2,1-*ij*]quinolin-1(2*H*)-ylidene)-3-ethyl-2-thioxothiazolidin-4-one 12m 47
^{1}H, ^{13}C NMR, IR and data HPLC-HRMS-ESI spectra of (*Z*)-5-(6',6'-dimethyl-2'-oxo-5',6'-dihydrospiro[cyclohexane-1,4'-pyrrolo[3,2,1-*ij*]quinolin]-1'(2'*H*)-ylidene)-2-thioxothiazolidin-4-one 12n 49
^{1}H, ^{13}C NMR and data HPLC-HRMS-ESI spectra of (*Z*)-5-(6',8'-dimethyl-2'-oxo-5',6'-dihydrospiro[cyclohexane-1,4'-pyrrolo[3,2,1-*ij*]quinolin]-1'(2'*H*)-ylidene)-2-thioxothiazolidin-4-one 12o 51
^{1}H, ^{13}C NMR and data HPLC-HRMS-ESI spectra of (*Z*)-5-(8'-fluoro-6'-methyl-2'-oxo-5',6'-dihydrospiro[cyclopentane-1,4'-pyrrolo[3,2,1-*ij*]quinolin]-1'(2'*H*)-ylidene)-2-thioxothiazolidin-4-one 12p 52
^{1}H, ^{13}C NMR and data HPLC-HRMS-ESI spectra of (*Z*)-5-(8'-fluoro-6'-methyl-2'-oxo-5',6'-dihydrospiro[cycloheptane-1,4'-pyrrolo[3,2,1-*ij*]quinolin]-1'(2'*H*)-ylidene)-2-thioxothiazolidin-4-one 12q 54
^{1}H, ^{13}C NMR, IR and data HPLC-HRMS-ESI spectra of (*Z*)-5-(8-fluoro-4,4,6-trimethyl-2-oxo-6-phenyl-5,6-dihydro-4*H*-pyrrolo[3,2,1-*ij*]quinolin-1(2*H*)-ylidene)-3-(prop-2-yn-1-yl)-2-thioxothiazolidin-4-one 14a 55
^{1}H, ^{13}C NMR and data HPLC-HRMS-ESI spectra of (*Z*)-5-(6-(4-chlorophenyl)-8-fluoro-4,4,6-trimethyl-2-oxo-5,6-dihydro-4*H*-pyrrolo[3,2,1-*ij*]quinolin-1(2*H*)-ylidene)-3-(prop-2-yn-1-yl)-2-thioxothiazolidin-4-one 14b 57
^{1}H, ^{13}C NMR and data HPLC-HRMS-ESI spectra of (*Z*)-8-ethoxy-4,4,6-trimethyl-1-(5-oxo-2-thioxoimidazolidin-4-ylidene)-5,6-dihydro-4*H*-pyrrolo[3,2,1-*ij*]quinolin-2(1*H*)-one 16a 59
^{1}H, ^{13}C NMR, IR and data HPLC-HRMS-ESI spectra of (*Z*)-4,4,6,8-tetramethyl-1-(5-oxo-2-thioxoimidazolidin-4-ylidene)-5,6-dihydro-4*H*-pyrrolo[3,2,1-*ij*]quinolin-2(1*H*)-one 16b60
^{1}H, ^{13}C NMR and data HPLC-HRMS-ESI spectra of (*Z*)-4,4,6-trimethyl-1-(5-oxo-2-thioxoimidazolidin-4-ylidene)-6-phenyl-5,6-dihydro-4*H*-pyrrolo[3,2,1-*ij*]quinolin-2(1*H*)-one 16c 62
^{1}H, ^{13}C NMR, IR and data HPLC-HRMS-ESI spectra of (*Z*)-8-fluoro-4,4,6-trimethyl-1-(5-oxo-2-thioxoimidazolidin-4-ylidene)-6-phenyl-5,6-dihydro-4*H*-pyrrolo[3,2,1-*ij*]quinolin-2(1*H*)-one 16d 64
^{1}H, ^{13}C NMR and data HPLC-HRMS-ESI spectra of (*Z*)-6-(4-chlorophenyl)-4,4,6,8-tetramethyl-1-(5-oxo-2-thioxoimidazolidin-4-ylidene)-5,6-dihydro-4*H*-pyrrolo[3,2,1-*ij*]quinolin-2(1*H*)-on 16e 66

^{1}H, ^{13}C NMR, IR and data HPLC-HRMS-ESI spectra of 8-bromo-4,4,6-trimethyl-6-phenyl-5,6-dihydro-4*H*-pyrrolo[3,2,1-*ij*]quinoline-1,2-dione 6

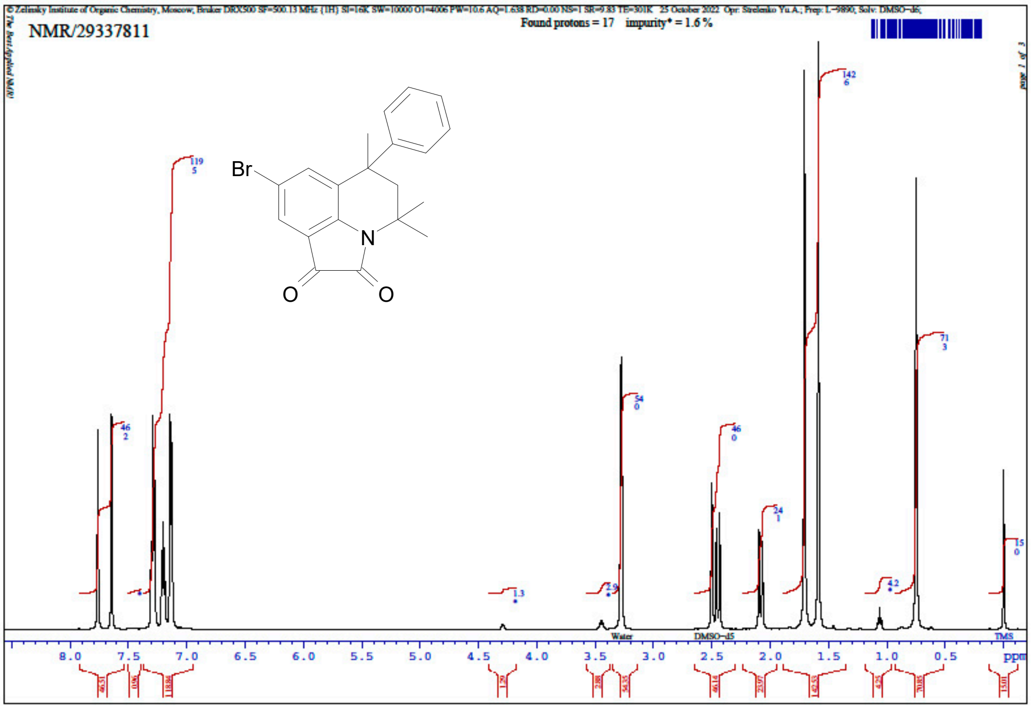

Figure S1. ^{1}H NMR spectrum of 5,6-dihydro-4*H*-pyrrolo[3,2,1-*ij*]quinoline-1,2-dione **6**

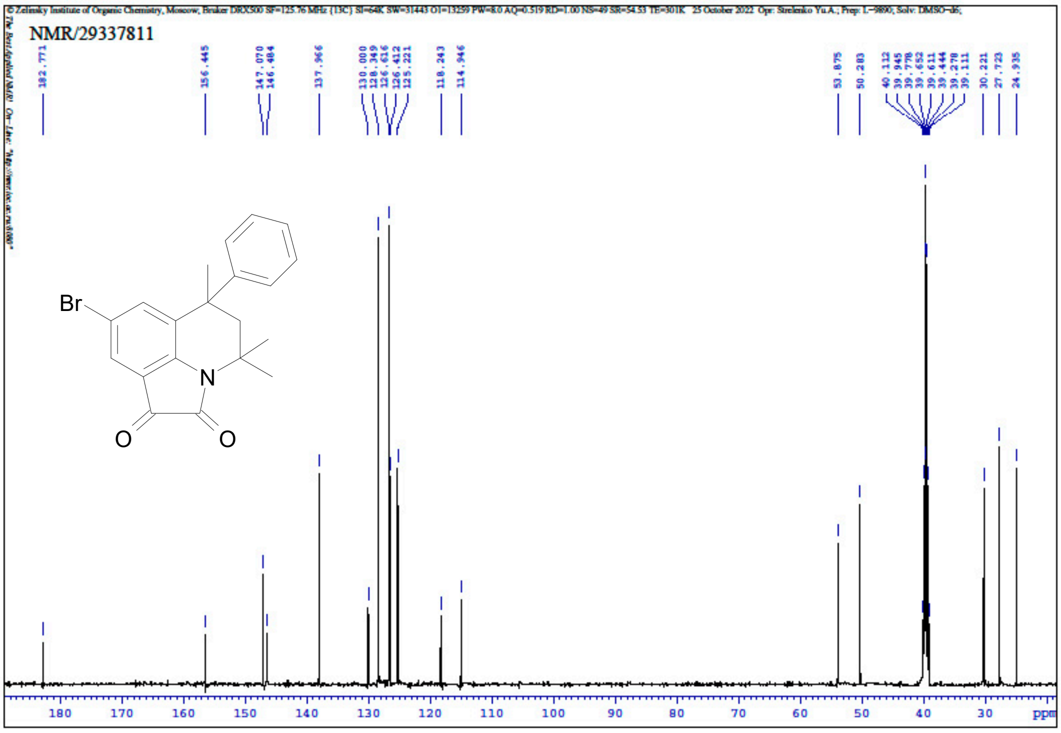

Figure S2. ^{13}C NMR spectrum of 5,6-dihydro-4*H*-pyrrolo[3,2,1-*ij*]quinoline-1,2-dione **6**

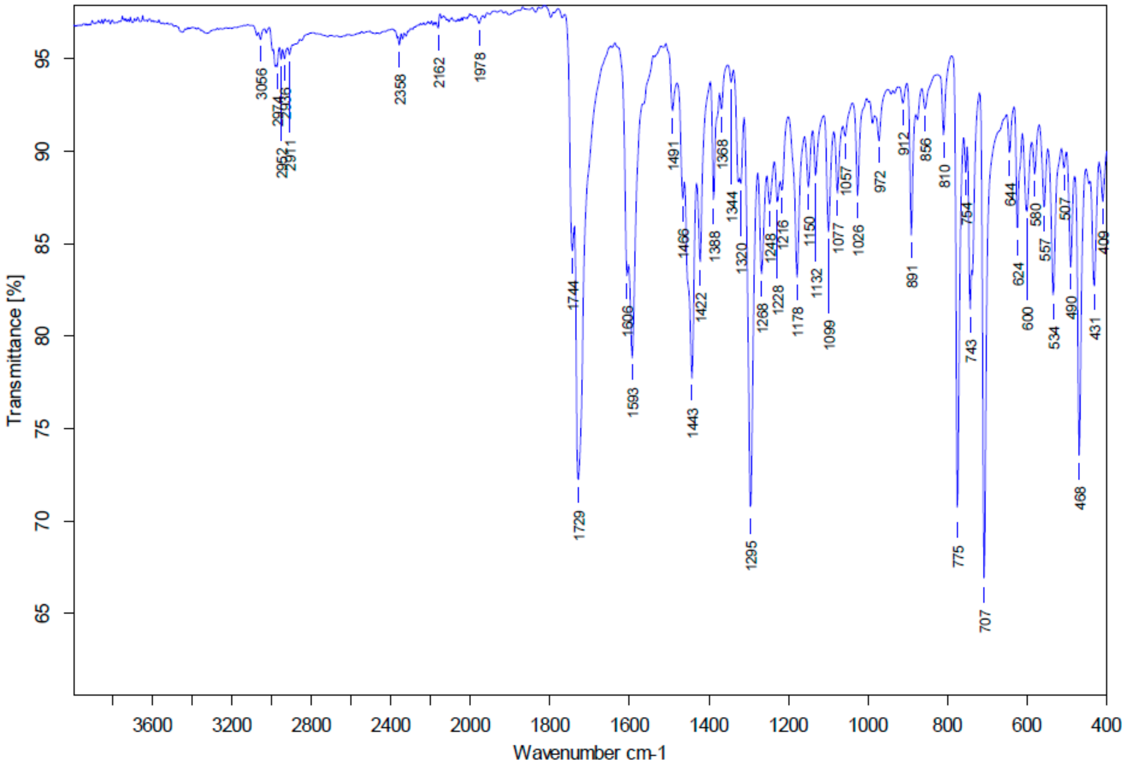

Figure S3. IR spectrum of 5,6-dihydro-4*H*-pyrrolo[3,2,1-*ij*]quinoline-1,2-dione **6**

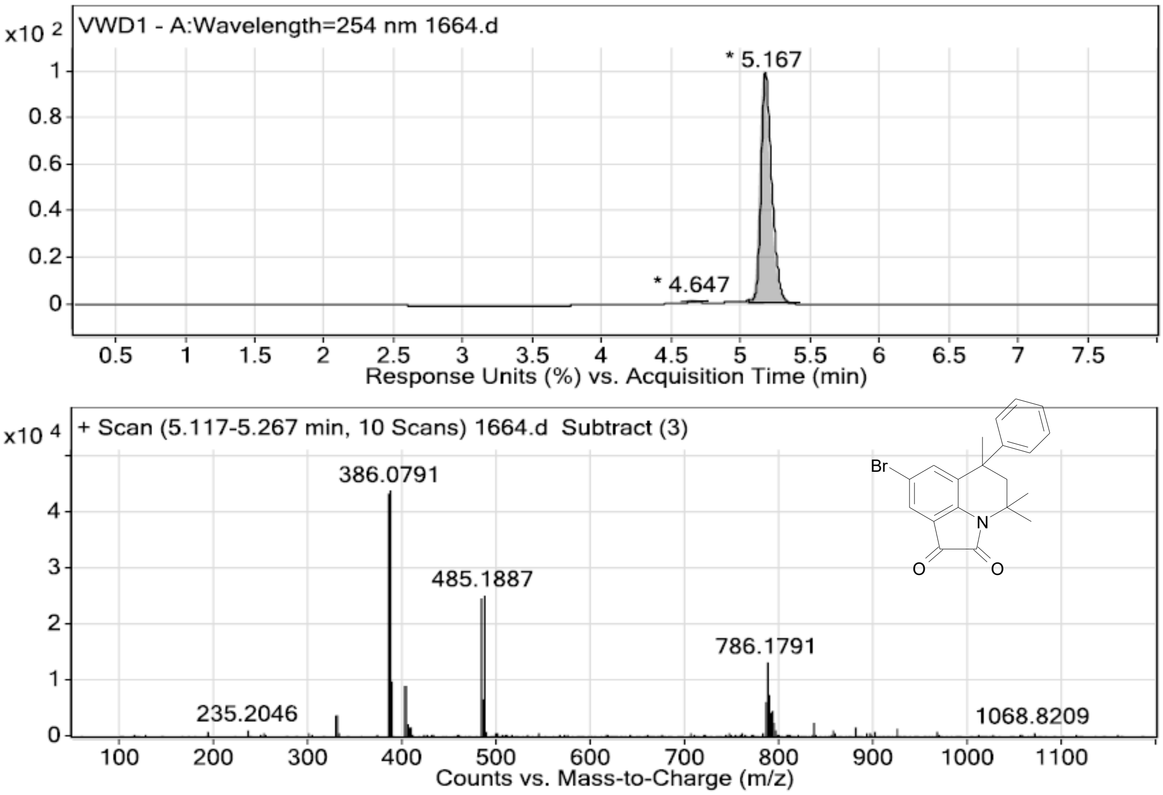

Figure S4. Data of HPLC-MS-ESI analysis of **6**

^{1}H, ^{13}C NMR and data HPLC-HRMS-ESI spectra of 6-iodo-2,2,4-trimethyl-1,2,3,4-tetrahydroquinoline hydrochloride 8a

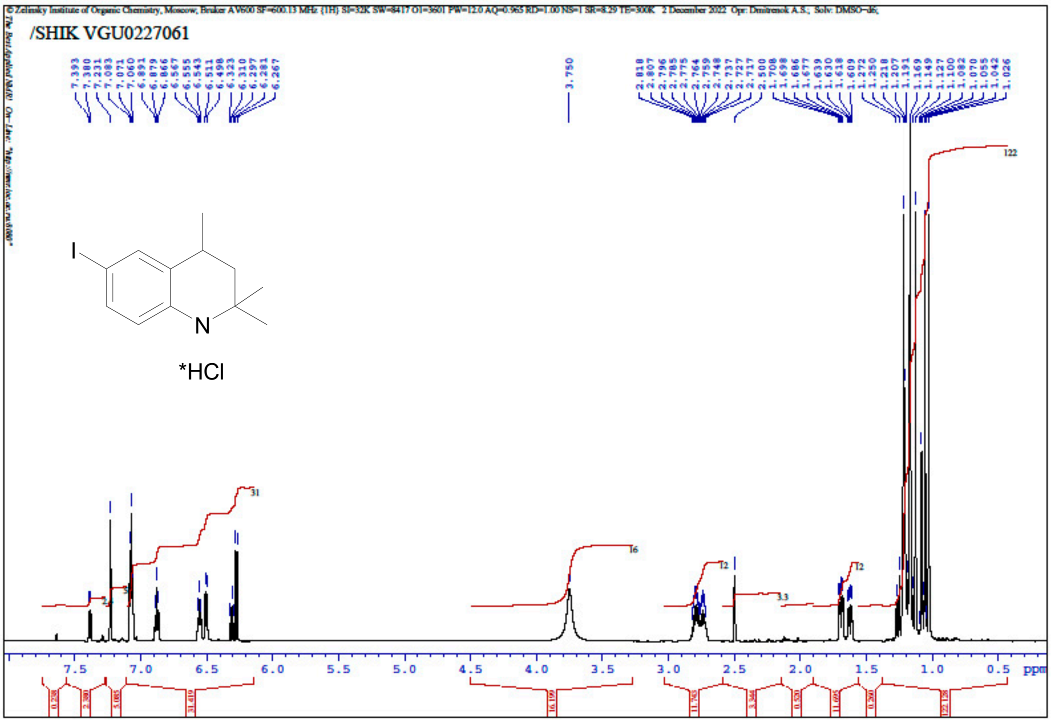

Figure S5. ^{1}H NMR spectrum of 6-iodo-2,2,4-trimethyl-1,2,3,4-tetrahydroquinoline hydrochloride **8a**

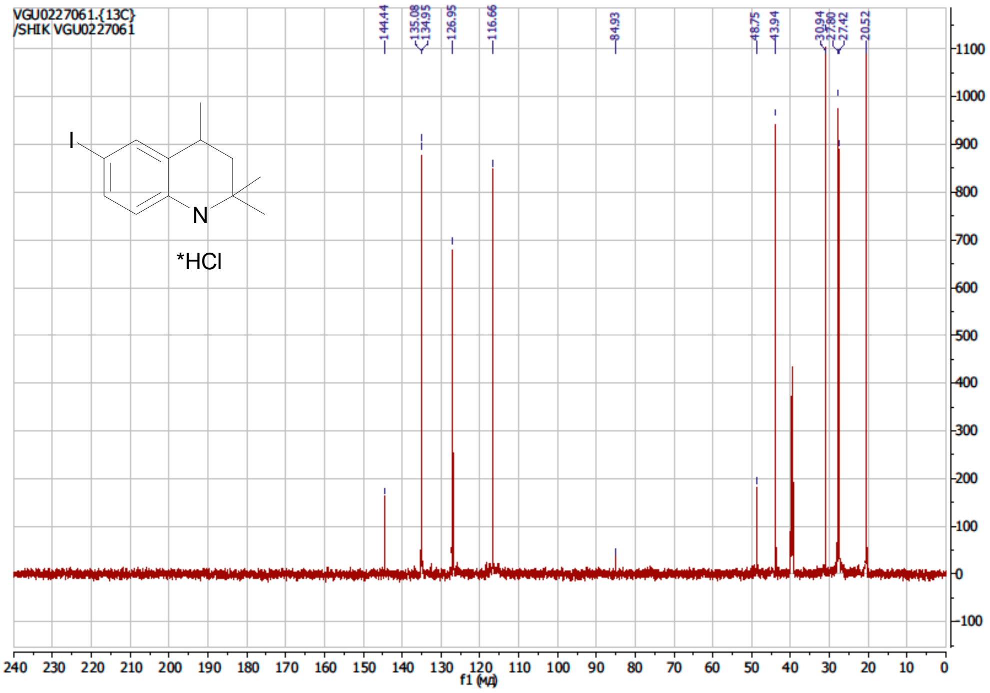

Figure S6. ^{13}C NMR spectrum of 6-iodo-2,2,4-trimethyl-1,2,3,4-tetrahydroquinoline hydrochloride **8a**

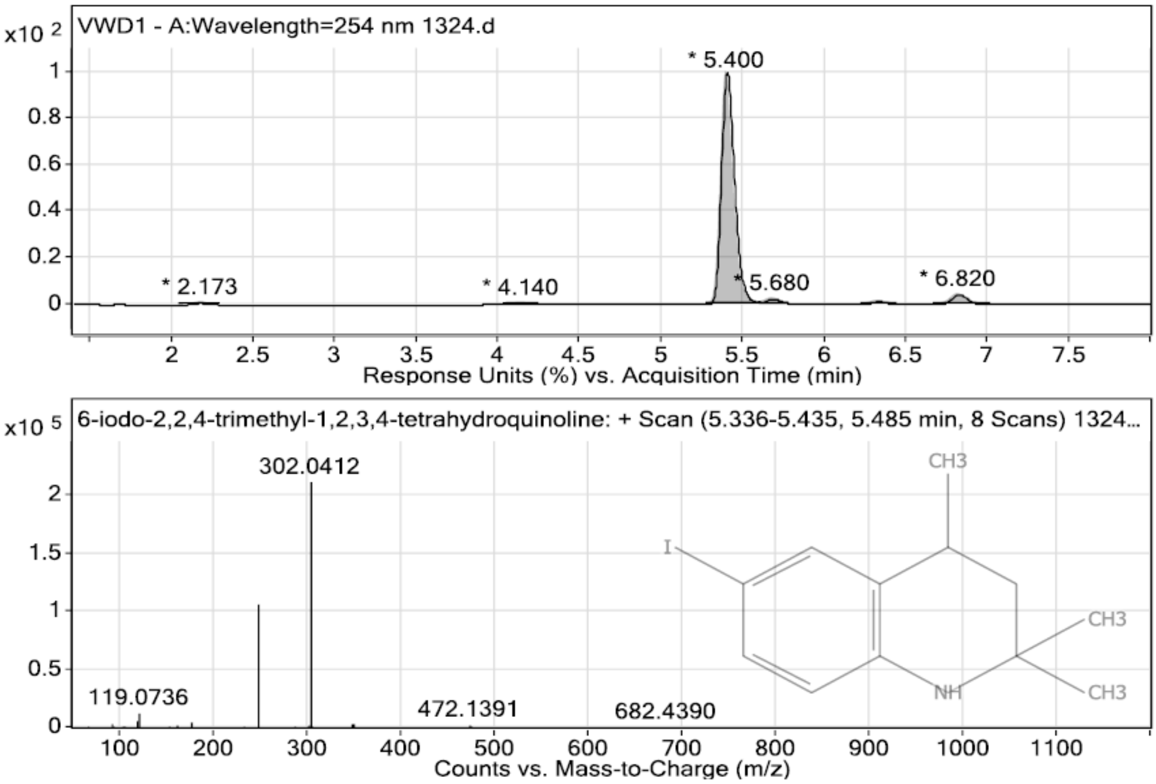

Figure S7. Data of HPLC-MS-ESI analysis of **8a**

^{1}H, ^{13}C NMR, IR and data HPLC-HRMS-ESI spectra of 6-iodo-2,2,4-trimethyl-4-phenyl-1,2,3,4-tetrahydroquinoline hydrochloride 8b

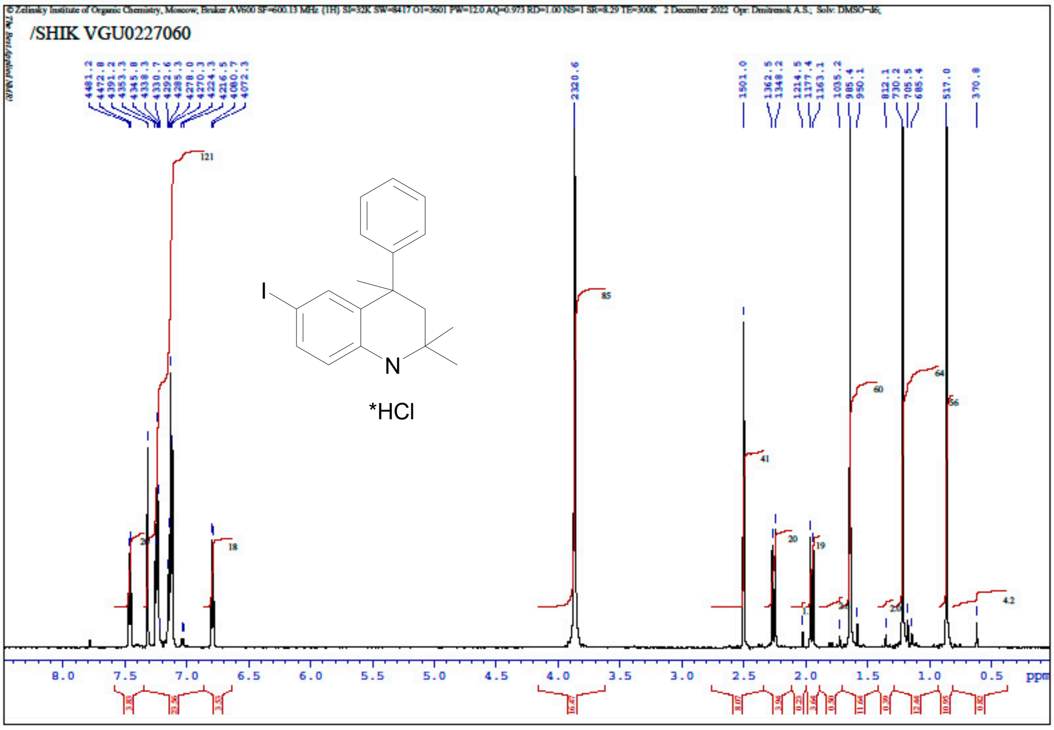

Figure S8. ^{1}H NMR spectrum of 6-iodo-2,2,4-trimethyl-4-phenyl-1,2,3,4-tetrahydroquinoline hydrochloride **8b**

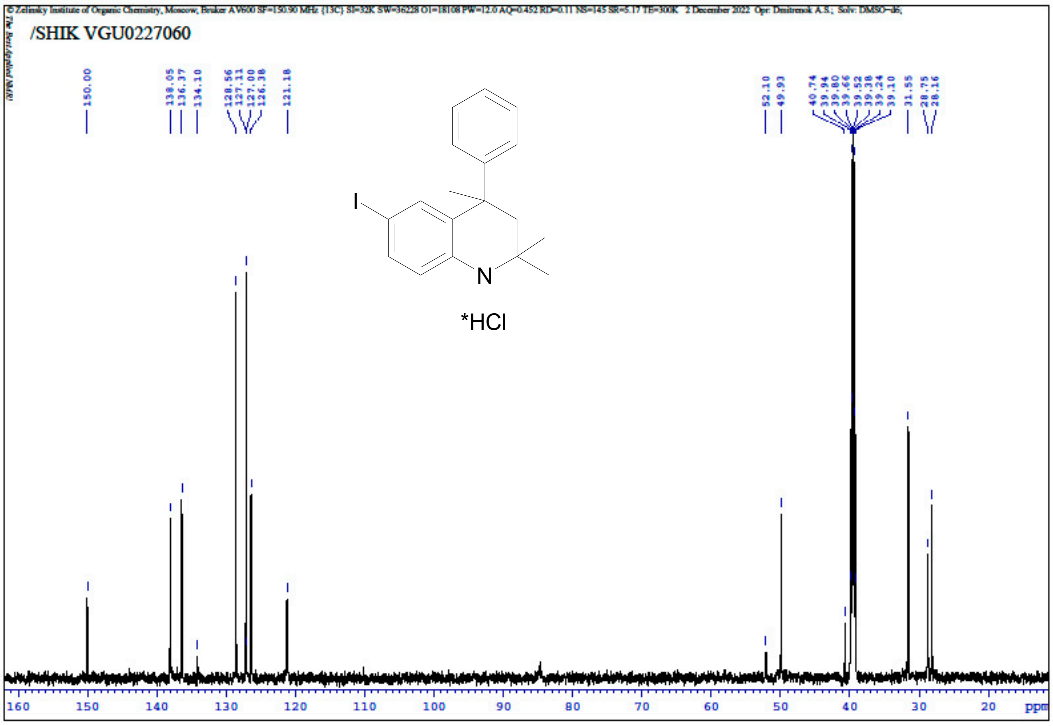

Figure S9. ^{13}C NMR spectrum of 6-iodo-2,2,4-trimethyl-4-phenyl-1,2,3,4-tetrahydroquinoline hydrochloride **8b**

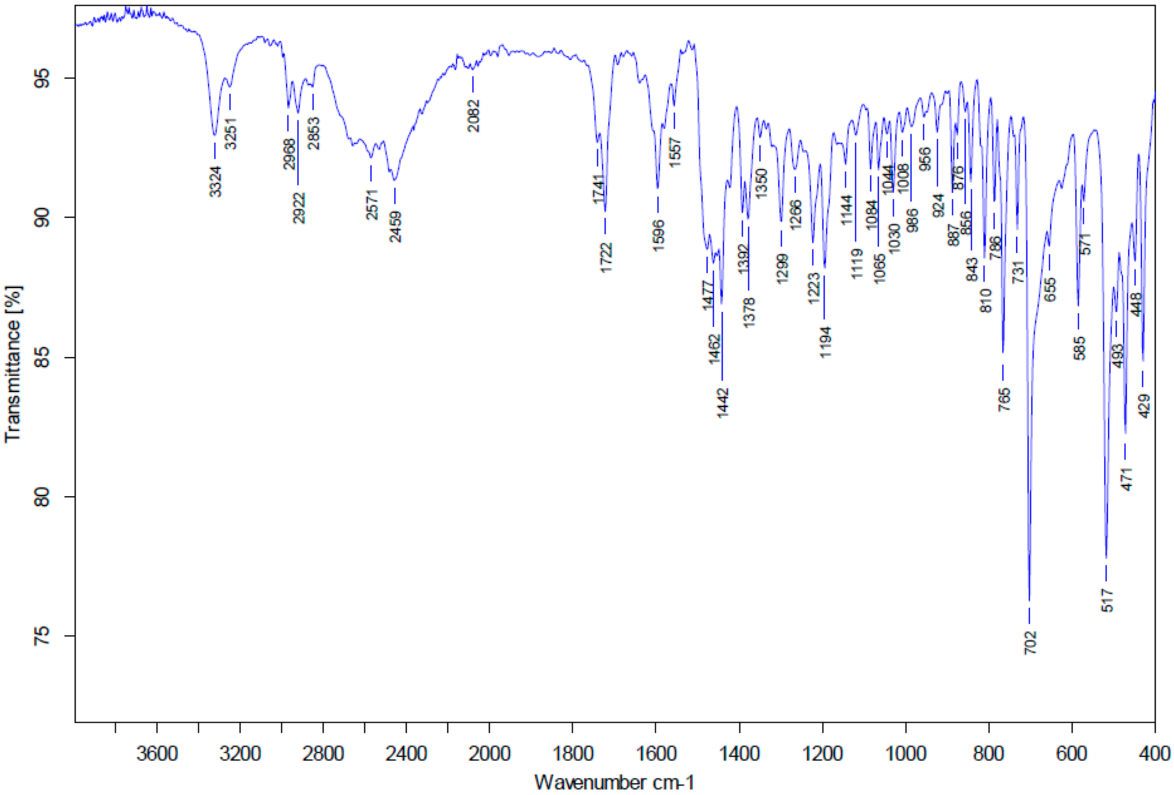

Figure S10. IR spectrum of **8b**

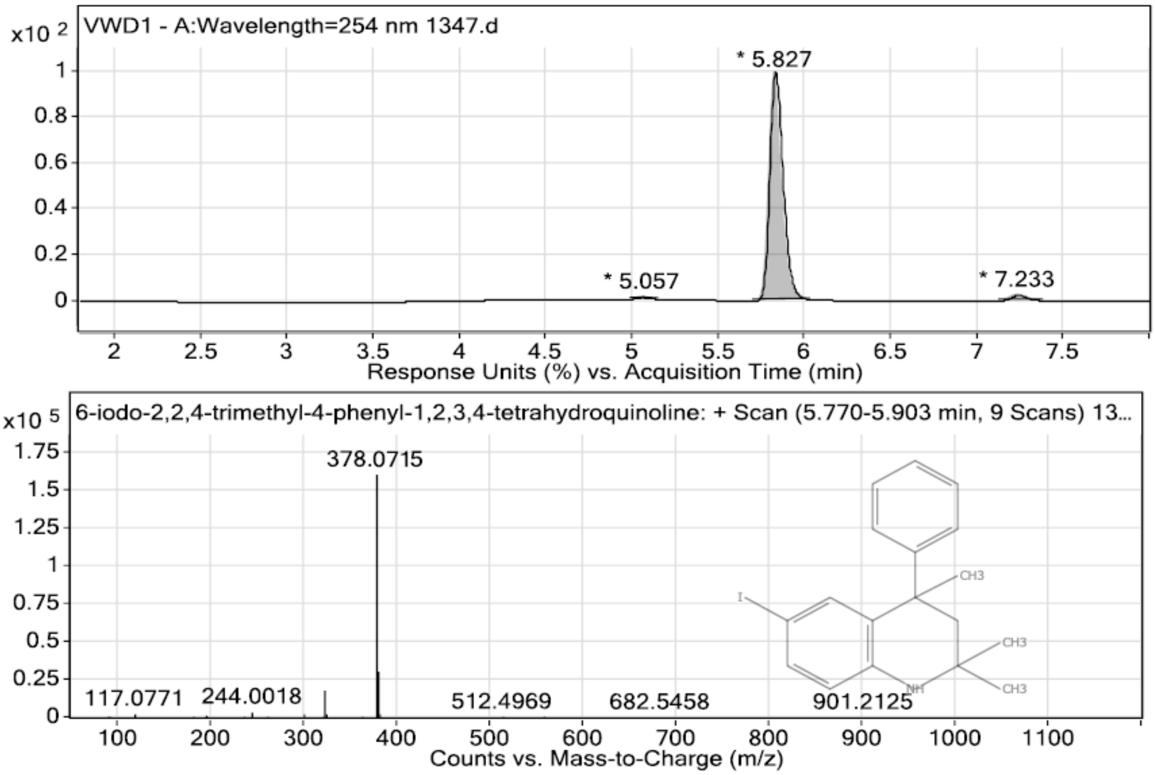

Figure S11. Data of HPLC-MS-ESI analysis of **8b**

^{1}H, ^{13}C NMR, IR and data HPLC-HRMS-ESI spectra of 4-(4-chlorophenyl)-6-iodo-2,2,4-trimethyl-1,2,3,4-tetrahydroquinoline hydrochloride 8c

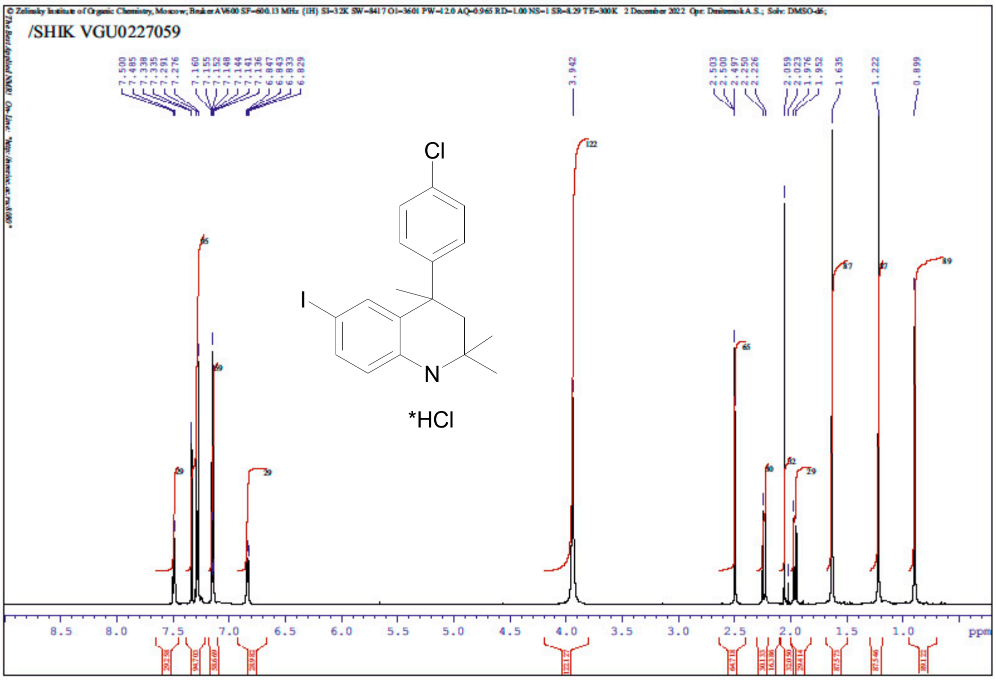

Figure S12. ^{1}H NMR spectrum of 4-(4-chlorophenyl)-6-iodo-2,2,4-trimethyl-1,2,3,4-tetrahydroquinoline hydrochloride **8c**

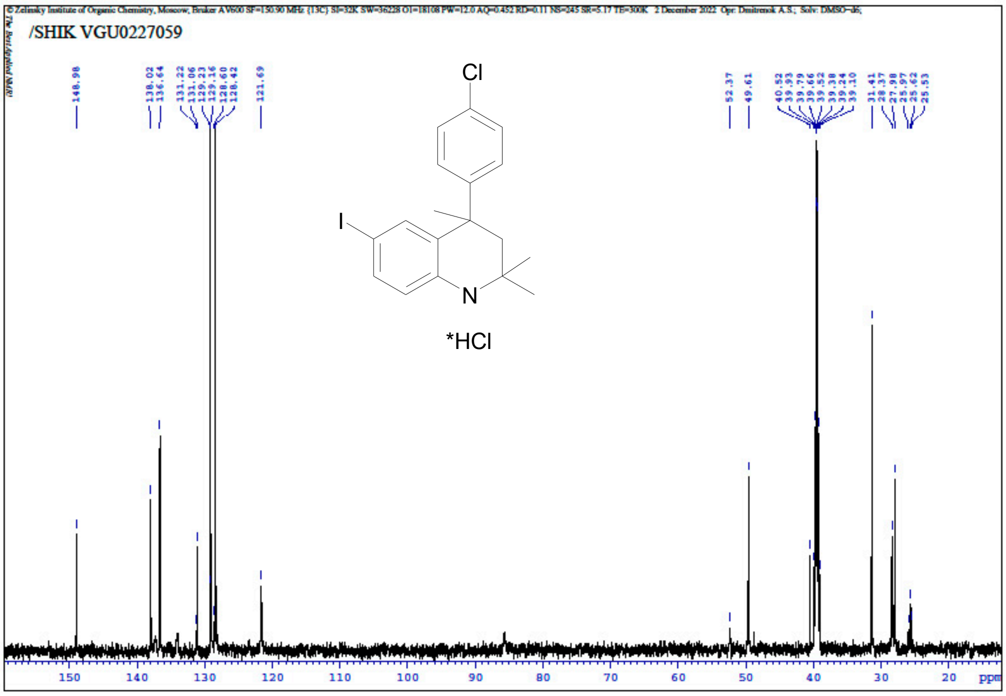

Figure S13. ^{1}H NMR spectrum of 4-(4-chlorophenyl)-6-iodo-2,2,4-trimethyl-1,2,3,4-tetrahydroquinoline hydrochloride **8c**

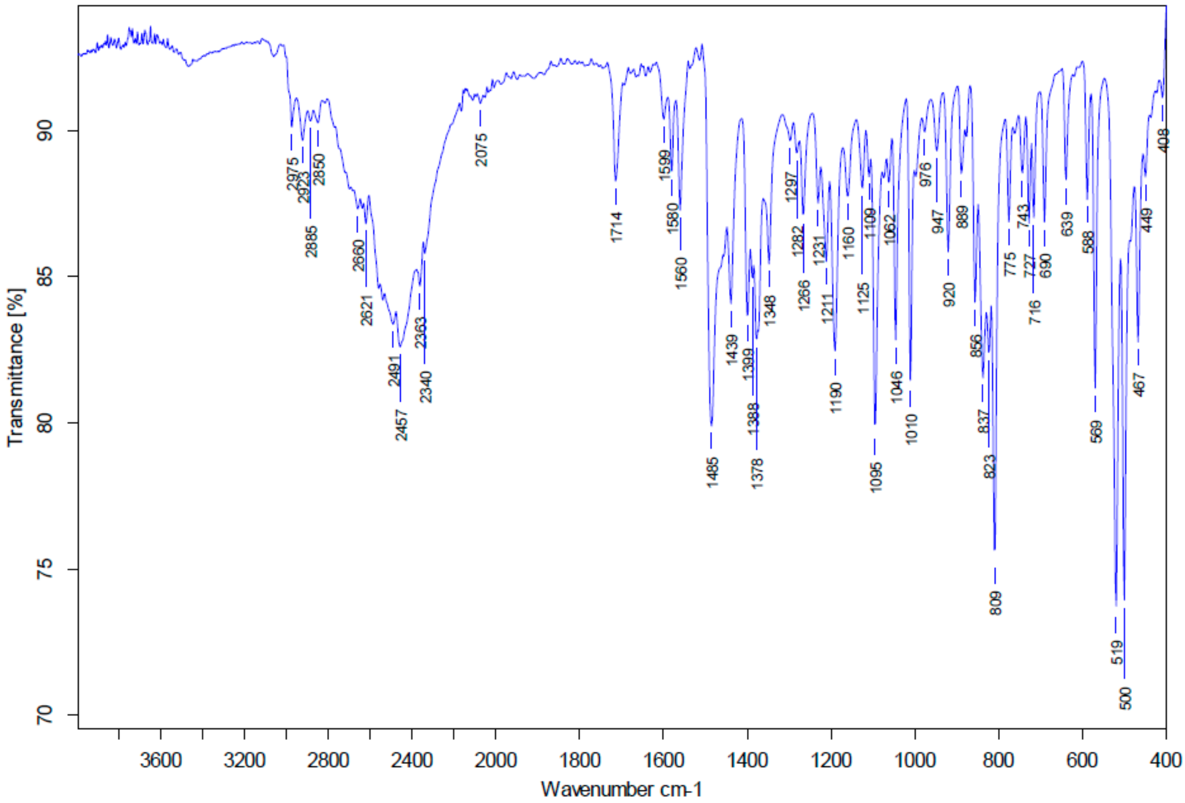

Figure S14. IR spectrum of **8c**

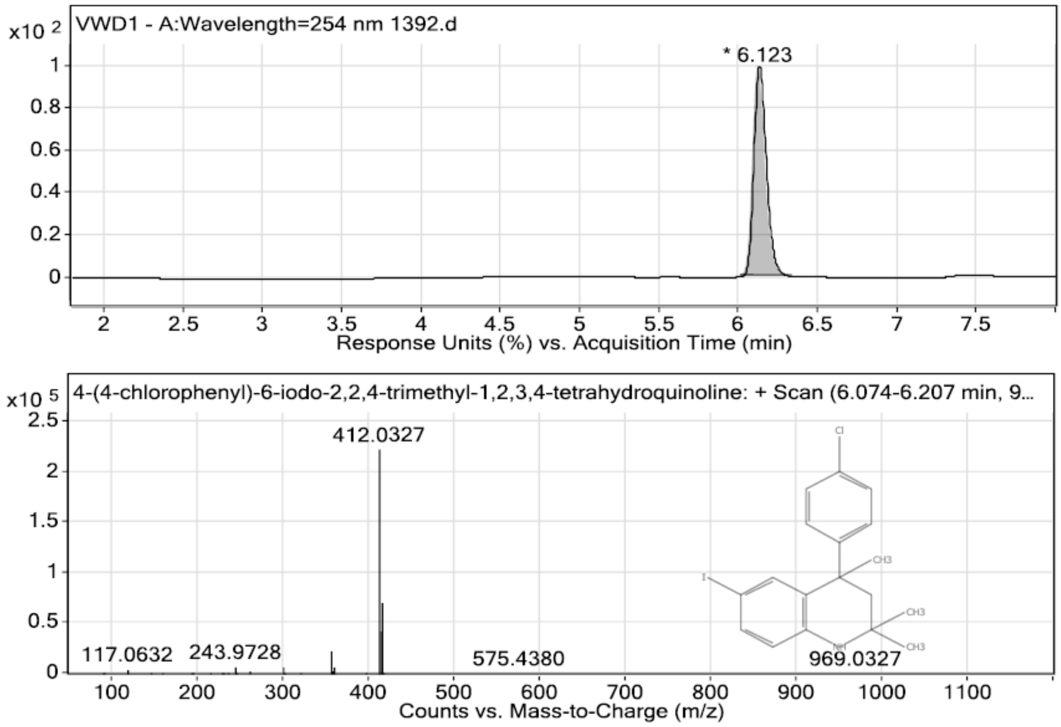

Figure S15. Data of HPLC-MS-ESI analysis of **8c**

^{1}H, ^{13}C NMR, IR and data HPLC-HRMS-ESI spectra of 8-iodo-4,4,6-trimethyl-5,6-dihydro-4*H*-pyrrolo[3,2,1-*ij*]quinoline-1,2-dione 9a

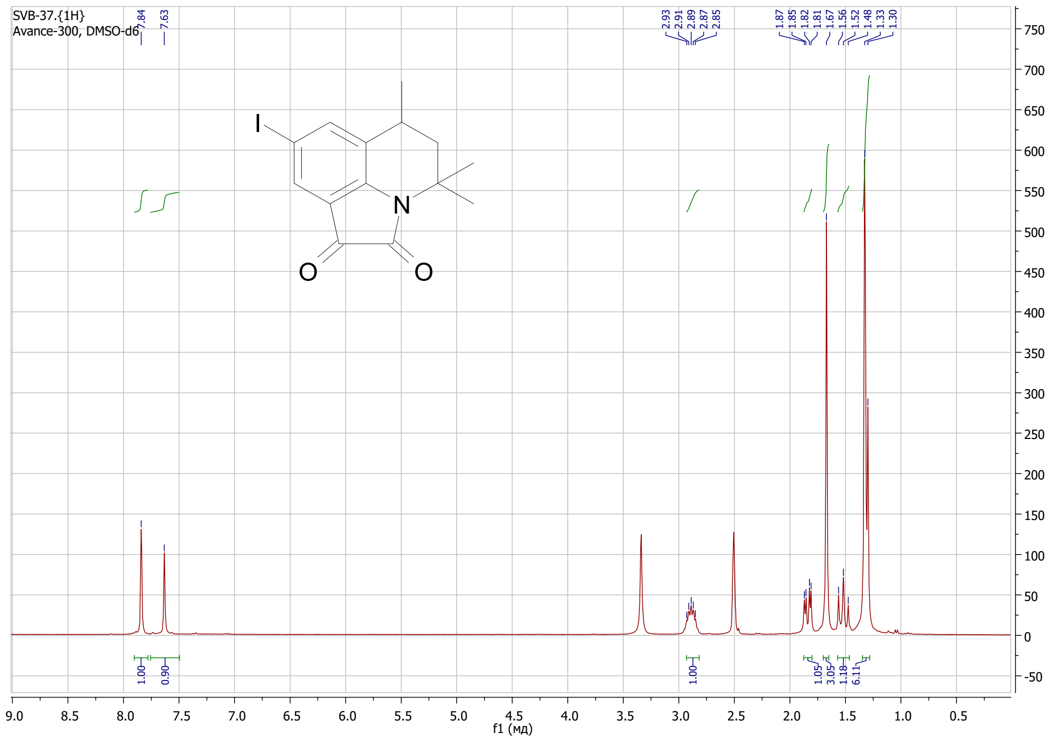

Figure S16. ^{1}H NMR spectrum of 5,6-dihydro-4*H*-pyrrolo[3,2,1-*ij*]quinoline-1,2-dione **9a**

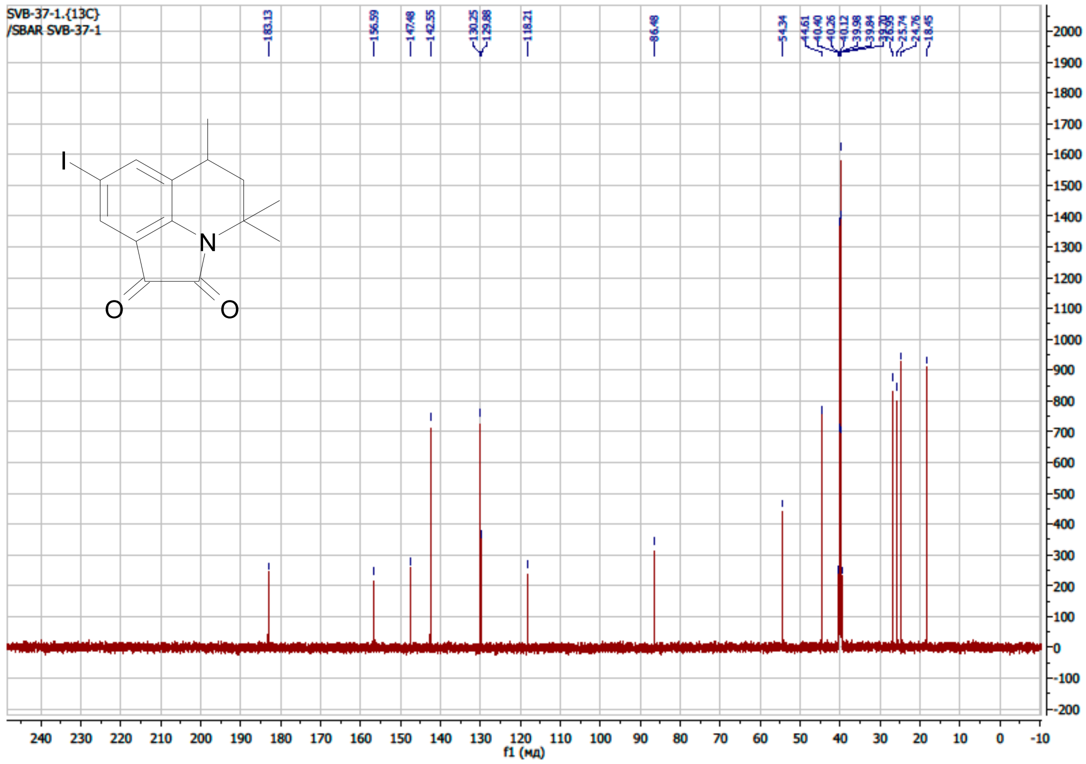

Figure S17. ^{13}C NMR spectrum of 5,6-dihydro-4*H*-pyrrolo[3,2,1-*ij*]quinoline-1,2-dione **9a**

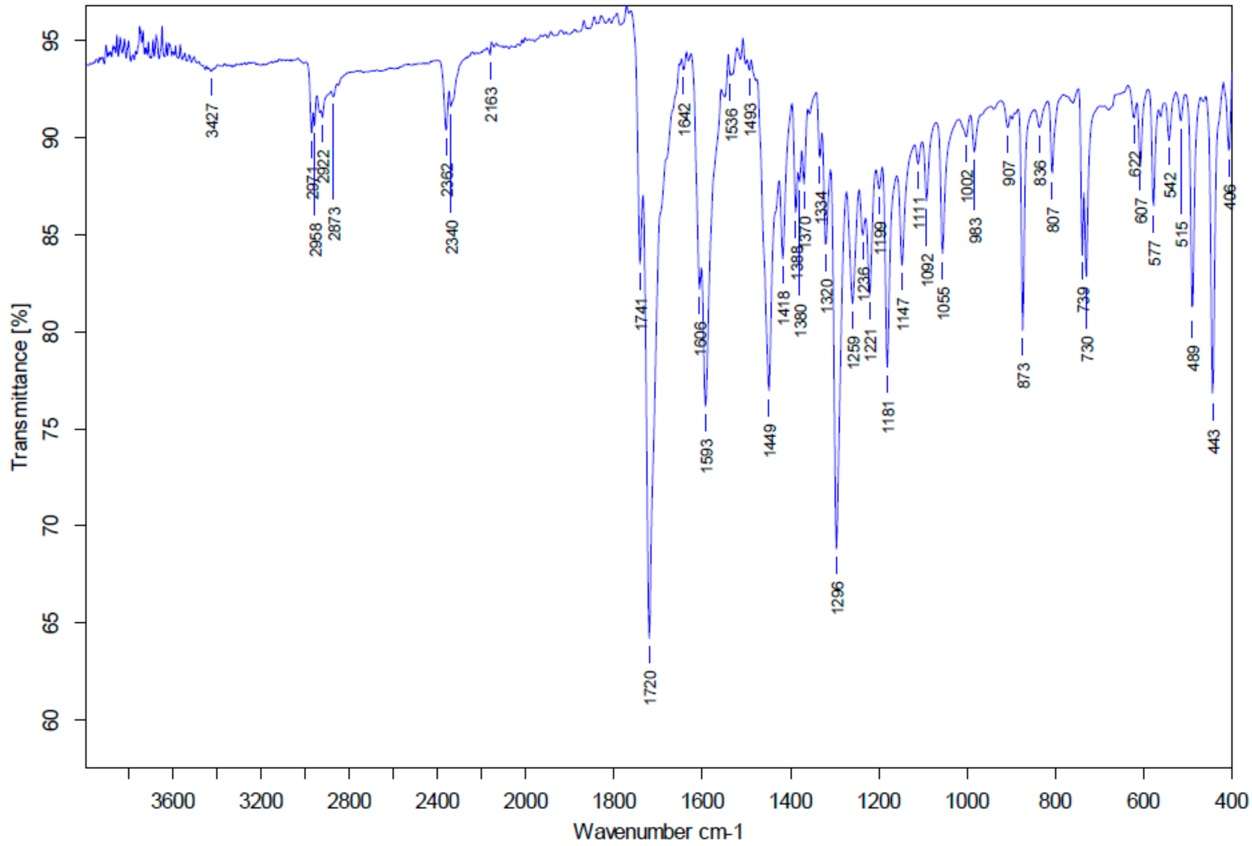

Figure S18. IR spectrum of **9a**

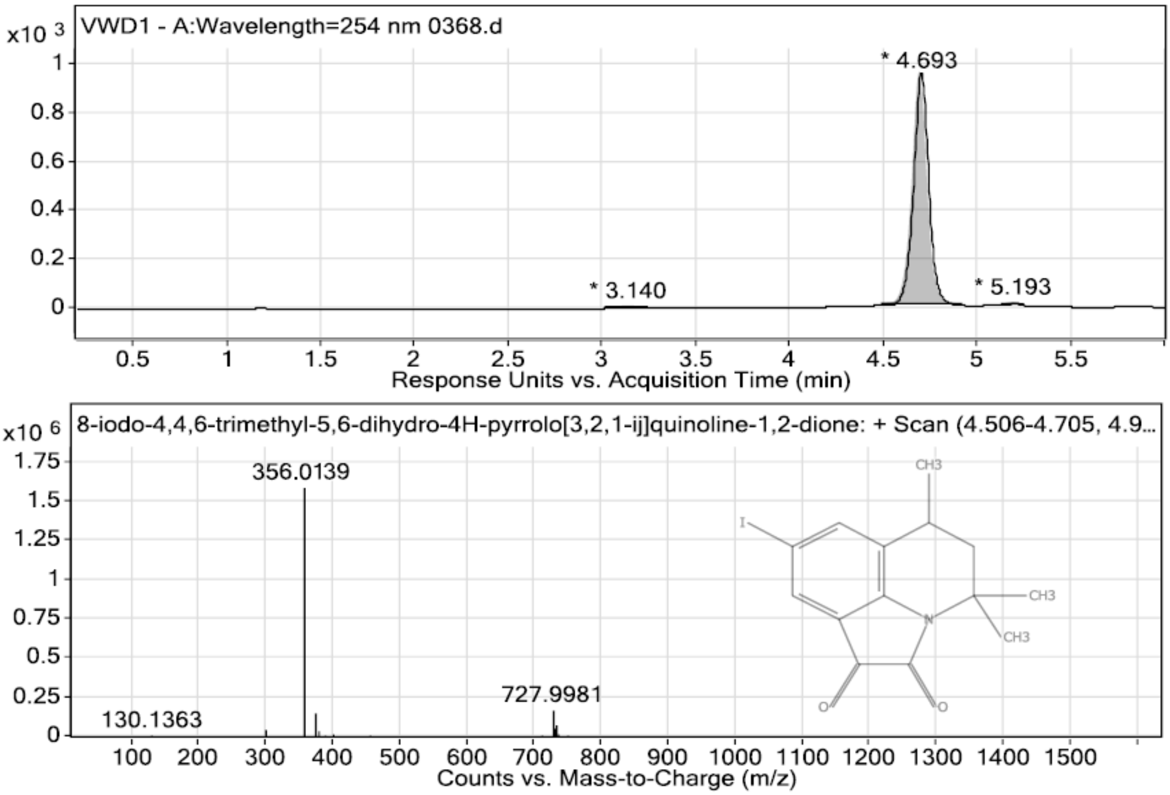

Figure S19. Data of HPLC-MS-ESI analysis of **9a**

^{1}H, ^{13}C NMR, IR and data HPLC-HRMS-ESI spectra of 8-iodo-4,4,6-trimethyl-6-phenyl-5,6-dihydro-4*H*-pyrrolo[3,2,1-*ij*]quinoline-1,2-dione 9b

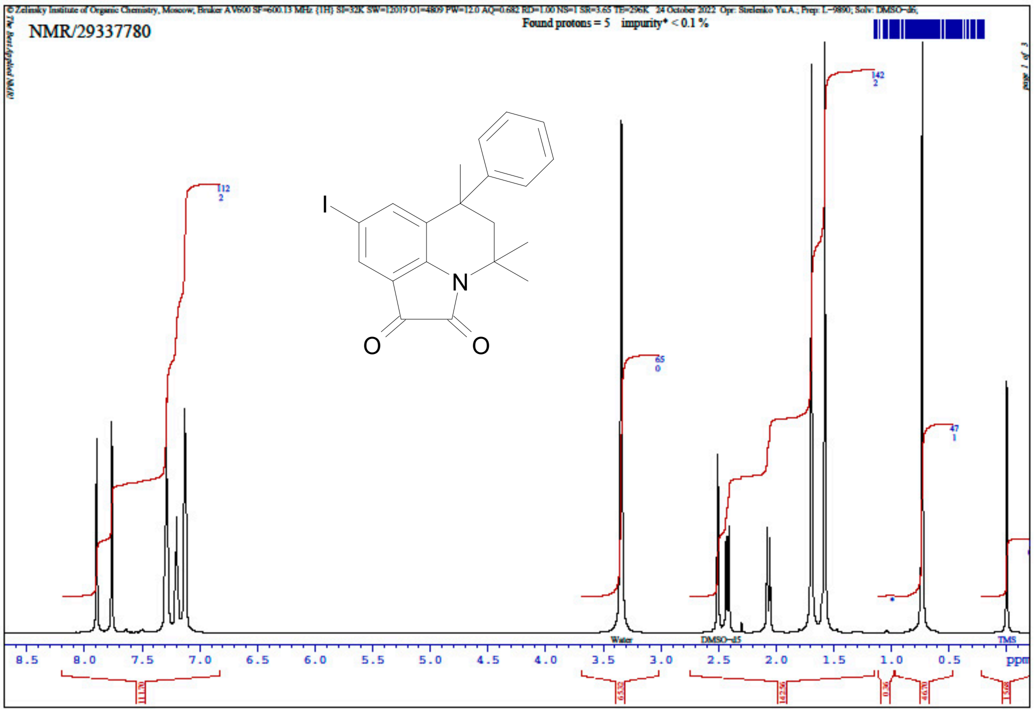

Figure S20. ^{1}H NMR spectrum of 5,6-dihydro-4*H*-pyrrolo[3,2,1-*ij*]quinoline-1,2-dione **9b**

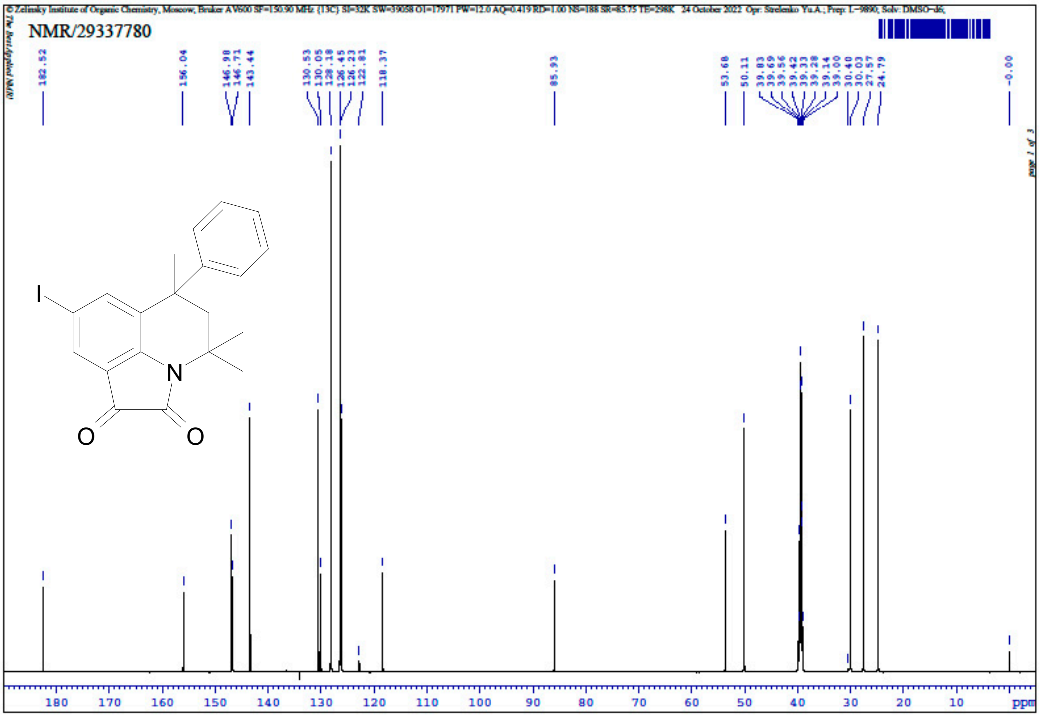

Figure S21. ^{13}C NMR spectrum of 5,6-dihydro-4*H*-pyrrolo[3,2,1-*ij*]quinoline-1,2-dione **9b**

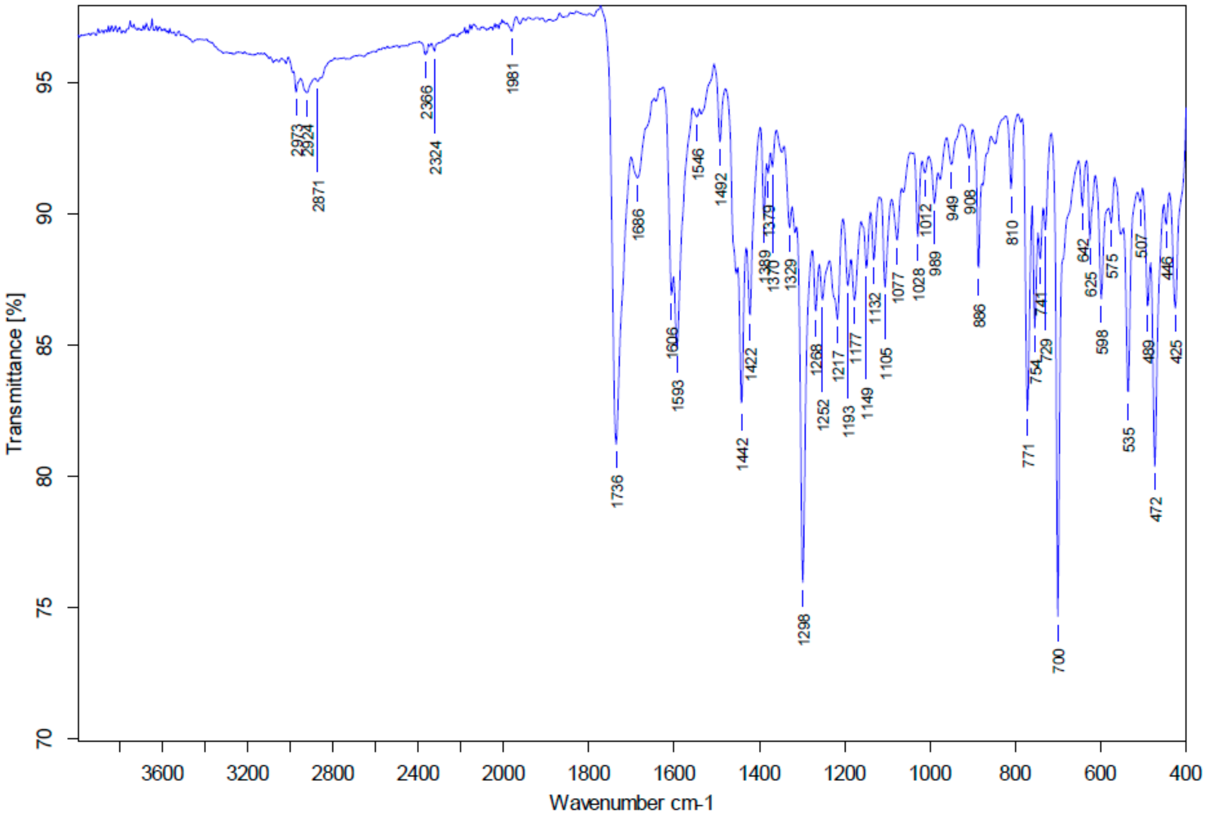

Figure S22. IR spectrum of **9b**

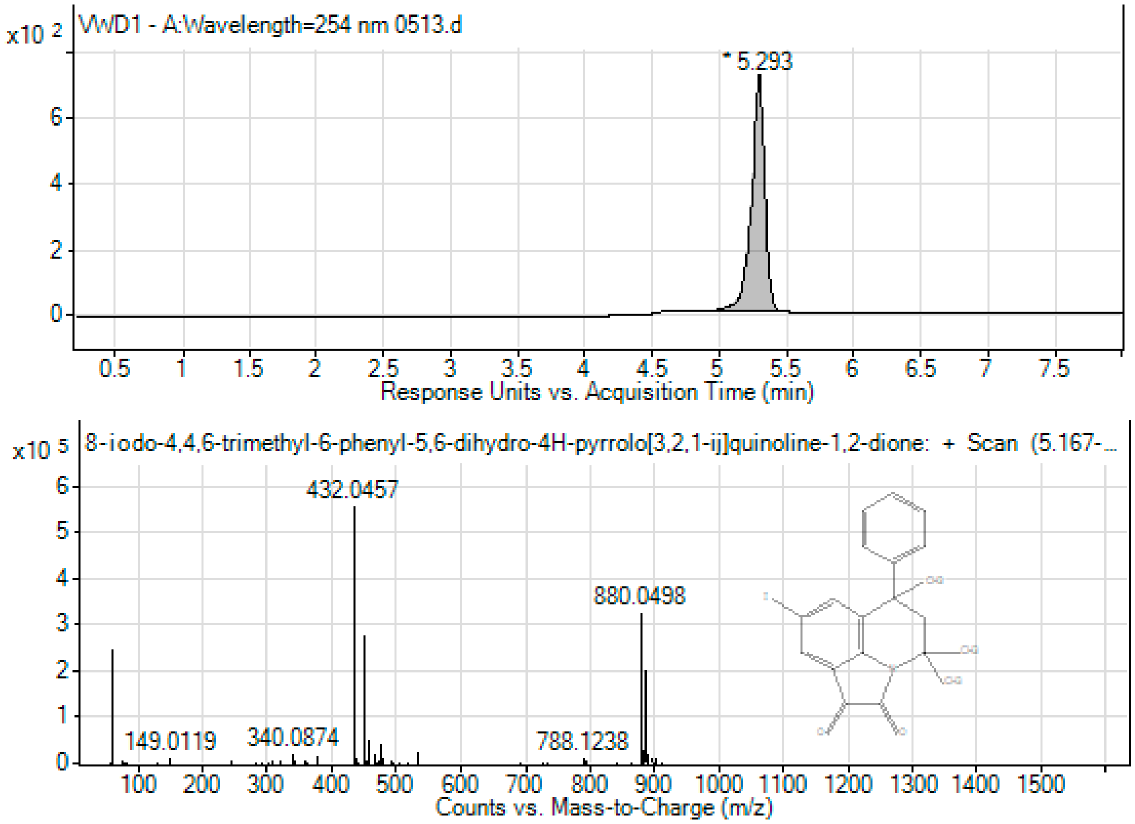

Figure S23. Data of HPLC-MS-ESI analysis of **9b**

^{1}H, ^{13}C NMR and data HPLC-HRMS-ESI spectra of 6-(4-chlorophenyl)-8-iodo-4,4,6-trimethyl-5,6-dihydro-4*H*-pyrrolo[3,2,1-*ij*]quinoline-1,2-dione 9c

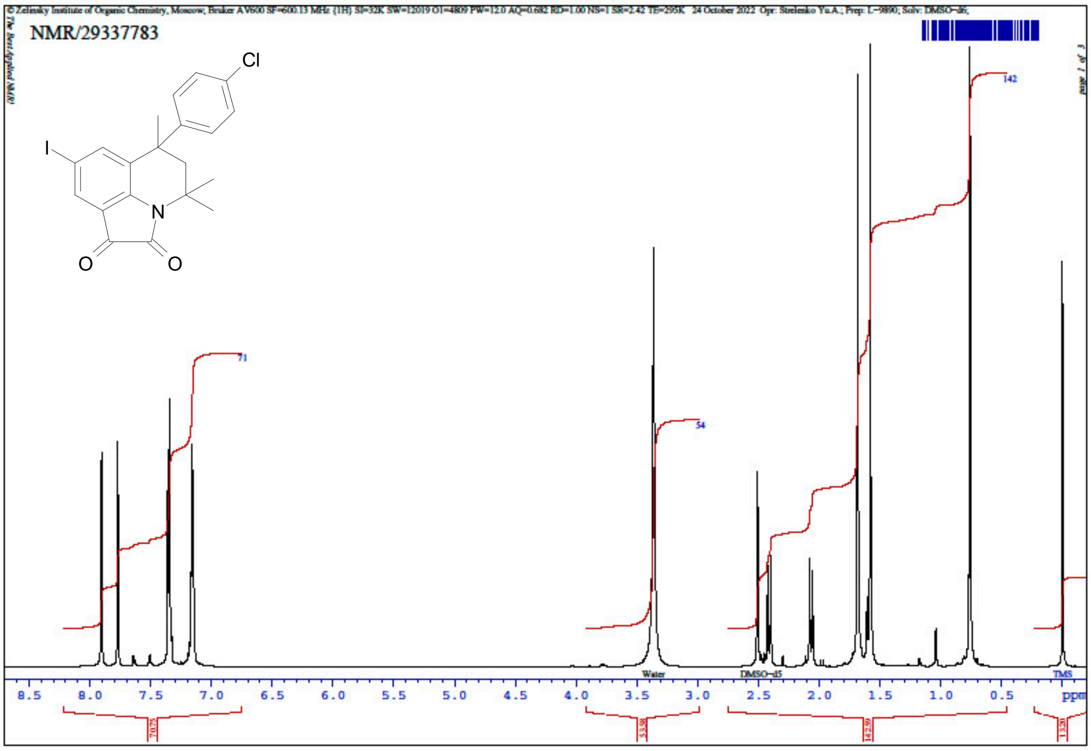

Figure S24. ^{1}H NMR spectrum of 5,6-dihydro-4*H*-pyrrolo[3,2,1-*ij*]quinoline-1,2-dione **9c**

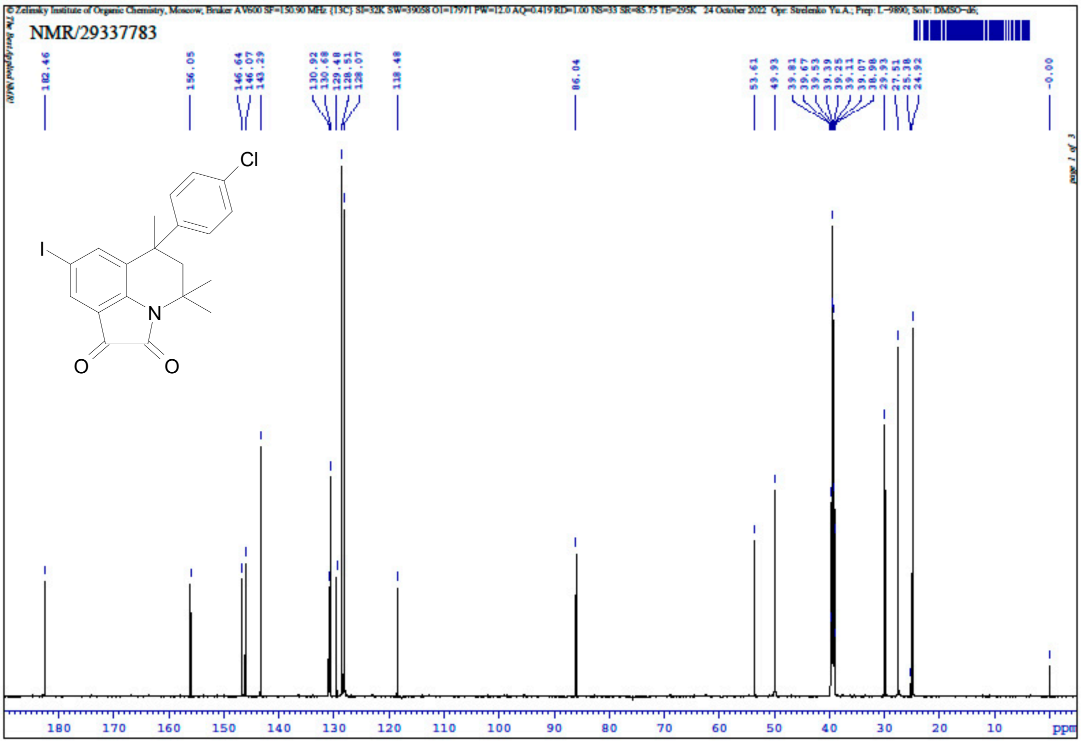

Figure S25. ^{13}C NMR spectrum of 5,6-dihydro-4*H*-pyrrolo[3,2,1-*ij*]quinoline-1,2-dione **9c**

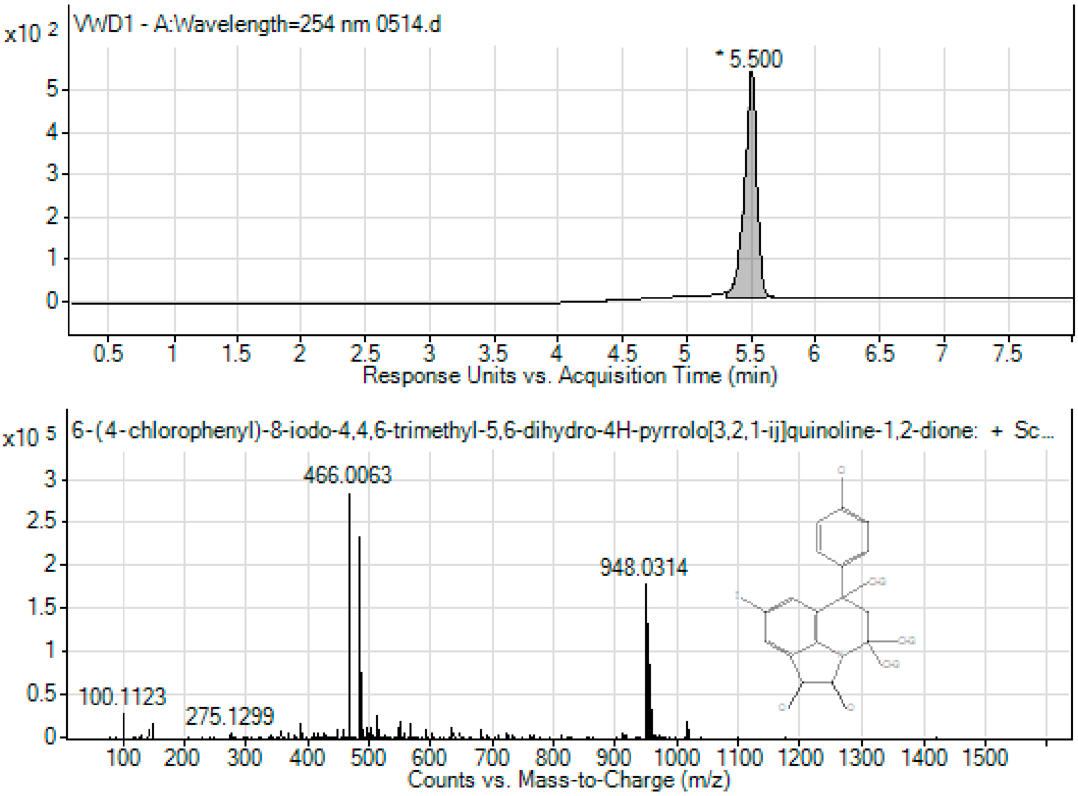

Figure S26. Data of HPLC-MS-ESI analysis of **9c**

^{1}H, ^{13}C NMR and data HPLC-HRMS-ESI spectra of 4,4,6-trimethyl-1,2-dioxo-1,2,5,6-tetrahydro-4*H*-pyrrolo[3,2,1-*ij*]quinolin-8-yl-2-methoxybenzoate 10c

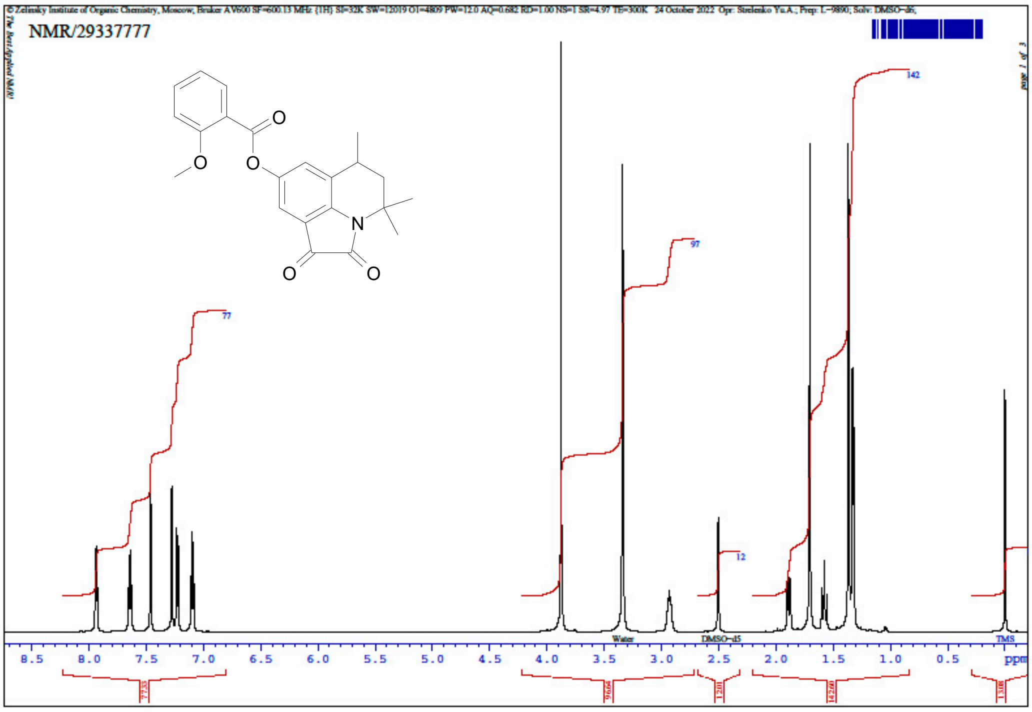

Figure S27. ^{1}H NMR spectrum of 5,6-dihydro-4*H*-pyrrolo[3,2,1-*ij*]quinoline-1,2-dione **10c**

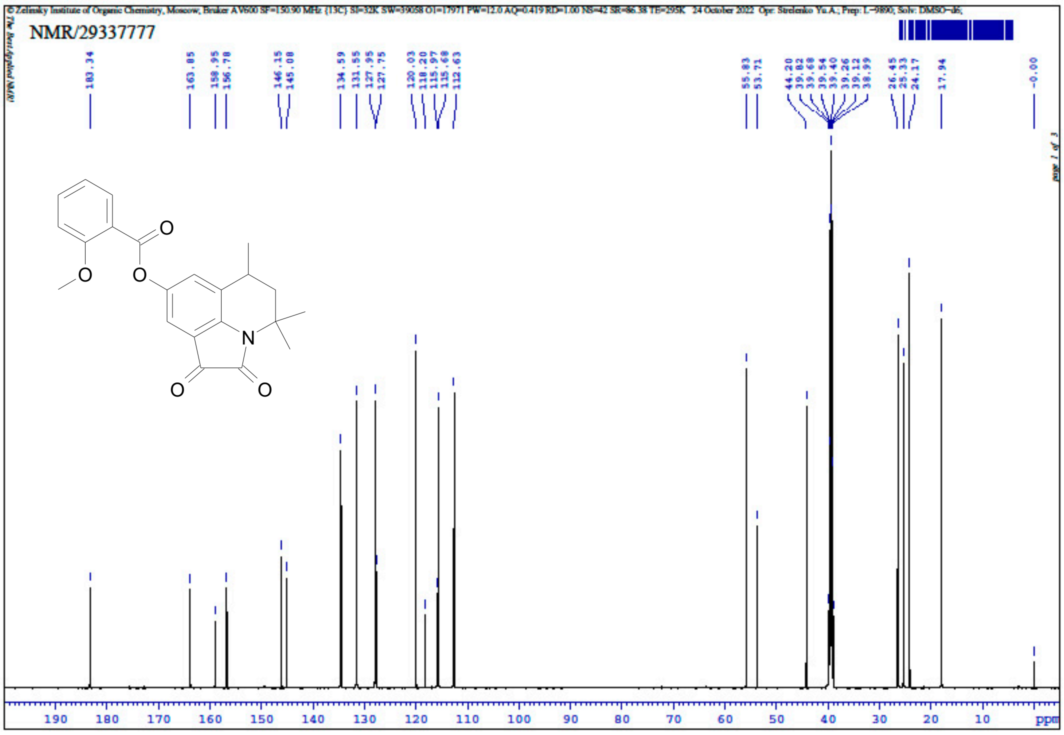

Figure S28. ^{13}C NMR spectrum of 5,6-dihydro-4*H*-pyrrolo[3,2,1-*ij*]quinoline-1,2-dione **10c**

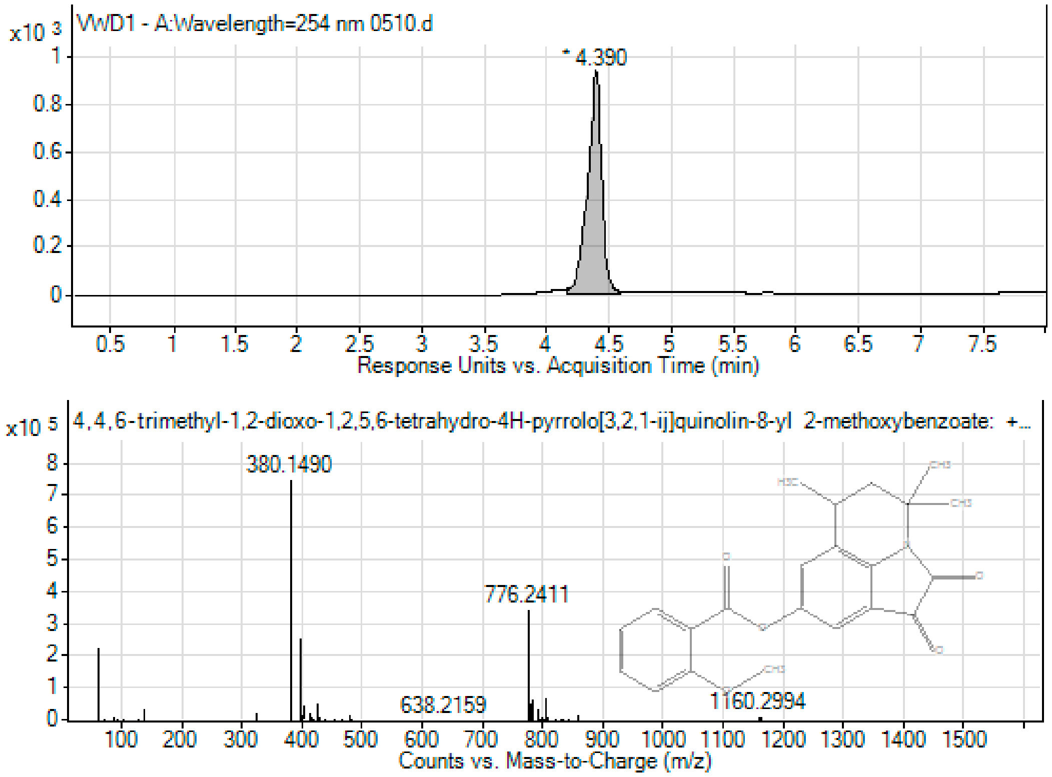

Figure S29. Data of HPLC-MS-ESI analysis of **10c**

^{1}H, ^{13}C NMR and data HPLC-HRMS-ESI spectra of 4,4,6-trimethyl-1,2-dioxo-1,2,5,6-tetrahydro-4*H*-pyrrolo[3,2,1-*ij*]quinolin-8-yl 3,4,5-trimethoxybenzoate 10d

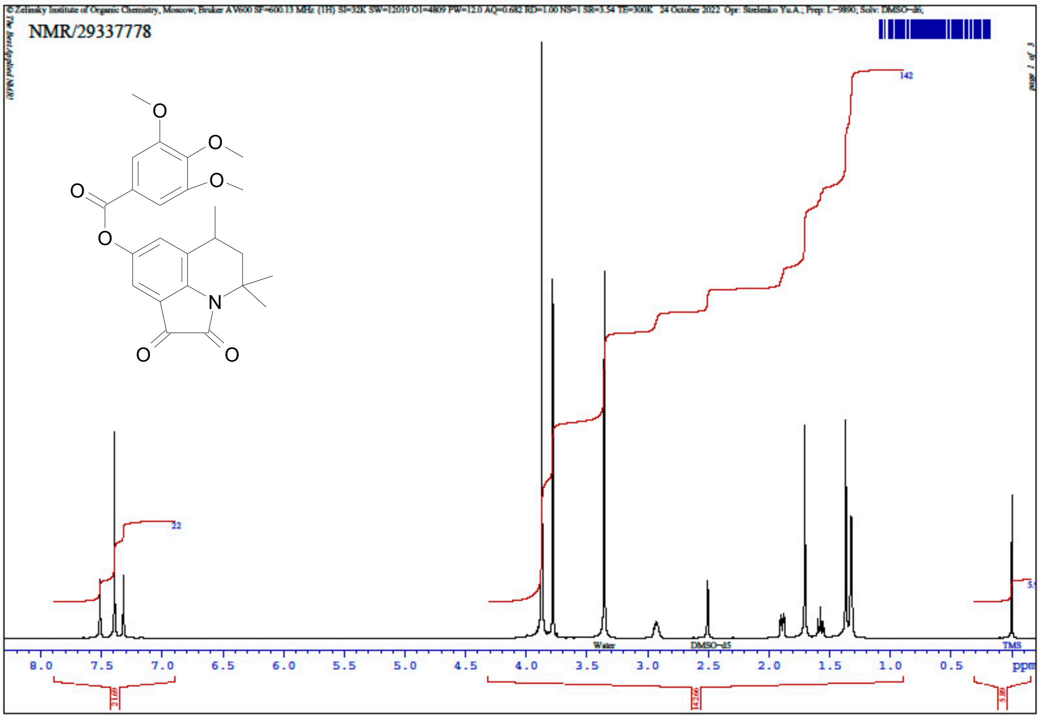

Figure S30. ^{1}H NMR spectrum of 5,6-dihydro-4*H*-pyrrolo[3,2,1-*ij*]quinoline-1,2-dione **10d**

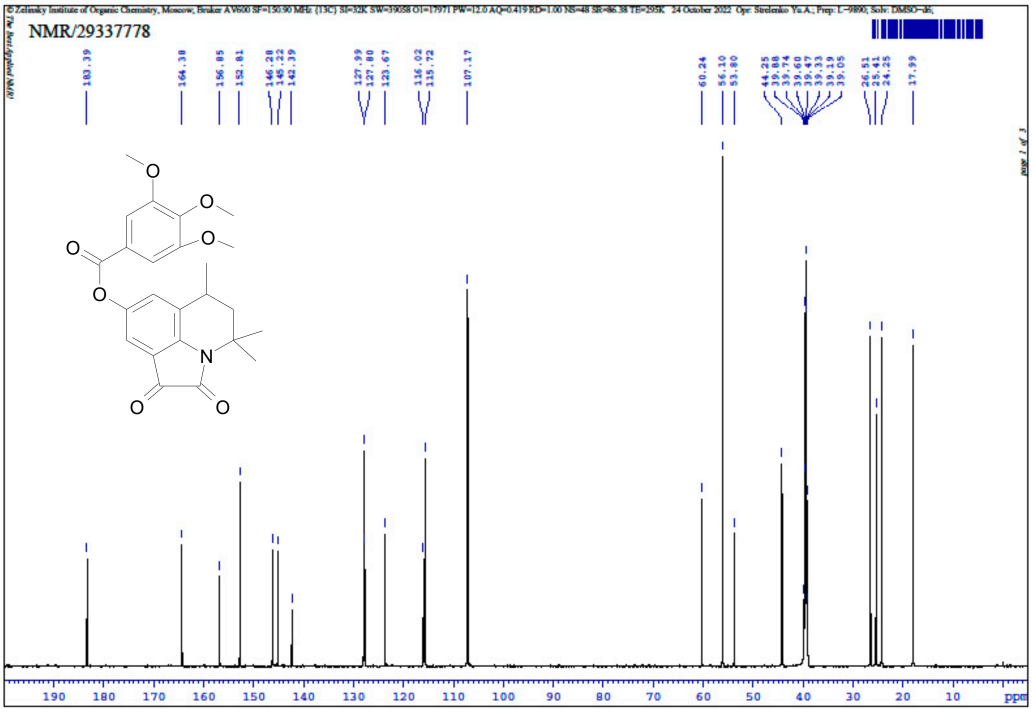

Figure S31. ^{13}C NMR spectrum of 5,6-dihydro-4*H*-pyrrolo[3,2,1-*ij*]quinoline-1,2-dione **10d**

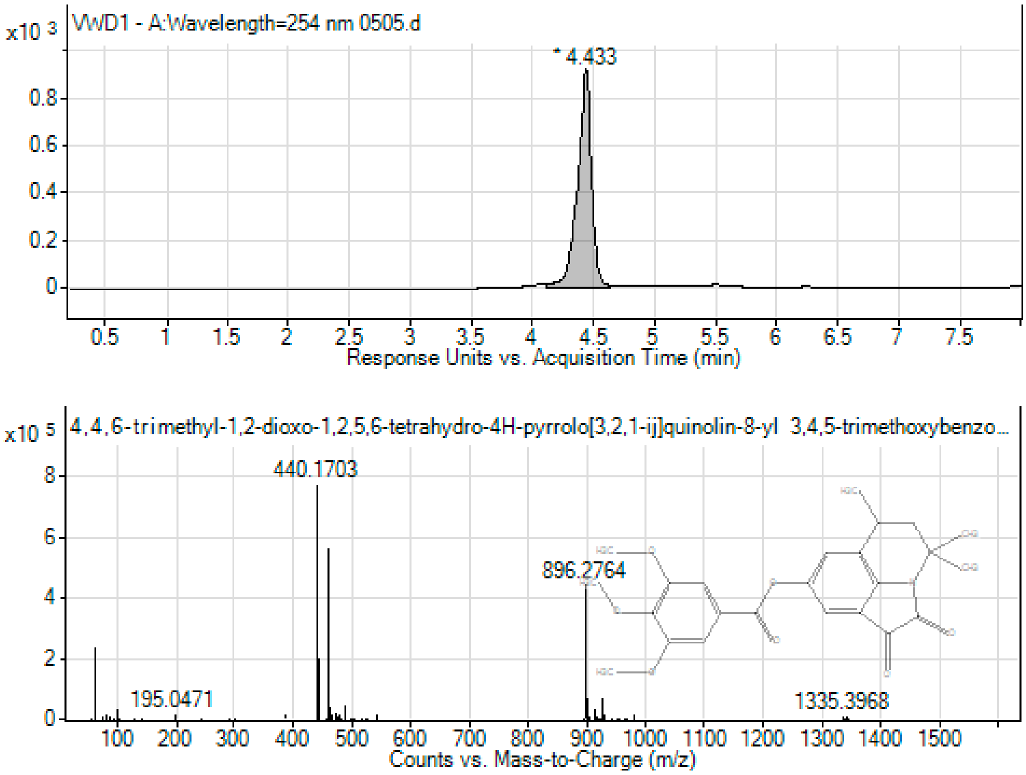

Figure S32. Data of HPLC-MS-ESI analysis of **10d**

^{1}H, ^{13}C NMR, IR and data HPLC-HRMS-ESI spectra of 8-fluoro-4,4,6-trimethyl-6-phenyl-5,6-dihydro-4*H*-pyrrolo[3,2,1-*ij*]quinoline-1,2-dione 10f

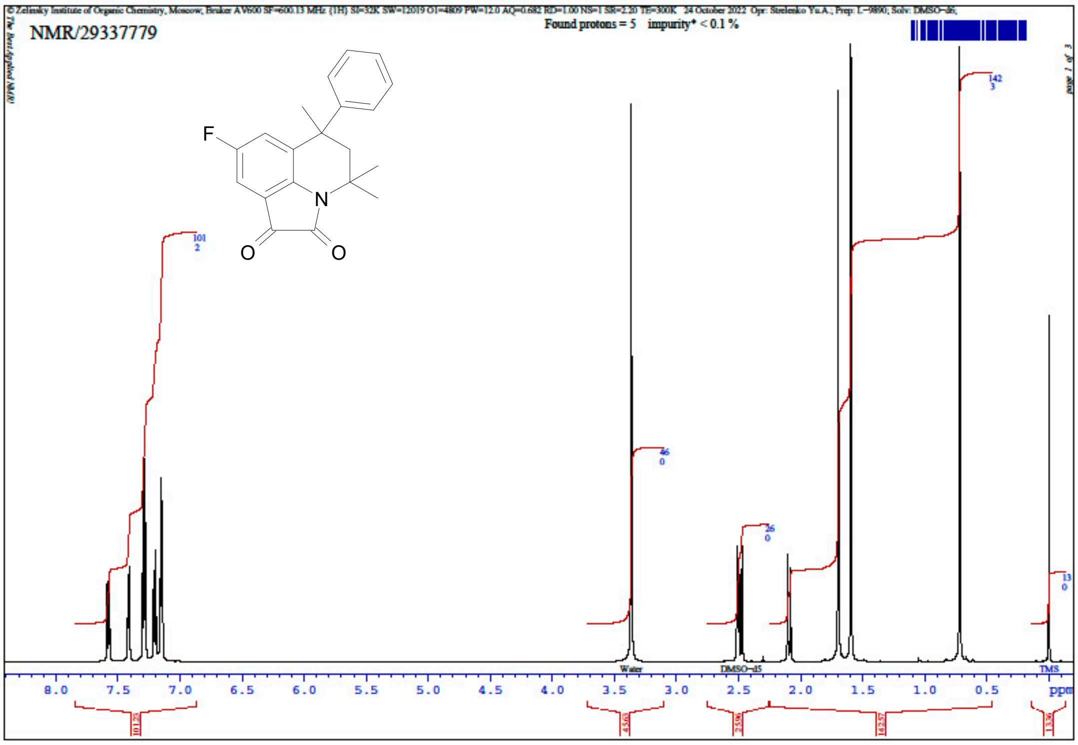

Figure S33. ^{1}H NMR spectrum of 5,6-dihydro-4*H*-pyrrolo[3,2,1-*ij*]quinoline-1,2-dione **10f**

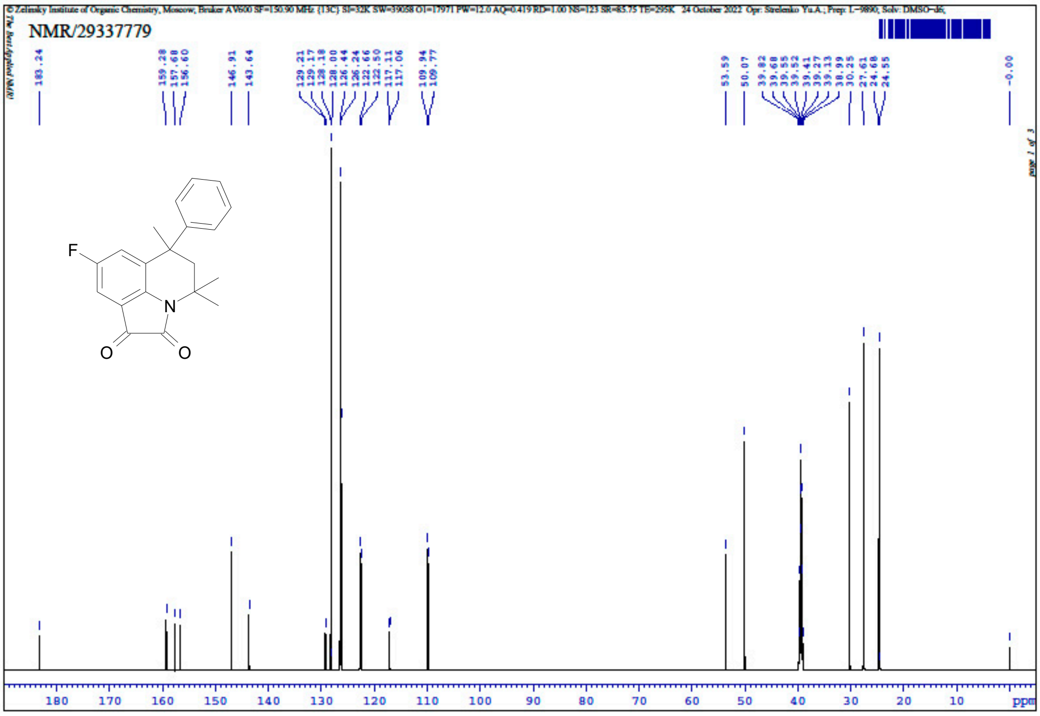

Figure S34. ^{13}C NMR spectrum of 5,6-dihydro-4*H*-pyrrolo[3,2,1-*ij*]quinoline-1,2-dione **10f**

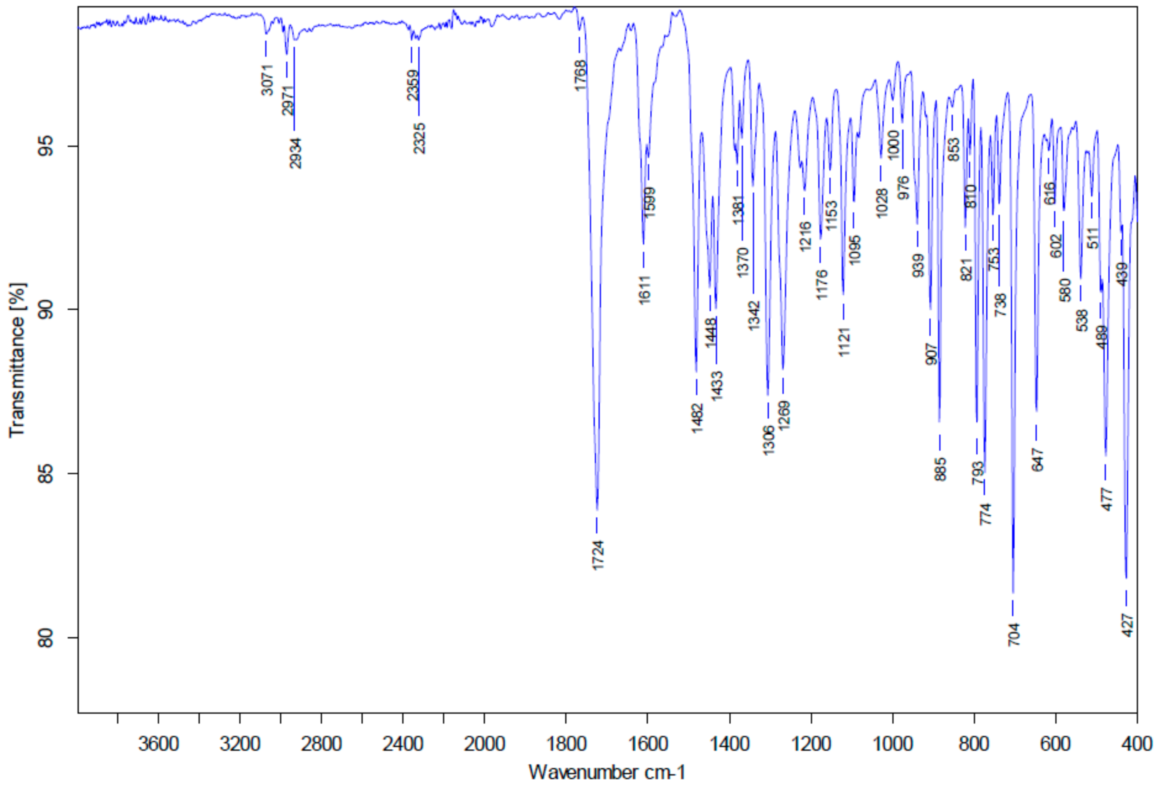

Figure S35. IR spectrum of **10f**

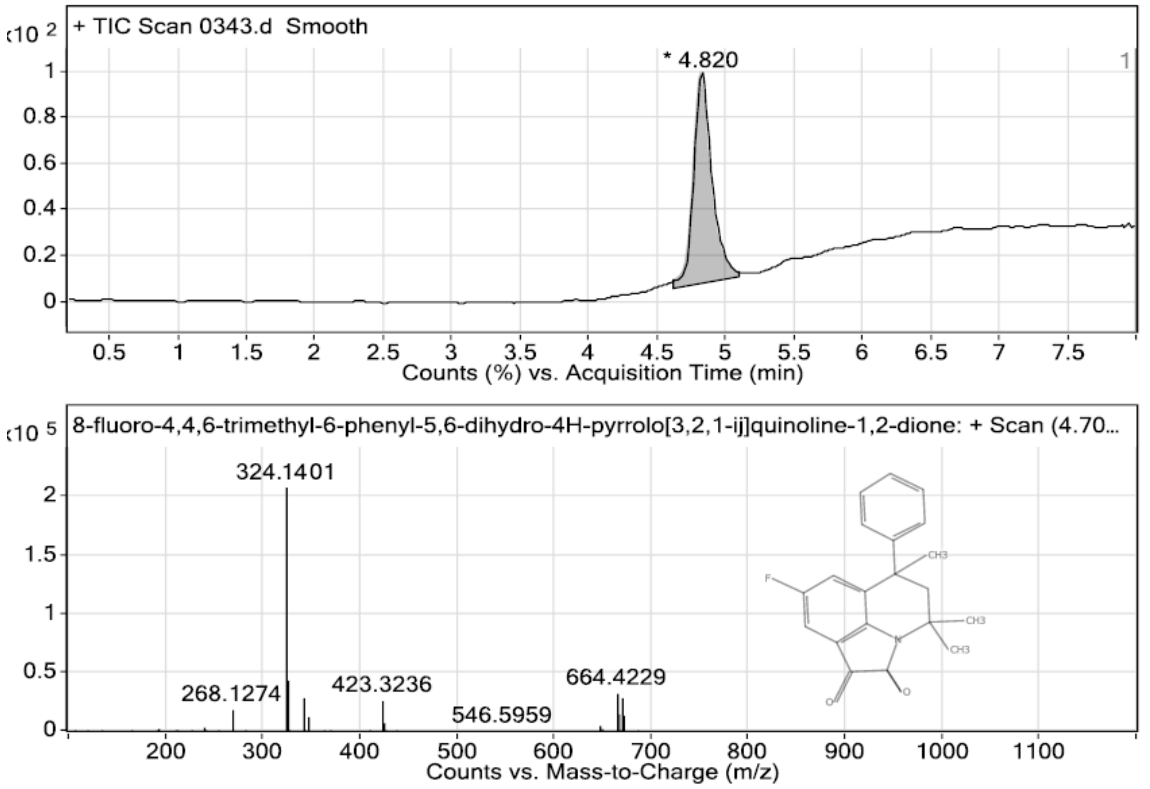

Figure S36. Data of HPLC-MS-ESI analysis of **10f**

^{1}H, ^{13}C NMR, IR and data HPLC-HRMS-ESI spectra of 6-(4-chlorophenyl)-8-fluoro-4,4,6-trimethyl-5,6-dihydro-4*H*-pyrrolo[3,2,1-*ij*]quinoline-1,2-dione 10g

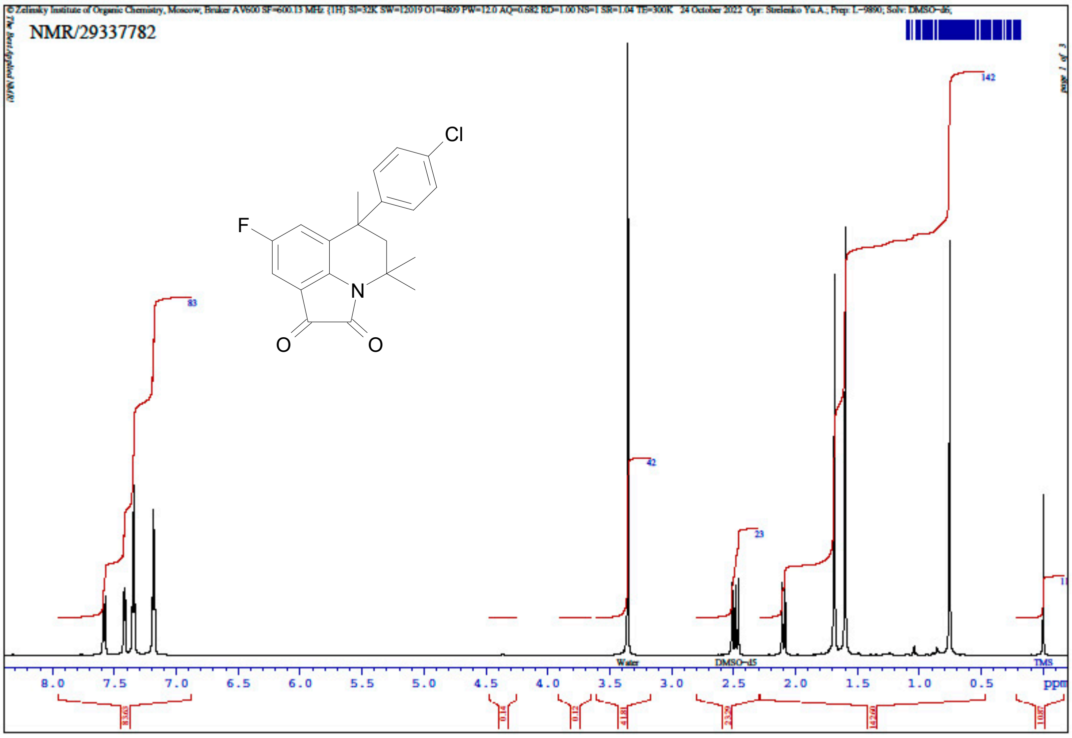

Figure S37. ^{1}H NMR spectrum of 5,6-dihydro-4*H*-pyrrolo[3,2,1-*ij*]quinoline-1,2-dione **10g**

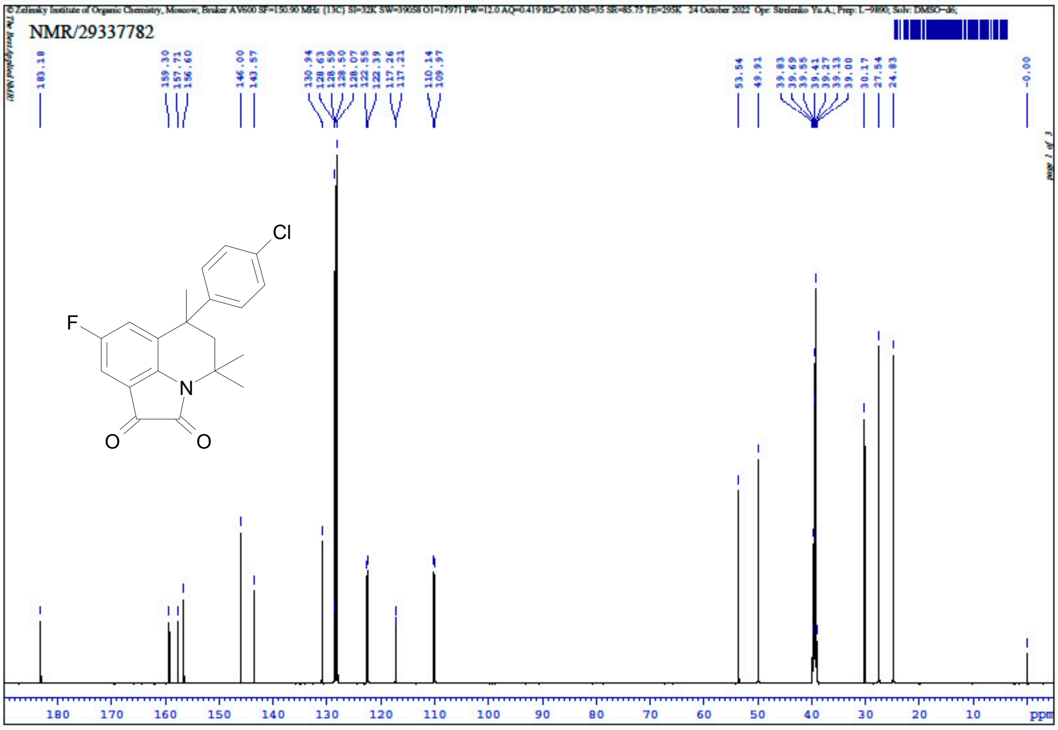

Figure S38. ^{13}C NMR spectrum of 5,6-dihydro-4*H*-pyrrolo[3,2,1-*ij*]quinoline-1,2-dione **10g**

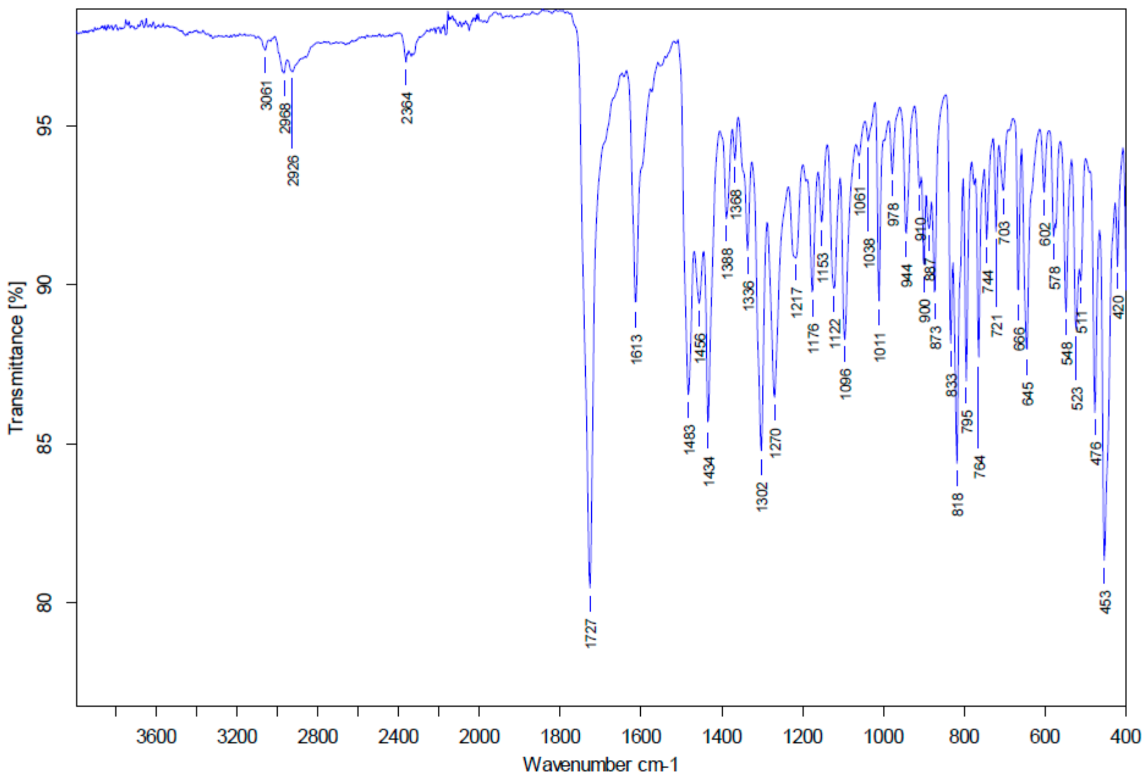

Figure S39. IR spectrum of **10g**

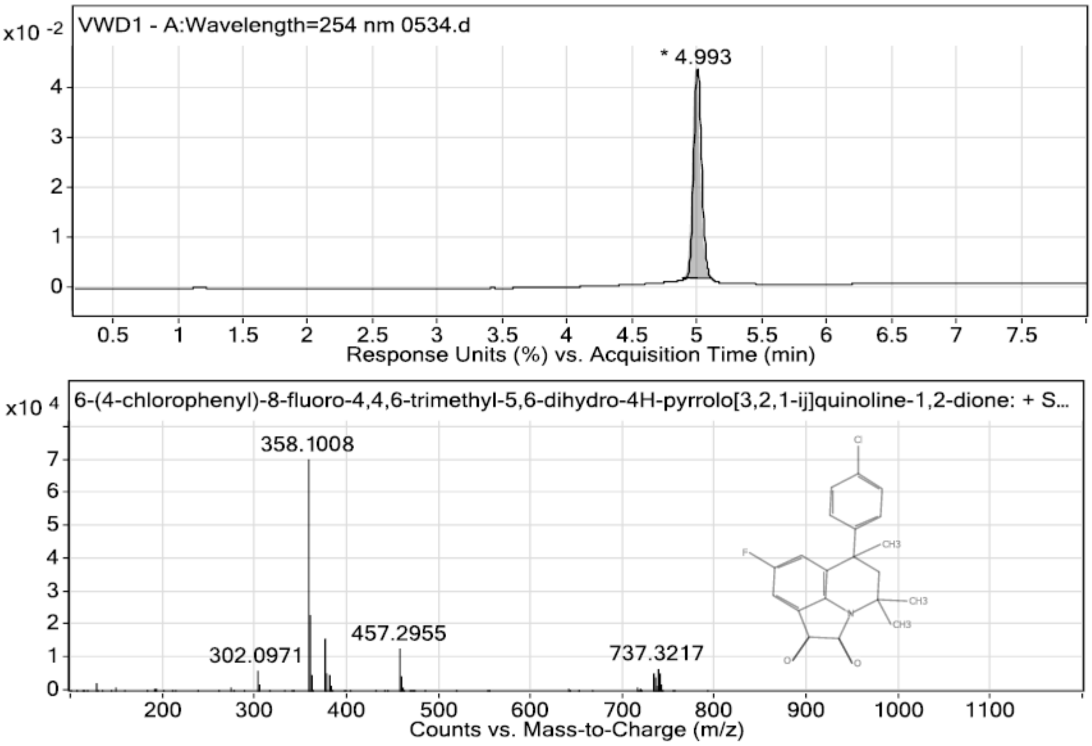

Figure S40. Data of HPLC-MS-ESI analysis of **10g**

^{1}H, ^{13}C NMR and data HPLC-HRMS-ESI spectra of 6-(4-chlorophenyl)-4,4,6,8-tetramethyl-5,6-dihydro-4*H*-pyrrolo[3,2,1-*ij*]quinoline-1,2-dione 10l

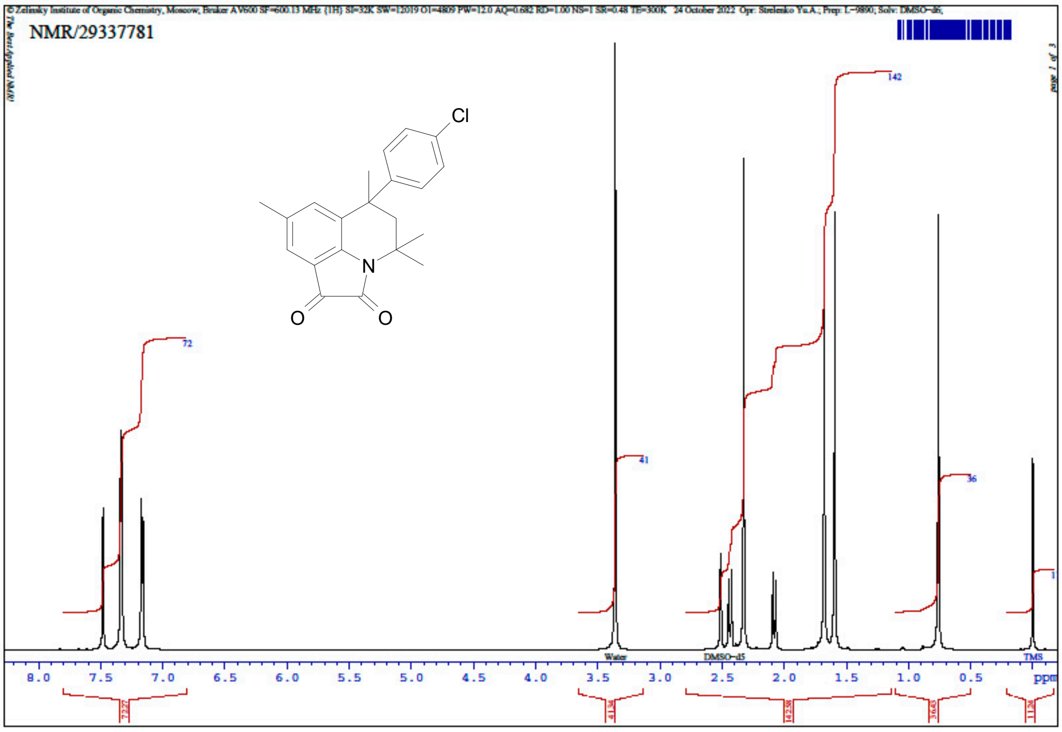

Figure S41. ^{1}H NMR spectrum of 5,6-dihydro-4*H*-pyrrolo[3,2,1-*ij*]quinoline-1,2-dione **10l**

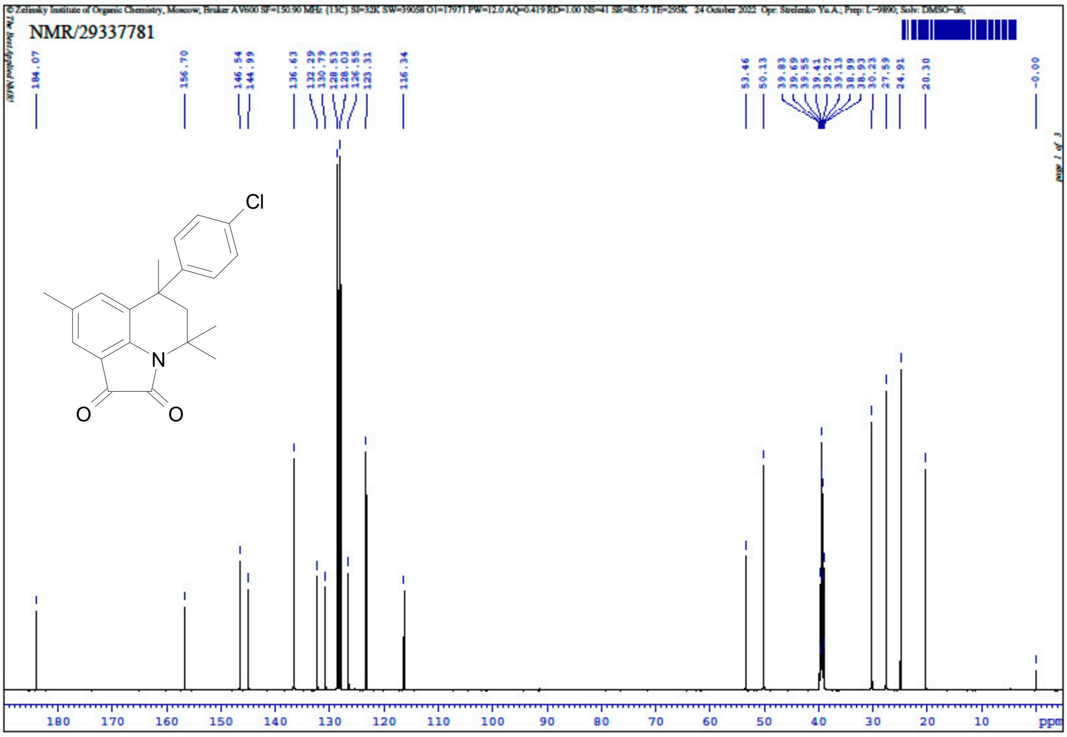

Figure S42. ^{13}C NMR spectrum of 5,6-dihydro-4*H*-pyrrolo[3,2,1-*ij*]quinoline-1,2-dione **10l**

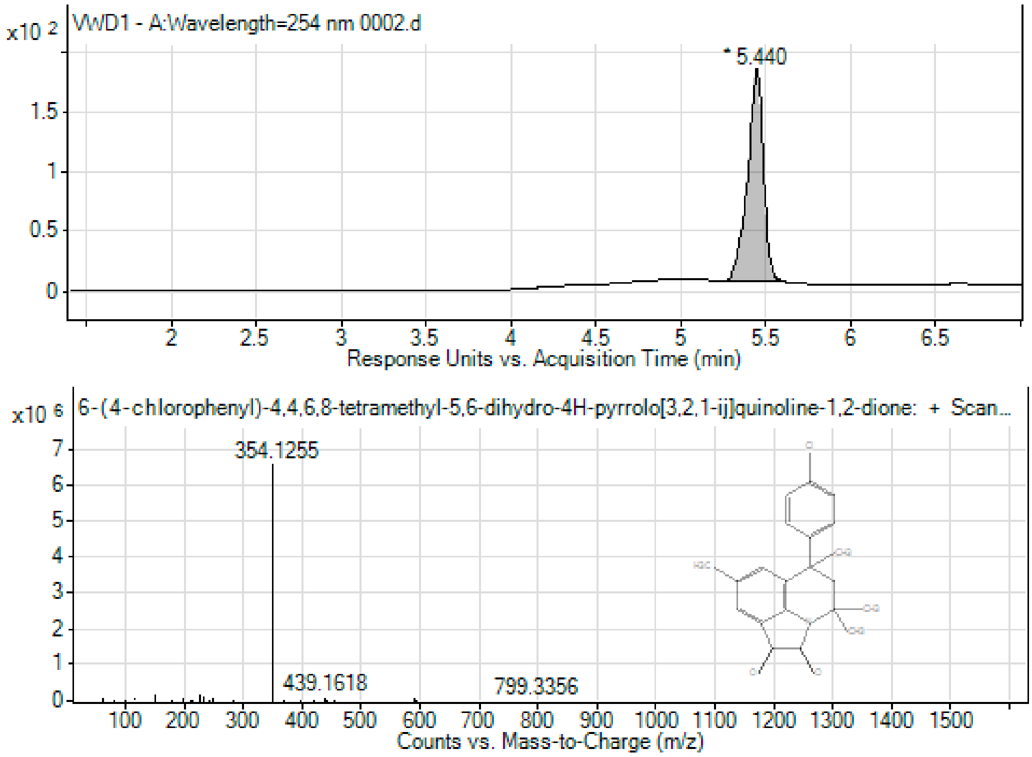

Figure S43. Data of HPLC-MS-ESI analysis of **10l**

^{1}H, ^{13}C NMR and data HPLC-HRMS-ESI spectra of 8-ethoxy-4,4,6-trimethyl-5,6-dihydro-4*H*-pyrrolo[3,2,1-*ij*]quinoline-1,2-dione 10m

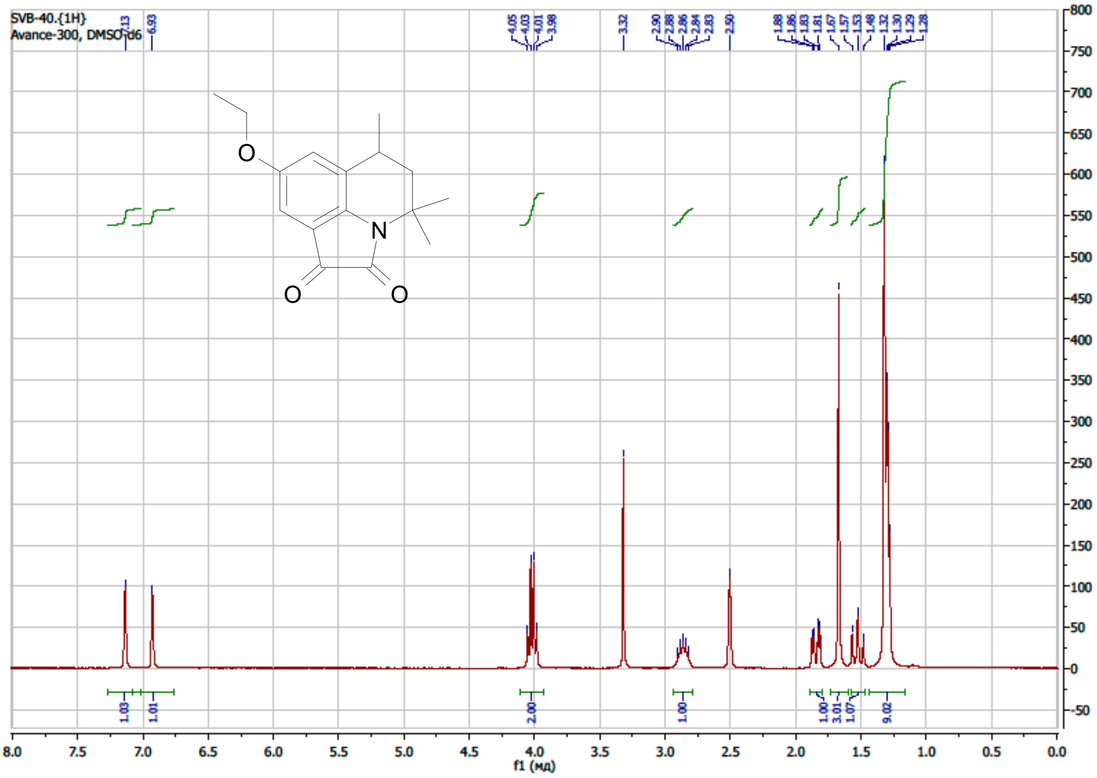

Figure S44. ^{1}H NMR spectrum of 5,6-dihydro-4*H*-pyrrolo[3,2,1-*ij*]quinoline-1,2-dione **10m**

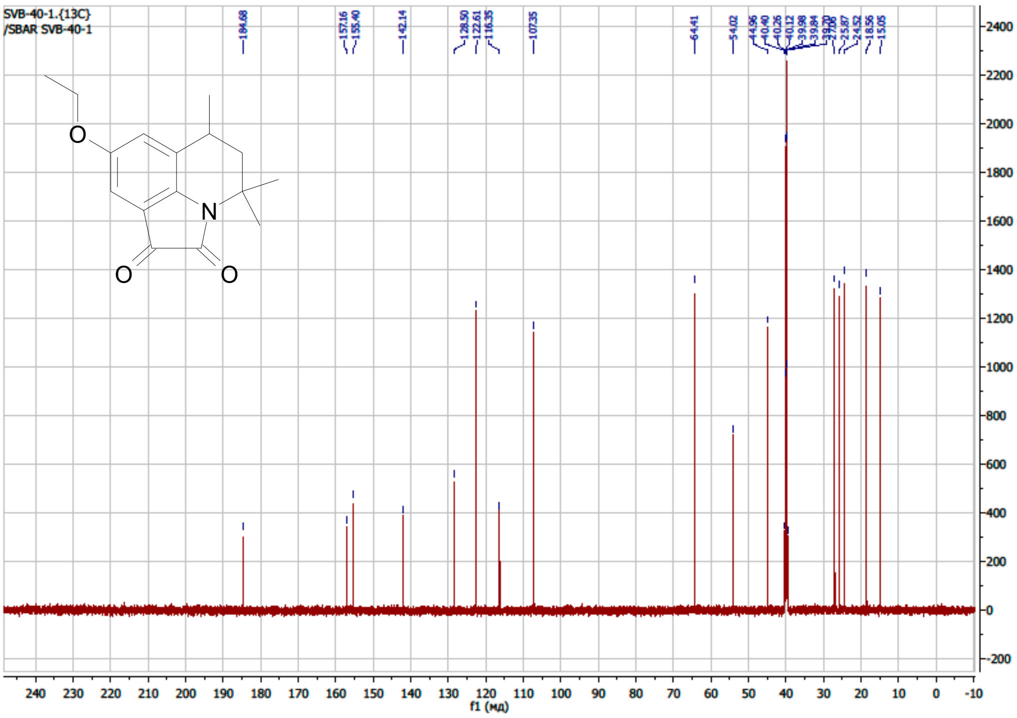

Figure S45. ^{13}C NMR spectrum of 5,6-dihydro-4*H*-pyrrolo[3,2,1-*ij*]quinoline-1,2-dione **10m**

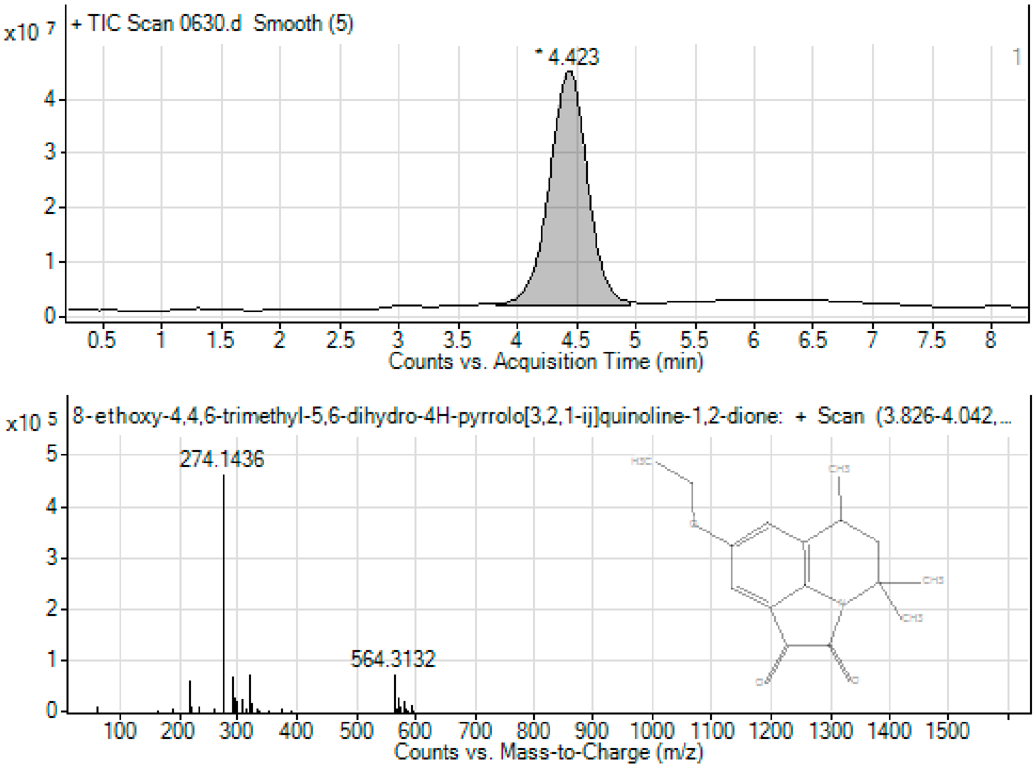

Figure S46. Data of HPLC-MS-ESI analysis of **10m**

^{1}H, ^{13}C NMR and data HPLC-HRMS-ESI spectra of 6-(4-chlorophenyl)-4,4,6-trimethyl-5,6-dihydro-4*H*-pyrrolo[3,2,1-*ij*]quinoline-1,2-dione 10o

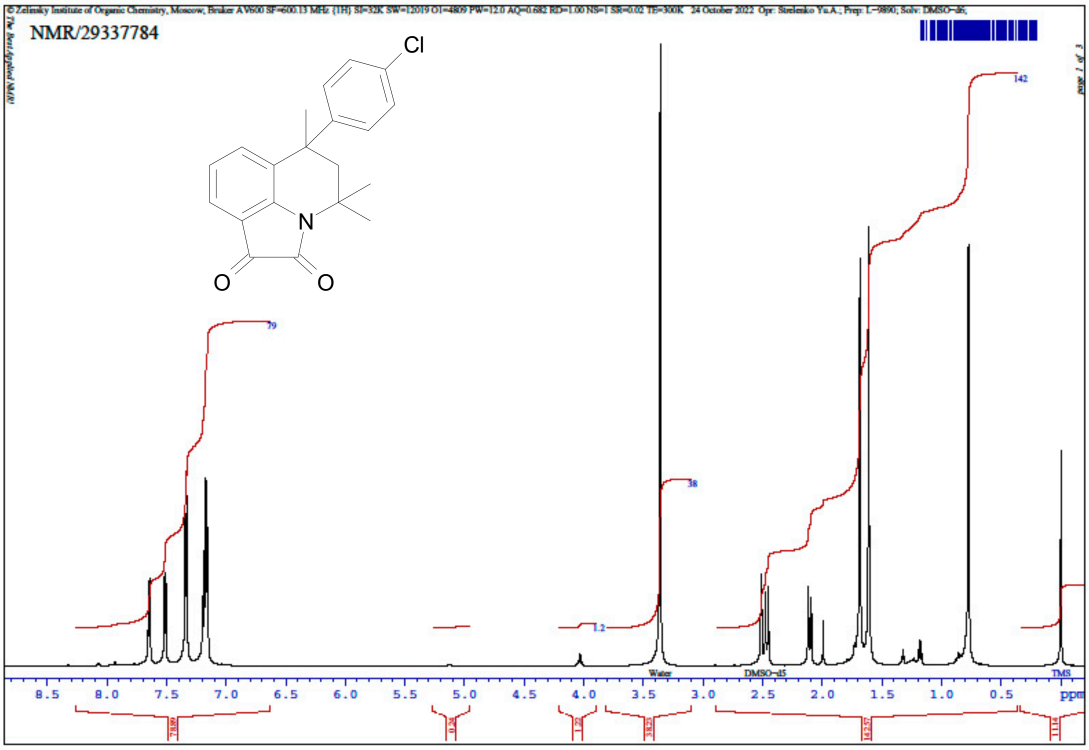

Figure S47. ^{1}H NMR spectrum of 5,6-dihydro-4*H*-pyrrolo[3,2,1-*ij*]quinoline-1,2-dione **10o**

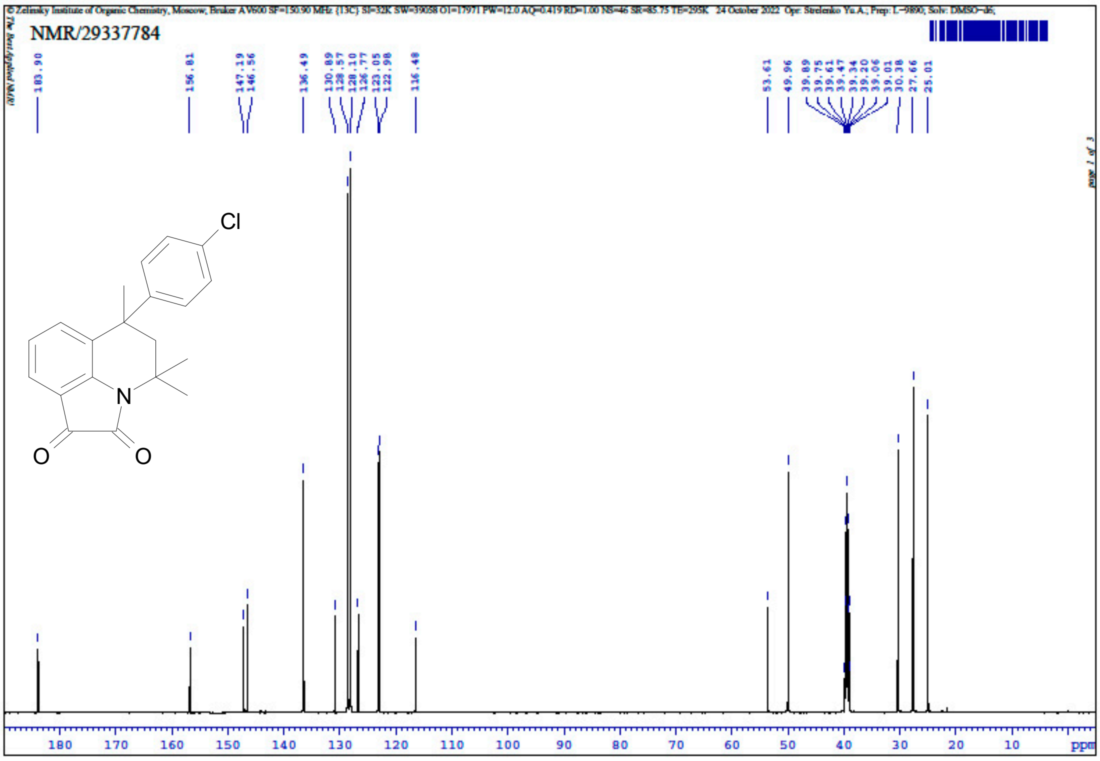

Figure S48. ^{13}C NMR spectrum of 5,6-dihydro-4*H*-pyrrolo[3,2,1-*ij*]quinoline-1,2-dione **10o**

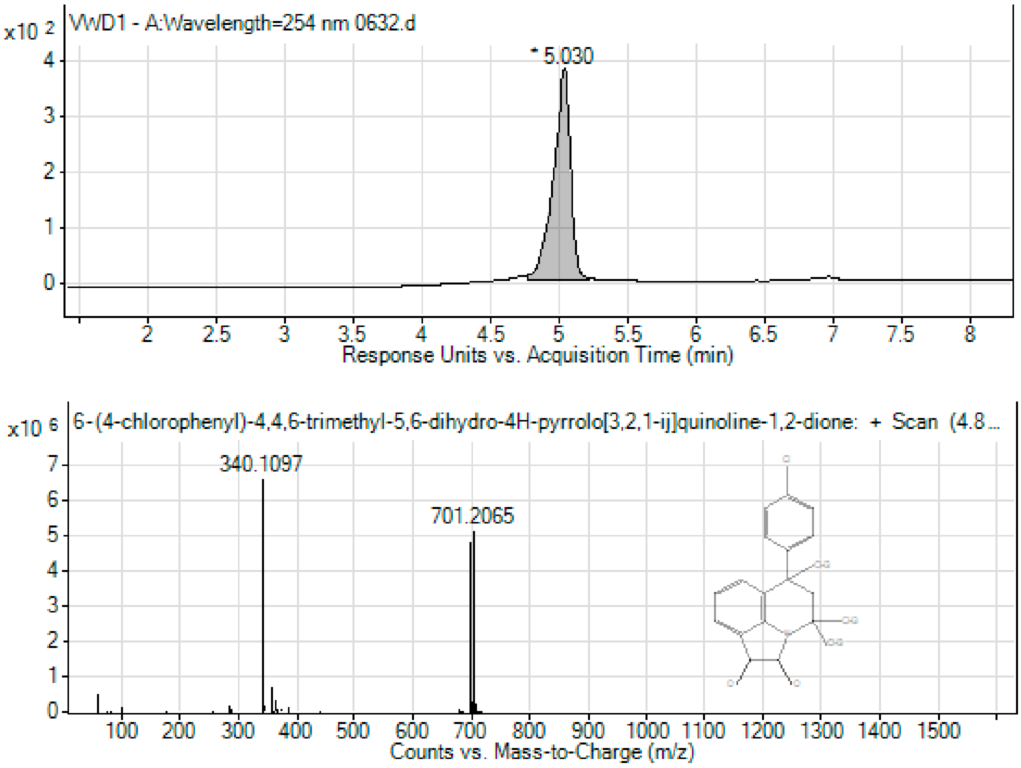

Figure S49. Data of HPLC-MS-ESI analysis of **10o**

^{1}H, ^{13}C NMR and data HPLC-HRMS-ESI spectra of (*Z*)-2-thioxo-5-(4,4,6-trimethyl-2-oxo-5,6-dihydro-4*H*-pyrrolo[3,2,1-*ij*]quinolin-1(2*H*)-ylidene)thiazolidin-4-one 12a

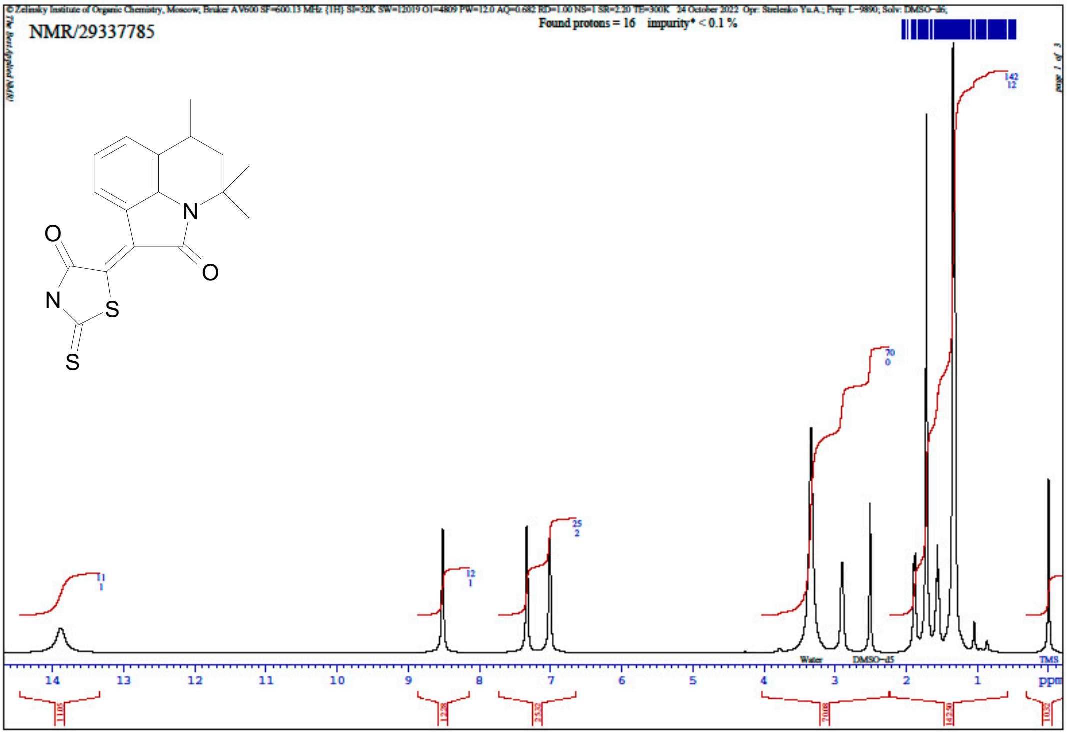

Figure S50. ^{1}H NMR spectrum of compound **12a**

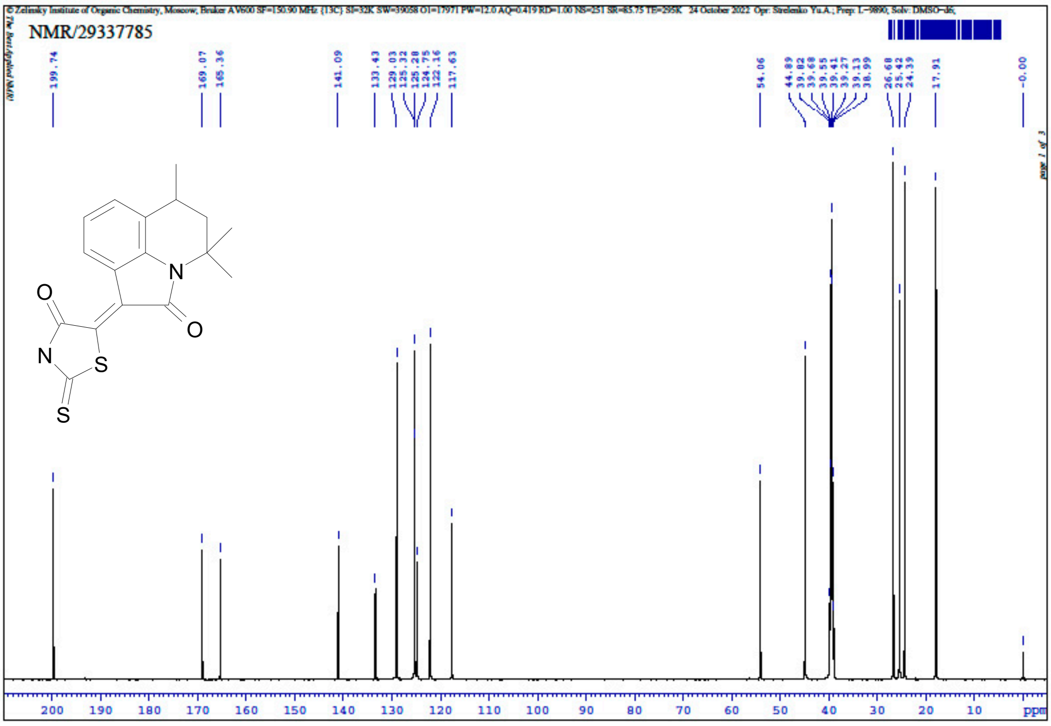

Figure S51. ^{13}C NMR spectrum of compound **12a**

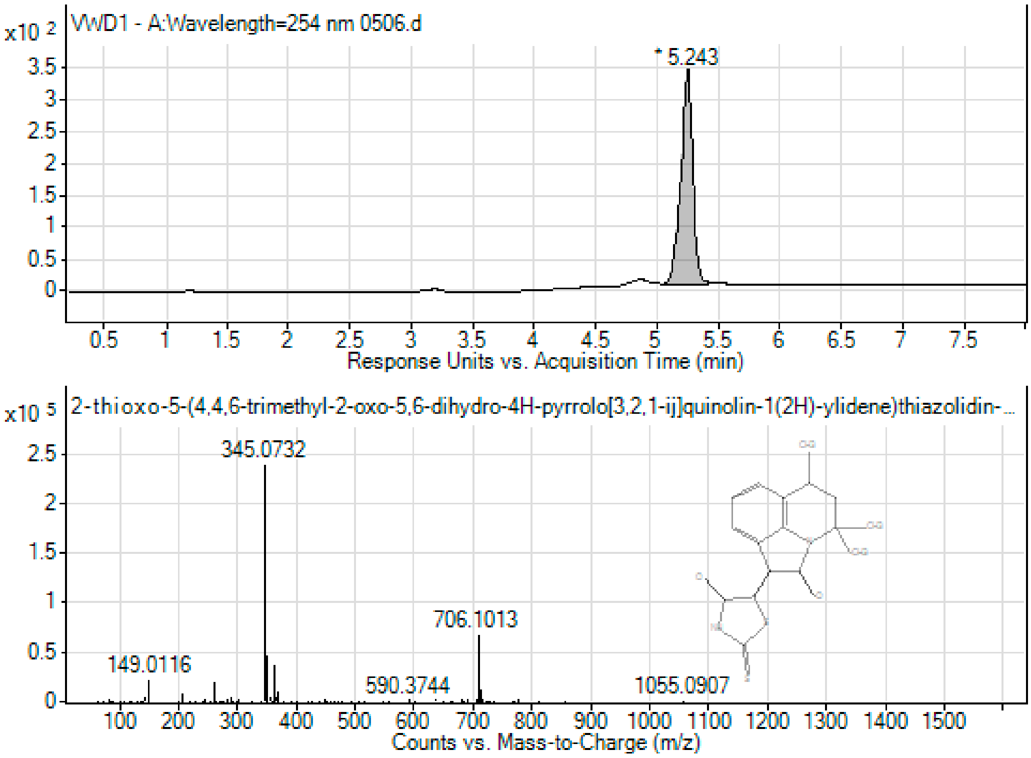

Figure S52. Data of HPLC-MS-ESI analysis of compound **12a**

^{1}H, ^{13}C NMR and data HPLC-HRMS-ESI spectra of (*Z*)-5-(8-iodo-4,4,6-trimethyl-2-oxo-5,6-dihydro-4*H*-pyrrolo[3,2,1-*ij*]quinolin-1(2*H*)-ylidene)-2-thioxothiazolidin-4-one 12b

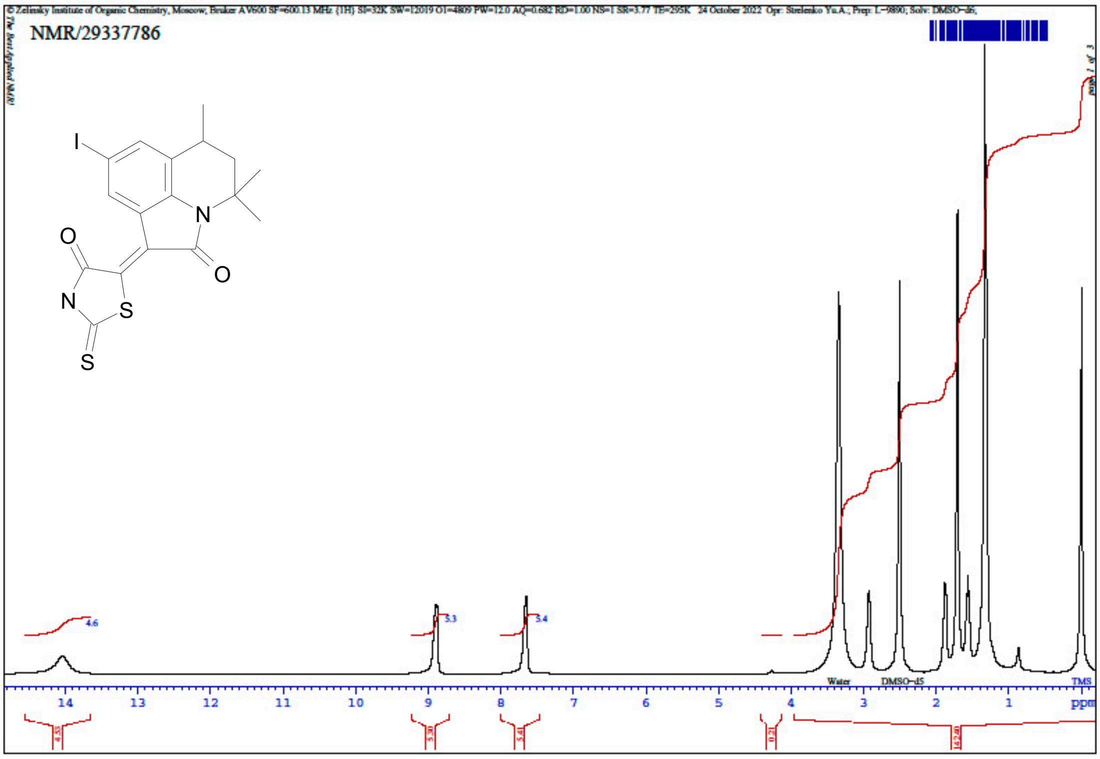

Figure S53. ^{1}H NMR spectrum of compound **12b**

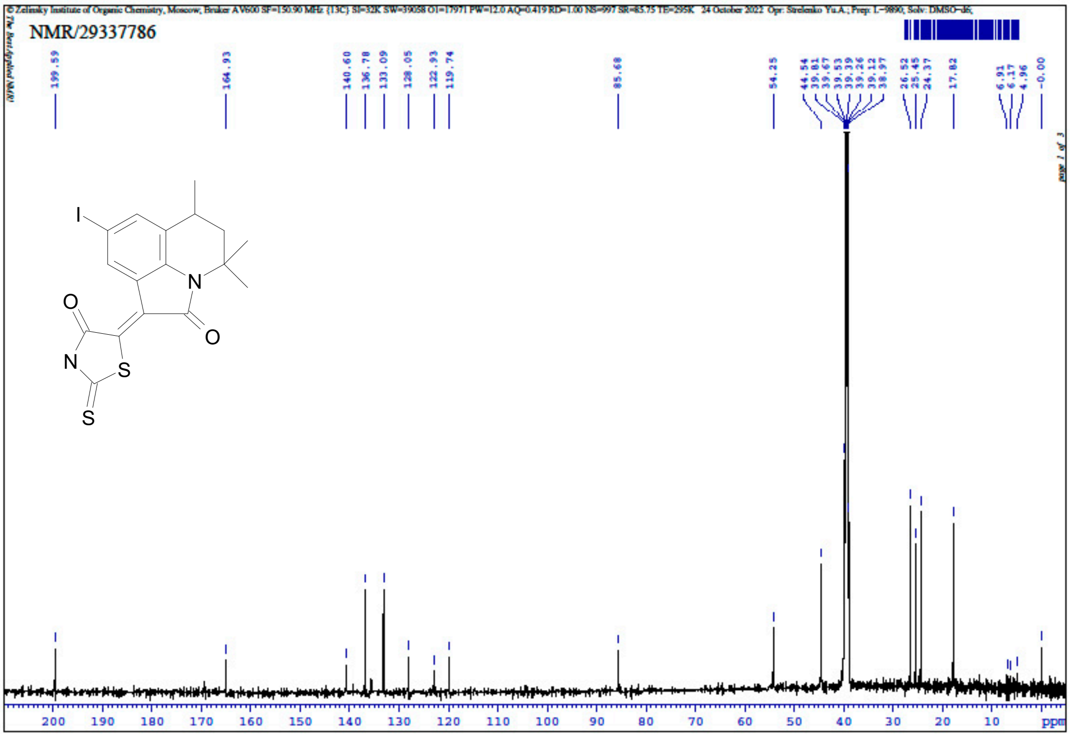

Figure S54. ^{13}C NMR spectrum of compound **12b**

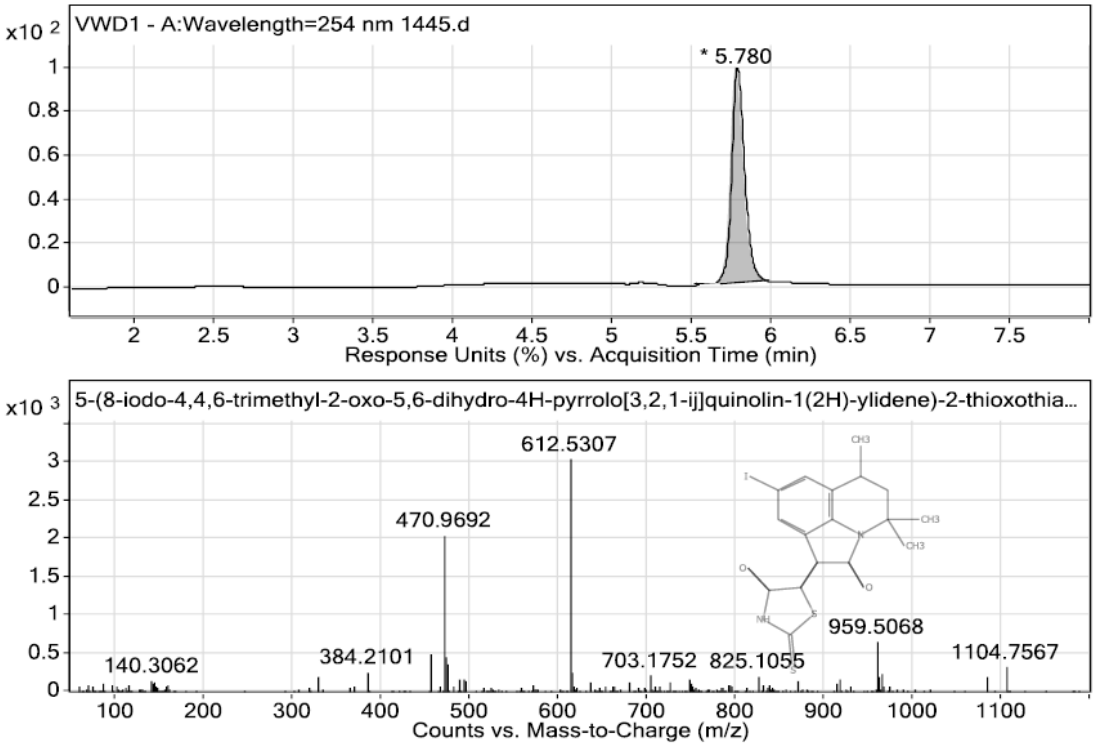

Figure S55. Data of HPLC-MS-ESI analysis of compound **12b**

^{1}H, ^{13}C NMR and data HPLC-HRMS-ESI spectra of (*Z*)-4,4,6-trimethyl-2-oxo-1-(4-oxo-2-thioxothiazolidin-5-ylidene)-1,2,5,6-tetrahydro-4*H*-pyrrolo[3,2,1-*ij*]quinolin-8-yl benzoate 12c

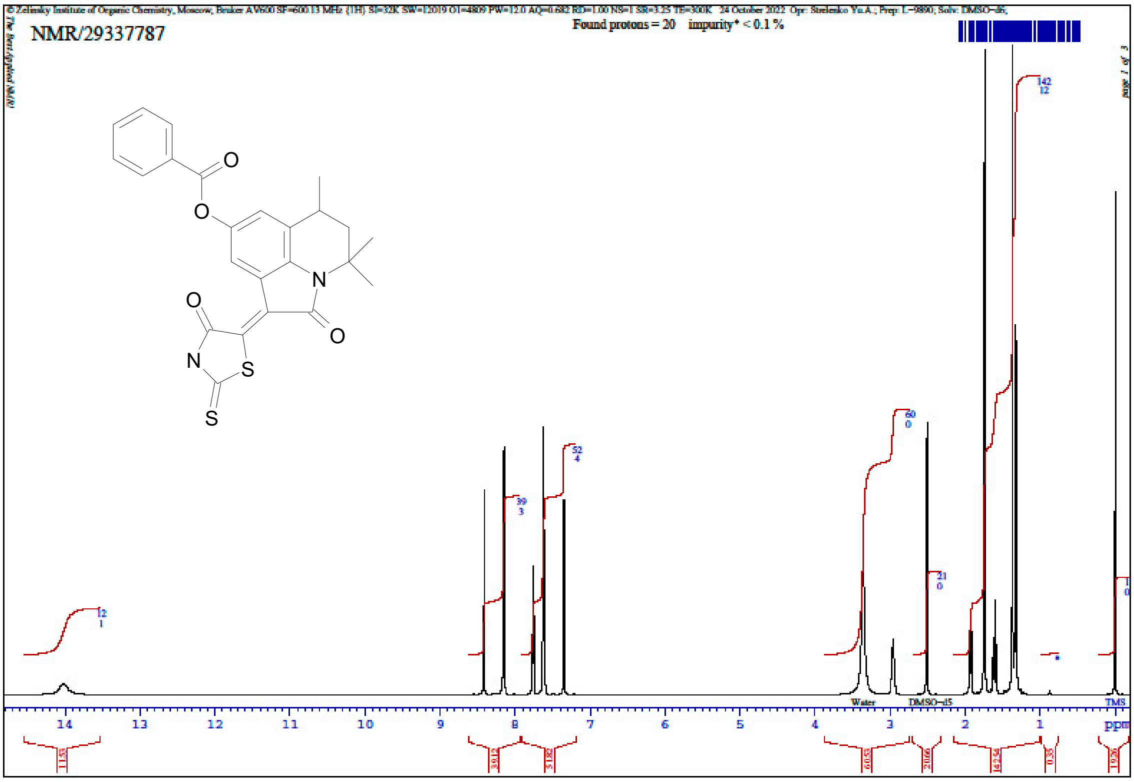

Figure S56. ^{1}H NMR spectrum of compound **12c**

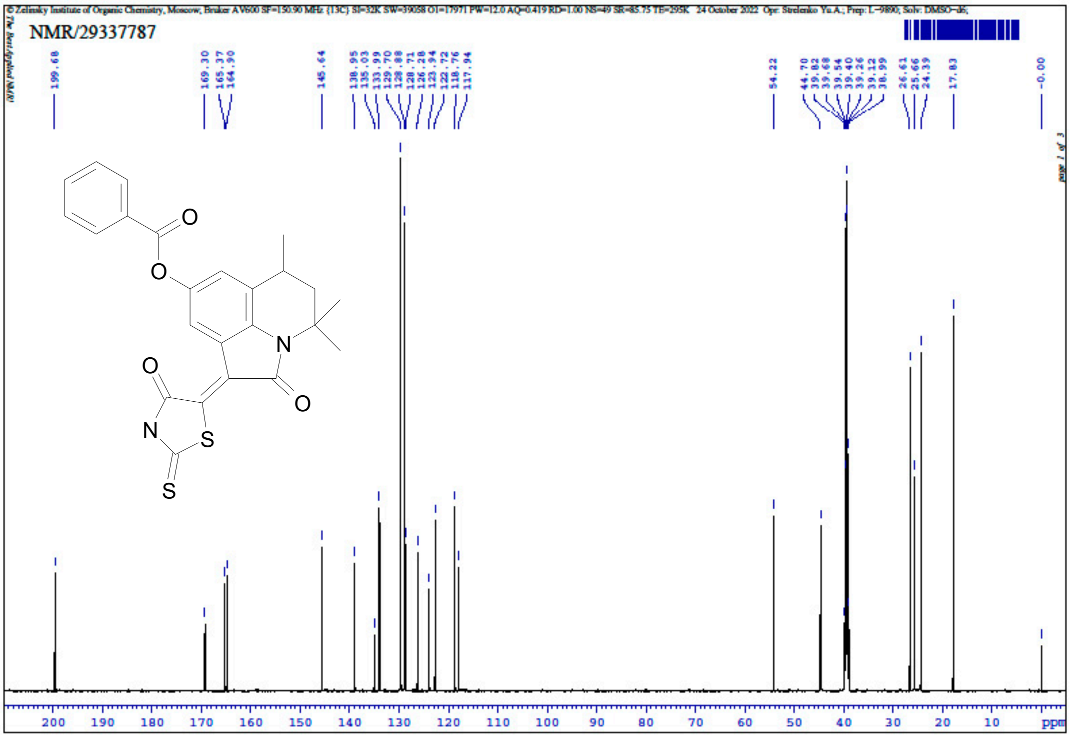

Figure S57. ^{13}C NMR spectrum of compound **12c**

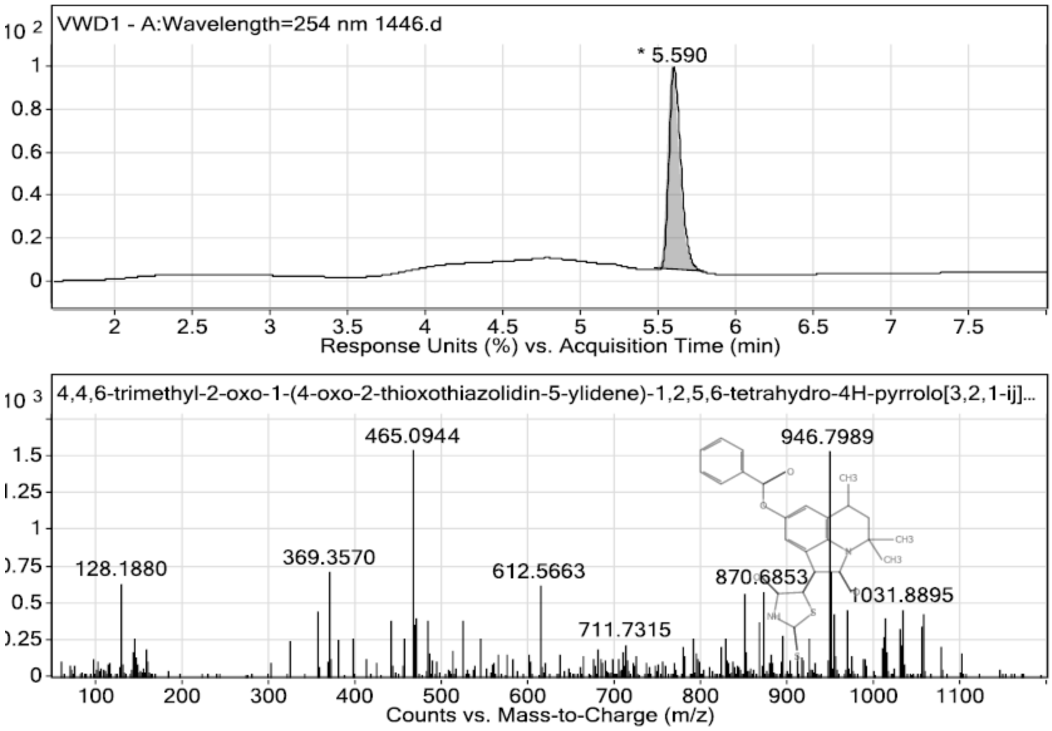

Figure S58. Data of HPLC-MS-ESI analysis of compound **12c**

¹H, ¹³C NMR and data HPLC-HRMS-ESI spectra of (*Z*)-4,4,6-trimethyl-2-oxo-1-(4-oxo-2-thioxothiazolidin-5-ylidene)-1,2,5,6-tetrahydro-4*H*-pyrrolo[3,2,1-*ij*]quinolin-8-yl 2-methoxybenzoate 12d

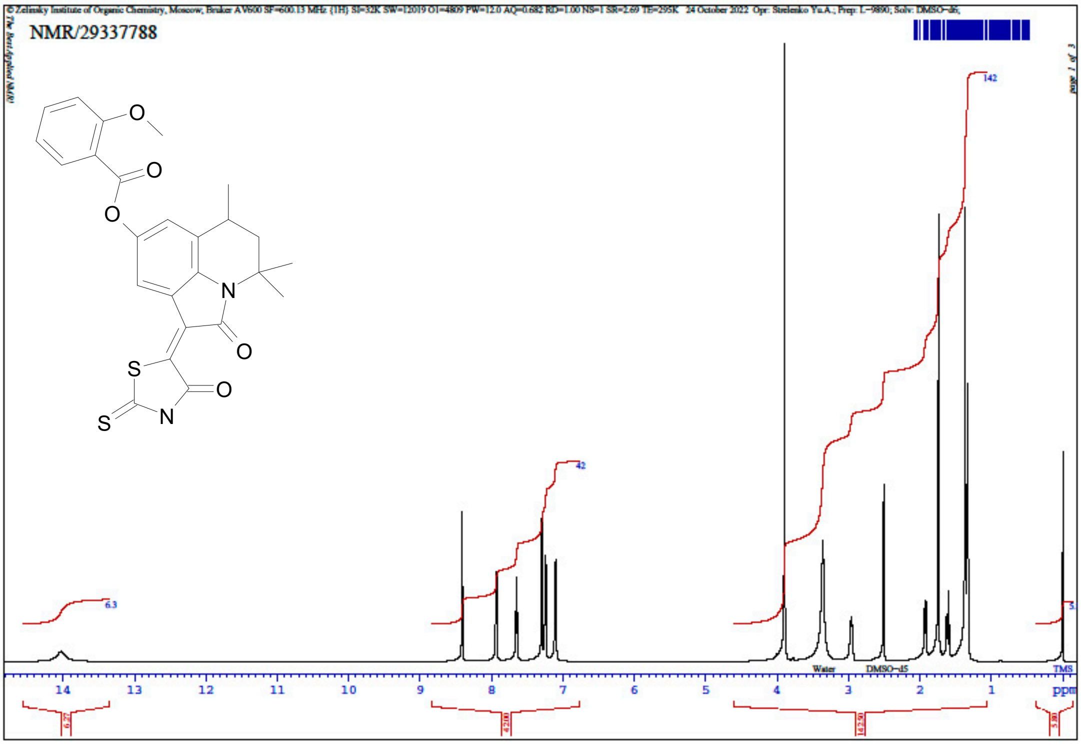

Figure S59. ^{1}H NMR spectrum of compound **12d**

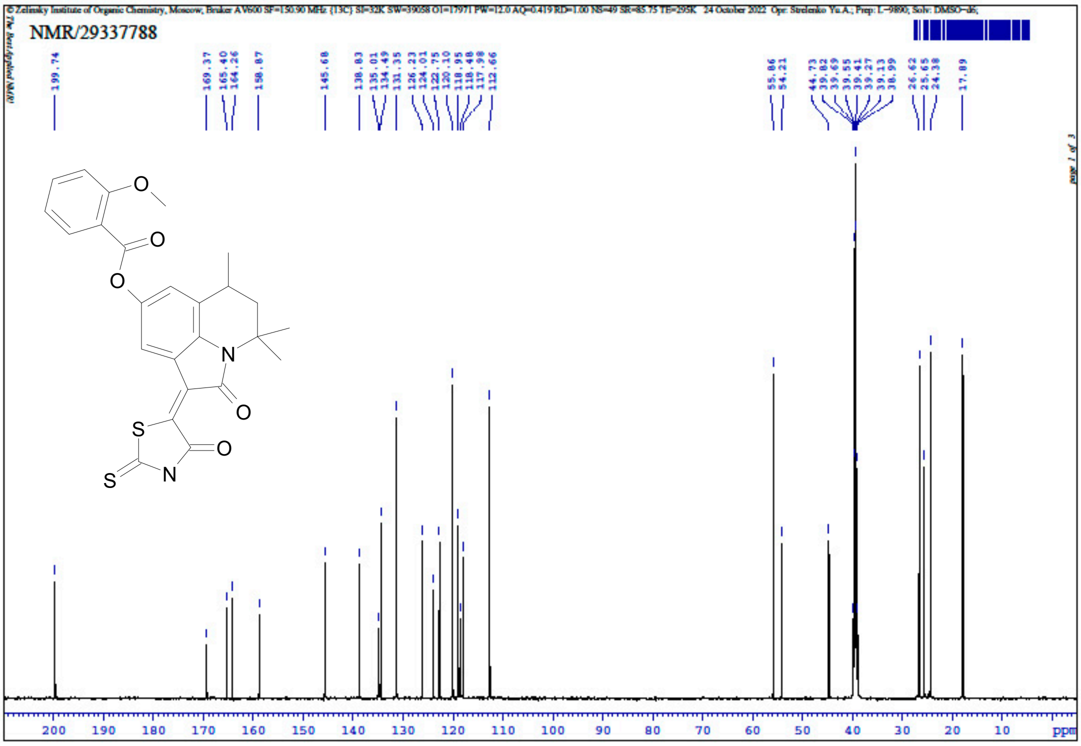

Figure S60. ^{13}C NMR spectrum of compound **12d**

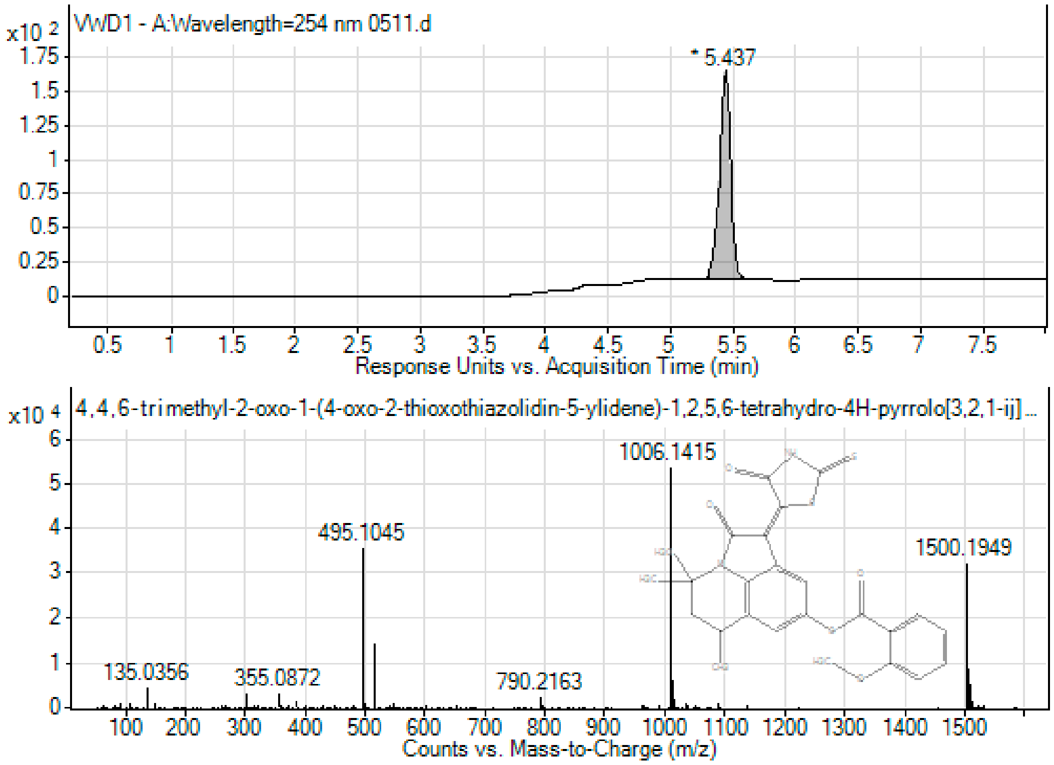

Figure S61. Data of HPLC-MS-ESI analysis of compound **12d**

¹H, ¹³C NMR, IR and data HPLC-HRMS-ESI spectra of (*Z*)-4,4,6-trimethyl-2-oxo-1-(4-oxo-2-thioxothiazolidin-5-ylidene)-1,2,5,6-tetrahydro-4*H*-pyrrolo[3,2,1-*ij*]quinolin-8-yl 3,4,5-trimethoxybenzoate 12e

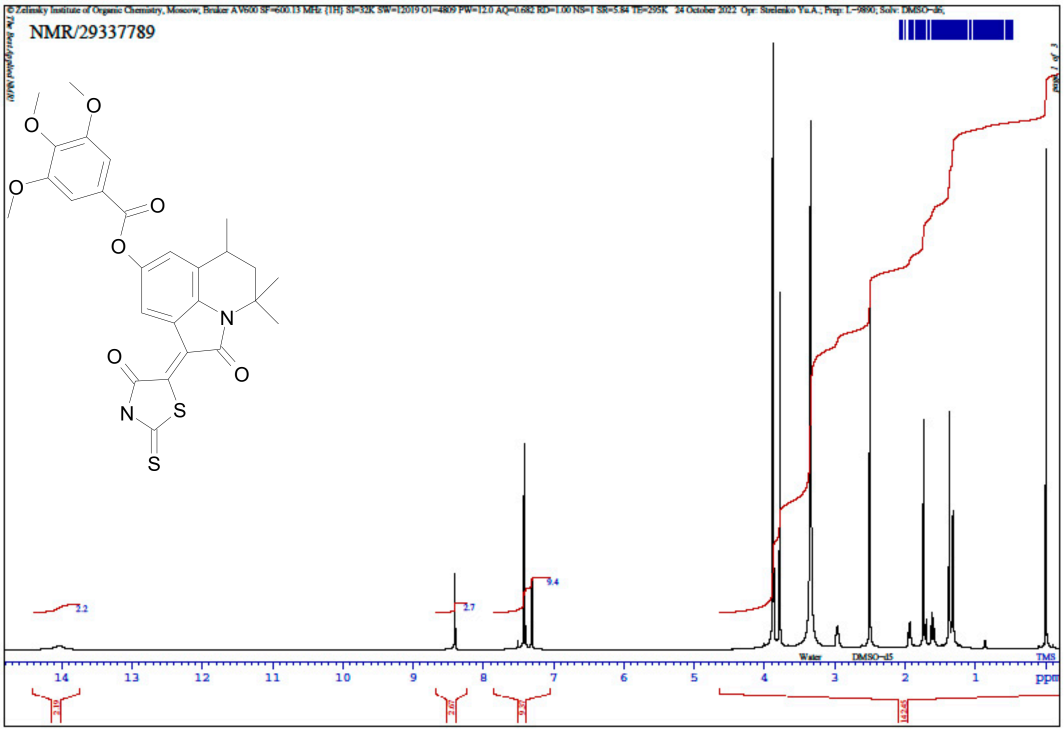

Figure S62. ¹H NMR spectrum of compound **12e**

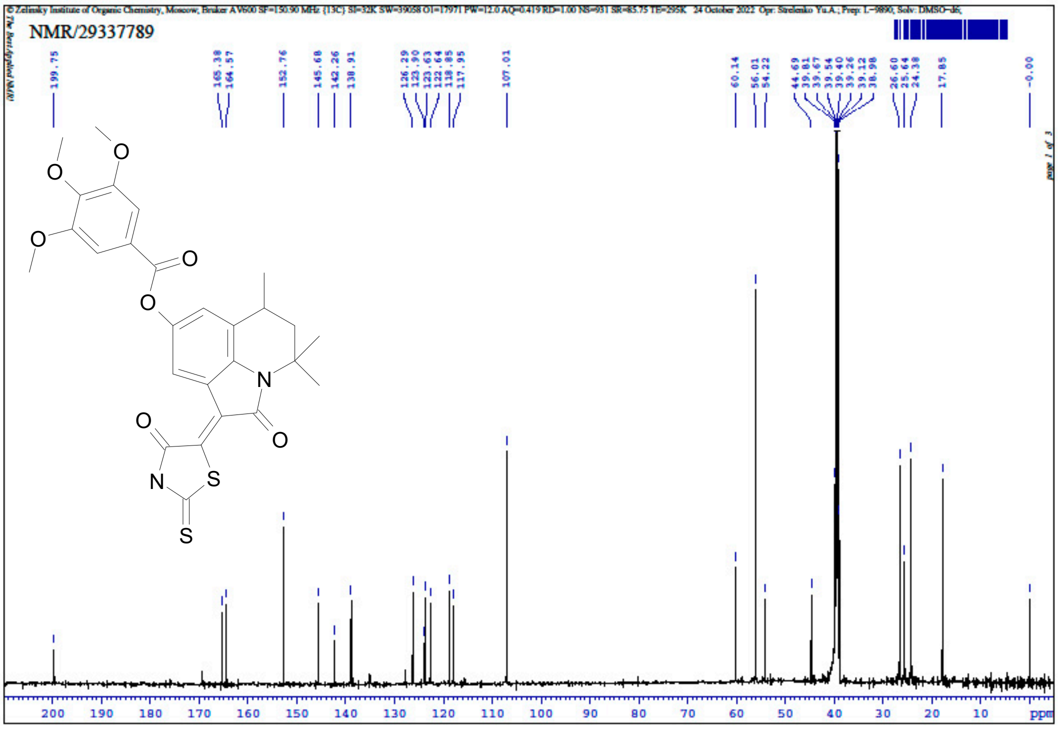

Figure S63. ^{13}C NMR spectrum of compound **12e**

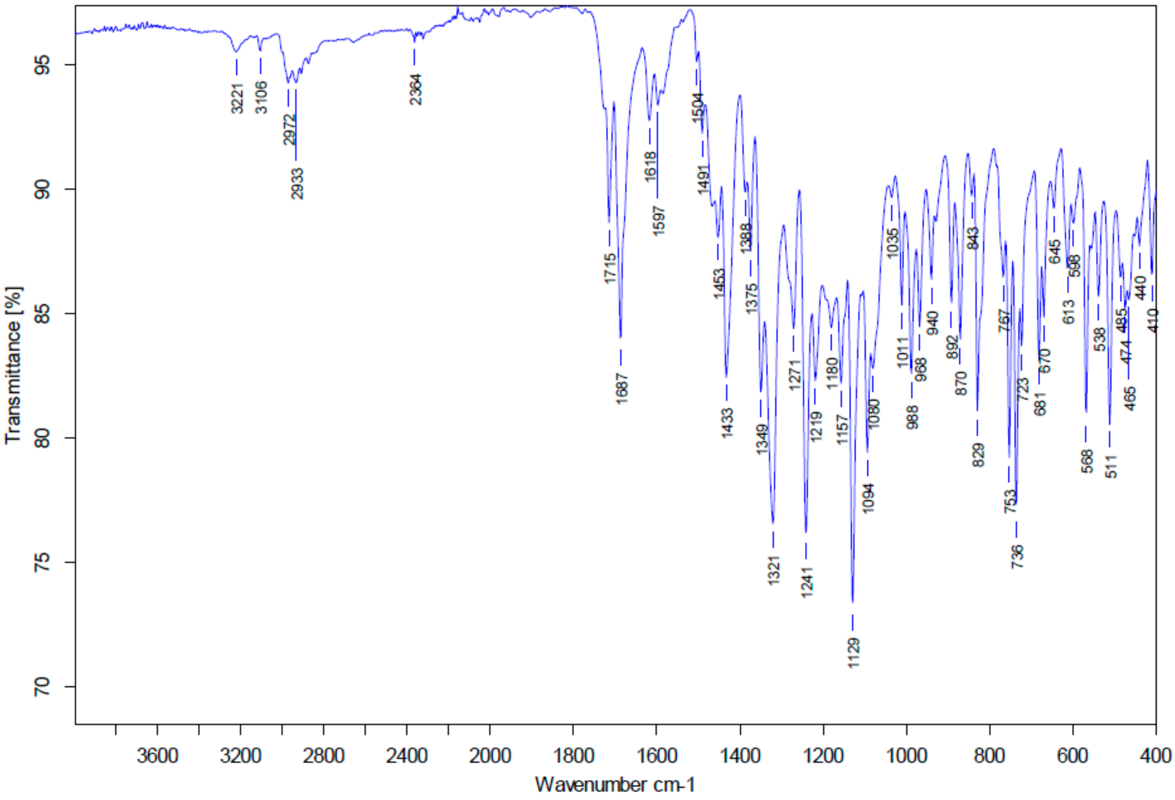

Figure S64. IR spectrum of compound **12e**

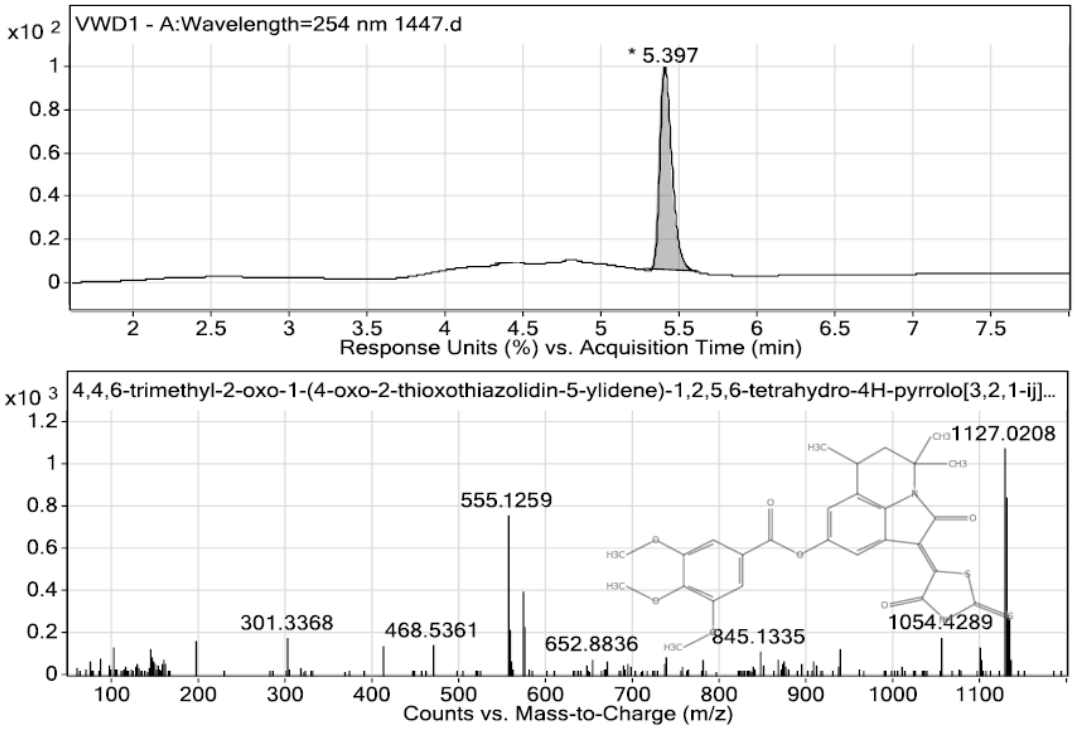

Figure S65. Data of HPLC-MS-ESI analysis of compound **12e**

^{1}H, ^{13}C NMR and data HPLC-HRMS-ESI spectra of (*Z*)-3-ethyl-5-(8-methoxy-4,4,6-trimethyl-2-oxo-5,6-dihydro-4*H*-pyrrolo[3,2,1-*ij*]quinolin-1(2*H*)-ylidene)-2-thioxothiazolidin-4-one 12f

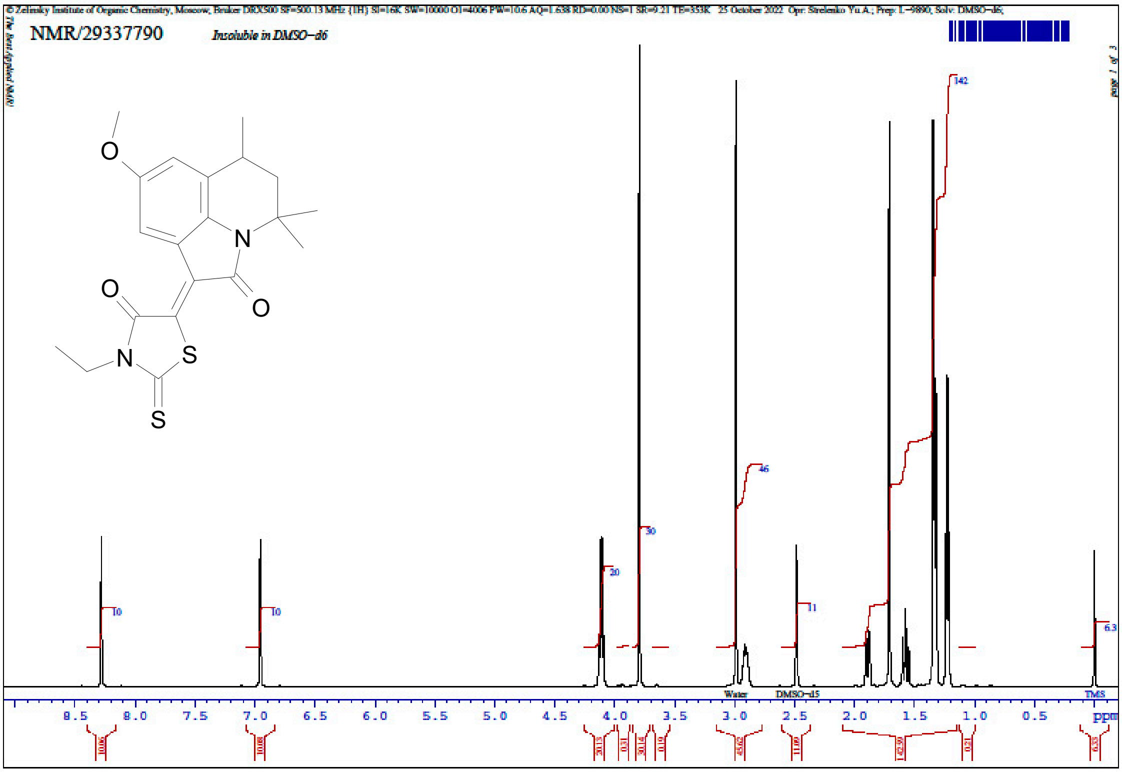

Figure S66. ^{1}H NMR spectrum of compound **12f**

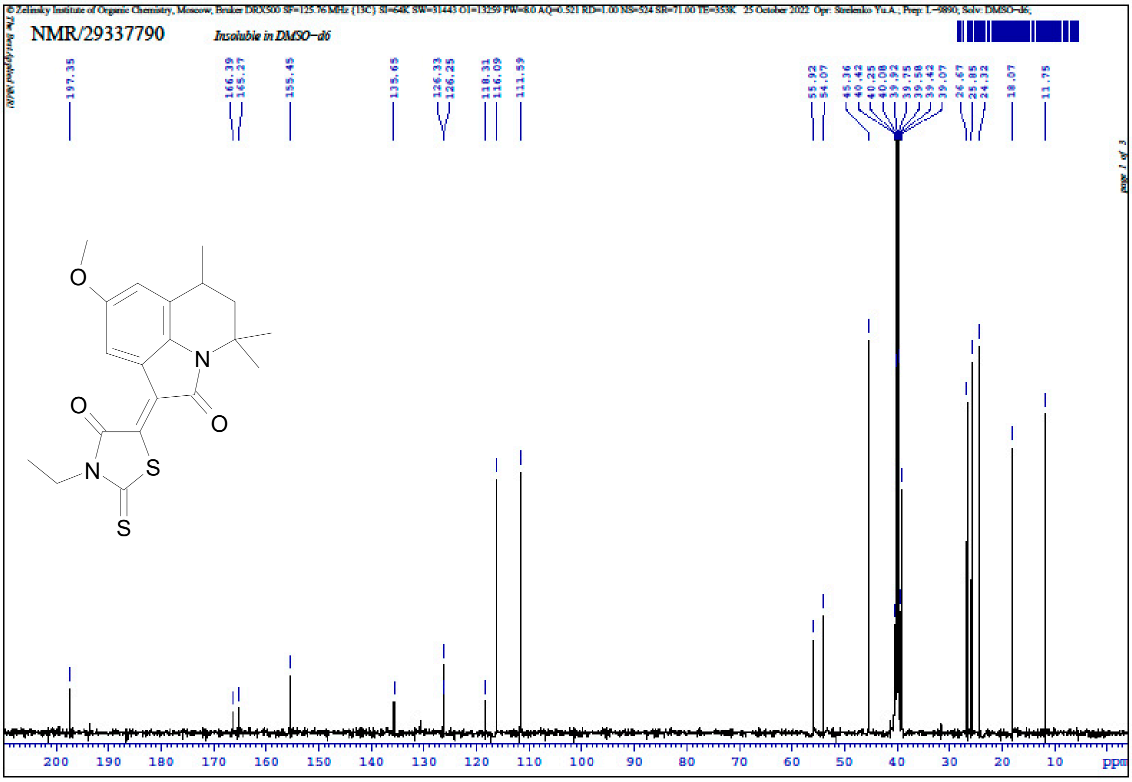

Figure S67. ^{13}C NMR spectrum of compound **12f**

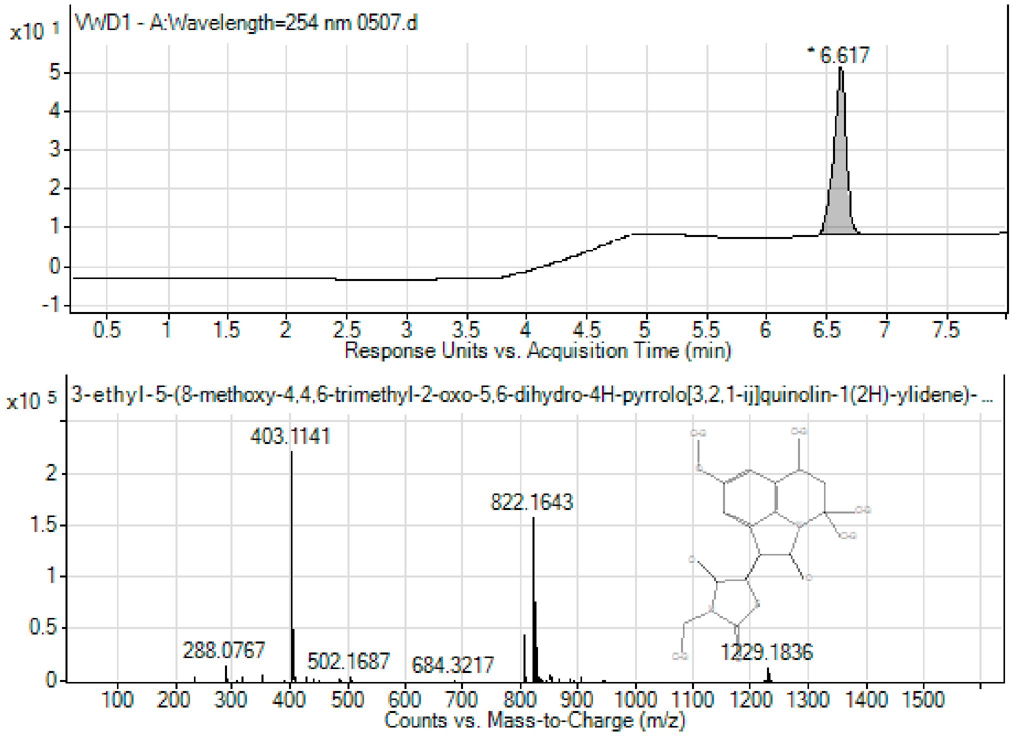

Figure S68. Data of HPLC-MS-ESI analysis of compound **12f**

^{1}H, ^{13}C NMR and data HPLC-HRMS-ESI spectra of (*Z*)-5-(8-fluoro-4,4,6-trimethyl-2-oxo-6-phenyl-5,6-dihydro-4*H*-pyrrolo[3,2,1-*ij*]quinolin-1(2*H*)-ylidene)-2-thioxothiazolidin-4-one 12g

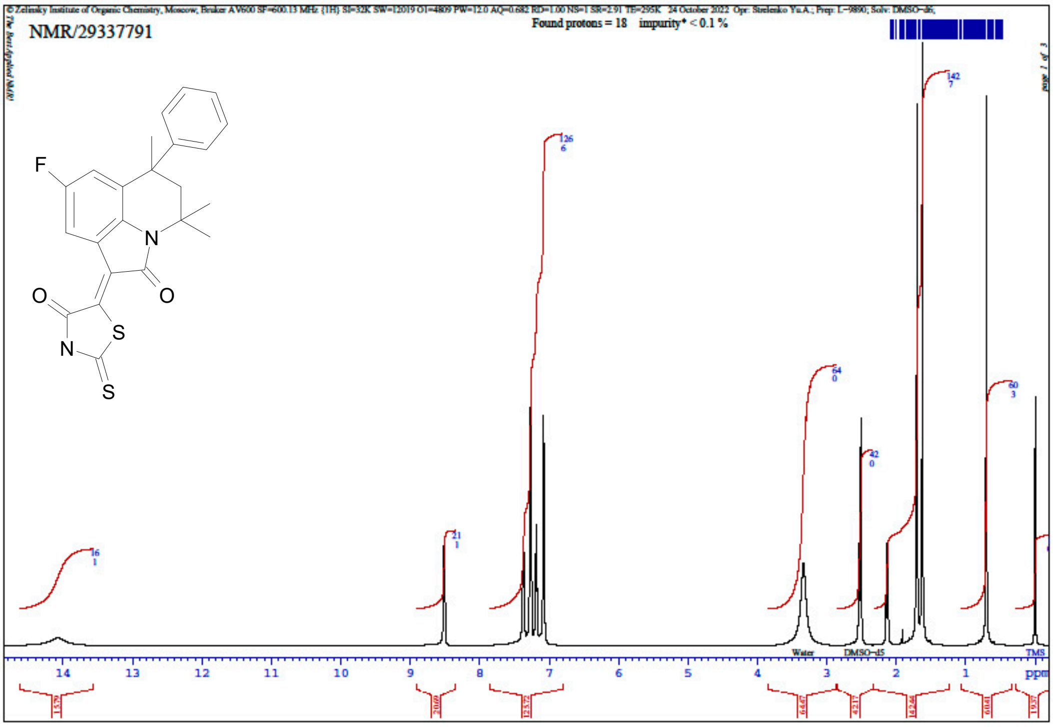

Figure S69. ^{1}H NMR spectrum of compound **12g**

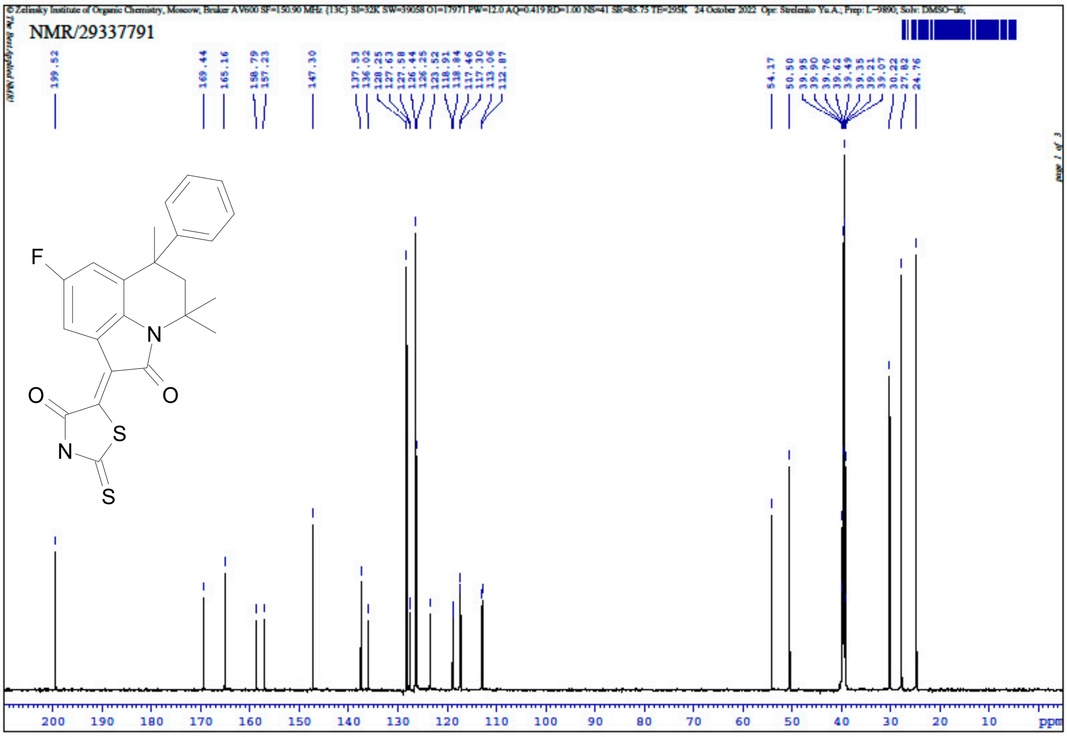

Figure S70. ^{13}C NMR spectrum of compound **12g**

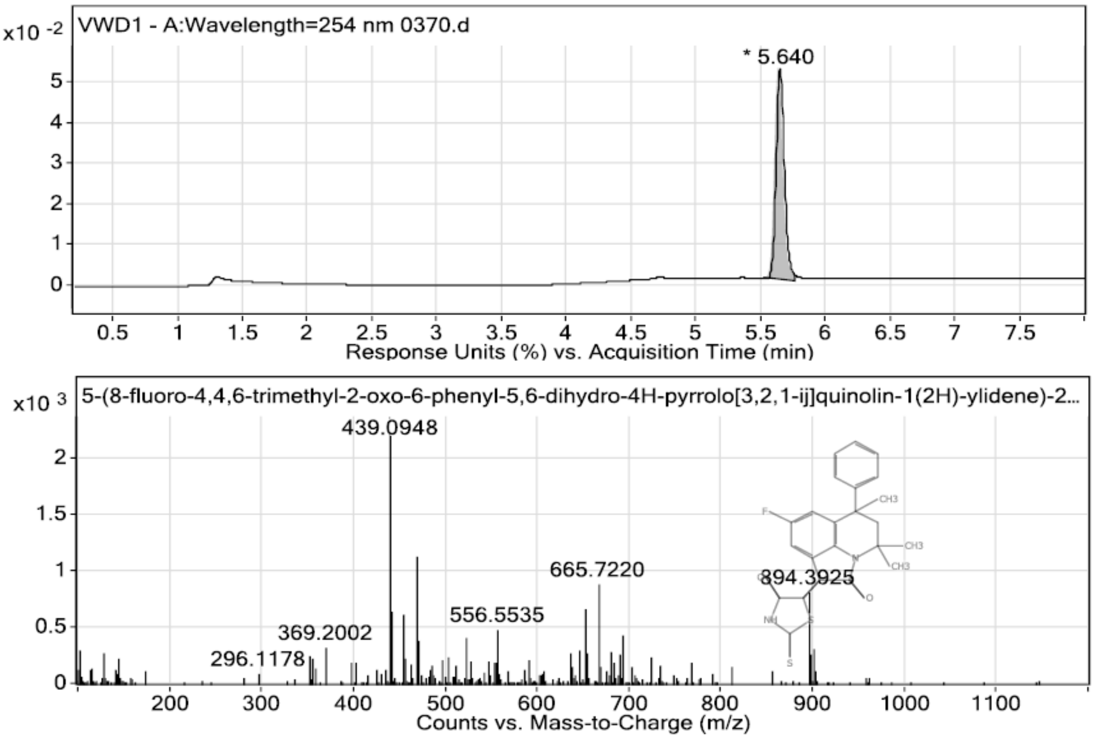

Figure S71. Data of HPLC-MS-ESI analysis of compound **12g**

^{1}H, ^{13}C NMR and data HPLC-HRMS-ESI spectra of (*Z*)-5-(8-bromo-4,4,6-trimethyl-2-oxo-6-phenyl-5,6-dihydro-4*H*-pyrrolo[3,2,1-*ij*]quinolin-1(2*H*)-ylidene)-2-thioxothiazolidin-4-one 12h

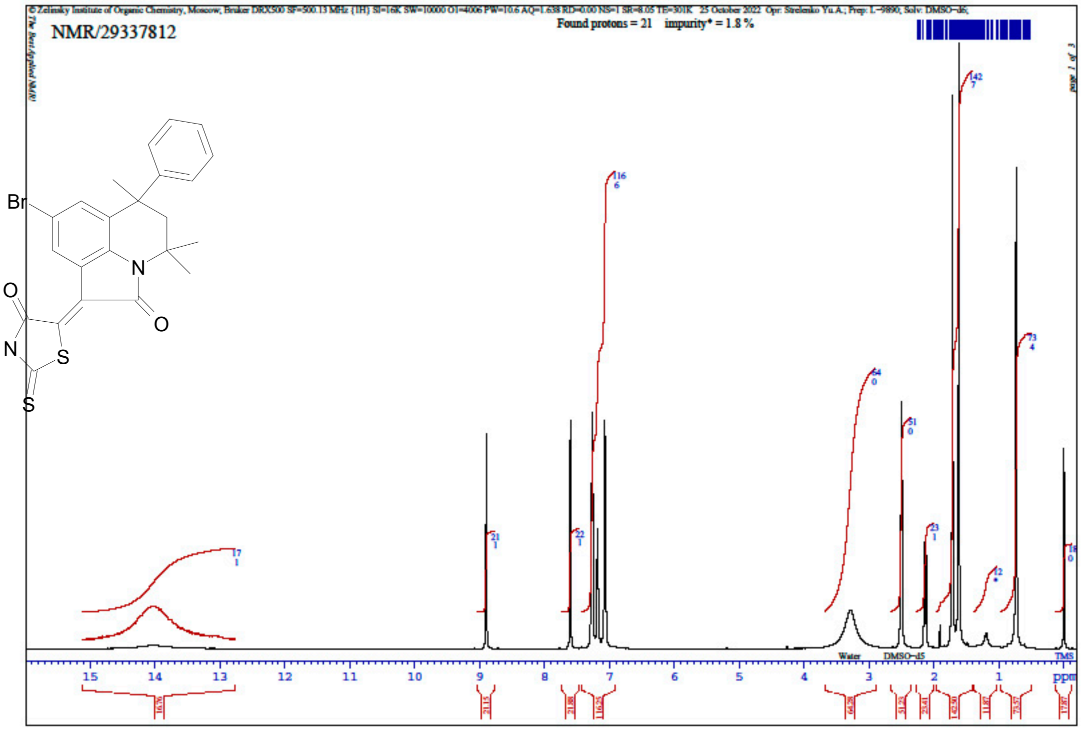

Figure S72. ^{1}H NMR spectrum of compound **12h**

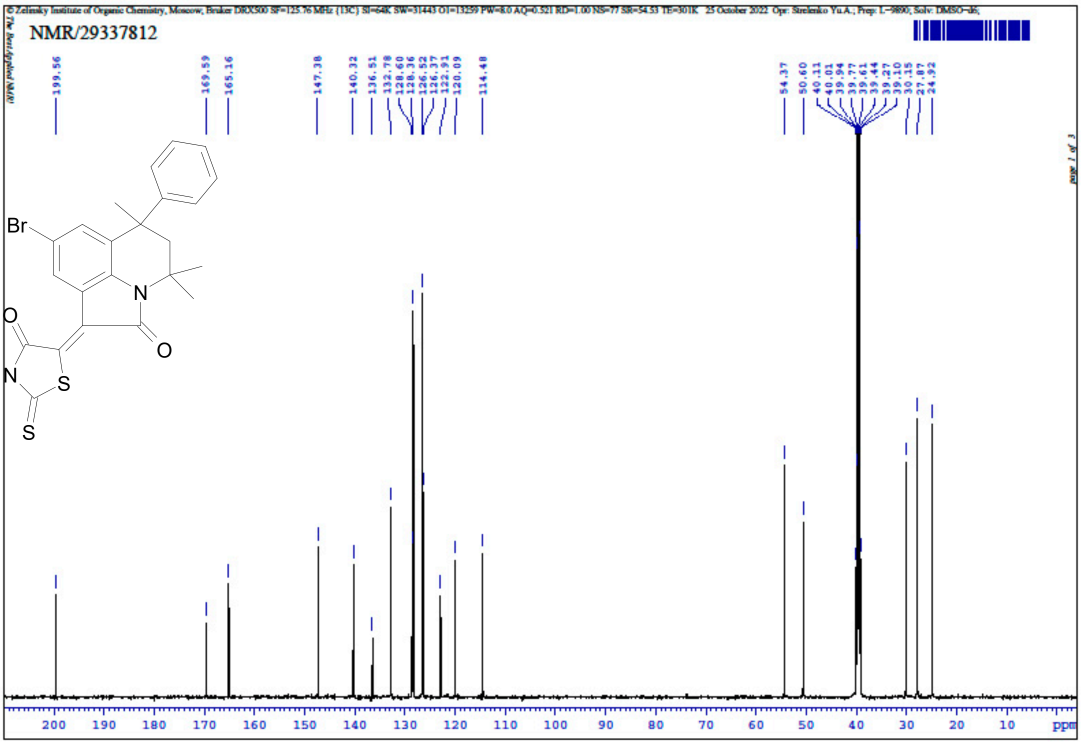

Figure S73. ^{13}C NMR spectrum of compound **12h**

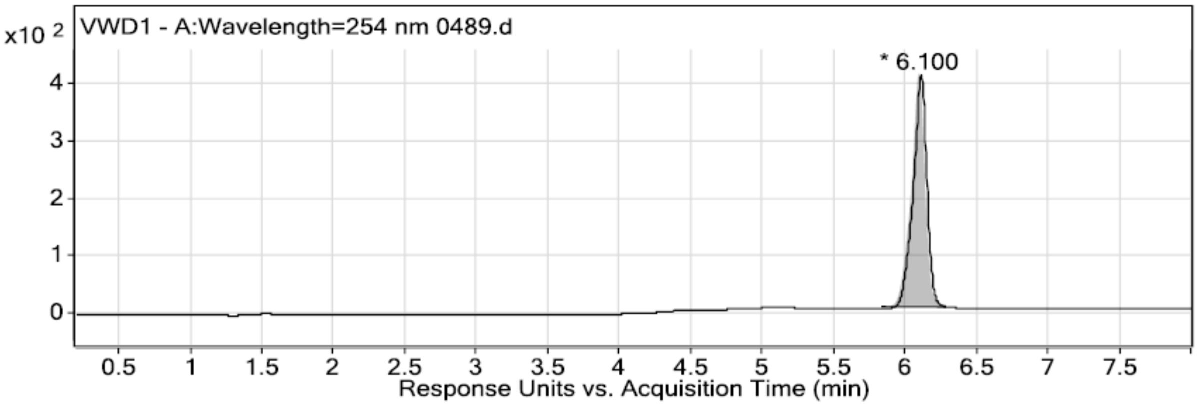

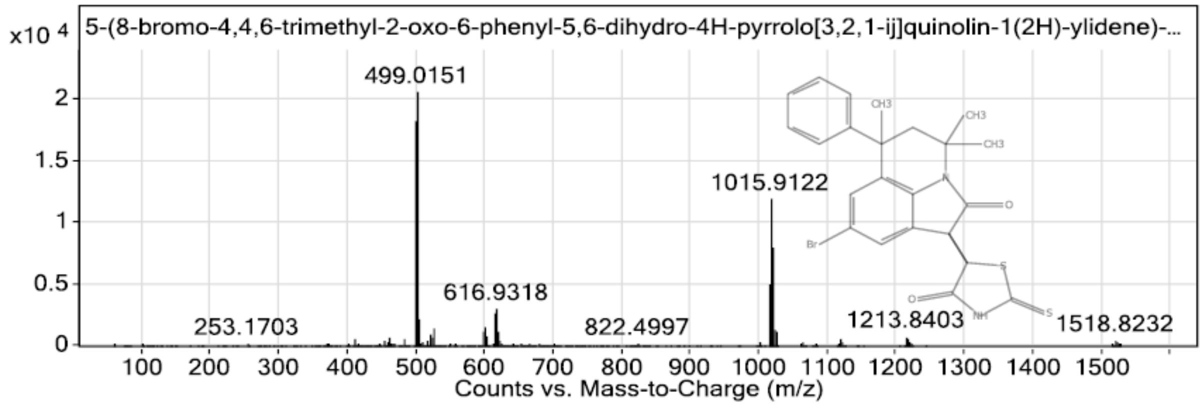

Figure S74. Data of HPLC-MS-ESI analysis of compound **12h**

^{1}H, ^{13}C NMR and data HPLC-HRMS-ESI spectra of (*Z*)-5-(8-iodo-4,4,6-trimethyl-2-oxo-6-phenyl-5,6-dihydro-4*H*-pyrrolo[3,2,1-*ij*]quinolin-1(2*H*)-ylidene)-2-thioxothiazolidin-4-one 12i

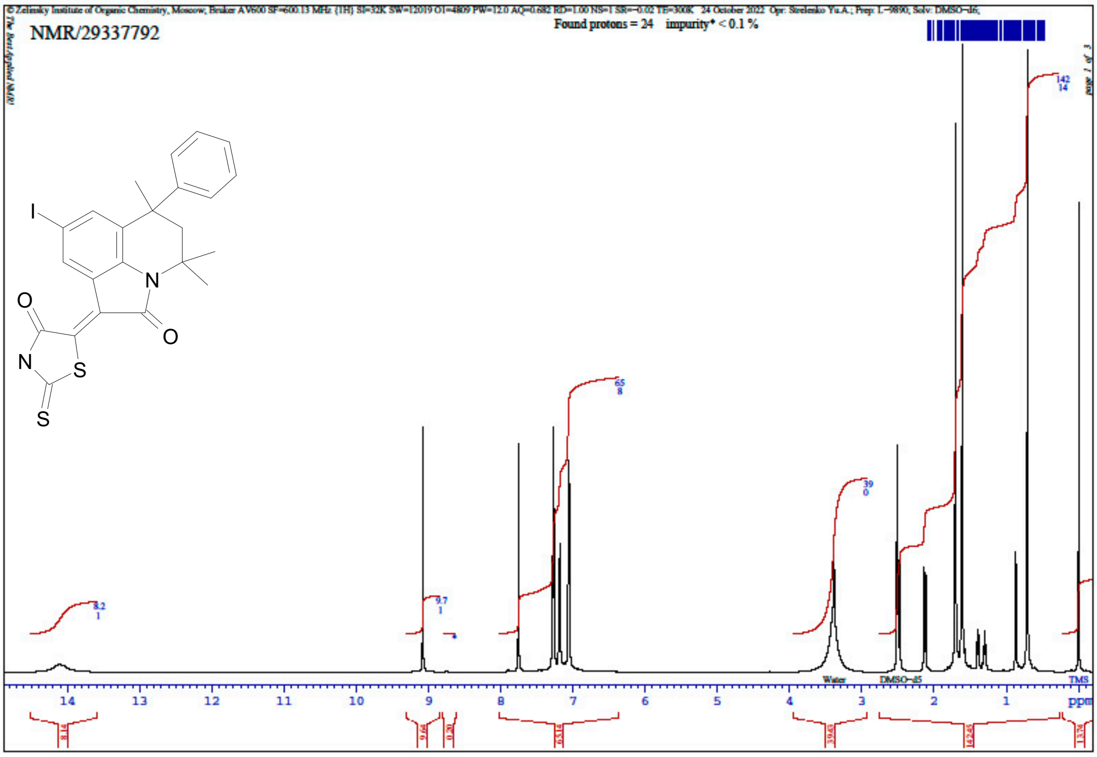

Figure S75. ^{1}H NMR spectrum of compound **12i**

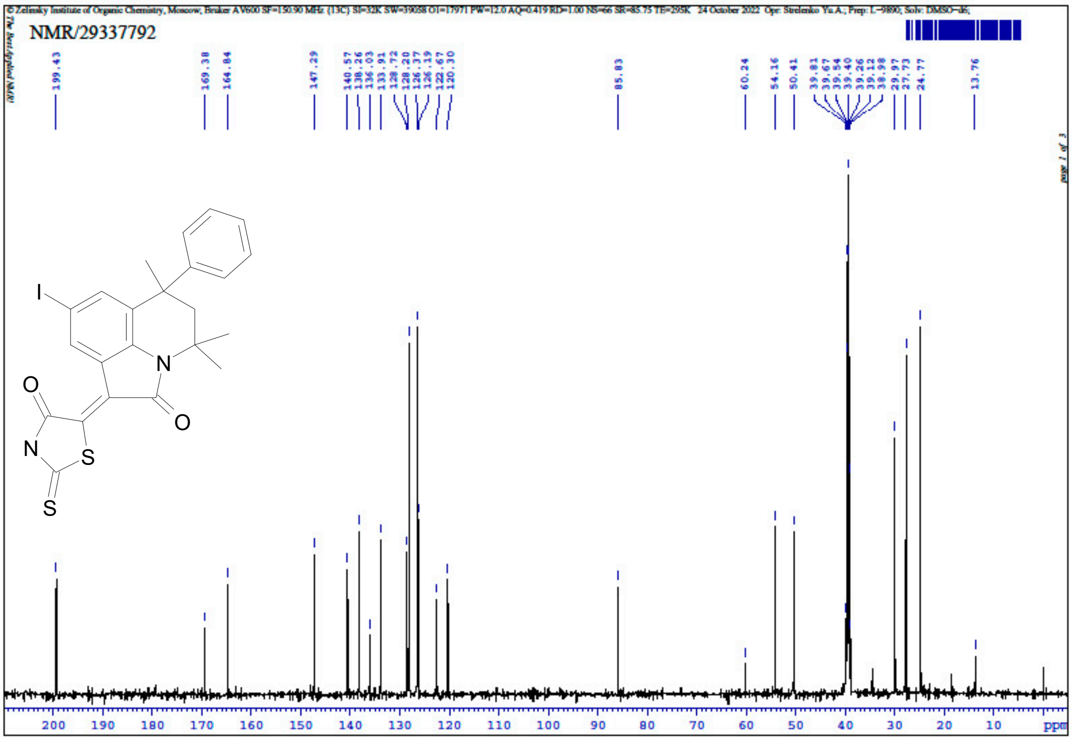

Figure S76. ^{13}C NMR spectrum of compound **12i**

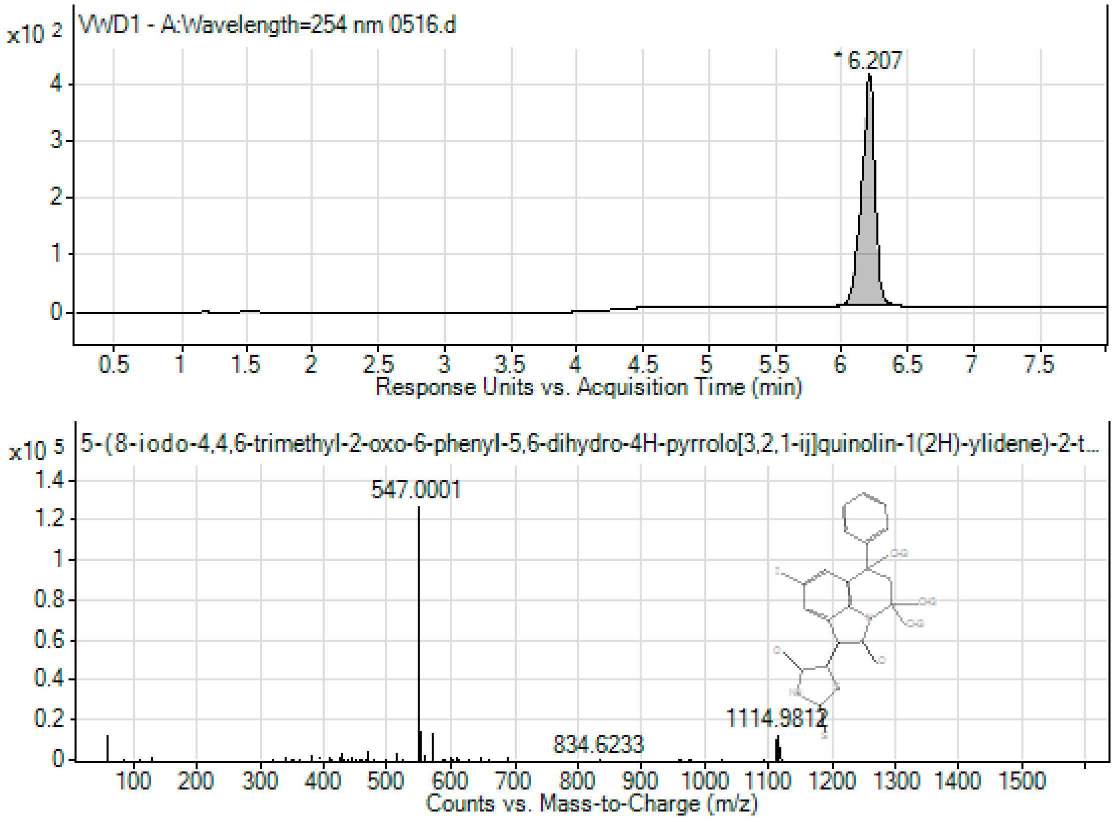

Figure S77. Data of HPLC-MS-ESI analysis of compound **12i**

^{1}H, ^{13}C NMR and data HPLC-HRMS-ESI spectra of (*Z*)-3-ethyl-2-thioxo-5-(4,4,6-trimethyl-2-oxo-6-phenyl-5,6-dihydro-4*H*-pyrrolo[3,2,1-*ij*]quinolin-1(2*H*)-ylidene)thiazolidin-4-one 12j

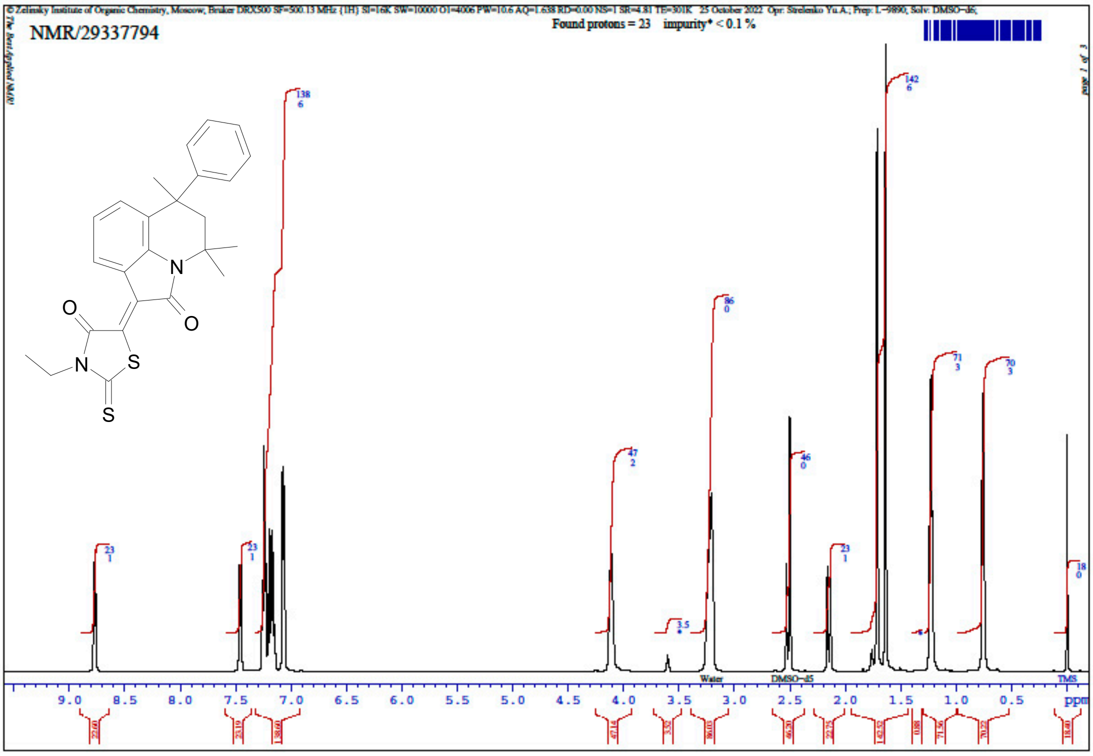

Figure S78. ^{1}H NMR spectrum of compound **12j**

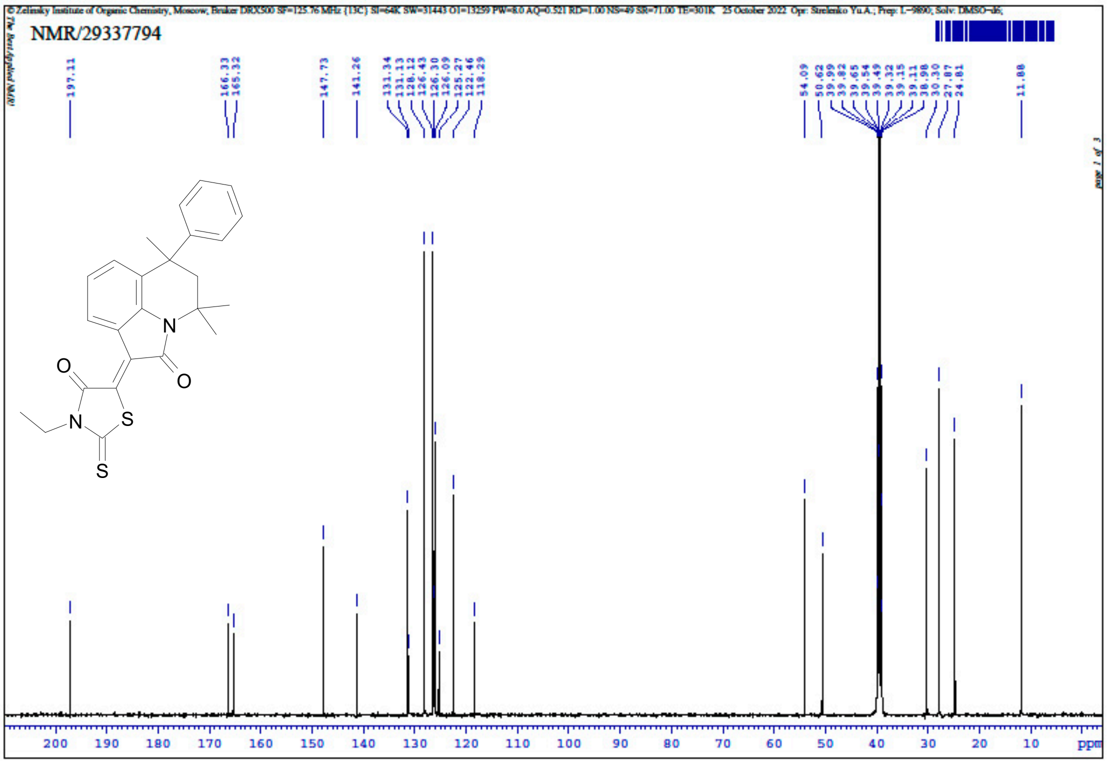

Figure S79. ^{13}C NMR spectrum of compound **12j**

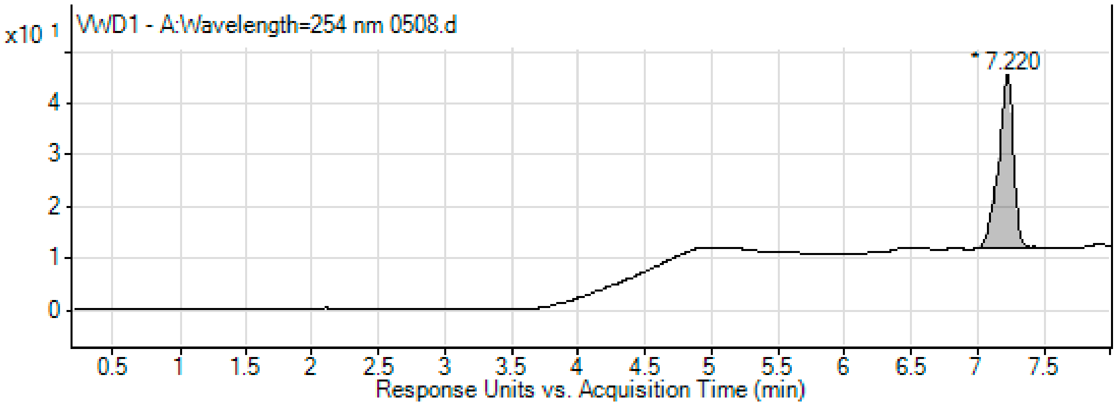

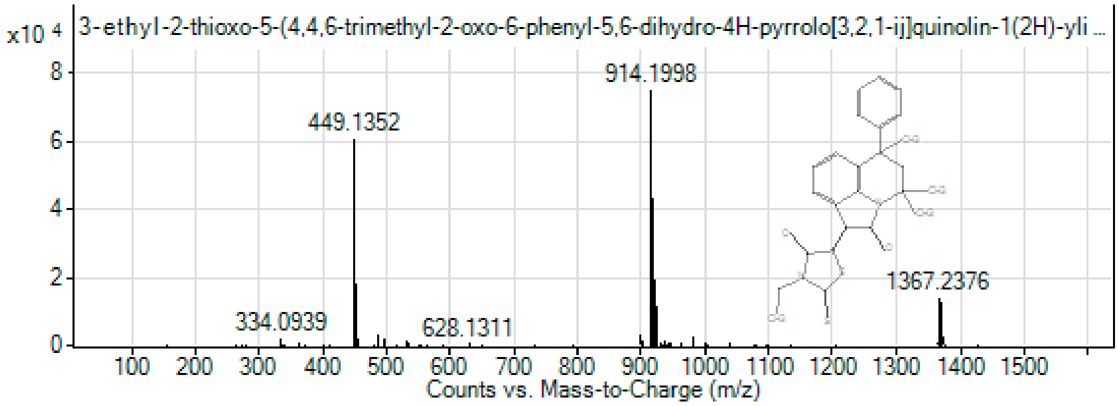

Figure S80. Data of HPLC-MS-ESI analysis of compound **12j**

^{1}H, ^{13}C NMR and data HPLC-HRMS-ESI spectra of (*Z*)-5-(6-(4-chlorophenyl)-8-fluoro-4,4,6-trimethyl-2-oxo-5,6-dihydro-4*H*-pyrrolo[3,2,1-*ij*]quinolin-1(2*H*)-ylidene)-2-thioxothiazolidin-4-one 12k

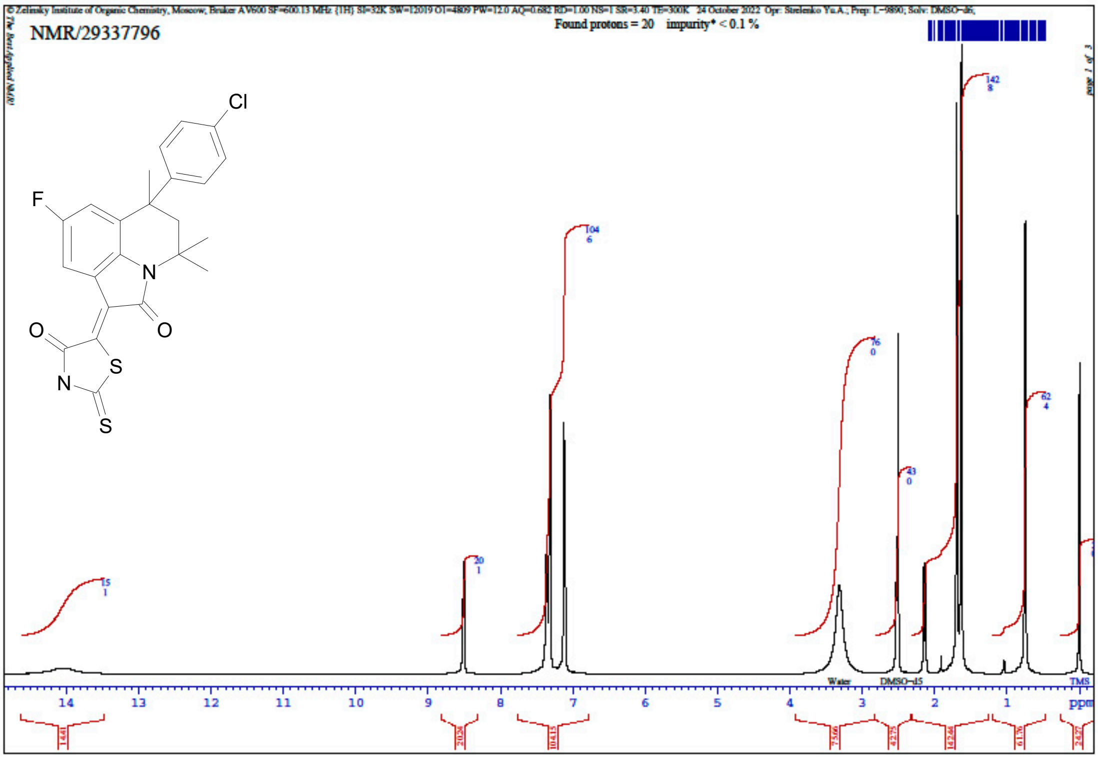

Figure S81. ^{1}H NMR spectrum of compound **12k**

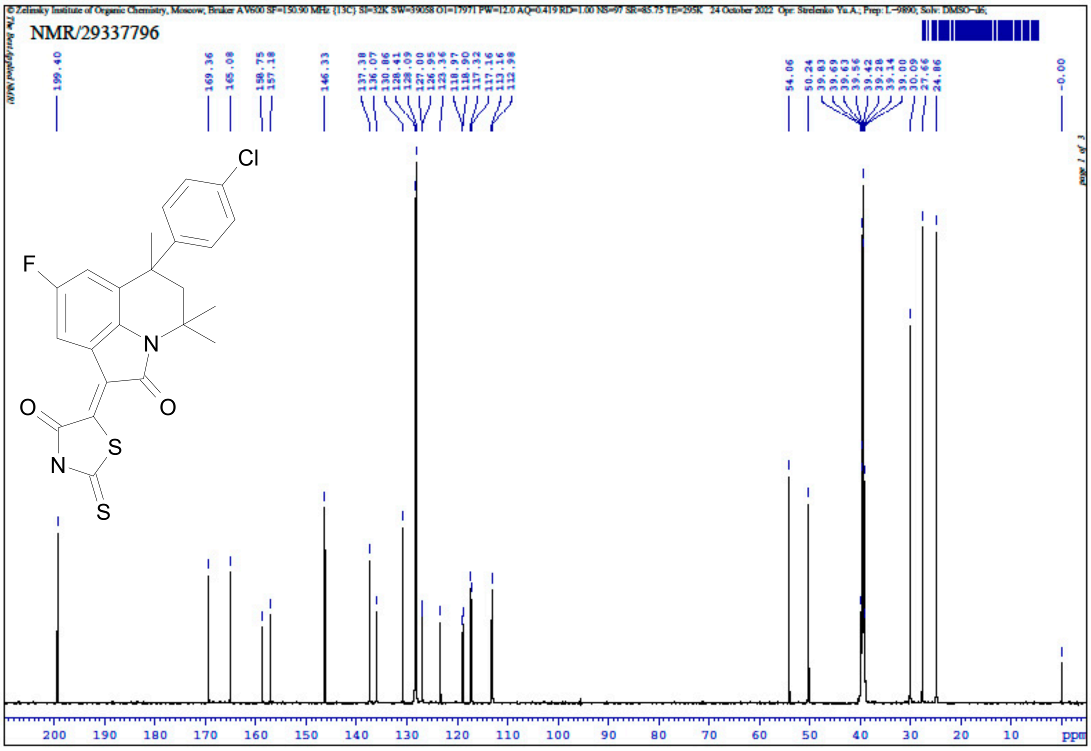

Figure S82. ^{13}C NMR spectrum of compound **12k**

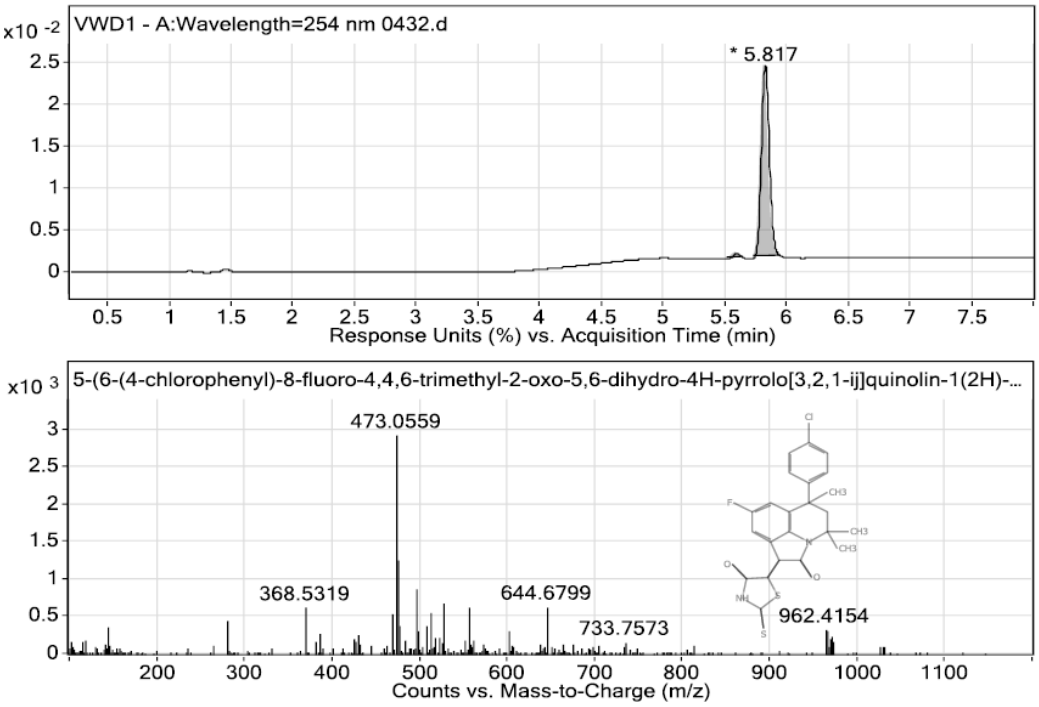

Figure S83. Data of HPLC-MS-ESI analysis of compound **12k**

^{1}H, ^{13}C NMR and data HPLC-HRMS-ESI spectra of (*Z*)-5-(6-(4-chlorophenyl)-8-iodo-4,4,6-trimethyl-2-oxo-5,6-dihydro-4*H*-pyrrolo[3,2,1-*ij*]quinolin-1(2*H*)-ylidene)-2-thioxothiazolidin-4-one 12l

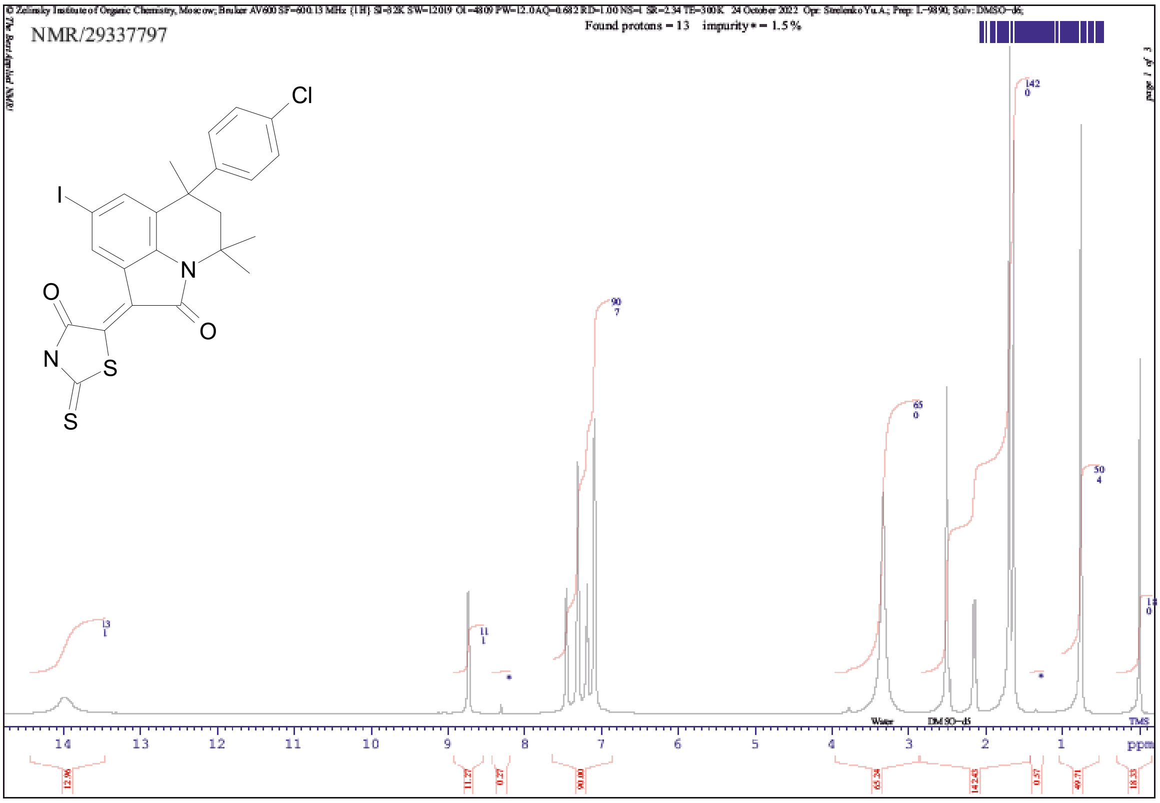

Figure S84. ^{1}H NMR spectrum of compound **12l**

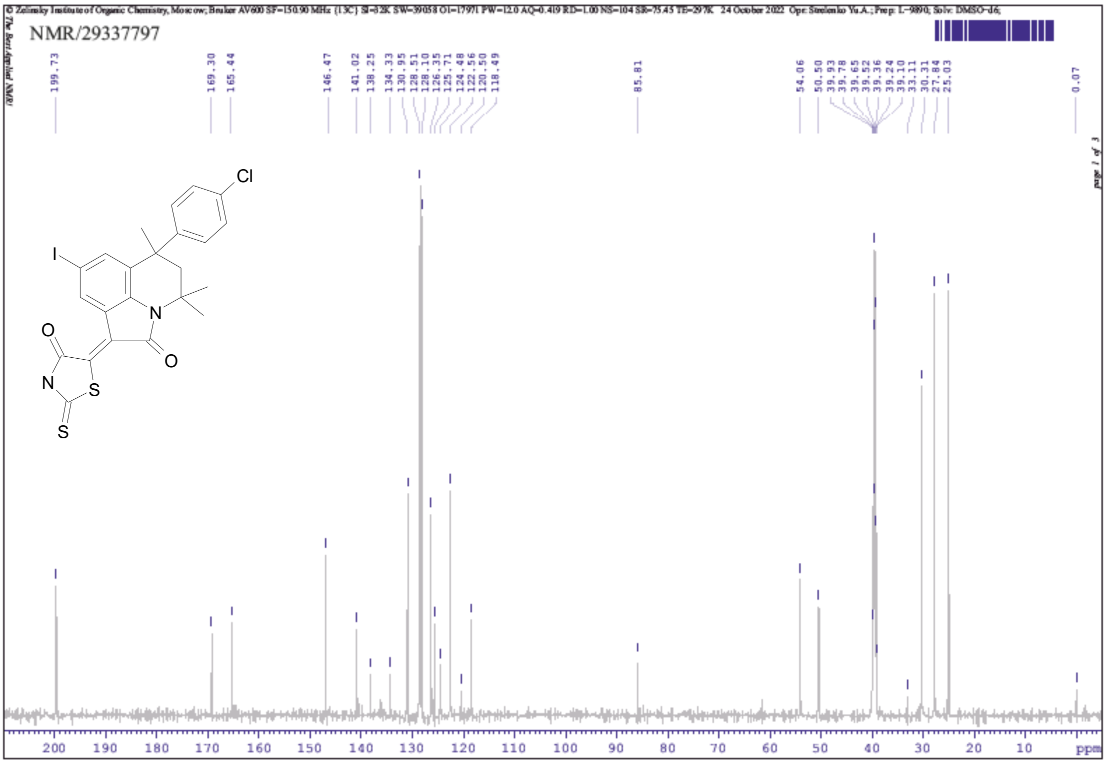

Figure S85. ^{13}C NMR spectrum of compound **12l**

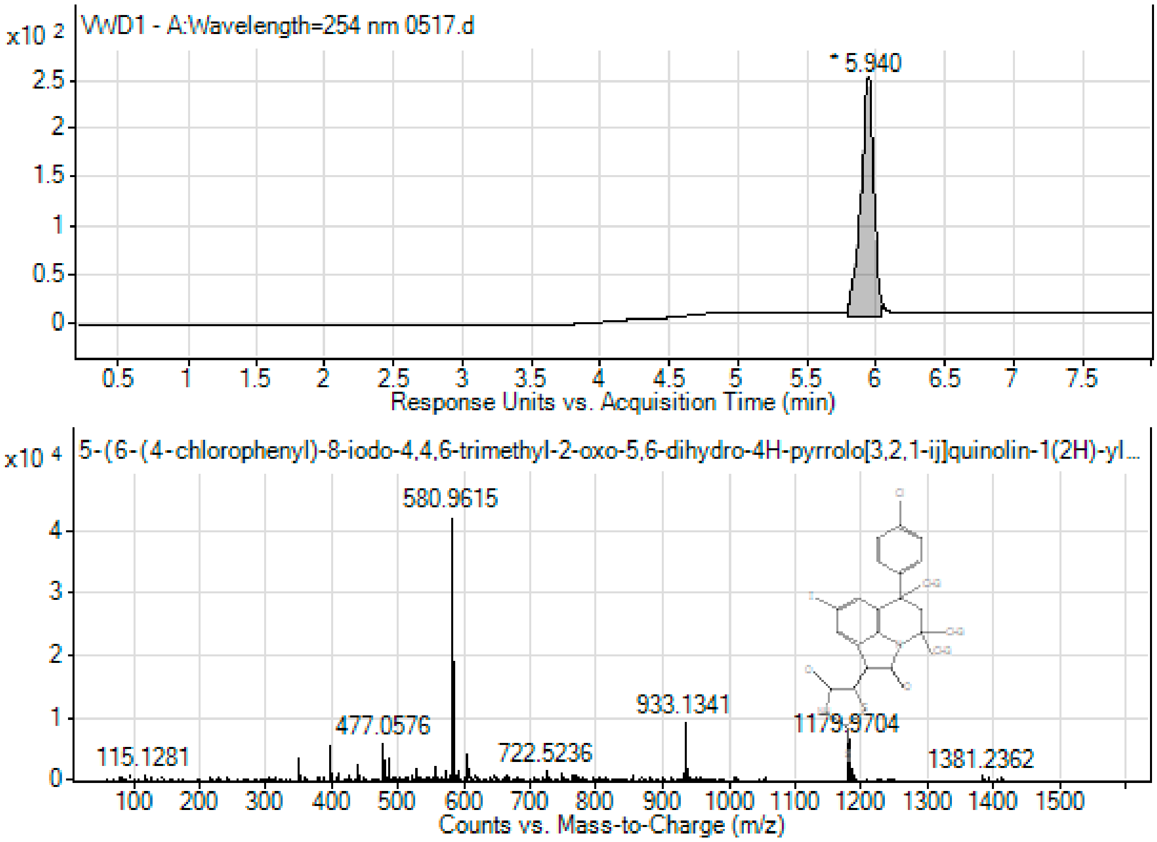

Figure S86. Data of HPLC-MS-ESI analysis of compound **12l**

^{1}H, ^{13}C NMR, IR and data HPLC-HRMS-ESI spectra of (*Z*)-5-(6-(4-chlorophenyl)-8-fluoro-4,4,6-trimethyl-2-oxo-5,6-dihydro-4*H*-pyrrolo[3,2,1-*ij*]quinolin-1(2*H*)-ylidene)-3-ethyl-2-thioxothiazolidin-4-one 12m

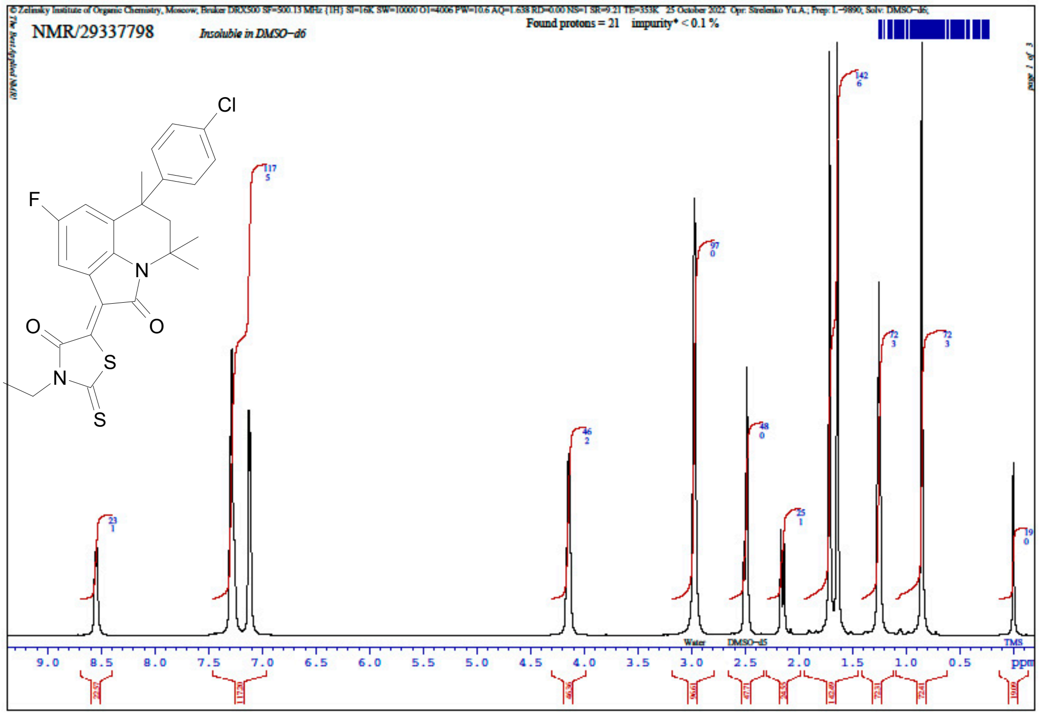

Figure S87. ^{1}H NMR spectrum of compound **12m**

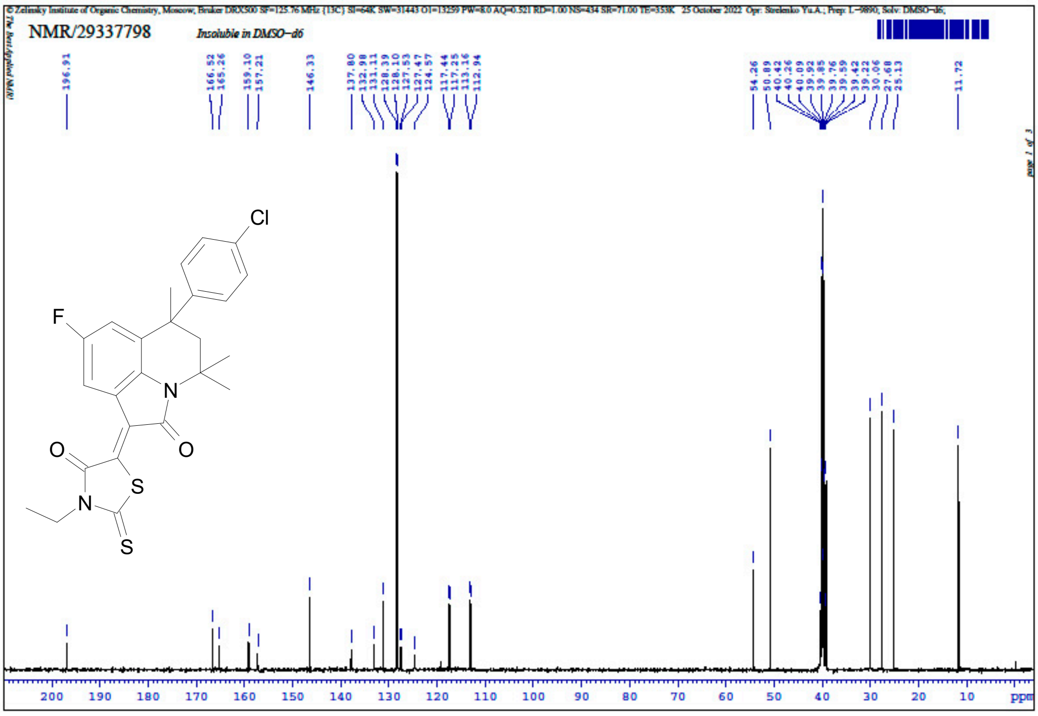

Figure S88. ^{13}C NMR spectrum of compound **12m**

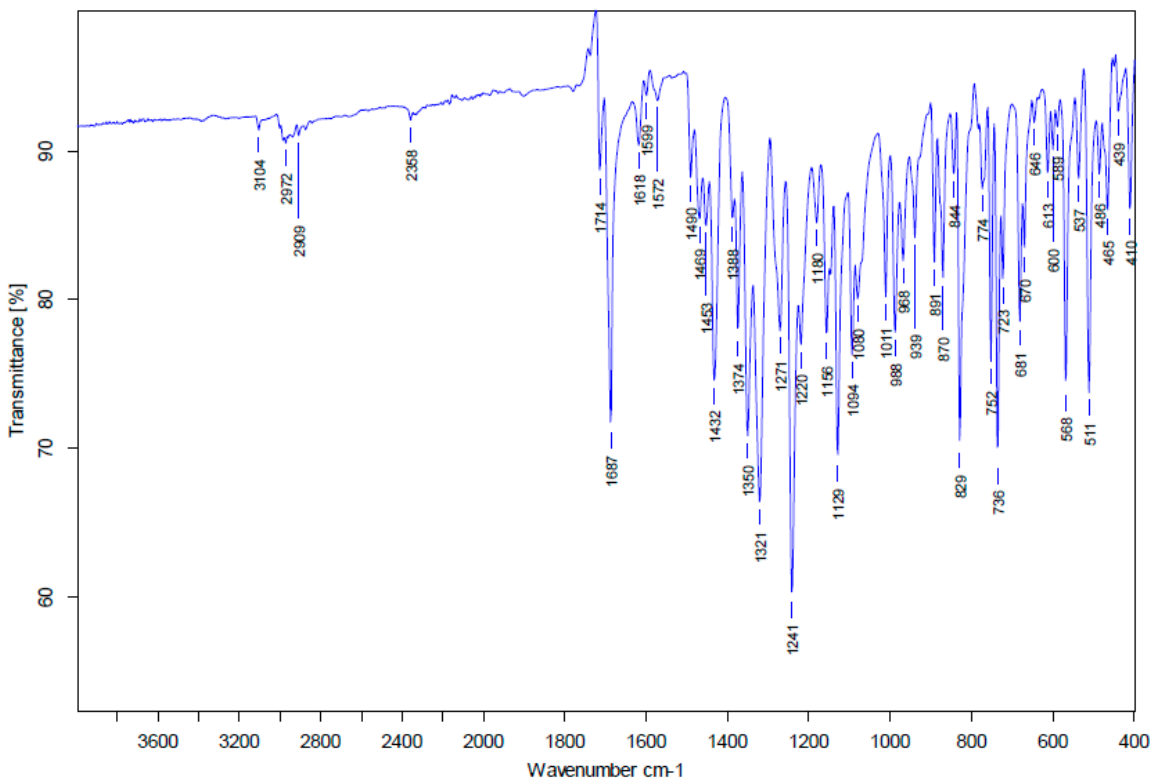

Figure S89. IR spectrum of compound **12m**

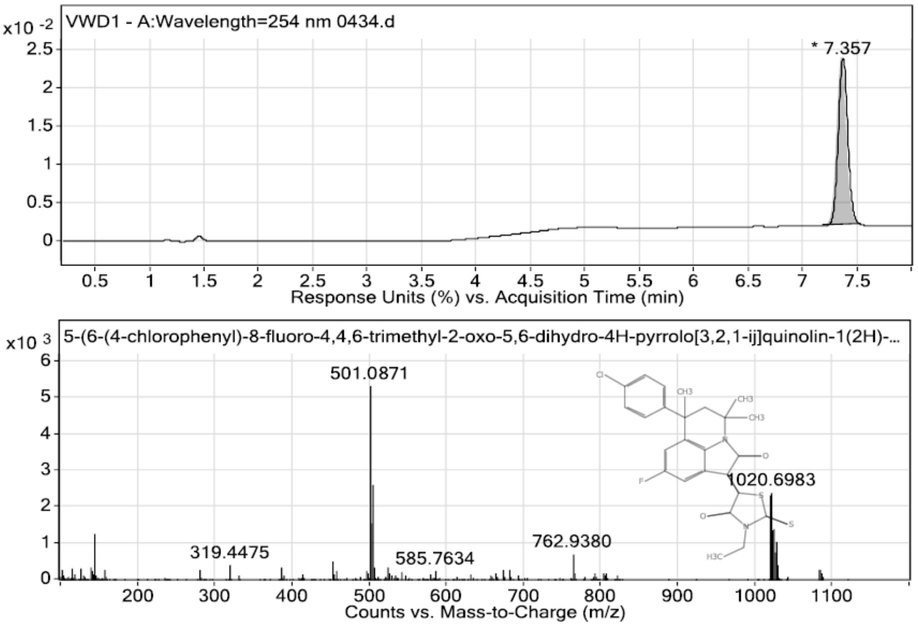

Figure S90. Data of HPLC-MS-ESI analysis of compound **12m**

^{1}H, ^{13}C NMR, IR and data HPLC-HRMS-ESI spectra of (*Z*)-5-(6',6'-dimethyl-2'-oxo-5',6'-dihydrospiro[cyclohexane-1,4'-pyrrolo[3,2,1-*ij*]quinolin]-1'(2'*H*)-ylidene)-2-thioxothiazolidin-4-one 12n

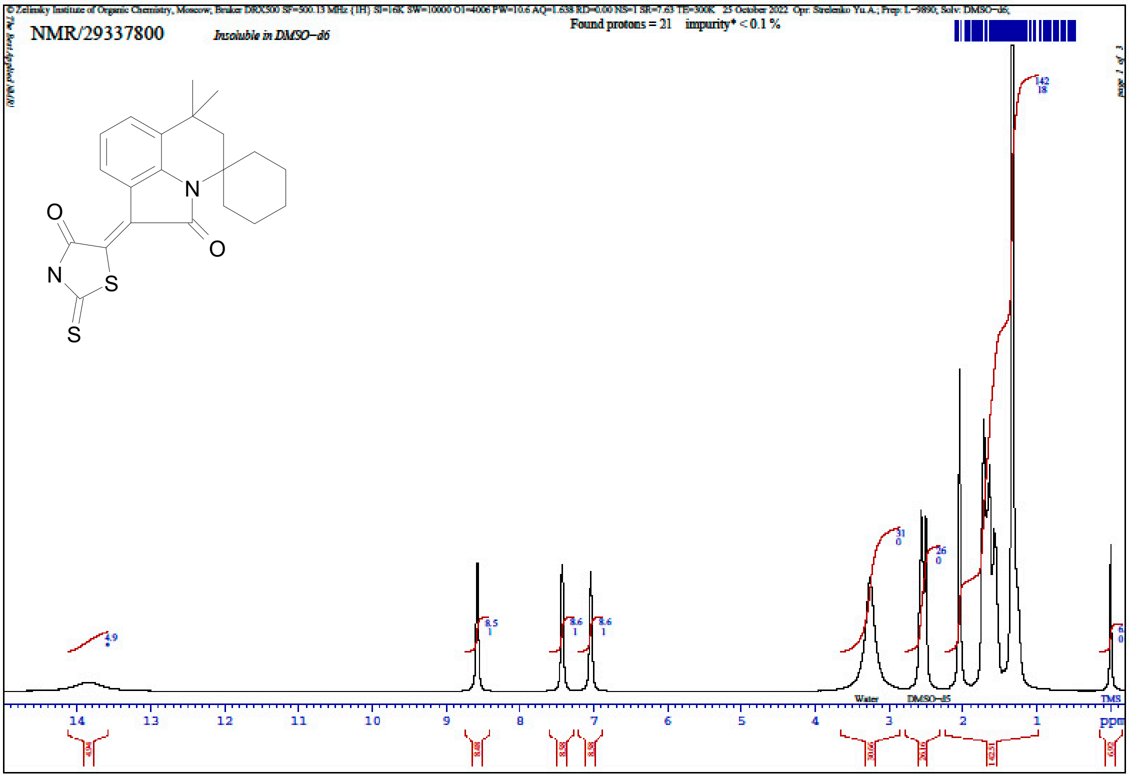

Figure S91. ^{1}H NMR spectrum of compound **12n**

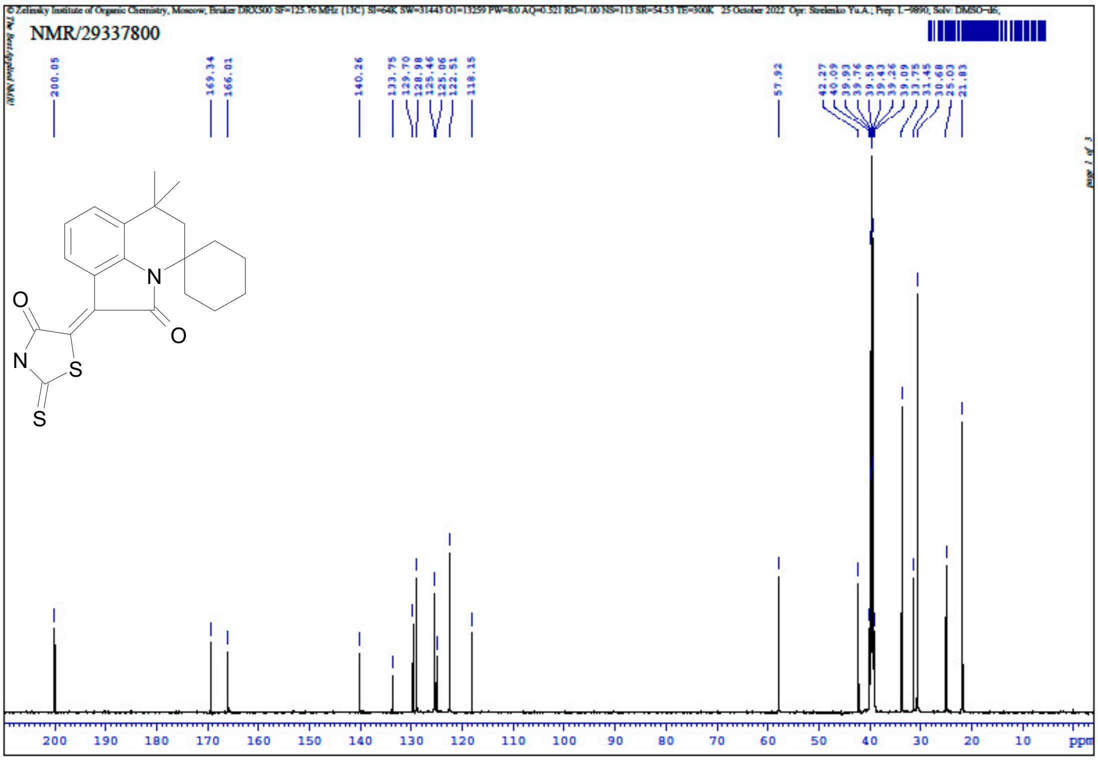

Figure S92. ^{13}C NMR spectrum of compound **12n**

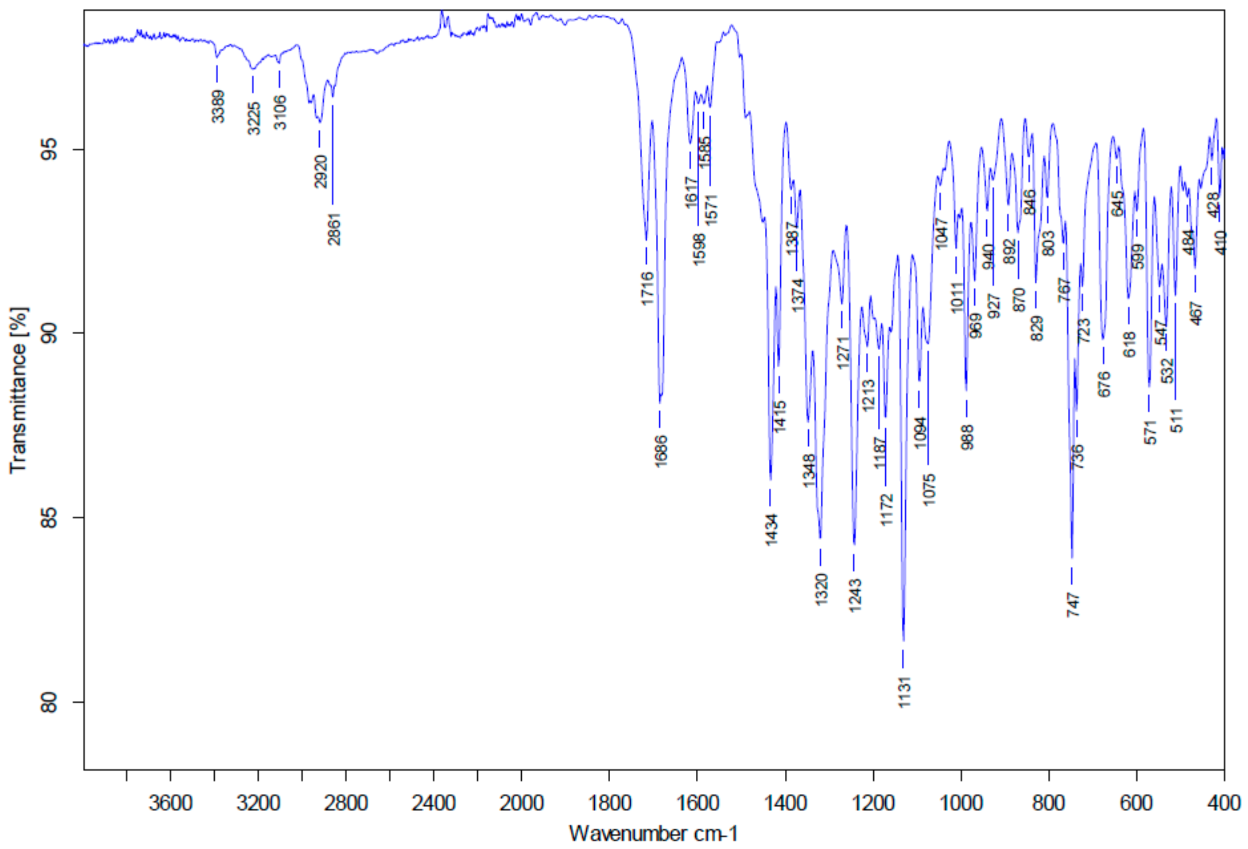

Figure S93. IR spectrum of compound **12n**

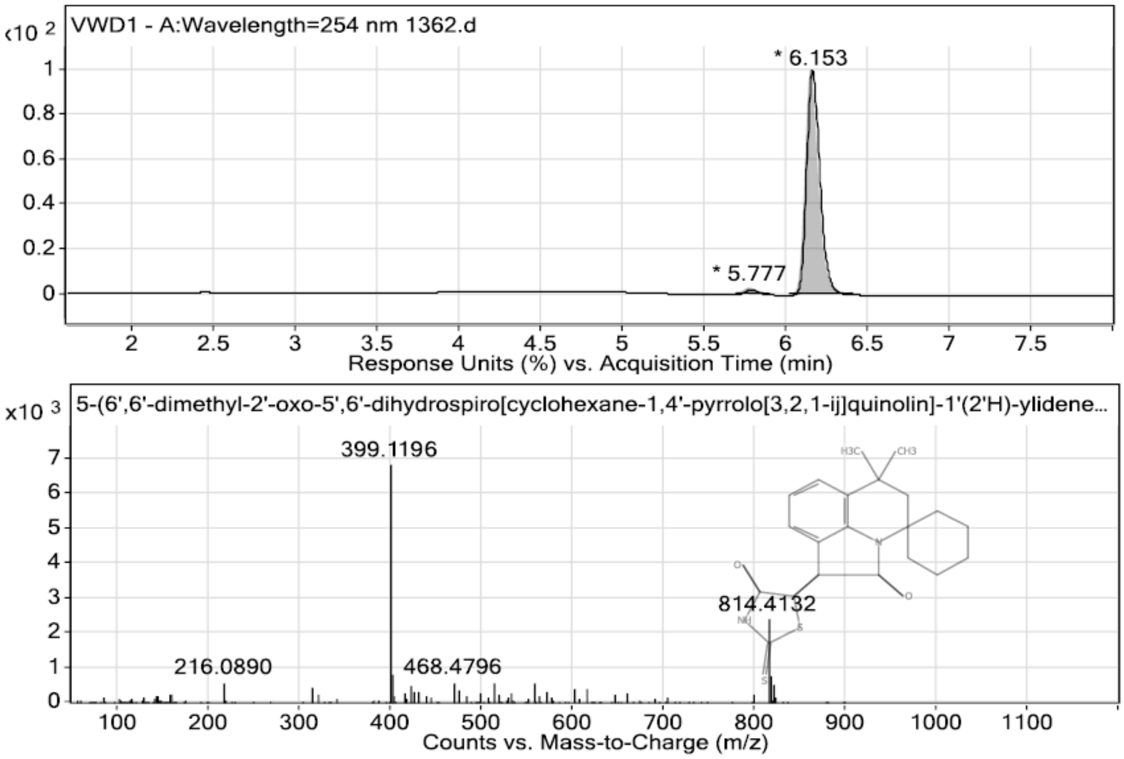

Figure S94. Data of HPLC-MS-ESI analysis of compound **12n**

^{1}H, ^{13}C NMR and data HPLC-HRMS-ESI spectra of (*Z*)-5-(6',8'-dimethyl-2'-oxo-5',6'-dihydrospiro[cyclohexane-1,4'-pyrrolo[3,2,1-*ij*]quinolin]-1'(2'*H*)-ylidene)-2-thioxothiazolidin-4-one 12o

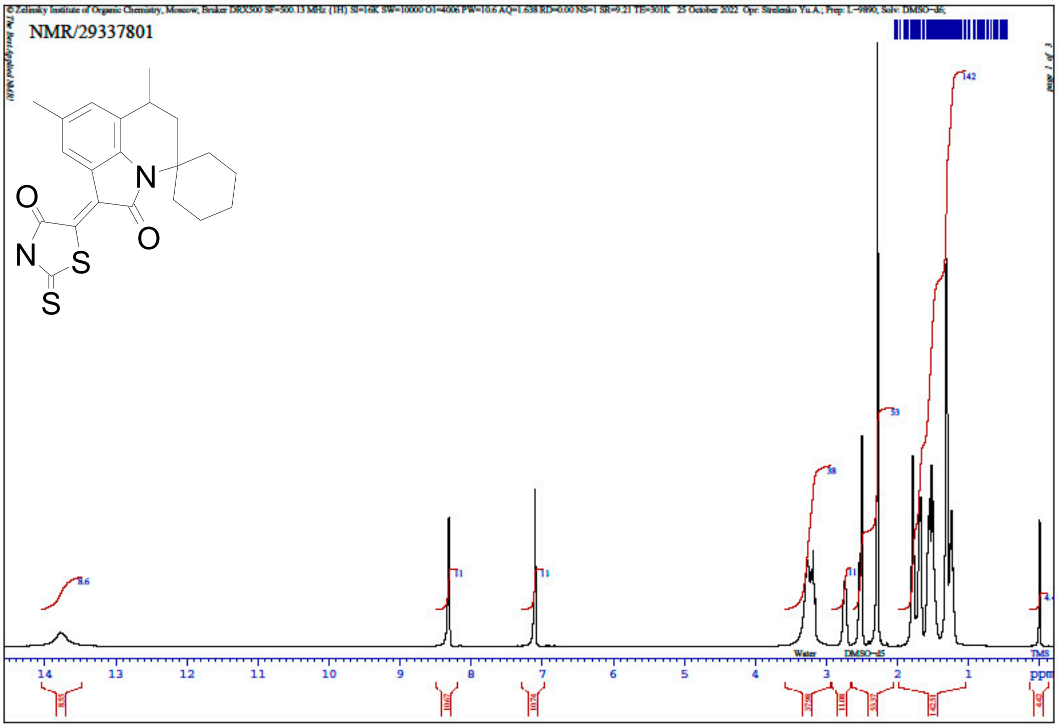

Figure S95. ^{1}H NMR spectrum of compound **12o**

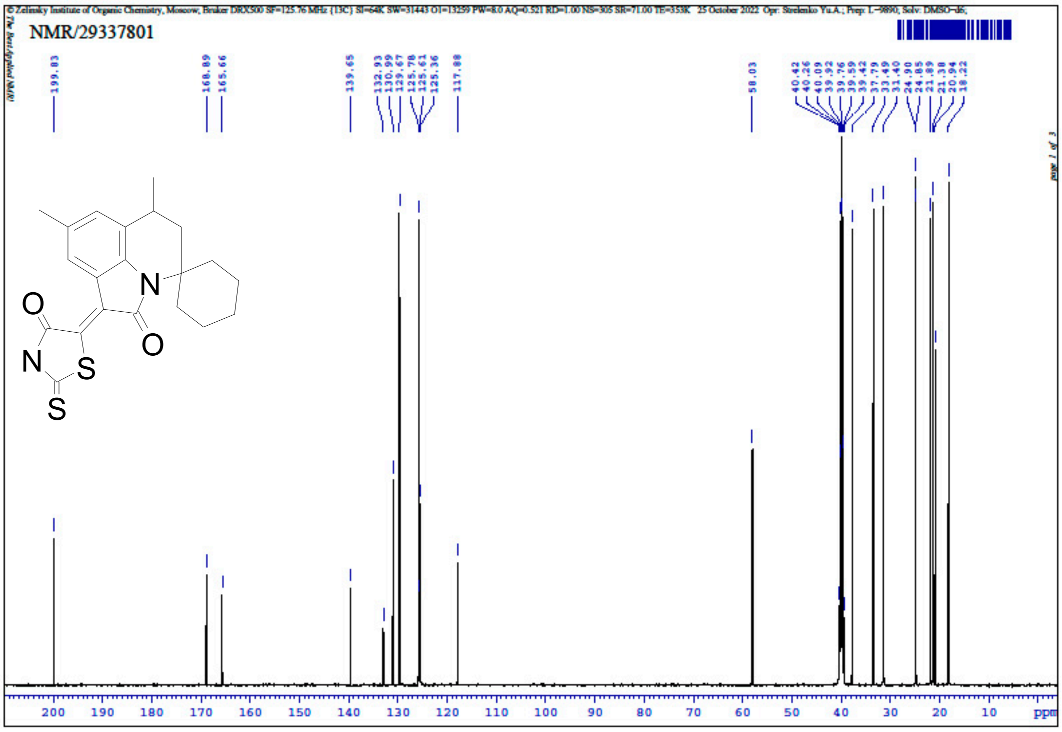

Figure S96. ^{13}C NMR spectrum of compound **12o**

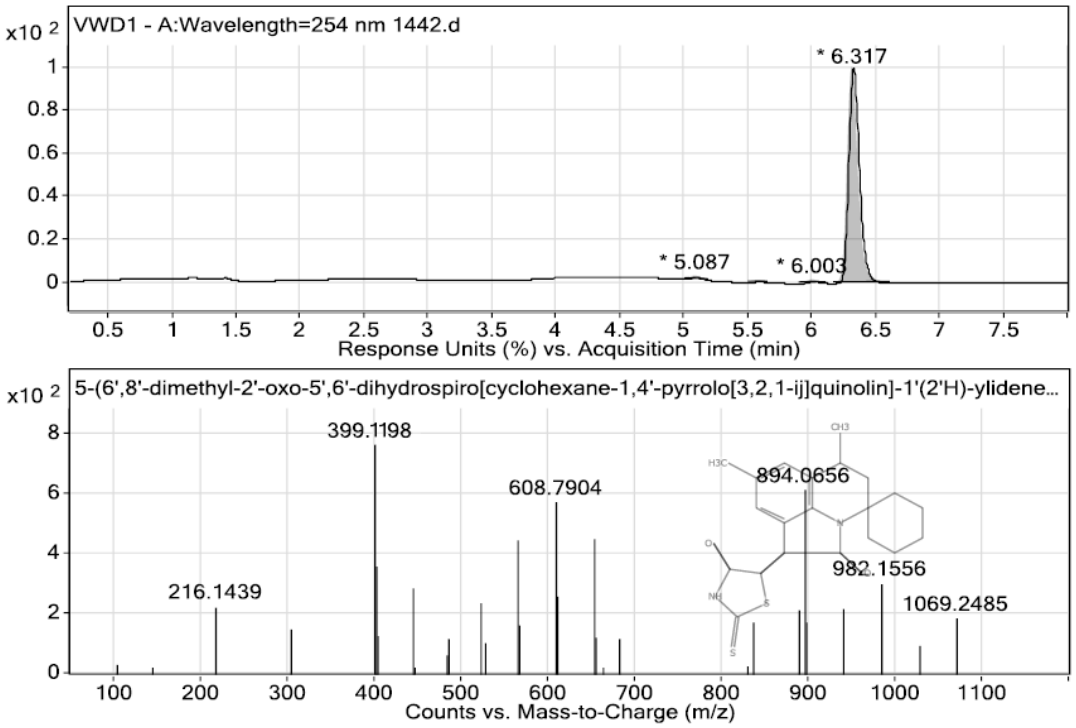

Figure S97. Data of HPLC-MS-ESI analysis of compound **12o**

^{1}H, ^{13}C NMR and data HPLC-HRMS-ESI spectra of (*Z*)-5-(8'-fluoro-6'-methyl-2'-oxo-5',6'-dihydrospiro[cyclopentane-1,4'-pyrrolo[3,2,1-*ij*]quinolin]-1'(2'*H*)-ylidene)-2-thioxothiazolidin-4-one 12p

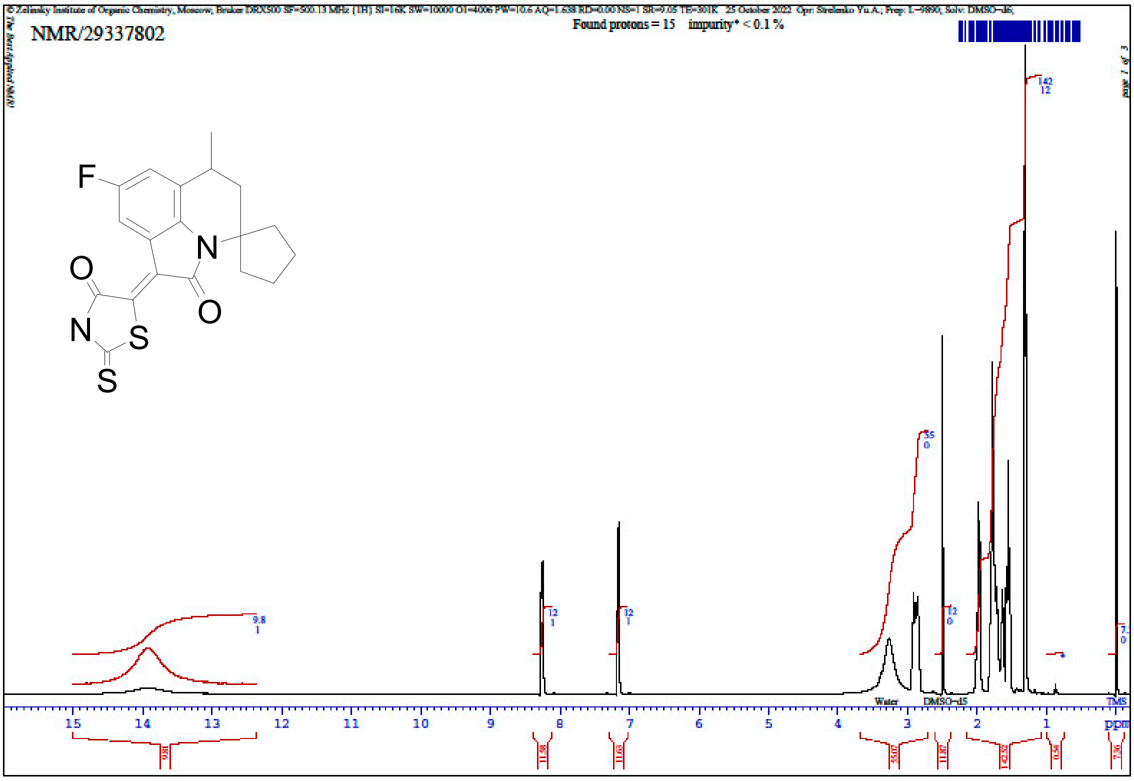

Figure S98. ^{1}H NMR spectrum of compound **12p**

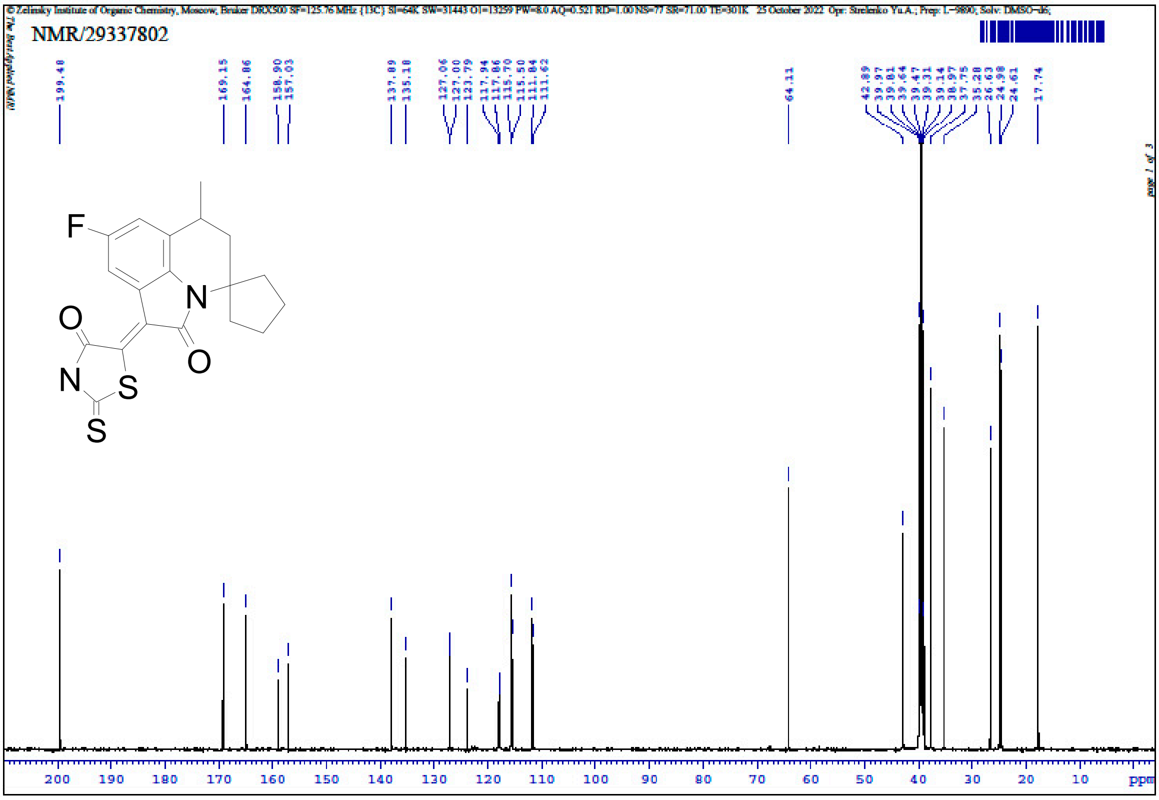

Figure S99. ^{13}C NMR spectrum of compound **12p**

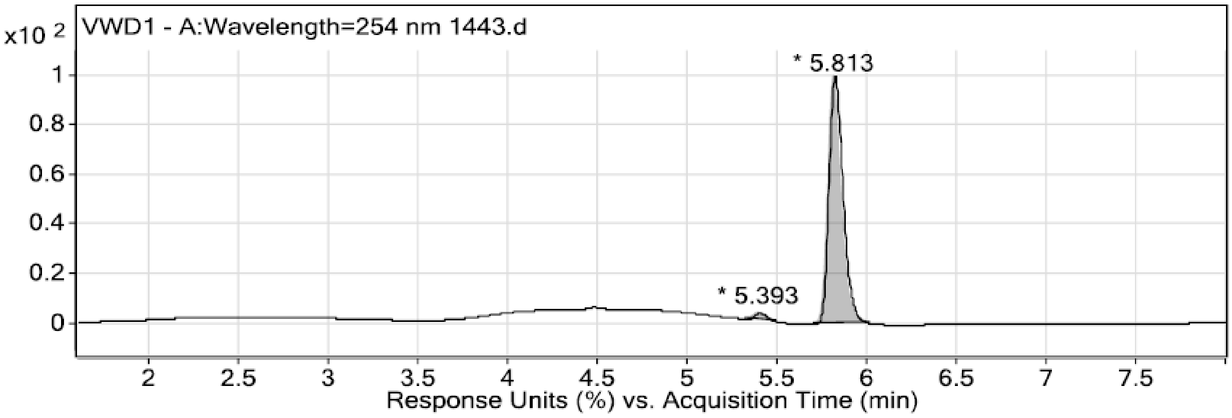

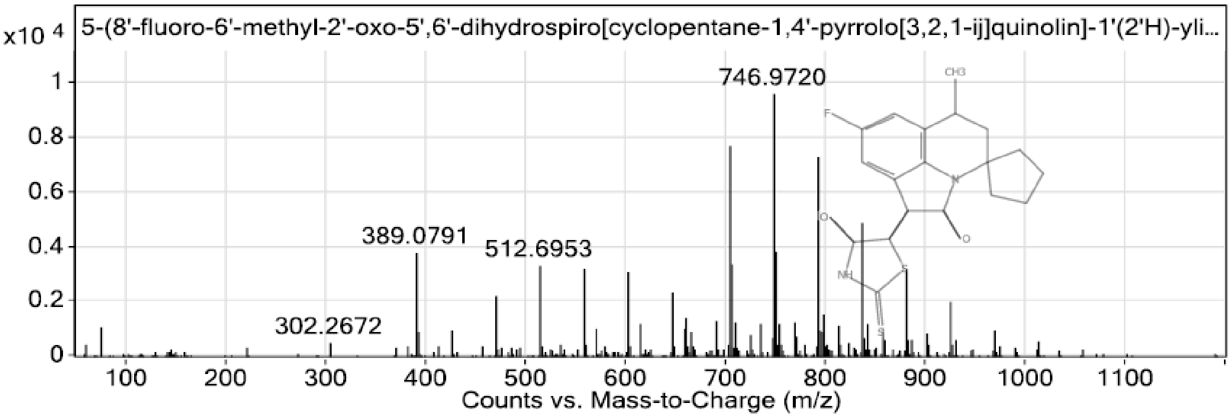

Figure S100. Data of HPLC-MS-ESI analysis of compound **12p**

^{1}H, ^{13}C NMR and data HPLC-HRMS-ESI spectra of (*Z*)-5-(8'-fluoro-6'-methyl-2'-oxo-5',6'-dihydrospiro[cycloheptane-1,4'-pyrrolo[3,2,1-*ij*]quinolin]-1'(2'*H*)-ylidene)-2-thioxothiazolidin-4-one 12q

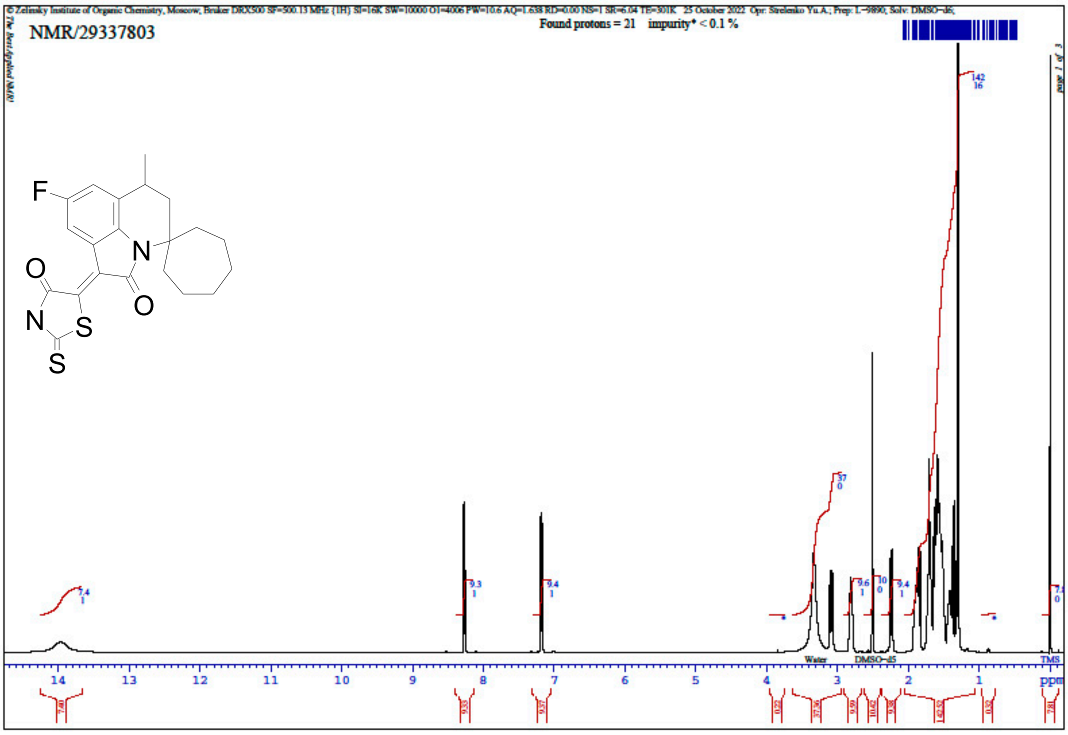

Figure S101. ^{1}H NMR spectrum of compound **12q**

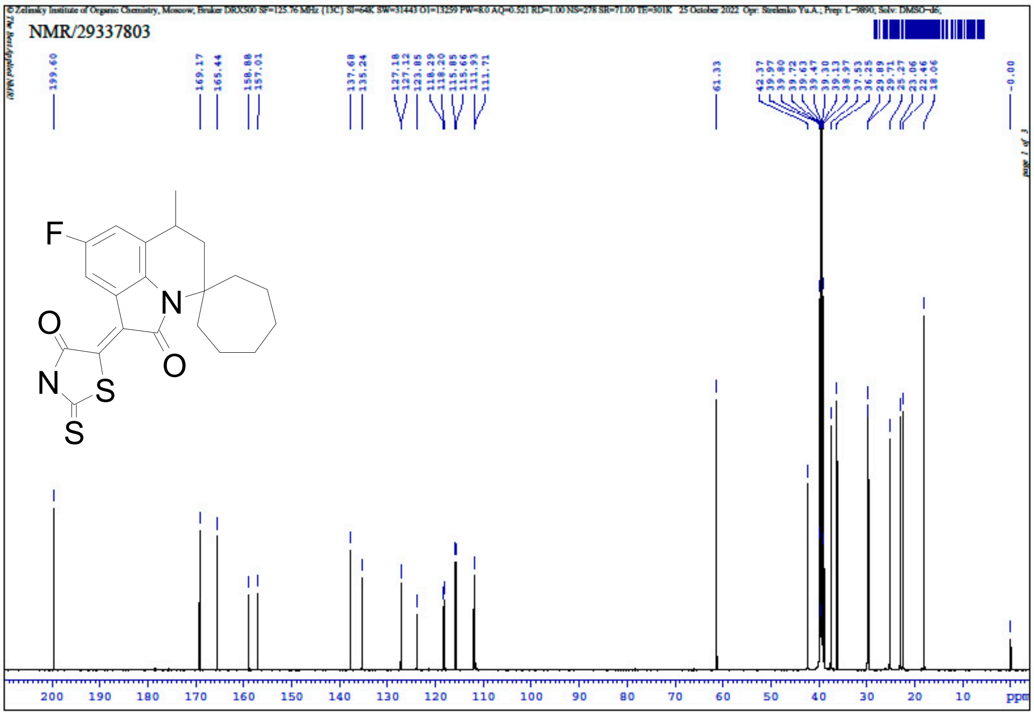

Figure S102. ^{13}C NMR spectrum of compound **12q**

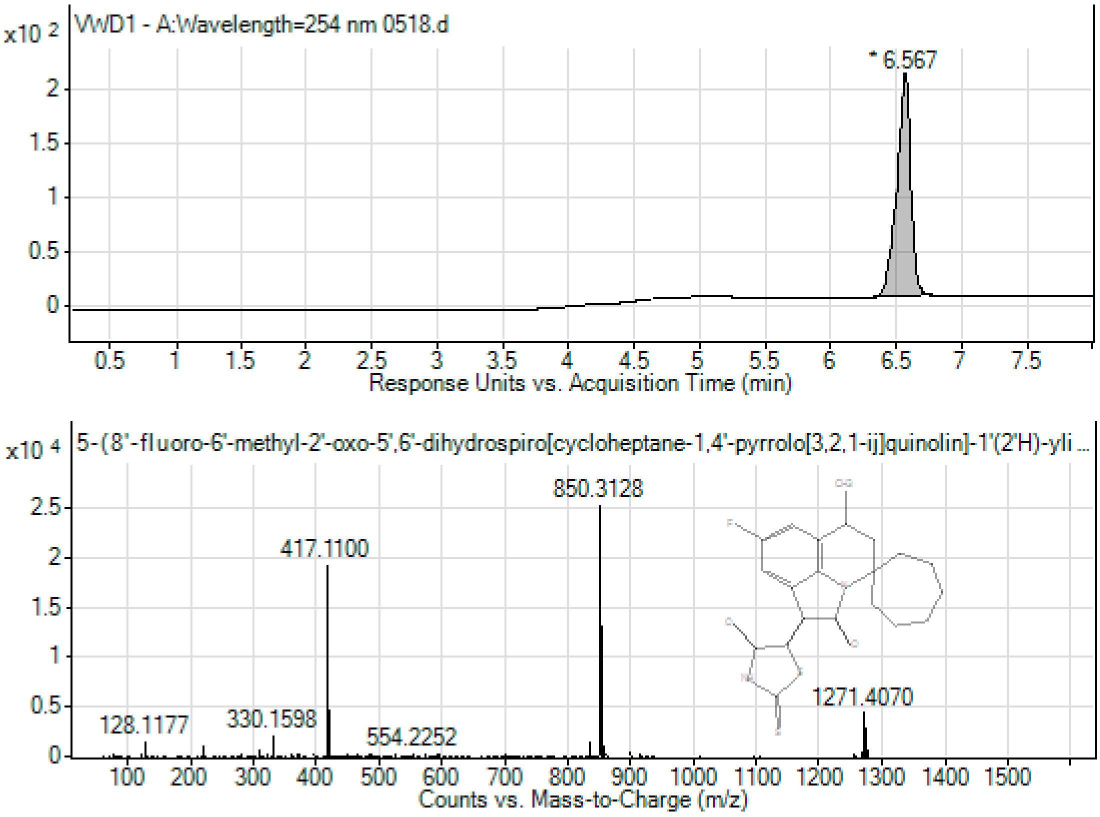

Figure S103. Data of HPLC-MS-ESI analysis of compound **12q**

^{1}H, ^{13}C NMR, IR and data HPLC-HRMS-ESI spectra of (*Z*)-5-(8-fluoro-4,4,6-trimethyl-2-oxo-6-phenyl-5,6-dihydro-4*H*-pyrrolo[3,2,1-*ij*]quinolin-1(2*H*)-ylidene)-3-(prop-2-yn-1-yl)-2-thioxothiazolidin-4-one 14a

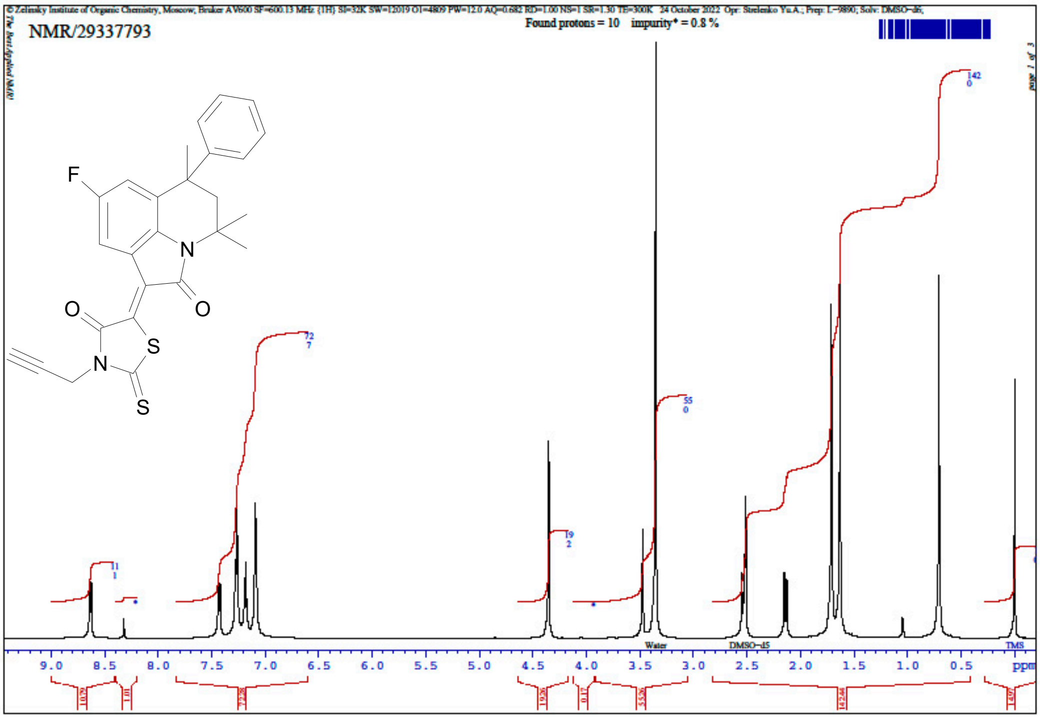

Figure S104. ^{1}H NMR spectrum of compound **14a**

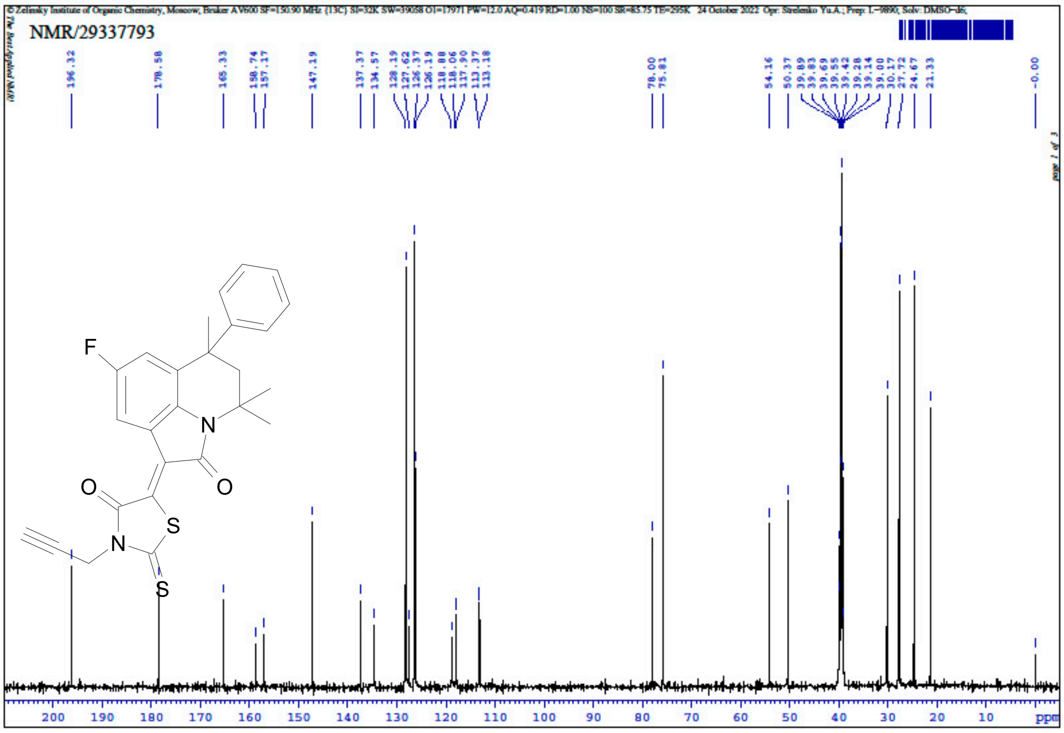

Figure S105. ^{13}C NMR spectrum of compound **14a**

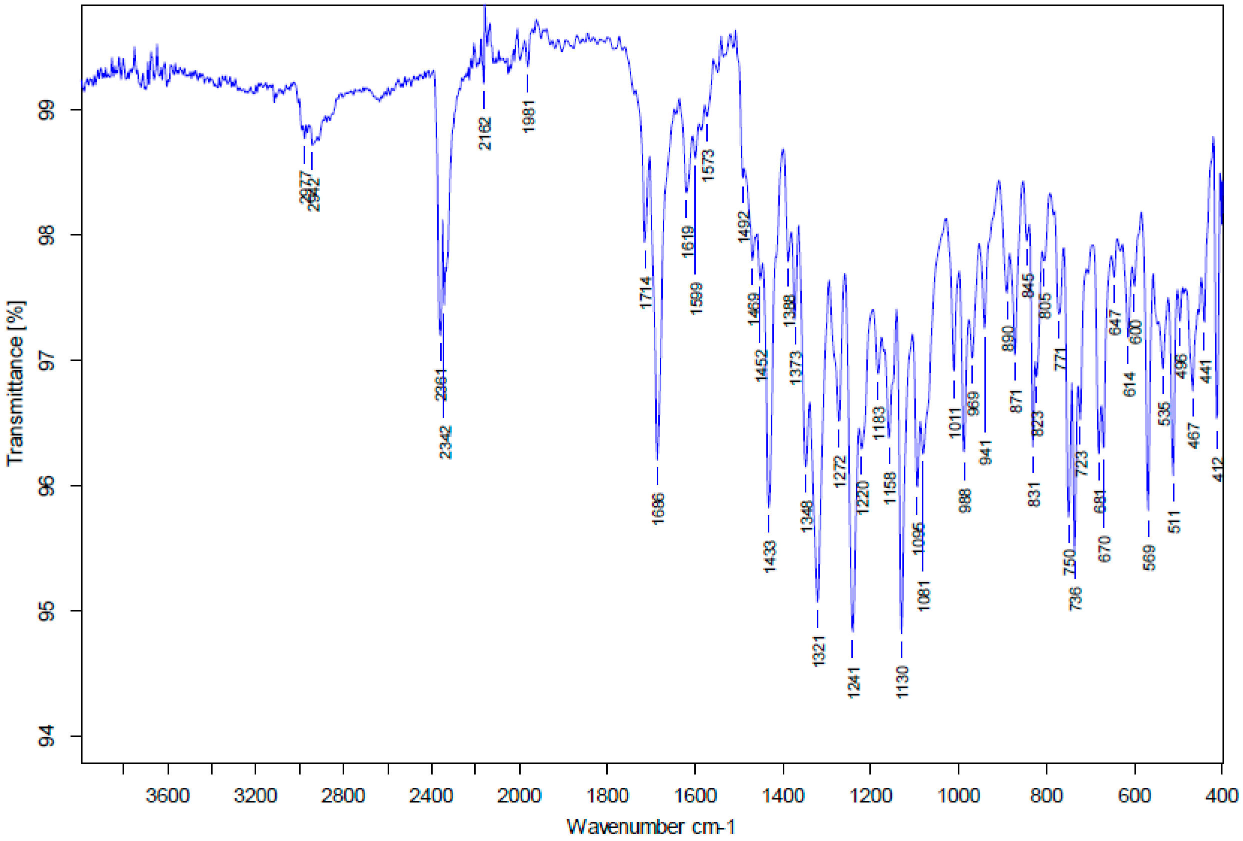

Figure S106. IR spectrum of compound **14a**

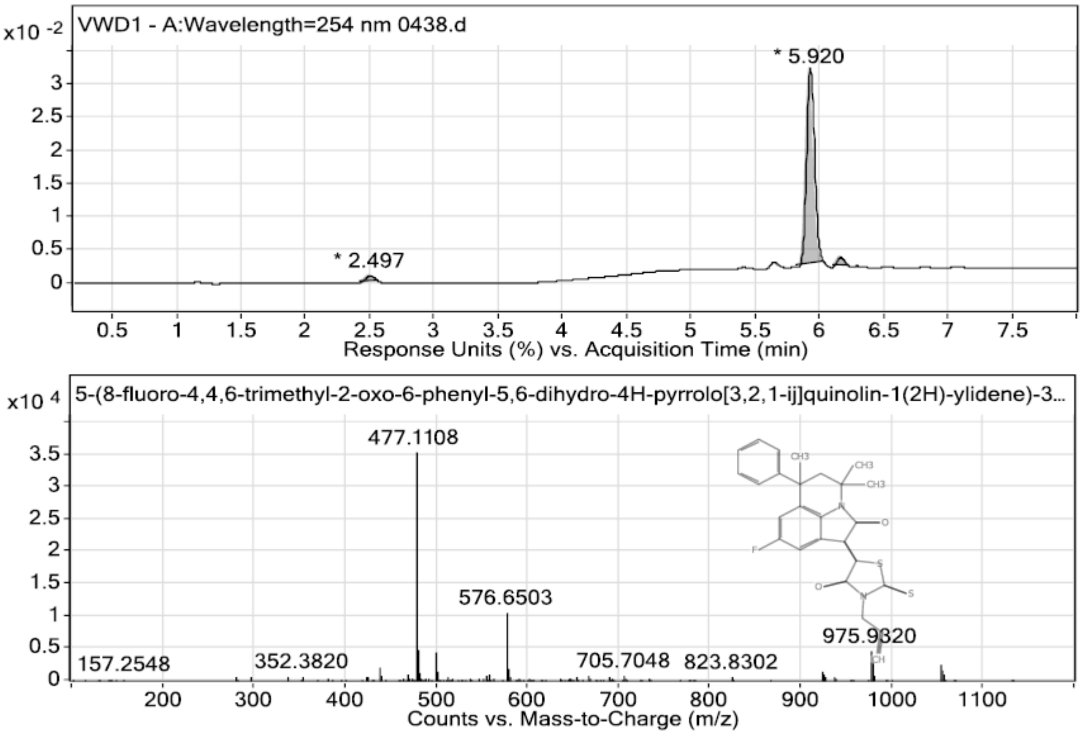

Figure S107. Data of HPLC-MS-ESI analysis of compound **14a**

^{1}H, ^{13}C NMR and data HPLC-HRMS-ESI spectra of (*Z*)-5-(6-(4-chlorophenyl)-8-fluoro-4,4,6-trimethyl-2-oxo-5,6-dihydro-4*H*-pyrrolo[3,2,1-*ij*]quinolin-1(2*H*)-ylidene)-3-(prop-2-yn-1-yl)-2-thioxothiazolidin-4-one 14b

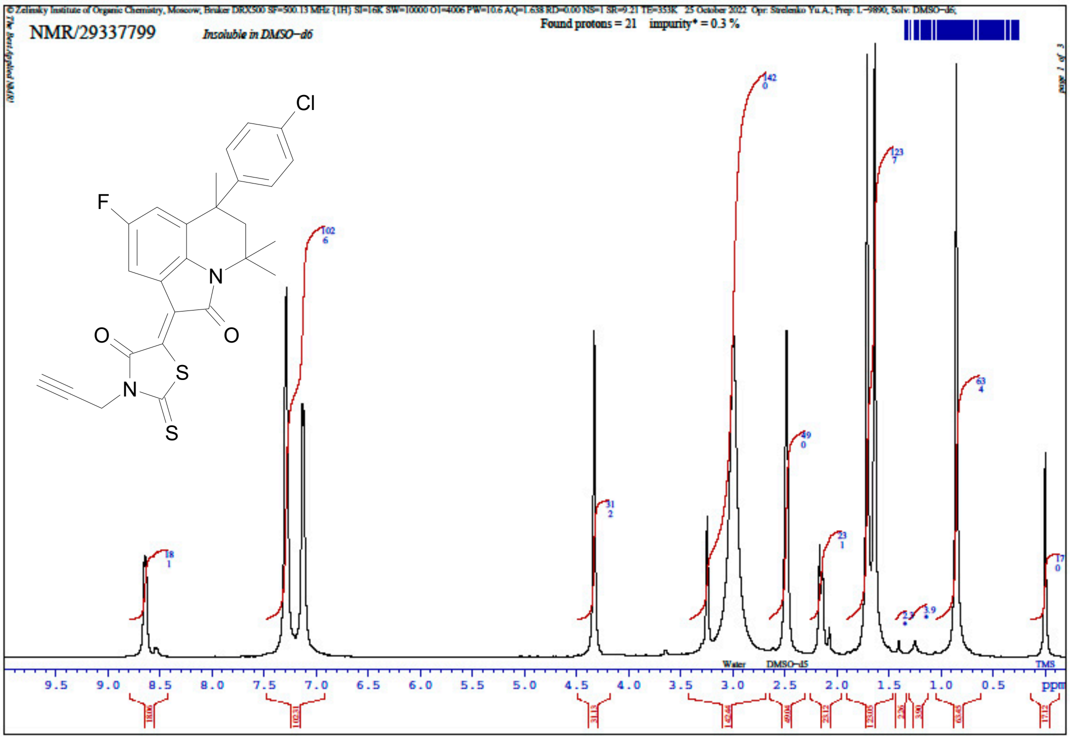

Figure S108. ^{1}H NMR spectrum of compound **14b**

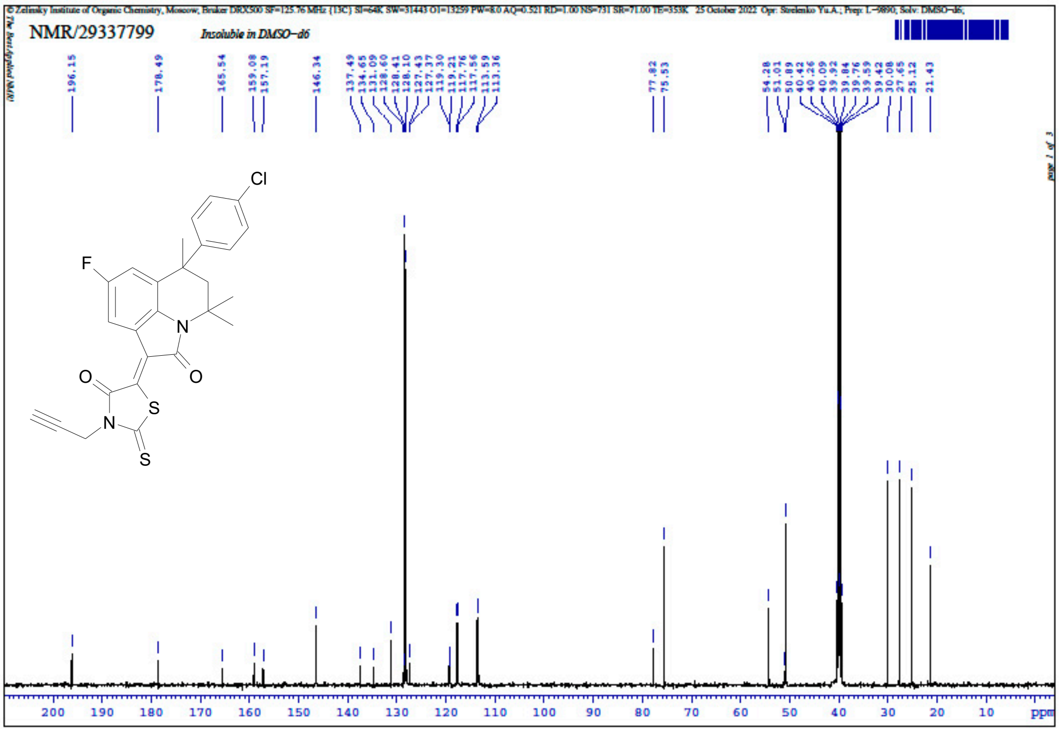

Figure S109. ^{13}C NMR spectrum of compound **14b**

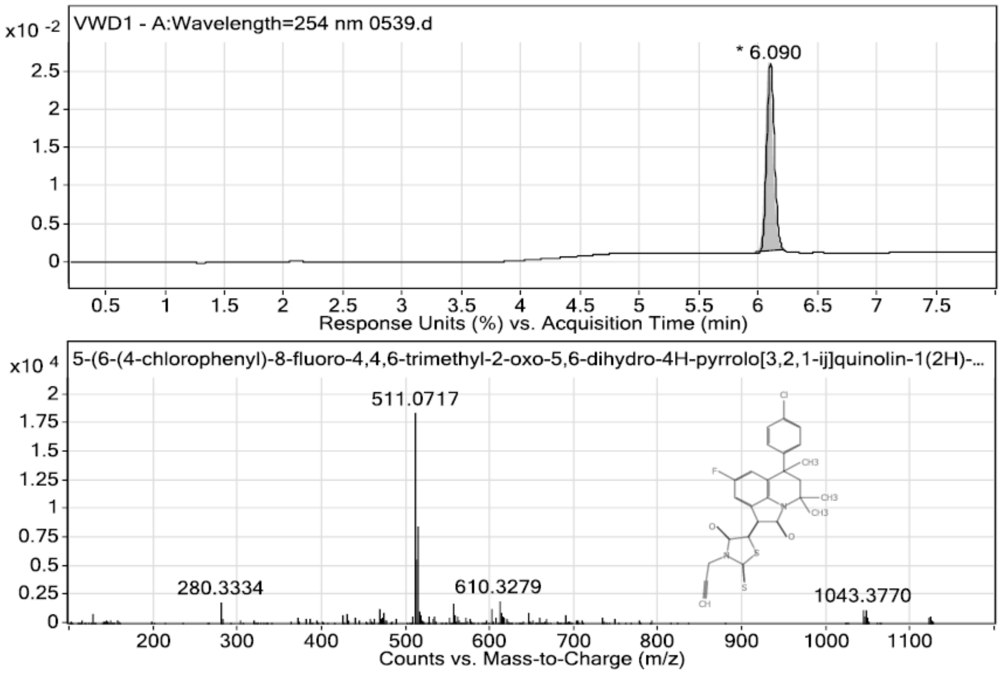

Figure S110. Data of HPLC-MS-ESI analysis of compound **14b**

^{1}H, ^{13}C NMR and data HPLC-HRMS-ESI spectra of (*Z*)-8-ethoxy-4,4,6-trimethyl-1-(5-oxo-2-thioxoimidazolidin-4-ylidene)-5,6-dihydro-4*H*-pyrrolo[3,2,1-*ij*]quinolin-2(1*H*)-one 16a

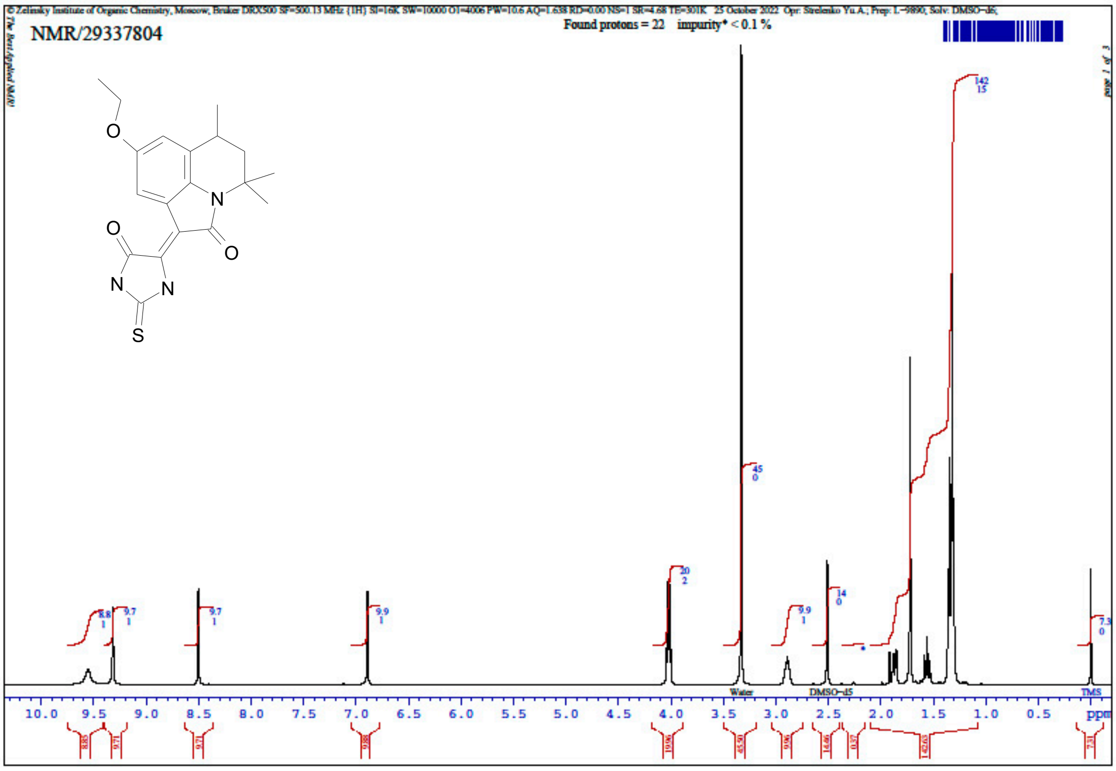

Figure S111. ^{1}H NMR spectrum of compound **16a**

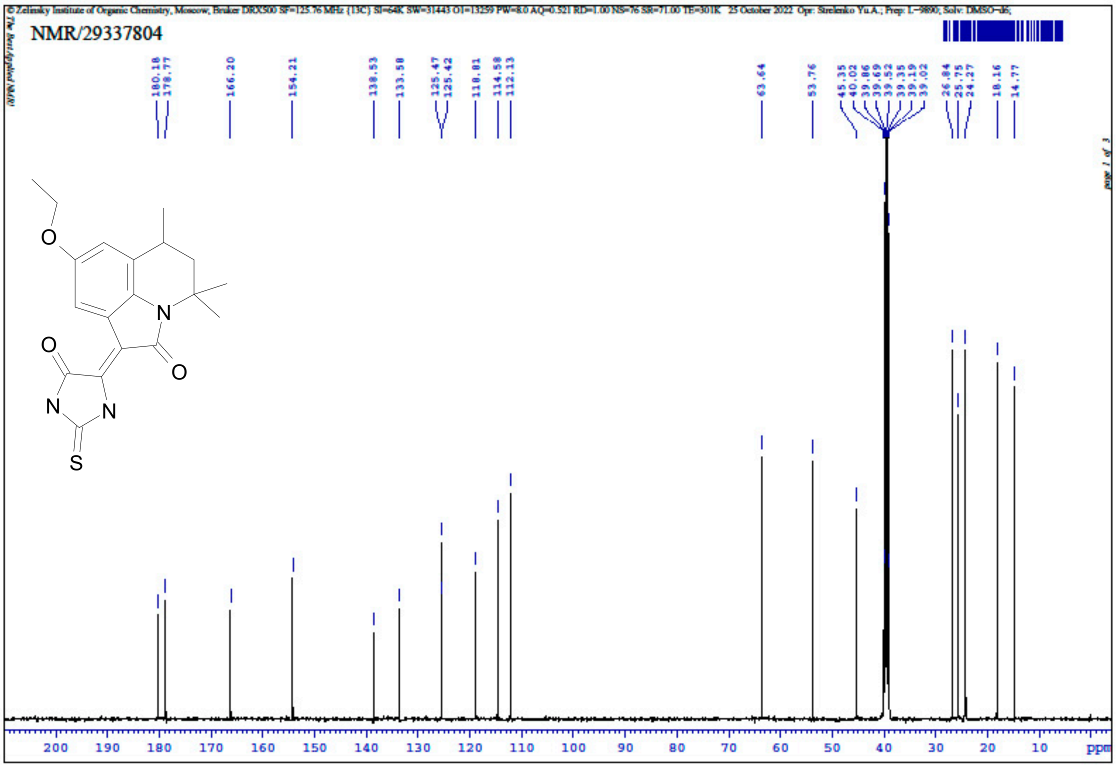

Figure S112. ^{13}C NMR spectrum of compound **16a**

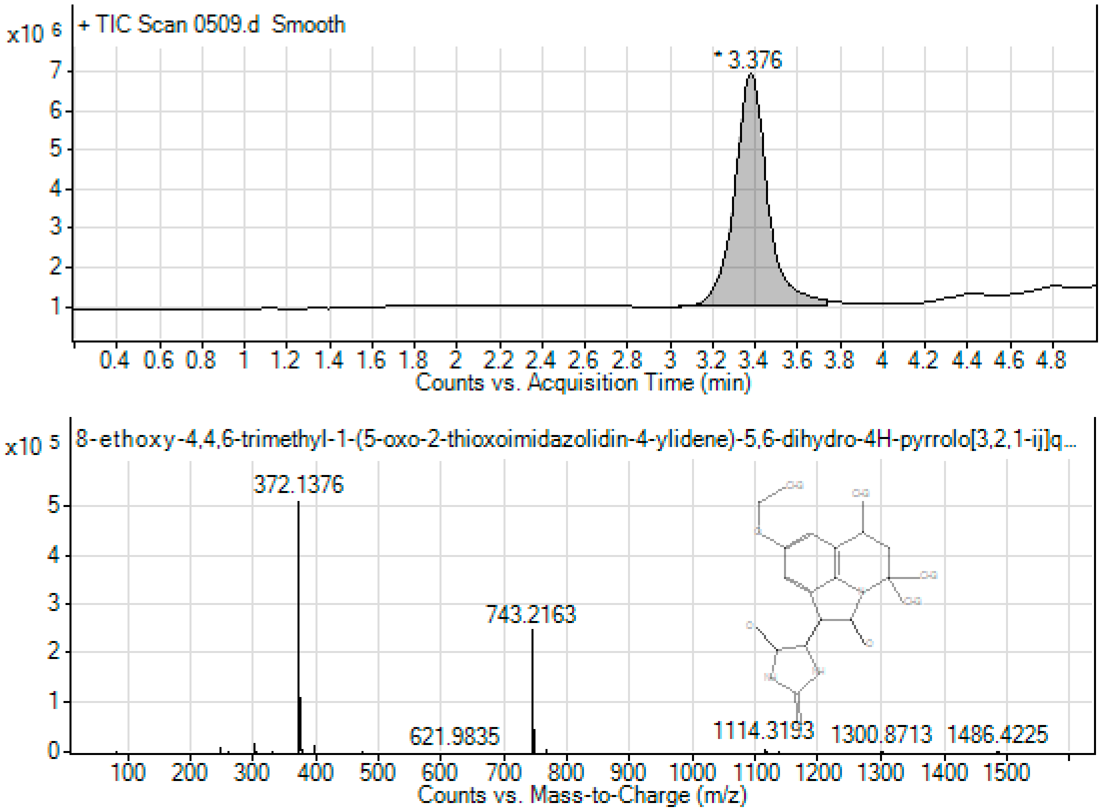

Figure S113. Data of HPLC-MS-ESI analysis of compound **16a**

¹H, ¹³C NMR, IR and data HPLC-HRMS-ESI spectra of (*Z*)-4,4,6,8-tetramethyl-1-(5-oxo-2-thioxoimidazolidin-4-ylidene)-5,6-dihydro-4*H*-pyrrolo[3,2,1-*ij*]quinolin-2(1*H*)-one 16b

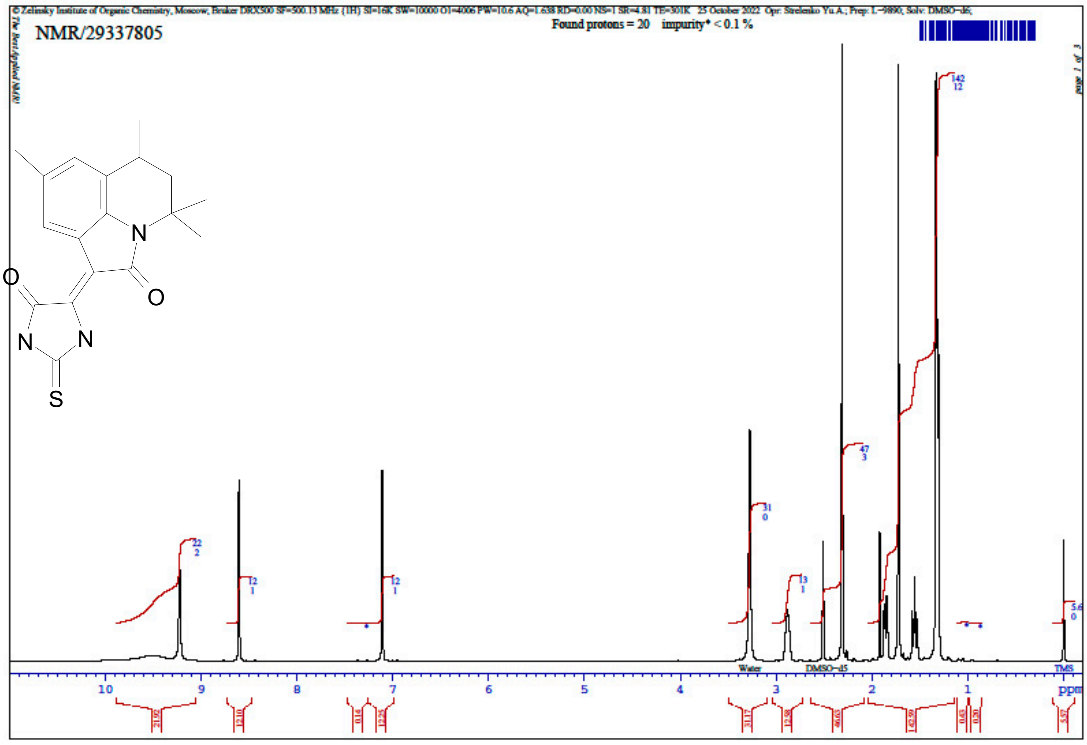

Figure S114. ¹H NMR spectrum of compound **16b**

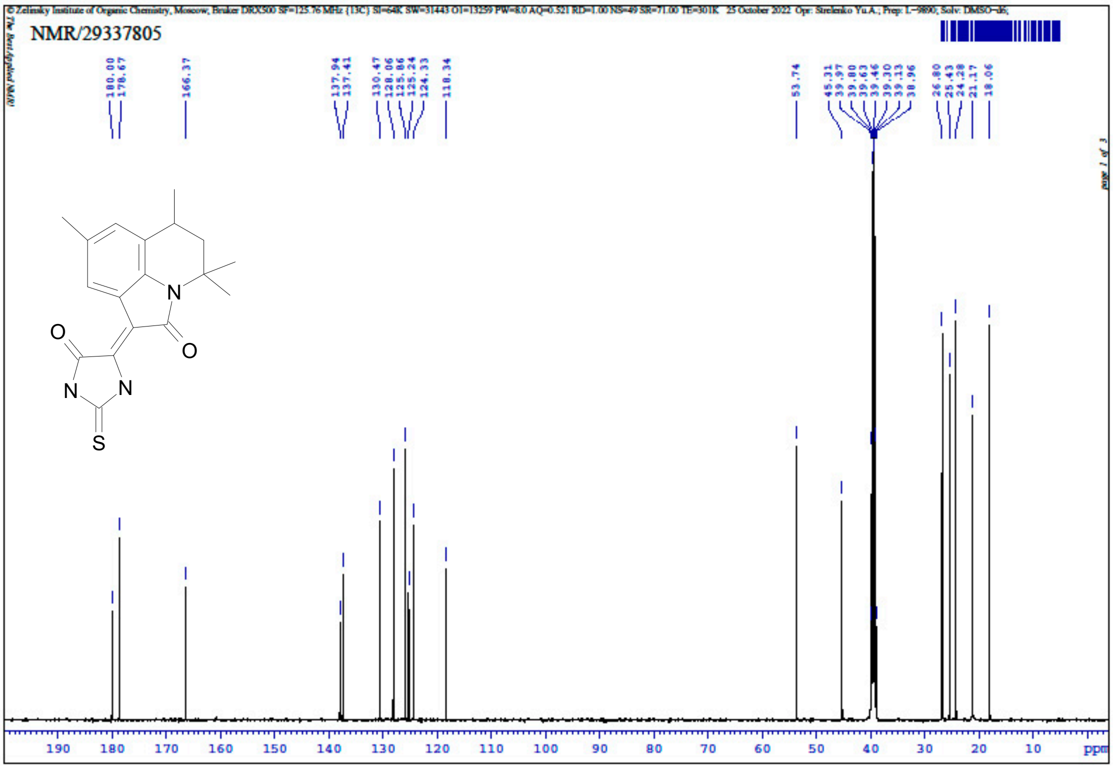

Figure S115. ^{13}C NMR spectrum of compound **16b**

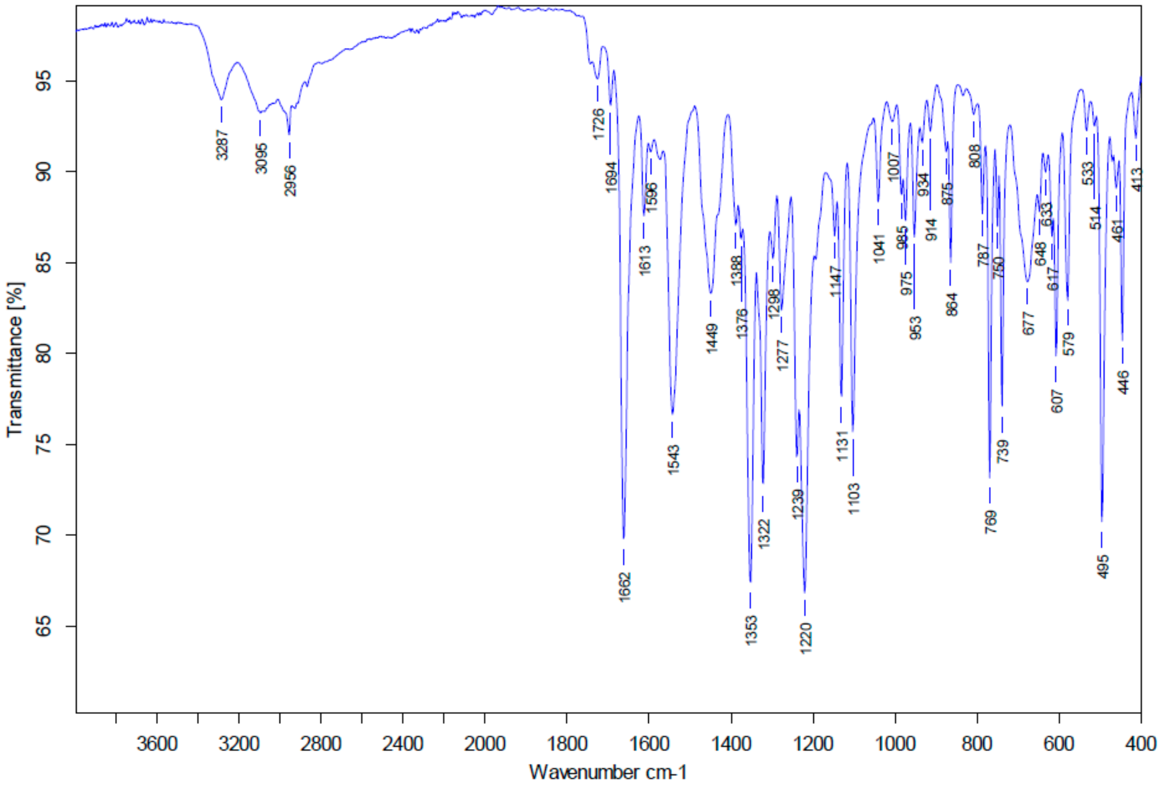

Figure S116. IR spectrum of compound **16b**

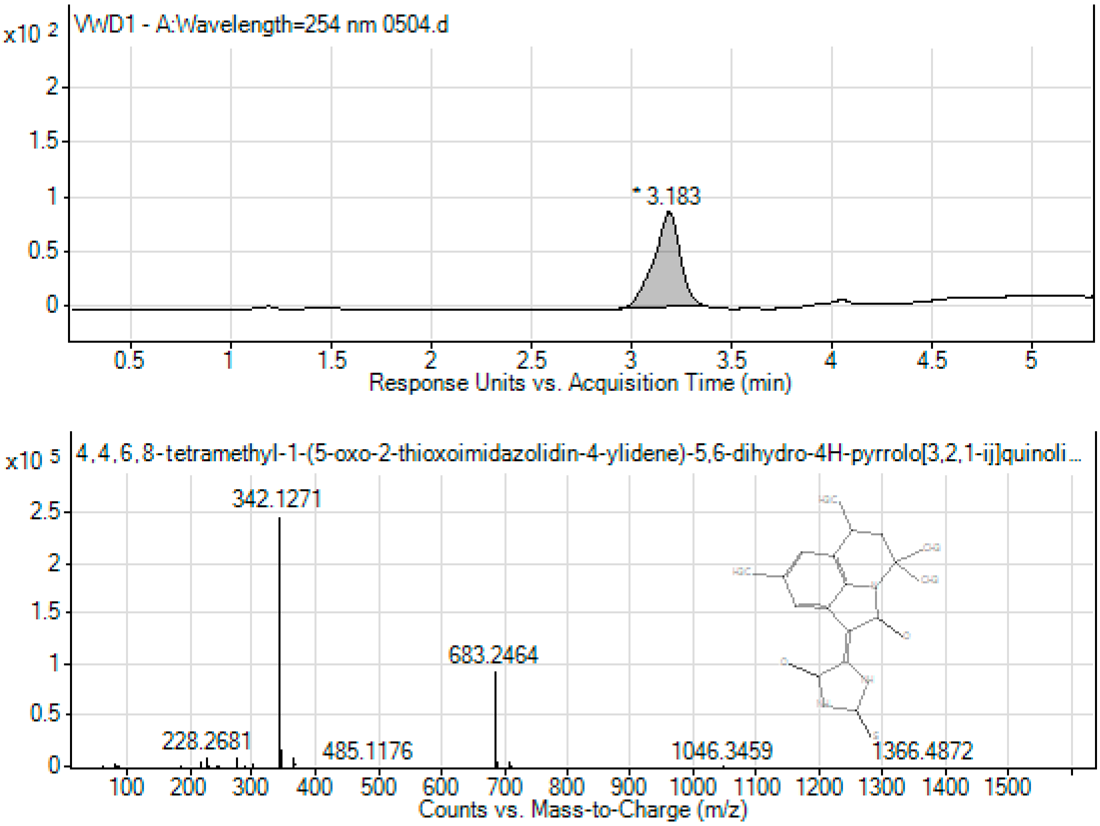

Figure S117. Data of HPLC-MS-ESI analysis of compound **16b**

^{1}H, ^{13}C NMR and data HPLC-HRMS-ESI spectra of (*Z*)-4,4,6-trimethyl-1-(5-oxo-2-thioxoimidazolidin-4-ylidene)-6-phenyl-5,6-dihydro-4*H*-pyrrolo[3,2,1-*ij*]quinolin-2(1*H*)-one 16c

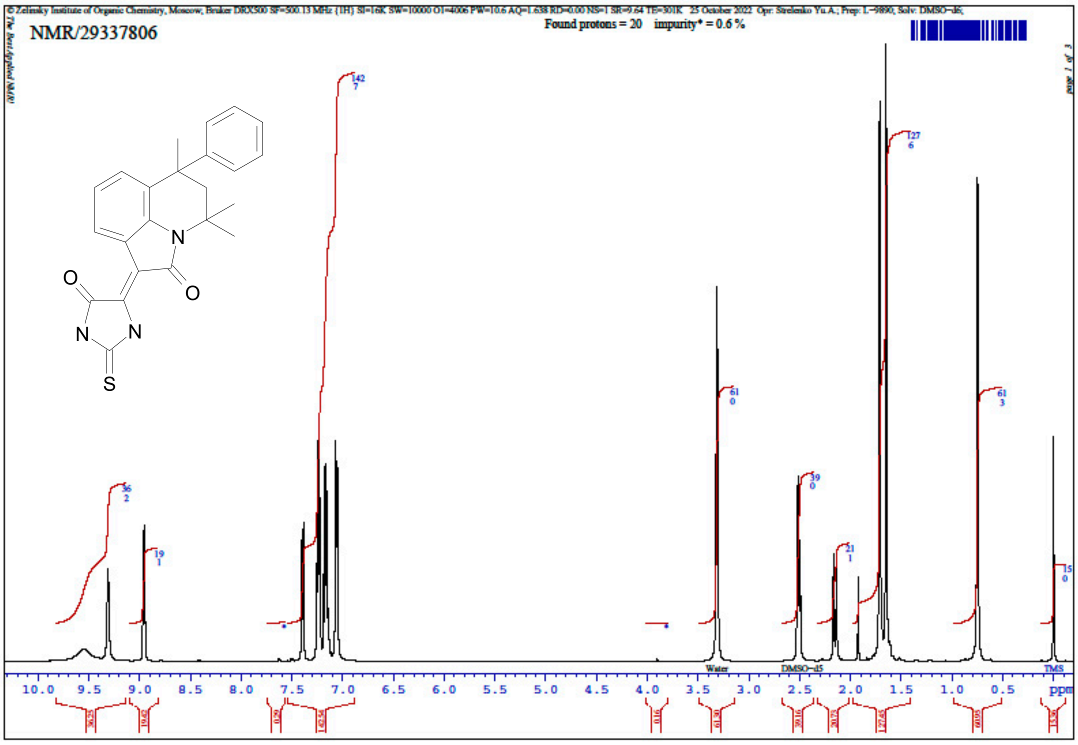

Figure S118. ^{1}H NMR spectrum of compound **16c**

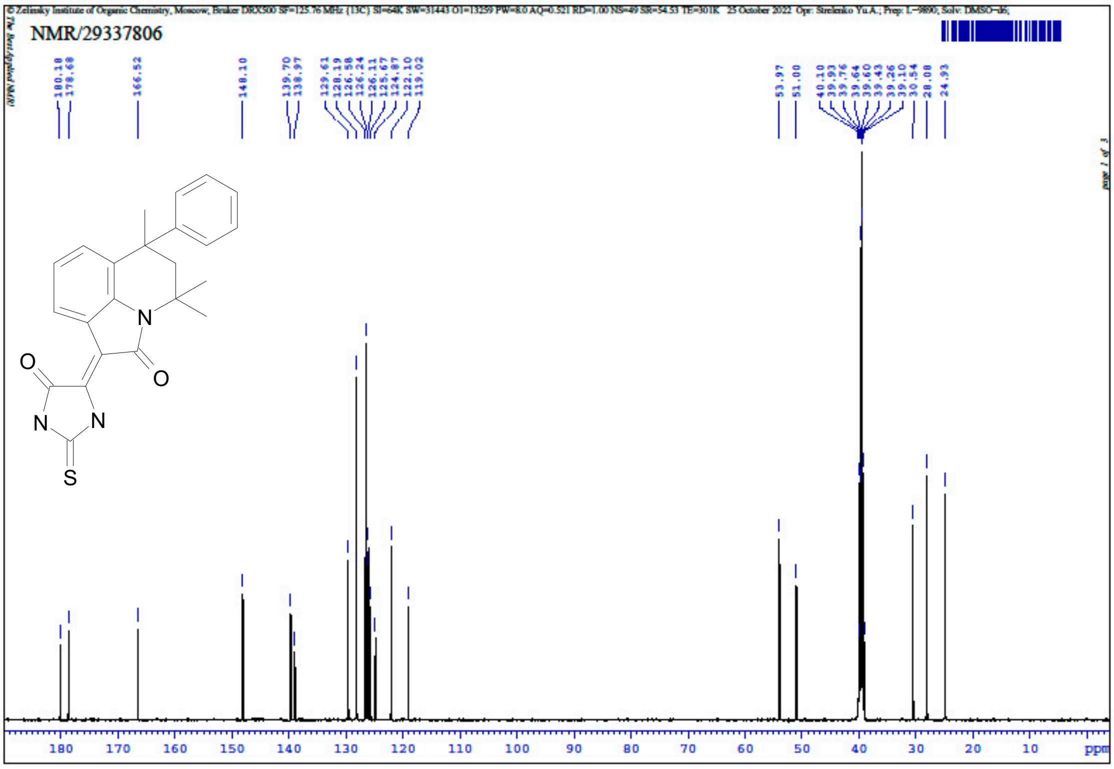

Figure S119. ^{13}C NMR spectrum of compound **16c**

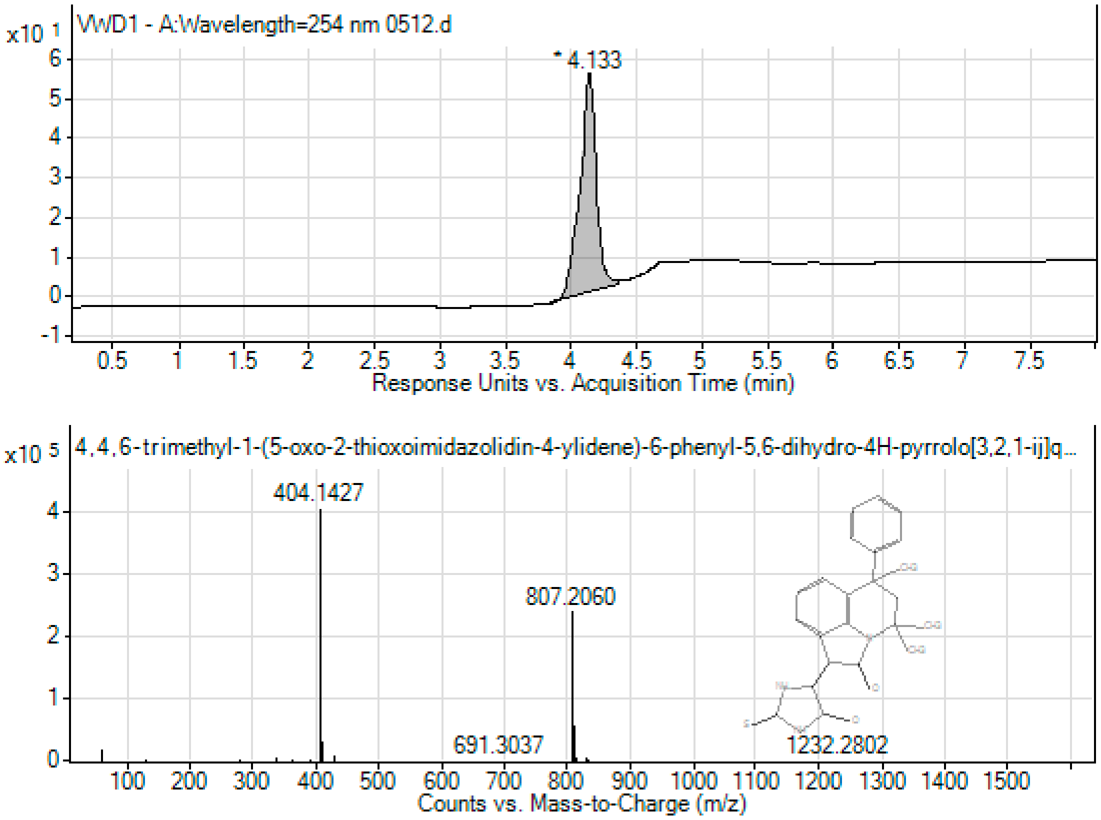

Figure S120. Data of HPLC-MS-ESI analysis of compound **16c**

^{1}H, ^{13}C NMR, IR and data HPLC-HRMS-ESI spectra of (*Z*)-8-fluoro-4,4,6-trimethyl-1-(5-oxo-2-thioxoimidazolidin-4-ylidene)-6-phenyl-5,6-dihydro-4*H*-pyrrolo[3,2,1-*ij*]quinolin-2(1*H*)-one 16d

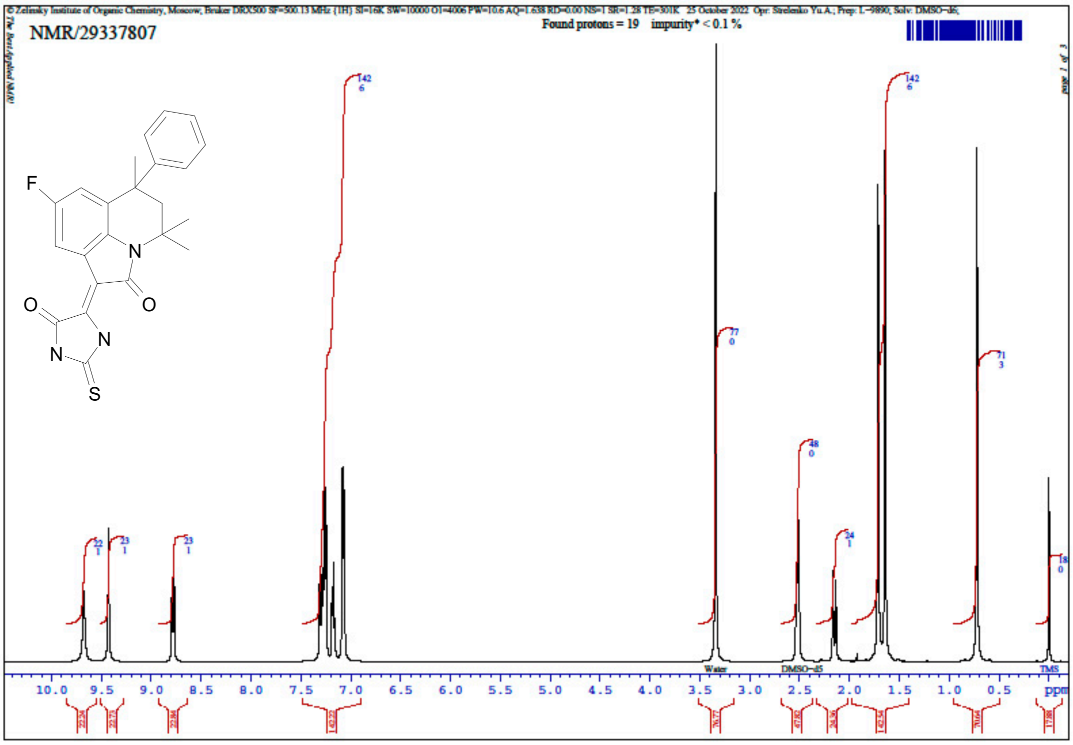

Figure S121. ^{1}H NMR spectrum of compound **16d**

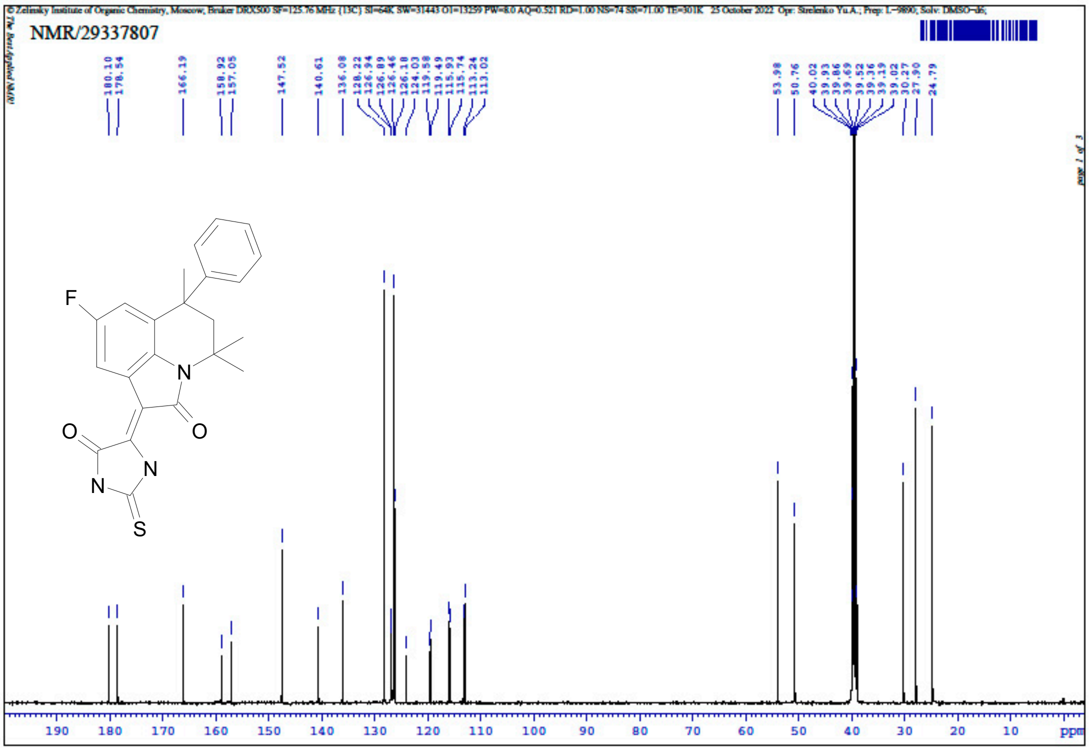

Figure S122. ^{13}C NMR spectrum of compound **16d**

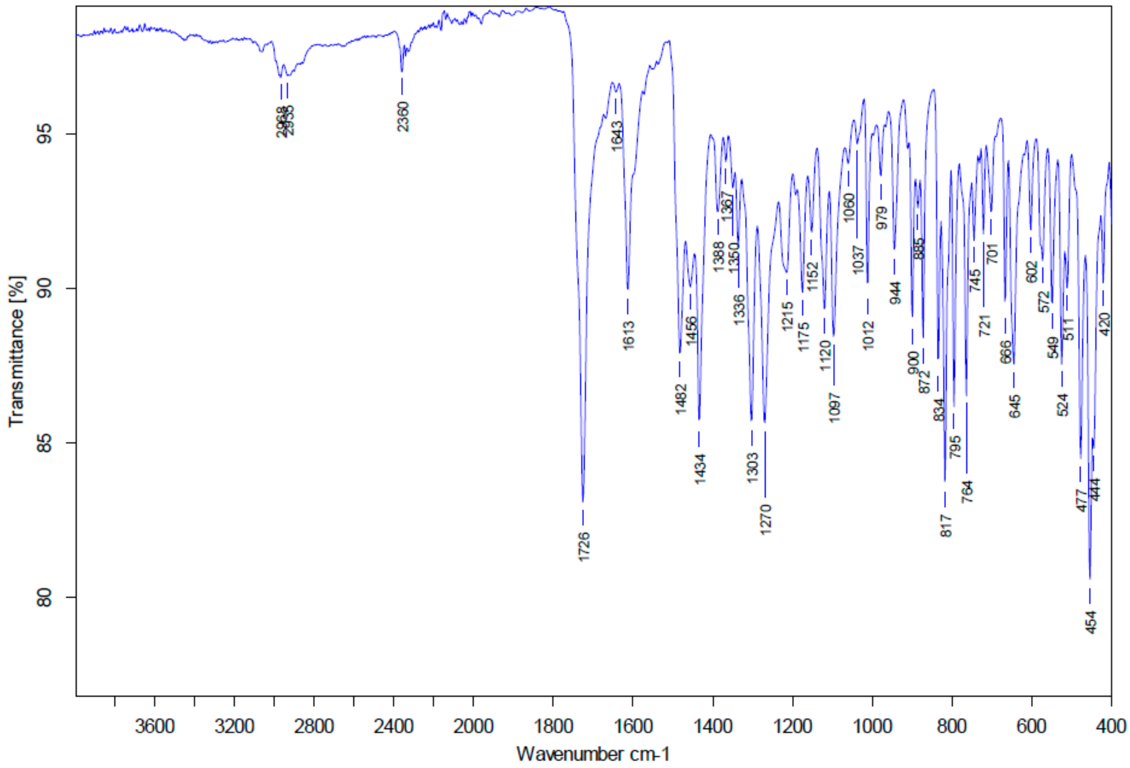

Figure S123. IR spectrum of compound **16b**

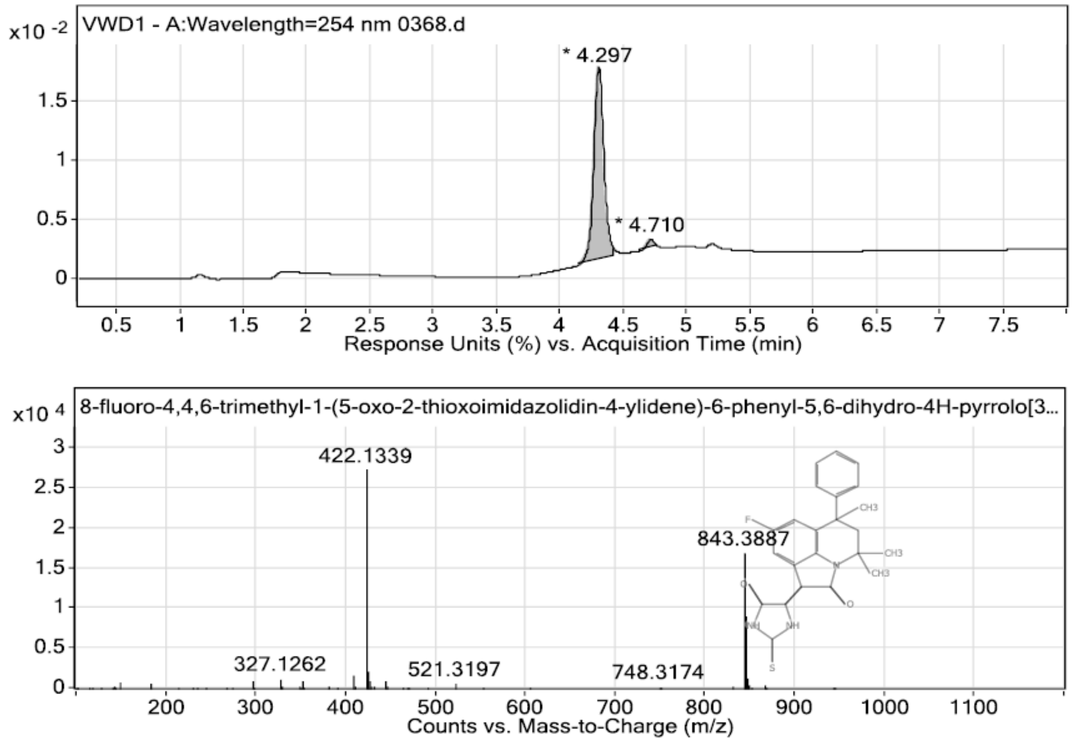

Figure S124. Data of HPLC-MS-ESI analysis of compound **16d**

^{1}H, ^{13}C NMR and data HPLC-HRMS-ESI spectra of (*Z*)-6-(4-chlorophenyl)-4,4,6,8-tetramethyl-1-(5-oxo-2-thioxoimidazolidin-4-ylidene)-5,6-dihydro-4*H*-pyrrolo[3,2,1-*ij*]quinolin-2(1*H*)-on 16e

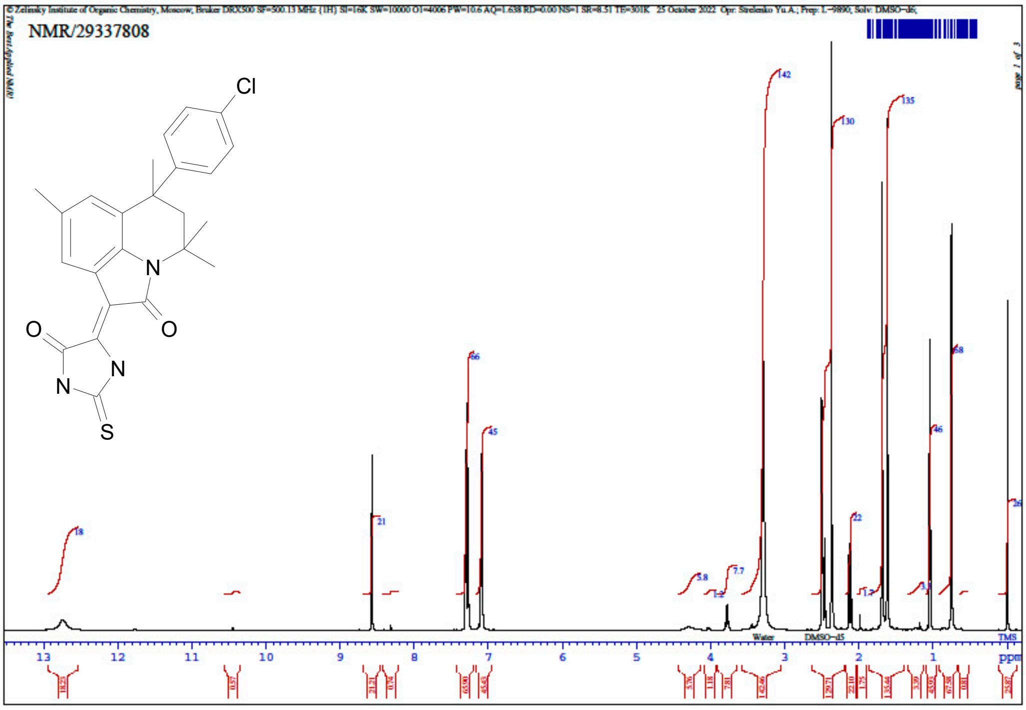

Figure S125. ^{1}H NMR spectrum of compound **16e**

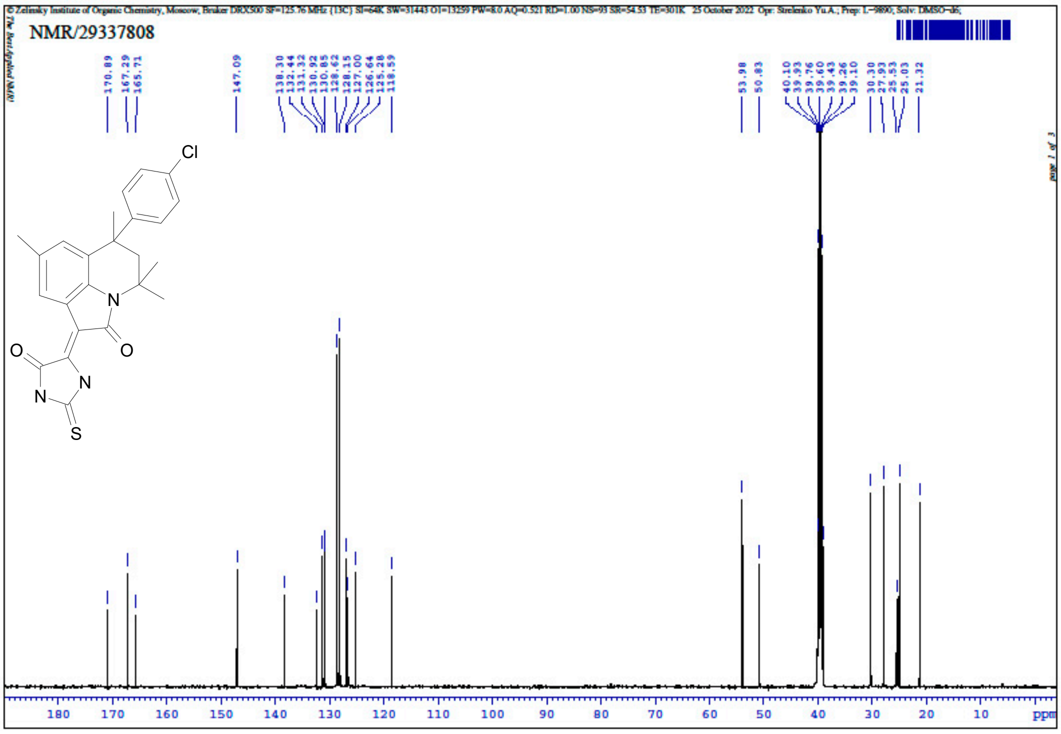

Figure S126. ^{13}C NMR spectrum of compound **16e**

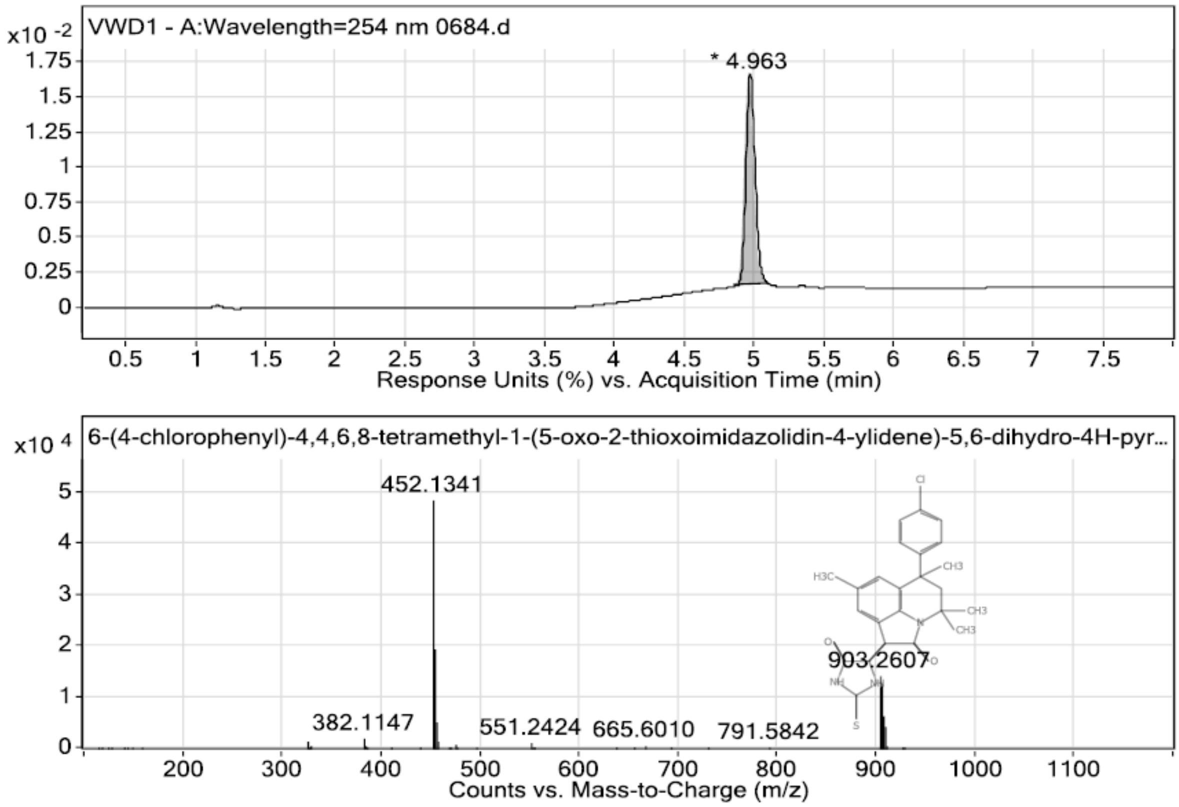

Figure S127. Data of HPLC-MS-ESI analysis of compound **16e**

Table S1. Residues participating in interaction with each of 24 tested compounds after docking into factor Xa and factor XIa active sites.

№	Interaction residues in factor Xa	Interaction residues in factor XIa
**12a**	Gly-216, Glu-217	Ser-195, Ser-214
**12b**	Gly-216	Lys-192, Gly-193, Ser-195
**12c**	Gly-216	Gly-216
**12d**	Gln-61, Gly-216	His-57
**12e**	Gly-216	Met-96
**12f**	Ala-190	His-57, Met-96, Glu-98
**12g**	Glu-217	His-57, Tyr-59
**12h**	Arg-222	Lys-192
**12i**	Glu-217	Tyr-59, Val-227
**12j**	Glu-217	Tyr-59
**12k**	Glu-217, Arg-222	His-57, Tyr-59
**12l**	Gly-216, Arg-222	Cys-40, Tyr-59
**12m**	Ala-190, Gln-192, Trp-215	Met-96, Glu-98
**12n**	Tyr-99	Arg-37, Cys-40, Ala-190
**12o**	Trp-215	-
**12p**	Gly-216	Lys-192
**12q**	Glu-217	His-57, Trp-215
**14a**	Glu-217	His-57, Trp-215
**14b**	Gly-216	Lys-192, Gly-193, Ser-195
**16a**	Tyr-99, Glu-217, Arg-222	His-57, Met-96, Ala-190, Ser-195
**16b**	Arg-222	Met-96, Glu-98
**16c**	Gly-216	Ser-195
**16d**	Phe-174	Cys-191, Gly-193
**16e**	Tyr-99	His-57, Tyr-59, Cys-191

Table S2. Crystal complexes with actives used in validation of docking protocol for factor Xa and factor XIa.

Factor Xa				Factor XIa			
PDB ID	Ligand ID	SOL score, kcal/mol	IC_{50}, nM	PDB ID	Ligand ID	SOL score, kcal/mol	IC_{50}, nM (Ki, nM)
2P3T	993	-6.89	0.06	4CRA	XJ8	-5.10	(60)
2P94	ME4	-7.39	0.67	4CRB	7P0	-3.70	(1.6)
2VH6	GSV	-6.78	0.2	4CRG	J4X	-5.98	<140
2VVC	LZF	-6.42	3	4CRE	MVN	-4.89	$>1.1 \cdot 10^5$
2W26	RIV	-6.87	0.4	-	BMS-654457 [1]	-6.04	(0.2)
2Y7X	MZA	-7.17	1.3	-	BMS-962212 [2]	-6.0	(0.7)
3IIT	D14	-6.64	9.5	-	3 [3]	-5.76	1.0
3TK6	D46	-6.95	1.7	-	4a [4]	-6.52	(0.31)
4ZH8	4O4	-7.09	-	-	6b [5]	-5.35	(3.7)
4ZHA	4O5	-5.77	-	-	8 [6]	-5.85	(2.0)

Structures of factor XIa inhibitors 3, 4a, 6b, and 8 can be found in Table 3 in ref. [7]; references to the original articles about these factor XIa inhibitors are presented in column "Ligand ID". For all cases of factor Xa presented in Table S2, and for PDB complexes of factor XIa 4CRA, 4CRB, 4CRG, 4CRE, and for the PDB complex 4CRC, the root-mean-square deviation, RMSD, between all atoms of the crystallized native ligand pose and the docked ligand pose corresponding to the lowest energy of the pro-tein-ligand complex, is less than 2.0 Å.

References

1. Wong P.C., Quan M.L., Watson C.A., Crain E.J., Harpel M.R., Rendina A.R., Luettgen J.M., Wexler R.R., Schumacher W.A., Seiffert D.A. In vitro, antithrombotic and bleeding time studies of BMS-654457, a small-molecule, reversible and direct inhibitor of factor XIa // J. Thromb. Thrombolysis. 2015. Vol. 40, № 4. P. 416–423.
2. Pinto D.J.P., Orwat M.J., Smith L.M., Quan M.L., Lam P.Y.S., Rossi K.A., Apedo A., Bozarth J.M., Wu Y., Zheng J.J., Xin B., Toussaint N., Stetsko P., Gudmundsson O., Maxwell B., Crain E.J., Wong P.C., Lou Z., Harper T.W., et al. Discovery of a Parenteral Small Molecule Coagulation Factor XIa Inhibitor Clinical Candidate (BMS-962212). // J. Med. Chem. 2017. Vol. 60, № 23. P. 9703–9723.
3. Fjellstrom O., Akkaya S., Beisel H.-G., Eriksson P.-O., Erixon K., Gustafsson D., Jurva U., Kang D., Karis D., Knecht W., Nerme V., Nilsson I., Olsson T., Redzic A., Roth R., Sandmark J., Tigerstrom A., Oster L. Creating novel activated factor XI inhibitors through fragment based lead generation and structure aided drug design. // PLoS One. United States, 2015. Vol. 10, № 1. P. e0113705.
4. Hu Z., Wang C., Han W., Rossi K.A., Bozarth J.M., Wu Y., Sheriff S., Myers Jr. J.E., Luettgen J.M., Seiffert D.A., Wexler R.R., Quan M.L. Pyridazine and pyridazinone derivatives as potent and selective factor XIa inhibitors // Bioorg. Med. Chem. Lett. 2018/03/05. 2018. Vol. 28, № 6. P. 987–992.
5. Corte J.R., Fang T., Hangeland J.J., Friends T.J., Rendina A.R., Luettgen J.M., Bozarth J.M., Barbera

F.A., Rossi K.A., Wei A., Ramamurthy V., Morin P.E., Seiffert D.A., Wexler R.R., Quan M.L. Pyridine and pyridinone-based factor XIa inhibitors. // Bioorg. Med. Chem. Lett. 2015. Vol. 25, № 4. P. 925–930.

6. Corte J.R., Fang T., Pinto D.J.P., Orwat M.J., Rendina A.R., Luettgen J.M., Rossi K.A., Wei A., Ramamurthy V., Myers J.E., Sheriff S., Narayanan R., Harper T.W., Zheng J.J., Li Y.-X., Seiffert D.A., Wexler R.R., Quan M.L. Orally bioavailable pyridine and pyrimidine-based Factor XIa inhibitors: Discovery of the methyl N-phenyl carbamate P2 prime group. // Bioorg. Med. Chem. 2016. Vol. 24, № 10. P. 2257–2272.

7. Podoplelova N.A., Sulimov V.B., Tashchilova A.S., Ilin I.S., Panteleev M.A., Ledeneva I.V., Shikhaliev K.S. Blood coagulation in the 21st cetrury: existing knowledge, current strategies for treatment and perspective // Pediatr. Hematol. Immunopathol. 2020. Vol. 19, № 1. P. 139–157 (in Russian).
